# Polyprenylated Acylphloroglucinols With Different Carbon Skeletons From the Fruits of *Garcinia multiflora*

**DOI:** 10.3389/fchem.2021.756452

**Published:** 2021-10-26

**Authors:** Haida Teng, Qingqing Li, Ziyu Ma, Xueni Li, Wenli Xie, Yu Chen, Guangzhong Yang

**Affiliations:** ^1^ College of Chemistry and Material Sciences, South-Central University for Nationalities, Wuhan, China; ^2^ School of Pharmaceutical Sciences, South-Central University for Nationalities, Wuhan, China

**Keywords:** *Garcinia multiflora*, *Garcinia*, polyprenylated acylphloroglucinols, NMR calculations, antiproliferative activity

## Abstract

Eleven new polycyclic polyprenylated acylphloroglucinols (PPAPs, **1**–**11**) and three new monocyclic polyprenylated acylphloroglucinols (MPAPs, **12**–**14**), together with ten known analogues were isolated from the fruits of *Garcinia multiflora*. These PPAPs belong to three types including the bicyclic polyprenylated acylphloroglucinols (BPAPs), the caged PPAPs, and the complicated PPAPs. Their structures and absolute configurations were determined through HRESIMS, NMR spectroscopy data, electronic circular dichroism (ECD) calculations, and gauge-independent atomic orbital (GIAO) NMR calculations with DP4+ analyses. Moreover, compounds **2** and **7** exhibited moderate cytotoxicity against three human cancer lines (MCF-7, T98, and HepG2) with IC_50_ values ranging from 9.81 ± 1.56 to 17.00 ± 2.75 μM.

## Introduction

The plants of Guttiferae and Hypericaceae family mainly including the genus *Garcinia* and *Hypericum* are well-known for producing structurally diverse and biologically polycyclic polyprenylated acylphloroglucinols (PPAPs). Previous phytochemical studies indicated that more than 500 PPAPs have been isolated the plants of Guttiferae family with diverse structural scaffolds including the bicyclic polyprenylated acylphloroglucinols (BPAPs), the caged PPAPs, and the complicated PPAPs. BPAPs comprise approximate 60% of PPAPs which share a bicyclo [3.3.1]nonane-2,4,9-trione core (Yang et al., 2018). Depending on the relative position of the acyl group connected to the phloroglucinol core, BPAPs are categorized into type A or B. Type A BPAPs contain a C-1 acyl group which is next to C-8 quaternary center, while type B BPAPs contain a C-3 acyl group (Ciochina and Grossman, 2006). BPAPs have attracted noticeable attention from both natural product and medicinal chemists due to their fascinating chemical structures and intriguing biological activities (Phang et al., 2020).

**FIGURE 1:**
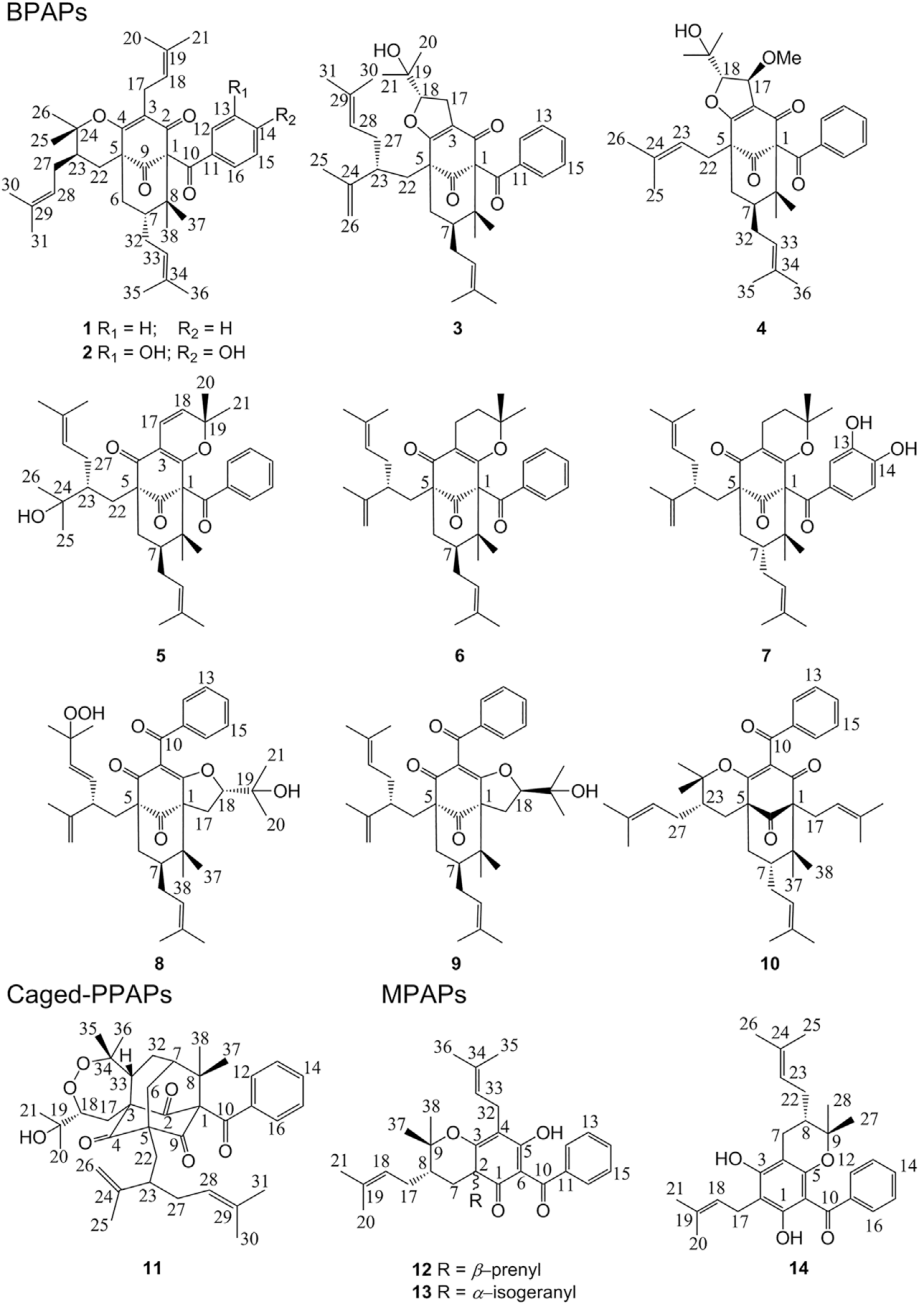
Chemical structures of PAPs (**1**–**14**) from *G*. *multiflora*.

*Garcinia multiflora* Champ belongs to the genus of *Garcinia*, which is mainly distributed in the southern region of China. The fruit can be eaten raw when it is ripe, which possesses a high nutritional value and contains pharmacologically active compounds (Liu et al., 2017a; Xu et al., 2017). Previous research results indicated that the fruits, stems, roots, leaves, and twigs of *G. multiflora* might be an important source of PPAPs (Chien et al., 2008; Chen et al., 2009; Liu et al., 2010; Ting et al., 2012; Ting et al., 2014; Fan et al., 2015; Fu et al., 2015; Tian et al., 2016; Fan et al., 2016; Cheng et al., 2018a; Cheng et al., 2018b; Wang et al., 2018). In our previous study, four new complicated PPAPs with new carbon skeletons tricyclo [3.3.1.1.^4,8^]decane, 14 new-caged PPAPs, and two new cyclohexanone—monocyclic polycyclic polyprenylated acylphloroglucinols (MPAPs) from the fruits of *G. multiflora* were reported (Chen et al., 2019a; Chen et al., 2019b; Teng et al., 2019; Teng et al., 2020). As part of ongoing research, phytochemical investigations of an extract of the fruits of *G. multiflora* afforded 11 new PPAPs and three new MPAPs (Figure 1). This study reported the isolation, structure identification, and biological activity of these compounds.

## Materials and Methods

### General Experimental Procedures

Optical rotations were determined in MeOH using an Autopol IV polarimeter (Rudolph Research Analytical, Hackettstown, NJ, United States). UV spectra were obtained using a UH5300 UV-VIS Double-Beam spectrophotometer (Hitachi Co., Tokyo, Japan). 1D and 2D NMR spectra were recorded with a Bruker AVANCE IIITM 600 MHz spectrometer (Bruker, Ettlingen, Germany) in CDCl_3_ using TMS as internal standard. HR-ESIMS data were obtained using a Thermo Fisher Scientific Q Exactive Orbitrap LC-MS/MS System (Thermo Fisher Scientific, Waltham, MA, United States). An Ultimate 3000 HPLC system (Dionex Co., Sunnyvale, CA, United States) with an Ultimate 3,000 pump and an Ultimate 3,000 Variable Wavelength Detector was used to perform semipreparative HPLC, with a Nacalai Tesque 5C_18_-MS-II column (250 × 10 mm, 5 μm). Silica gel for CC (200–300 mesh and 300–400 mesh) was obtained from the Qingdao Hai Yang Chemical Group Co. (Qingdao, China). The human tumor cell lines HepG2, T98G, and MCF-7 were purchased from the cell bank of the Chinese Academy of Sciences (Shanghai, China). Cisplatin was purchased from Sigma-Aldrich (Saint Louis, MO, United States). The Cell Counting Kit (CCK-8) was purchased from Beyotime Biotechnology (Shanghai, China). Dulbecco’s modified Eagle’s medium (DMEM) and penicillin–streptomycin solution were purchased from GE Healthcare Life Sciences (Logan, UT, United States). Fetal bovine serum (FBS) was purchased from Gibco and Life Technologies (Grand Island, NY, United States). Reagent grade DMSO was purchased from Vetec and Sigma Chemical Co. (St Louis, MO, United States). The absorbance was recorded with a Multiskan GO microplate reader (Thermo Fisher Scientific, Inc., Waltham, MA, United States). The organic solvents were obtained from Sinopharm Chemical Reagent Co., Ltd. (Shanghai, China).

### Plant Material

The fruits of *G. multiflora* were purchased from Nanning, Guangxi Zhuang Autonomous Region, P. R. China, and identified by Prof. Hongli Teng, Guangxi Zhuang Medicine International Hospital. The voucher specimen (2014091201) was deposited in the herbarium of School of Pharmaceutical Sciences, South Central University for Nationalities.

### Extraction and Isolation

The dried fruits of *G. multiflora* Champ (5.2 kg) were powdered and extracted with 95% EtOH at room temperature for three times (each times for 24 h) to obtain EtOH extract 2.21 kg and then successively partitioned with petroleum ether (PE), EtOAc, and *n*-BuOH to get PE extract 125 g, EtOAc extract 166 g, and *n*-BuOH extract 80 g. The PE extract (125 g) was chromatographed on a silica gel column (200–300 mesh) eluted successively with PE/acetone gradient (50:1, 25:1, 10:1, 7:3, 1:1, and 0:1) to obtain six fractions (Fr. 1-Fr. 6). Fr. 2 (42.5 g) was chromatographed on a silica gel column (200–300 mesh) eluted successively with PE/CH_2_Cl_2_ gradient (10:1 to 0:1) to obtain 11 fractions (Fr. 2.1–Fr. 2.11). Fr. 2.7 (9.2 g) was separated on an ODS column, eluted with H_2_O-MeOH (7:3 to 0:1), and repeated semi-preparative HPLC to afford compounds **20** (20.0 mg; CH_3_CN-H_2_O, 78:22, *t*_*R*_ 31.7 min); **21** (3.2 mg; CH_3_CN-H_2_O, 84:16, *t*_*R*_ 91.5 min); **23** (3.4 mg; CH_3_CN-H_2_O, 85:15, *t*_*R*_ 26.3 min); and **24** (15.9 mg; CH_3_CN-H_2_O, 85:15, *t*_*R*_ 56.6 min). Fr. 2.9 (2.3 g) was further separated by silica gel CC (PE/CH_2_Cl_2_/MeOH, 10:1:0.1 to 0:1:0.1) and repeated semi-preparative HPLC to afford compounds **10** (1.2 mg; CH_3_CN-H_2_O, 93:7, *t*_*R*_ 20.0 min); **13** (2.2 mg; CH_3_CN-H_2_O, 93:7, *t*_*R*_ 54.0 min); and **17** (1.0 mg; CH_3_CN-H_2_O, 90:10, *t*_*R*_ 25.0 min). Fr. 2.10 (4.6 g) was further purified by semi-preparative HPLC (CH_3_CN-H_2_O, 87:13) to afford compounds **1** (2.2 mg) at *t*_*R*_ 55.1 min, **12** (1.7 mg) at *t*_*R*_ 64.2 min, and **15** (7.5 mg) at *t*_*R*_ 70.8 min. Fr. 3 (31.0 g) was subjected to repeated silica gel CC with PE/CH_2_Cl_2_ (50:1 to 0:1), ODS CC with H_2_O-MeOH (7:3 to 0:1), and semi-preparative HPLC to afford compounds **3** (1.4 mg; MeOH-H_2_O, 90:10, *t*_*R*_ 18.0 min); **5** (2.0 mg; MeOH-H_2_O, 93:7, *t*_*R*_ 12.9 min); **8** (2.6 mg; CH_3_CN-H_2_O, 75:25, *t*_*R*_ 40.0 min); **9** (15.1 mg; CH_3_CN-H_2_O, 93:7, *t*_*R*_ 16.5 min); **16** (7.8 mg; CH_3_CN-H_2_O, 80:20, *t*_*R*_ 19.2 min); and **18** (5.0 mg; CH_3_CN-H_2_O, 90:10, *t*_*R*_ 17.5 min). The EtOAc extract (166 g) was chromatographed on a silica gel column (200–300 mesh) eluted successively with PE/EtOAc gradient (20:1 to 0:1) to obtain nine fractions (Fr. 1–Fr. 9). Fr. 1 (5.7 g) was subjected to ODS CC with H_2_O-MeOH (7:3 to 0:1), a silica gel CC with PE/CH_2_Cl_2_ (10:1 to 0:1), and semi-preparative HPLC to afford compounds **4** (2.3 mg; CH_3_CN-H_2_O, 85:15, *t*_*R*_ 20.1 min); **6** (11.4 mg; CH_3_CN-H_2_O, 87.7:12.3, *t*_*R*_ 59.3 min); and **14** (4.3 mg; CH_3_CN-H_2_O, 85:15, *t*_*R*_ 27.9 min). Fr. 2 (15.6 g) was subjected to a silica gel CC with PE/CH_2_Cl_2_ (10:1 to 0:1) and semi-preparative HPLC to afford compounds **11** (11 mg; CH_3_CN-H_2_O, 74:26, *t*_*R*_ 61.7 min) and **19** (10 mg; CH_3_CN-H_2_O, 74:26, *t*_*R*_ 55.9 min). Fr. 4 (18.7 g) was subjected to a silica gel CC with PE/EtOAc (10:1 to 0:1), ODS CC with H_2_O-MeOH (7:3 to 0:1), and semi-preparative HPLC to afford compounds **2** (24.5 mg; CH_3_CN-H_2_O, 83:17, *t*_*R*_ 31.7 min); **7** (3.8 mg; CH_3_CN-H_2_O, 80:20, *t*_*R*_ 32.4 min); and **22** (5.5mg; CH_3_CN-H_2_O, 83:17, *t*_*R*_ 23.8 min).

### Spectroscopic Data

Garcimultinone D (**1**): white amorphous powder; [*α*]^20^_D_ +186.27 (*c* 0.02, MeOH); UV (MeOH) *λ*_max_ (log *ε*) 215 (3.71) and 245 (3.89), 280 (3.81) nm; ECD (MeOH) *λ* (θ) 204 (−13.49), 229 (+0.59), 249 (−3.23), 274 (+8.77), 300 (+3.02), and 317 (+4.37) nm; ^1^H and ^13^C NMR (CDCl_3_), see Tables 1 and 2; HRESIMS *m/z* 571.3781 [M+H]^+^ (calcd for C_38_H_51_O_4_, 571.3782).

**TABLE 1:** ^13^C NMR data of compounds **1**–**14** in CDCl_3_ (150 MHz, *δ* in ppm).

No	1	2	3	4	5	6	7	8	9	10	11	12	13	14
1	79.3	78.9	78.1	78.4	71.1	71.5	72.4	69.3	69.5	68.4	82.1	196.3	195.8	161.2
2	193.0	194.7	188.9	188.9	167.9	167.0	166.5	173.2	173.1	194.0	204.1	52.7	56.5	104.6
3	124.7	124.7	118.5	119.8	112.3	114.1	113.9	119.1	118.5	125.5	66.6	171.9	174.1	160.3
4	167.3	169.0	176.6	179.4	192.6	195.7	195.6	192.5	192.2	171.7	208.2	116.6	117.4	100.5
5	51.2	51.4	53.4	54.3	63.2	62.4	64.0	61.9	62.0	51.4	69.1	189.0	189.2	154.5
6	40.7	40.9	42.1	39.9	43.3	43.5	44.6	43.4	42.6	39.6	37.8	107.6	107.2	105.2
7	42.8	43.0	48.9	48.4	48.8	49.1	42.9	48.8	48.9	46.5	43.3	29.1	29.7	22.1
8	48.0	48.2	49.7	50.1	50.8	49.3	47.8	49.7	48.9	46.4	48.7	41.0	44.2	40.9
9	208.4	208.5	206.2	207.3	209.7	208.2	207.8	208.4	206.9	207.4	204.3	85.6	87.7	78.6
10	193.9	192.1	193.9	193.2	192.9	194.2	192.5	192.5	193.6	193.8	193.3	196.6	196.4	200.6
11	137.0	129.1	137.0	136.6	136.7	137.4	130.5	136.8	137.6	137.7	135.7	139.0	139.1	143.3
12	128.4	115.5	128.6	128.3	128.7	128.6	115.5	128.6	128.2	129.0	128.7	127.5	127.6	127.4
13	128.0	143.5	128.1	128.2	128.3	127.8	142.6	128.8	128.1	128.5	128.3	127.9	127.8	127.6
14	132.1	148.3	132.2	132.4	132.6	132.1	148.3	133.4	132.4	133.2	132.6	130.9	130.8	130.1
15	128.0	114.3	128.1	128.2	128.3	127.8	114.1	128.8	128.1	128.5	128.3	127.9	127.8	127.6
16	128.4	122.1	128.6	128.3	128.7	128.6	123.3	128.6	128.2	129.0	128.7	127.5	127.6	127.4
17	22.0	22.0	28.2	80.2	114.6	16.5	16.3	26.8	27.8	25.7	31.1	30.2	30.0	21.9
18	121.4	121.4	93.4	99.5	124.5	31.4	31.9	93.6	93.7	120.0	88.5	121.8	121.8	122.3
19	132.0	132.3	70.8	71.1	83.8	80.3	80.3	71.7	70.8	134.9	73.7	133.8	133.1	136.2
20	18.1	18.2	25.2	24.0	28.6	25.7	25.9	23.9	25.1	18.2	25.6	18.0	18.1	18.1
21	26.0	25.9	27.0	26.0	30.6	27.5	27.2	26.7	26.4	26.4	27.2	26.0	26.0	26.0
22	29.1	29.1	34.3	29.8	29.4	35.5	35.0	36.2	35.2	28.5	33.3	38.6	47.7	29.3
23	40.0	40.0	44.7	120.5	46.9	44.0	44.0	47.1	44.0	43.0	43.5	117.6	42.8	122.2
24	84.2	84.9	148.3	135.3	74.2	148.9	148.6	148.9	148.7	86.6	149.3	136.1	147.9	133.3
25	28.4	28.4	18.0	18.4	24.1	18.2	18.3	21.3	18.1	21.4	18.7	18.3	19.5	18.0
26	21.4	21.6	112.1	26.1	29.8	112.2	112.3	109.8	112.5	28.7	112.4	26.0	112.5	26.1
27	30.2	30.1	33.6	—	32.3	33.1	32.8	135.7	33.1	29.8	34.0	—	34.6	26.7
28	121.5	121.4	122.8	—	124.9	123.3	123.5	132.5	123.1	125.1	122.5	—	122.5	20.1
29	134.4	134.5	132.5	—	133.1	131.7	132.8	81.5	131.9	133.3	132.6	—	132.9	—
30	18.2	18.2	18.2	—	18.2	18.2	18.2	24.1	18.2	18.3	18.2	—	18.2	—
31	26.1	26.1	26.0	—	26.0	26.0	25.9	30.1	26.0	26.2	26.0	—	26.0	—
32	26.9	26.8	30.6	30.7	31.5	29.4	27.8	29.3	29.5	29.5	28.8	21.5	21.8	—
33	122.8	122.7	125.1	125.6	125.7	125.1	122.7	124.7	124.9	121.6	41.5	122.1	122.2	—
34	133.4	133.5	132.5	132.9	131.3	132.9	133.5	133.2	133.1	133.9	88.6	131.9	132.1	—
35	18.1	18.1	18.2	18.1	18.1	18.1	18.1	18.1	18.0	18.3	29.5	18.1	18.0	—
36	26.0	26.0	26.0	26.2	26.2	26.0	26.0	26.0	26.0	26.0	19.4	26.2	26.0	—
37	23.2	23.3	22.7	23.0	23.7	23.6	24.5	23.9	24.3	22.7	25.3	28.5	29.8	—
38	16.3	16.2	27.0	27.1	27.3	27.5	16.3	26.9	26.8	27.0	22.7	21.4	21.8	—
17-OMe	—	—	—	59.1	—	—	—	—	—	—	—	—	—	—

**TABLE 2:** ^1^H NMR data of compounds **1–7** in CDCl_3_ (600 MHz, *δ* in ppm, *J* in Hz).

No	1	2	3	4	5	6	7
6	1.28 m	1.29 m	2.29 m	2.29 d (14.4)	2.15 m	2.11 m	1.44 m
	2.27 dd (13.2, 4.2)	2.27 dd (13.2, 4.2)	2.18 m	2.22 dd (13.8, 7.2)	2.25 m		1.95 dd (13.2, 4.2)
7	1.62 m	1.61 m	1.48 m	1.56 m	1.50 m	1.41 m	1.59 m
12	7.52 d (7.2)	6.92 d (4.2)	7.60 d (7.8)	7.54 d (7.8)	7.70 d (8.4)	7.67 d (7.2)	7.25 m
13	7.24 t (7.2)	—	7.27 t (7.8)	7.26 m	7.33 t (8.4)	7.27 t (7.2)	—
14	7.39 t (7.2)	—	7.41 t (7.8)	7.41 t (7.8)	7.47 t (8.4)	7.43 t (7.2)	—
15	7.24 t (7.2)	6.35 dd (8.4, 4.2)	7.27 t (7.8)	7.26 m	7.33 t (8.4)	7.27 t (7.2)	6.73 br s
16	7.52 d (7.2)	6.80 d (8.4)	7.60 d (7.8)	7.54 d (7.8)	7.70 d (8.4)	7.67 d (7.2)	7.19 d (8.4)
17	3.08 dd (13.2, 7.8)	3.13 dd (13.8, 7.2)	2.89 dd (14.4, 9.6)	4.79 d (3.0)	6.43 d (10.2)	2.29 m	2.33 m
	3.13 dd (13.2, 7.8)	3.17 dd (13.8, 7.2)	2.81 dd (15.0, 11.4)			2.43 m	2.61 m
18	5.09 br t (7.2)	5.07 m	4.78 dd (11.4, 9.6)	4.53 d (3.0)	5.28 d (10.2)	1.57 m	1.61 m
						1.38 m	1.51 m
20	1.67 s	1.71 s	1.28 s	1.30 s	0.62 s	0.53 s	0.71 s
21	1.64 s	1.69 s	1.45 s	1.35 s	1.41 s	1.23 s	1.22 s
22	1.68 m	1.72 m	1.67 m	2.55 m	2.06 m	1.78 dd (13.8, 4.8)	1.89 dd (13.8, 6.0)
	1.99 t (13.8)	2.02 t (13.8)	2.24 m	2.60 dd (15.6, 9.0)	2.29 m	2.20 m	2.10 m
23	1.85 m	1.85 m	2.46 m	5.03 d (7.8)	1.76 m	2.62 m	2.59 m
25	1.55 s	1.56 s	1.61 s	1.71 s	1.12 s	1.60 s	1.60 s
26	1.20 s	1.24 s	4.62 s	1.70 s	1.19 s	4.59 d (1.8)	4.63 s
						4.69 d (1.2)	4.70 s
27	1.80 m	1.83 m	2.11 m	—	2.13 m	2.04 m	2.01 m
	2.17 m	2.16 m	2.03 m				
28	5.03 br t (7.2)	5.09 m	5.02 br t (6.6)	—	4.87 br t (7.2)	5.02 br t (5.4)	5.04 br t (6.0)
30	1.63 s	1.62 s	1.62 s	—	1.54 s	1.61 s	1.60 s
31	1.74 s	1.74 s	1.70 s	—	1.68 s	1.68 s	1.69 s
32	2.21 m	2.19 m	2.26 m	2.36 m	2.08 m	2.23 m	2.07 m
	1.70 m	1.70 m	1.77 m			1.95 m	1.67 m
33	5.00 br t (6.0)	5.00 br t (6.0)	5.02 br t (6.6)	4.90 br t (7.2)	5.11 br t (7.2)	4.86 br t (6.6)	4.94 br t (6.6)
35	1.58 s	1.58 s	1.55 s	1.59 s	1.62 s	1.55 s	1.54 s
36	1.72 s	1.72 s	1.65 s	1.70 s	1.69 s	1.67 s	1.67 s
37	1.35 s	1.35 s	1.43 s	1.53 s	1.54 s	1.45 s	1.31 s
38	1.15 s	1.14 s	1.33 s	1.36 s	1.39 s	1.39 s	1.16 s
17-OMe	—	—	—	3.55 s	3.25 s	—	—
24-OH							

Garcimultinone E (**2**): white amorphous powder; [*α*]^20^_D_ +11.22 (*c* 0.15, MeOH); UV (MeOH) *λ*_max_ (log *ε*) 235 (3.18) and 310 (3.17) nm; ECD (MeOH) *λ* (θ) 248 (+0.33), 274 (−0.39), and 311 (+0.96) nm; ^1^H and ^13^C NMR (CDCl_3_), see Tables 1 and 2; HRESIMS *m/z* 603.3679 [M+H]^+^ (calcd for C_38_H_51_O_6_, 603.3641).

Garcimultinone F (**3**): white amorphous powder; [*α*]^20^_D_ +117.78 (*c* 0.02, MeOH); UV (MeOH) *λ*_max_ (log *ε*) 245 (4.04) and 290 (4.01) nm; ECD (MeOH) *λ* (θ) 225 (+4.85), 249 (−13.36), 284 (+12.82), 318 (−0.05), and 338 (+1.39) nm; ^1^H and ^13^C NMR (CDCl_3_), see Tables 1 and 2; HRESIMS *m/z* 587.37311 [M+H]^+^ (calcd for C_38_H_51_O_5_, 587.3731).

Garcimultinone G (**4**): colorless oil; [*α*]^20^_D_ +159.26 (*c* 0.02, MeOH); UV (MeOH) *λ*_max_ (log *ε*) 245 (4.02) and 280 (3.97) nm; ECD (MeOH) *λ* (θ) 222 (+4.07), 248 (−11.65), 273 (+14.02), 319 (+0.62), and 331 (+1.66) nm; ^1^H and ^13^C NMR (CDCl_3_), see Tables 1 and 2; HRESIMS *m/z* 549.3209 [M+H]^+^ (calcd for C_34_H_45_O_6_, 549.3211).

Garcimultinone H (**5**): colorless oil; [*α*]^20^_D_ +35.0 (*c* 0.02, MeOH); UV (MeOH) *λ*_max_ (log *ε*) 210 (3.51) nm; ECD (MeOH) *λ* (θ) 223 (+1.54), 256 (−1.26), 281 (−0.32), 306 (−0.62), and 349 (+0.57) nm; ^1^H and ^13^C NMR (CDCl_3_), see Tables 1 and 2; HRESIMS *m/z* 585.3587 [M-H]^−^ (calcd for C_38_H_49_O_5_, 585.3586).

Garcimultinone I (**6**): colorless oil; [*α*]^20^_D_ +73.90 (*c* 0.09, MeOH); UV (MeOH) *λ*_max_ (log *ε*) 235 (3.38) and 290 (3.35) nm; ECD (MeOH) *λ* (θ) 207 (−10.10), 218 (+9.57), 247 (−27.76), 266 (−1.51), 276 (−4.48), and 305 (+10.00) nm; ^1^H and ^13^C NMR (CDCl_3_), see Tables 1 and 2; HRESIMS *m/z* 571.3778 [M+H]^+^ (calcd for C_38_H_51_O_4_, 571.3782).

Garcimultinone J (**7**): colorless oil; [*α*]^20^_D_ −5.07 (*c* 0.05, MeOH); UV (MeOH) *λ*_max_ (log *ε*) 210 (3.44), 230 (3.39), and 275 (3.48) nm; ECD (MeOH) *λ* (θ) 225 (+0.51), 250 (+0.07), 267 (+0.41), 298 (−0.97), and 329 (+0.34) nm; ^1^H and ^13^C NMR (CDCl_3_), see Tables 1 and 2; HRESIMS *m/z* 603.3676 [M+H]^+^ (calcd for C_38_H_51_O_6_, 603.3641).

Garcimultinone K (**8**): pale yellow oil; [*α*]^20^_D_−12.00 (*c* 0.05, MeOH); UV (MeOH) *λ*_max_ (log *ε*) 220 (3.58), 245 (3.61), and 280 (3.50) nm; ECD (MeOH) *λ* (θ) 219 (+2.00), 250 (−15.45), 269 (−6.47), 284 (−11.22), and 321 (+5.44) nm; ^1^H and ^13^C NMR (CDCl_3_), see Tables 1 and 3; HRESIMS *m/z* 617.3488 [M-H]^−^ (calcd for C_38_H_49_O_7_, 617.3484).

**TABLE 3:** ^1^H NMR data of compounds **8–14** in CDCl_3_ (600 MHz, *δ* in ppm, *J* in Hz).

No	8	9	10	11	12	13	14
6	2.10 dd (13.8, 6.6)	2.12 m	2.29 d (14.4)	2.81 dd (14.4, 6.6)	—	—	—
	2.22 m		1.97 m	2.12 m			
7	1.47 m	1.44 m	1.45 m	2.06 m	2.18 dd (14.4, 3.6)	2.70 dd (14.4, 4.8)	2.04 dd (10.2, 6.6)
					1.26 m	1.26 m	2.65 dd (16.8, 5.4)
8	—	—	—	—	1.93 m	1.22 m	1.50 m
12	7.78 d (7.8)	7.68 d (7.2)	7.73 d (7.8)	7.18 d (7.2)	7.43 m	7.46 m	7.46 m
13	7.48 t (7.8)	7.32 t (7.2)	7.38 t (7.8)	7.29 t (7.2)	7.38 t (7.2)	7.38 t (7.2)	7.37 t (7.8)
14	7.57 t (7.8)	7.46 t (7.2)	7.50 t (7.8)	7.43 t (7.2)	7.46 m	7.46 m	7.44 m
15	7.48 t (7.8)	7.32 t (7.2)	7.38 t (7.8)	7.29 t (7.2)	7.38 t (7.2)	7.38 t (7.2)	7.37 t (7.8)
16	7.78 d (7.8)	7.68 d (7.2)	7.73 d (7.8)	7.18 d (7.2)	7.43 m	7.46 m	7.46 m
17	2.80 m	2.80 m	2.68 m	1.66 m	1.63 m	2.00 m	3.44 m
	3.05 m		2.43 dd (13.2, 4.2)	3.09 dd (14.4, 10.8)	2.06 m	1.71 m	
18	4.68 dd (10.8, 7.8)	4.03 t (10.8)	4.95 br t (7.2)	4.79 dd (11.4, 4.2)	5.02 m	4.98 t (7.2)	5.11 br t (7.2)
20	0.97 s	1.15 s	1.58 s	1.17 s	1.56 s	1.55 s	1.86 s
21	0.89 s	1.17 s	1.62 s	1.27 s	1.68 s	1.63 s	1.80 s
22	2.47 dd (13.8, 11.4)	1.75 dd (14.4, 4.8)	3.05 dd (14.4, 4.2)	2.25 dd (15.0, 10.2)	2.58 m	2.22 m	2.10 m
	1.90 m	2.16 m	0.95 m	1.88 m		1.72 m	1.68 m
23	3.04 m	2.59 m	1.39 m	2.55 m	5.04 m	2.19 m	5.32 br t (7.2)
25	1.73 s	1.57 s	1.23 s	1.67 s	1.60 s	1.61 s	1.57 s
26	4.78 s	4.61 s	0.83 s	4.70 s	1.70 s	4.72 s	1.70 s
	4.73 s	4.67 s		4.74 s		4.68 s	
27	5.62 dd (16.2, 10.2)	2.06 m	2.01 m	2.12 m	—	1.94 m	0.78 s
			1.78 m	2.20 m			
28	5.45 d (16.2)	5.01 br t (6.6)	4.91 br t (7.2)	5.07 br t (6.6)	—	4.94 t (7.2)	0.89 s
30	1.36 s	1.61 s	1.69 s	1.61 s	—	1.55 s	—
31	1.39 s	1.68 s	1.68 s	1.68 s	—	1.67 s	—
32	1.93 m	1.87 m	2.66 m	1.88 m	3.14 m	3.14 m	—
	2.22 m	2.22 m	2.18 m	2.03 m			
33	4.87 br t (6.0)	4.85 br t (6.6)	5.20 br t (7.2)	2.65 dd (11.4, 8.4)	5.09 br t (6.6)	5.12 t (7.2)	—
35	1.55 s	1.55 s	1.60 s	1.31 s	1.77 s	1.77 s	—
36	1.67 s	1.66 s	1.77 s	1.18 s	1.71 s	1.71 s	—
37	1.49 s	1.48 s	1.17 s	1.47 s	1.52 s	1.39 s	—
38	1.44 s	1.39 s	0.98 s	1.36 s	1.08 s	1.35 s	—
1-OH							12.65 s
3-OH							6.28 s

Garcimultinone L (**9**): white amorphous powder; [*α*]^20^_D_ +110.00 (*c* 0.04, MeOH); UV (MeOH) *λ*_max_ (log *ε*) 305 (3.24) nm; ECD (MeOH) *λ* (θ) 204 (−3.44), 218 (+6.00), 250 (−15.64), 272 (−1.72), 284 (−3.25), and 314 (+7.34) nm; ^1^H and ^13^C NMR (CDCl_3_), see Tables 1 and 3; HRESIMS *m/z* 587.3726 [M+H]^+^ (calcd for C_38_H_51_O_5_, 587.3731).

Garcimultinone M (**10**): white amorphous powder; [*α*]^20^_D_ −114.44 (*c* 0.02, MeOH); UV (MeOH) *λ*_max_ (log *ε*) 240 (4.03) nm; ECD (MeOH) *λ* (θ) 221 (+12.96), 272 (−11.16), 302 (+2.51), 322 (−0.79), and 351 (+2.08) nm; ^1^H and ^13^C NMR (CDCl_3_), see Tables 1 and 3; HRESIMS *m/z* 571.3780 [M+H]^+^ (calcd for C_38_H_51_O_4_, 571.3782).

Garcimultinone N (**11**): white amorphous powder; [*α*]^20^_D_ +12.59 (*c* 0.04, MeOH); UV (MeOH) *λ*_max_ (log *ε*) 210 (3.48) and 245 (3.44) nm; ECD (MeOH) *λ* (θ) 210 (−2.47), 248 (+13.52), 289 (−8.74), and 323 (+2.92) nm; ^1^H and ^13^C NMR (CDCl_3_), see Tables 1 and 3; HRESIMS *m/z* 619.3630 [M+H]^+^ (calcd for C_38_H_51_O_7_, 619.3629).

Garcimultinone O (**12**): white amorphous powder; [*α*]^20^_D_ +71.43 (*c* 0.03, MeOH); UV (MeOH) *λ*_max_ (log *ε*) 230 (3.73) and 355 (3.50) nm; ECD (MeOH) *λ* (θ) 215 (+2.95), 230 (−1.46), 254 (+5.15), 281 (−1.13), and 359 (+2.31) nm; ^1^H and ^13^C NMR (CDCl_3_), see Tables 1 and 3; HRESIMS *m/z* 503.3158 [M+H]^+^ (calcd for C_33_H_43_O_4_, 503.3156).

Garcimultinone P (**13**): white amorphous powder; [*α*]^20^_D_ −76.67 (*c* 0.04, MeOH); UV (MeOH) *λ*_max_ (log *ε*) 235 (3.75) and 350 (3.81) nm; ECD (MeOH) *λ* (θ) 227 (+5.26), 257 (−11.64), 295 (+3.38), and 364 (−3.93) nm; ^1^H and ^13^C NMR (CDCl_3_), see Tables 1 and 3; HRESIMS *m/z* 571.3780 [M+H]^+^ (calcd for C_38_H_51_O_4_, 571.3782).

Garcimultinone Q (**14**): yellow powder; [*α*]^20^_D_ +41.60 (*c* 0.04, MeOH); UV (MeOH) *λ*_max_ (log *ε*) 235 (3.63) and 315 (3.65) nm; ECD (MeOH) *λ* (θ) 220 (+1.21), 250 (−0.54), and 279 (+2.23) nm; ^1^H and ^13^C NMR (CDCl_3_), see Tables 1 and 3; HRESIMS *m/z* 435.2532 [M+H]^+^ (calcd for C_28_H_35_O_4_, 435.2530).

### NMR Calculations

The calculated NMR data were acquired using the Gauge-Including Atomic Orbitals (GIAO) method at the mPW1PW91/6–311+G (2 d,p) level in CHCl_3_ with the IEFPCM model (the detailed NMR calculations are described in the Supplementary information).

### ECD Calculations

The ECD calculation was conducted using time-dependent density functional theory (TD-DFT) in methanol by the IEFPCM model (the detailed ECD calculations are described in the Supplementary information).

### Antiproliferative Activity Bioassay

The antiproliferative activities against HepG2, T98, and MCF-7 cell lines of isolated compounds were measured by the CCK-8 method using cisplatin as the positive control, according to the protocol described previously (Teng et al., 2019).

## Results and Discussion

### PPAPs

Depending on the relative configuration at C-7 relative to C-1/C-5, BPAPs can also be subclassified into *endo*- and *exo*-subtypes. In case of *endo*-BPAPs, the chemical shifts of C-7 and Me-38 (axial position) appeared at 45–49 and 26–27 ppm, respectively. In case of *exo*-BPAPs, the chemical shifts of C-7 and Me-38 (axial position) displayed upfield signals at 41–44 and 16–17 ppm resulting from a γ-gauche effect between Me-38 and the CH_2_-32. Therefore, the analysis of ^13^C-NMR data is a powerful tool to distinguish the two types of BPAPs (Marti et al., 2010; Phang et al., 2020).

### Type A BPAPs

**FIGURE 2:**
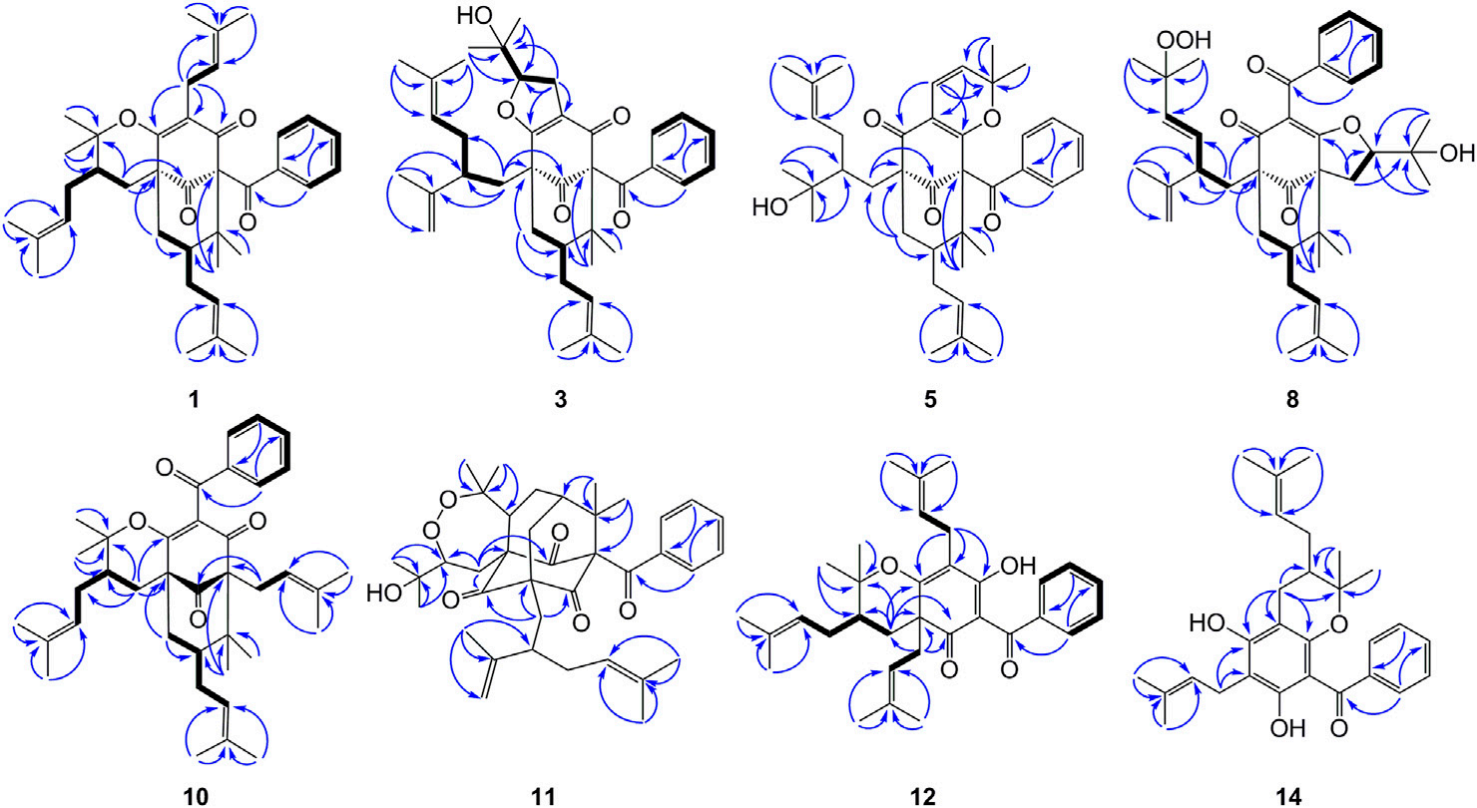
Key HMBC for compounds **1**, **3**, **5**, **8**, **10**–**12**, and **14**, and ^1^H–^1^H COSY correlations for **1**, **3**, **8**, **10**, **12**, and **14**.

**FIGURE 3:**
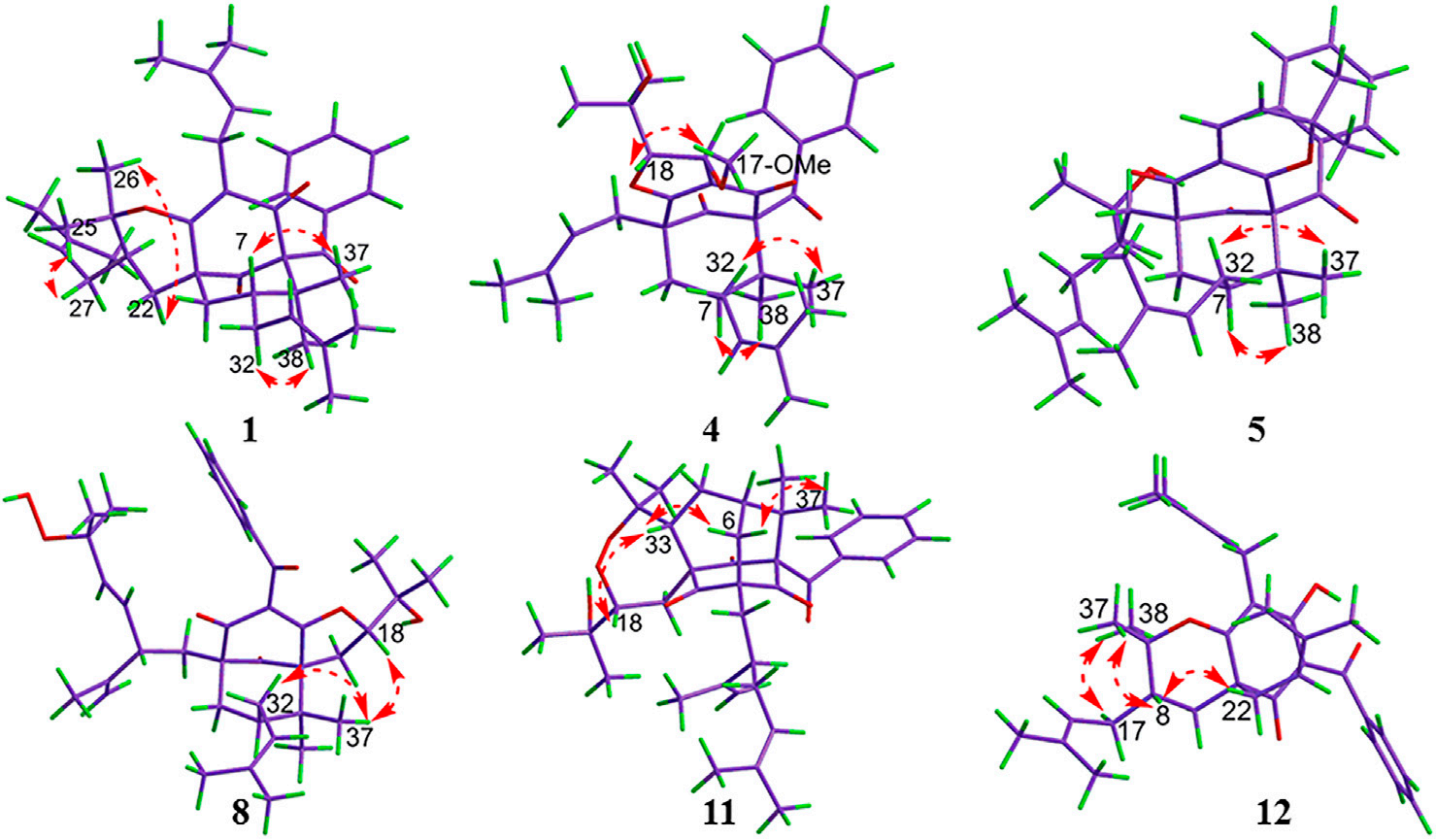
ROESY correlations for compounds **1**, **4**, **5**, **8**, **11**, and **12**.

**FIGURE 4:**
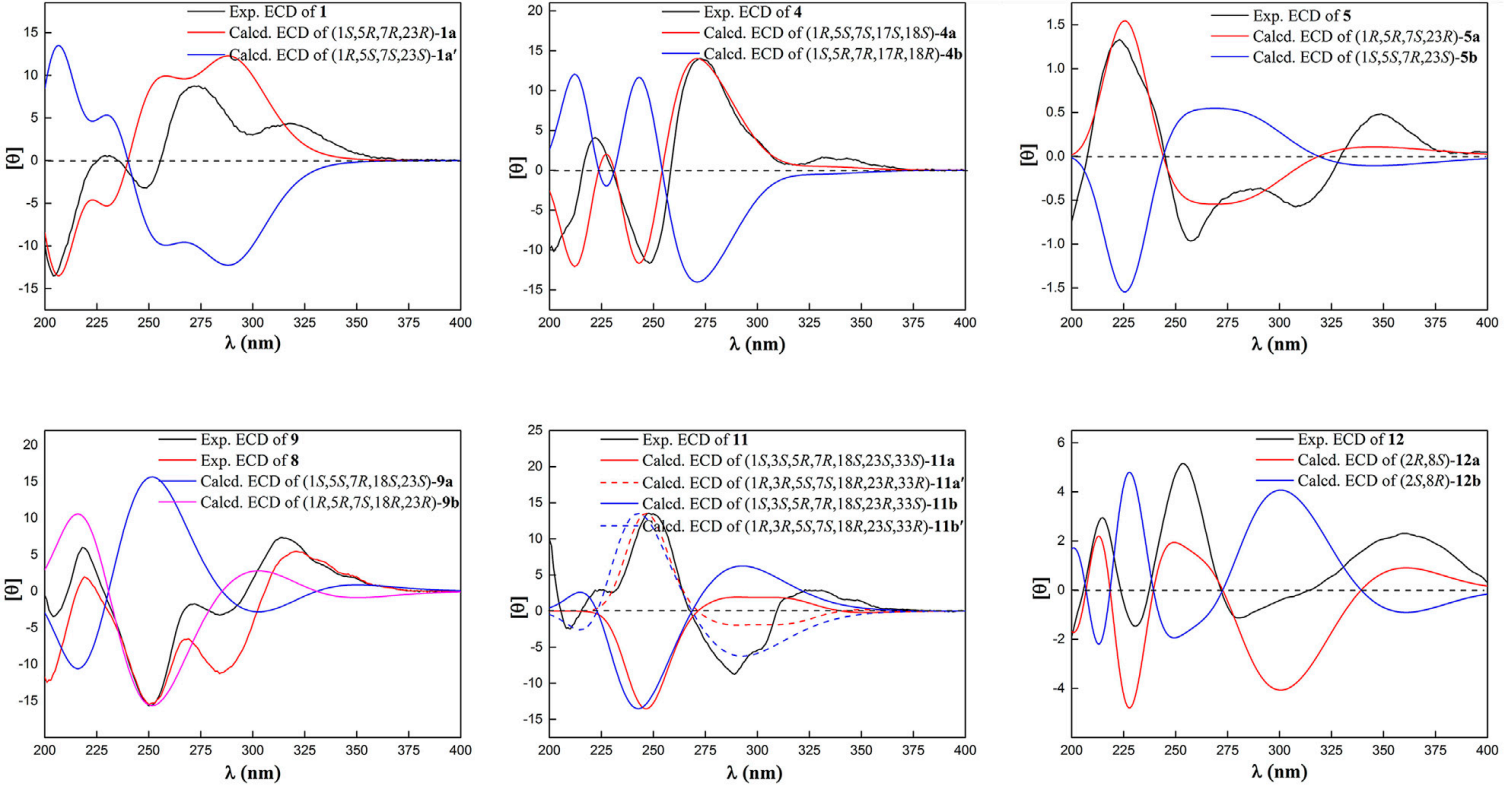
Calculated and experimental ECD spectra of **1**, **4**, **5**, **8**, **9**, **11**, and **12**.

Compound **1** was isolated as a white amorphous powder with the molecular formula of C_38_H_50_O_4_ implied by the HRESIMS at *m/z* 571.3781 [M + H]^+^ (calcd 571.3782) suggesting 14 degrees of unsaturation. Its ^1^H NMR spectrum (Table 2) contained signals of three olefinic protons (*δ*_H_ 5.09, 1H, br t, *J* = 7.2 Hz; 5.03, 1H, br t, *J* = 7.2 Hz; 5.00, 1H, br t, *J* = 6.0 Hz), 10 singlet methyls (*δ*_H_1.15–1.74), and one unsubstituted phenyl group (*δ*_H_ 7.52, 2H, d, *J* = 7.2 Hz; 7.24, 2H, t, *J* = 7.2 Hz; 7.39, 1H, t, *J* = 7.2 Hz). Detailed analysis of ^13^C-NMR, DEPT, and HSQC spectrum indicated the presence of characteristic peaks of the bicyclo[3.3.1]nonane skeleton, including a methylene at *δ*_C_ 40.7 (C-6), a methine at *δ*_C_ 42.8 (C-7), three *sp* ^*3*^ quaternary carbons at *δ*_C_ 79.3 (C-1), 51.2 (C-5), and 48.0 (C-8), a conjugated carbonyl carbon at *δ*_C_ 193.9 (C-10), a non-conjugated carbonyl carbon at *δ*_C_ 208.4 (C-9), and an enolized 1,3-diketo group at *δ*_C_ 193.0 (C-2), 124.7 (C-3), and 167.3 (C-4). The chemical shift of C-1 at *δ*_C_ 79.3 and HMBC correlations (Figure 2) from Me-37 and Me-38 to C-8 (*δ*_C_ 48.0) and C-1 suggested that compound **1** might belong to Type A BPAPs. By comparison of NMR data of **1** with those of garcimultiflorone A suggested that their structures were closely resembled (Chen et al., 2009). The major difference between **1** and garcimultiflorone A was that the resonances for C-7 (*δ*_C_ 42.8) and C-38 (*δ*_C_ 16.3) in **1** were shifted upfield compared to C-7 (*δ*_C_ 47.8) and C-38 (*δ*_C_ 26.8) in garcimultiflorone A, suggesting that **1** was the 7-epimer of garcimultiflorone A which was further supported by ROESY correlations of H_2_-32/H_3_-38 and H-7/H_3_-37 (Figure 3). To further determine the relative configuration of C-23, two possible isomers (1S*, 5R*, 7R*, and 23R*)-**1a** and (1S*, 5R*, 7R*, and 23S*)-**1b** were calculated by the DP4+ method. The results revealed that the experimental NMR data for **1** gave the best match of DP4+ probability 100% with the (1S*, 5R*, 7R*, and 23R*)-**1a** isomer. To ascertain the absolute configuration of **1** (1*S*, 5*R*, 7*R*, and 23*R*)-**1a**, and its enantiomer **1a′** were calculated by the time-dependent density functional theory (TDDFT) method. As a result, the experimental ECD spectrum of **1** matched well with the calculated ECD spectrum of (1*S*, 5*R*, 7*R*, and 23*R*)-**1a** (Figure 4). Thus, the absolute configuration of **1** was determined as (1*S*, 5*R*, 7*R*, and 23*R*). Finally, the structure of **1** was elucidated to be *exo*-BPAPs and named as garcimultinone D.

Compound **2** was isolated as a white amorphous powder. The molecular formula was established as C_38_H_50_O_6_ based on a *pseudo* molecular ion peak at *m/z* 603.3679 [M + H]^+^ (calcd 603.3641), indicating 32 mass units more than **1**. The NMR data (Tables 1 and 2) of **2** closely matched those of **1**, except for the presence of a 3,4-dihydroxybenzoyl group at C-1, replacing a benzoyl group at C-1 in **1**, which was supported by HMBC correlations H-12 and H-16 to C-10. The relative configuration of **2** was established as the same as that of **1** by ROESY spectrum (Supplementary Figure S138, Supplementary information) and ^13^C NMR data. The absolute configuration of **2** was finally assigned as (1*S*, 5*R*, 7*R*, and 23*R*) by comparing the experimental and calculated ECD spectra (Supplementary Figure S155, Supplementary information). Thus, the structure of compound **2** was elucidated, as shown in Figure 1, and named as garcimultinone E.

Compound **3** was obtained as a white amorphous powder and showed a *pseudo* molecular ion peak at *m/z* 587.3733 [M + H]^+^ (calcd 587.3731) in the HRESIMS, corresponding to the molecular formula C_38_H_50_O_5_. The ^1^H and ^13^C NMR data of **3** closely resembled those of hyperattenin C with the only difference being the presence of isogeranyl located at C-5 in **3**, instead of geranyl located at C-5 in hyperattenin C (Li et al., 2015). The assignment was further corroborated by the HMBC correlations from H_2_-22 to C-5 and C-9 (Figure 2). The relative configuration of **3** was determined by ROESY spectrum (Supplementary Figure S138, Supplementary information) and the ^13^C NMR data (Table 1) analysis. Compared to **1** and **2**, the chemical shift of C-7 (*δ*_C_ 48.9) and C-38 (*δ*_C_ 27.0) were shifted downfield. These findings suggested that **3** belonged to *endo*-BPAPs, which was confirmed by ROESY correlations of H_2_-32/H_3_-37 and H-7/H_3_-38. By comparison of NMR data of **3** with those of hyperattenin C and otogirinin D (Ishida et al., 2010) indicated that the chemical shifts of C-18, 19, 20, and 21 of **3** were consistent with those of hyperattenin C, suggesting the *β*-orientation of H-18. Many PPAPs with the isogeranyl group have been isolated from *G. multiflora* (Wang et al., 2018; Chen et al., 2019b; Teng et al., 2019). Owing to the characteristics of structural flexibility of the isogeranyl group, it is difficult to solve the absolute configuration of C-23 of the isogeranyl group by conventional structural elucidation methods. Thus, the relative configuration of compound **3** except C-23 was determined, named as garcimultinone F. The relative configuration of C-23 of **3** and its absolute configuration are discussed later together with compounds **5–7**.

Compound **4** was obtained as a colorless oil and showed a *pseudo* molecular ion peak at *m/z* 549.3209 [M + H]^+^ (calcd 549.3211) in the HRESIMS, corresponding to the molecular formula C_34_H_44_O_6_. The ^1^H NMR and ^13^C NMR spectrum of **4** and hyperattenin D were highly similar, except for the additional methoxy and prenyl groups and the absence of ethoxy and geranyl groups in **4** (Li et al., 2015). HMBC correlations (Supplementary Figure S137, Supplementary information) from MeO to C-17 and H_2_-22 to C-5 and C-9 suggested that methoxy and prenyl groups were located at C-17 and C-5, respectively. The relative configuration of **4** was established as the same as that of hyperattenin D by ROESY spectrum (Figure 3) and ^13^C NMR data (Table 1). The absolute configuration of **4** was defined to be 1*R*, 5*S*, 7*S*, 17*S*, and 18*S* by the ECD calculation (Figure 4). Thus, the structure of compound **4** was elucidated, as shown in Figure 1, and named as garcimultinone G.

Compound **5** was obtained as colorless oil. The molecular formula of **5** was deduced to be C_38_H_50_O_5_ based on its negative-ion HRESIMS data at *m/z* 585.3587 [M - H]^−^ (calcd for C_38_H_49_O_5_, 585.3586). Comparison of the NMR spectroscopic data (Tables 1, 2) of **5** with those of garcimultine A implied that they possessed a similar structure, except for the presence of an oxygenated tertiary carbon C-24 (*δ*_C_ 74.2) and a methyl [*δ*_H_ 1.19 (3H, s); *δ*_C_ 29.8] in **5**, replacing the terminal double bond Δ^24(26)^ in garcimultine A. These findings suggested that **5** was a Δ^24(26)^-hydrate of garcimultine A (Liu et al., 2017b), which was ascertained by HMBC cross-peaks (Figure 2) from H_3_-25/H_3_-26 to C-23 (*δ*_C_ 46.9) and C-24 (*δ*_C_ 74.2). Compound **5** was defined as *endo*-BPAPs based on the analyses of ROESY spectrum (Figure 3) and ^13^C-NMR data (Table 1). Therefore, the relative configuration of **5** except C-23 was defined and named as garcimultinone H.

Compound **6** isolated as a colorless oil and gave the molecular formula C_38_H_50_O_4_ as revealed by its HRESIMS at *m/z* 571.3778 [M + H]^+^ (calcd for C_38_H_51_O_4_, 571.3782). Comparison of the NMR data of **6** and hypersampsone T indicated that their structures were highly similar, except for the C-5 substituent. Obviously, isogeranyl NMR signals of **6** replaced those for a prenyl group in hypersampsone T (Tian et al., 2016). This deduction was further confirmed by HMBC correlations from H_2_-22 to C-4, C-5, C-9, C-23, and C-24 (Supplementary Figure S138, Supplementary information). Thus, the relative configuration of **6** except C-23 was defined and named as garcimultinone I.

Compound **7** were isolated as a colorless oil and gave the molecular formula C_38_H_50_O_6_, as revealed by its HRESIMS at *m/z* 603.3676 [M + H]^+^ (calcd C_38_H_51_O_6_, 603.3641), indicating 32 mass units more than **6**. Comparison of NMR data (Tables 1, 2) of **6** and **7** showed many similarities with two major differences. Firstly, the presence of a 3,4-dihydroxybenzoyl group at C-1 in **7** instead of a benzoyl group at C-1 in **6** was observed in NMR data, which was further confirmed by HMBC spectrum (Supplementary Figure S138, Supplementary information). Secondly, the chemical shifts of C-7 and CH_3_-38 were both shifted upfield 6.2 and 11.2 ppm, respectively, compared to **6**, suggesting to be *exo*-BPAPs which was further supported by ROESY spectrum. Thus, the relative configuration of **7** except C-23 was established, and named as garcimultinone J.

Compounds **3** and **5–7** all contain the isogeranyl group featuring a stereocenter at C-23, which is a challenge for the determination of the absolute configuration. Recently, the absolute configuration of C-23 of the isogeranyl of PPAPs bearing exocyclic stereocenters such as guttiferone F has been determined and revised by total synthesis and X-ray diffraction, and a preliminary conclusion has been drawn that BPAP-bearing exocyclic stereocenters from natural sources mostly carries *S* configuration of the isogeranyl. According to this rule, all (23*R*)-*endo*-type B BPAPs with a isogeranyl at C-5 are corrected to be (23*S*)-*endo*-type B BPAPs, including garcimultiflorones D–F, 18-hydroxygarcimultiflorone D, isogarcimultiflorone F, and garcimultiflorone J isolated from the same plant (Wang et al., 2021; Zheng et al., 2021). Combined with the biosynthetic pathway and this rule, the configuration of C-23 could be tentatively determined as S*. Therefore, the relative configuration of **3** and **5–7** was defined as (1R*, 5R*, 7R*, 18R*, and 23S*), (1S*, 5S*, 7R*, and 23S*), (1S*, 5R*, 7R*, and 23S*), and (1S*, 5R*, 7S*, and 23S*), respectively. Subsequently, by comparing the calculated ECD spectrum of compounds **3** and **5–7** with the experimental ECD spectrum, the absolute configurations of compounds **3** and **5–7** were determined as to be (1*S*, 5*S*, 7*S*, 18*S*, and 23*R*), (1*R*, 5*R*, 7*S*, and 23*R*), (1*R*, 5*S*, 7*S*, and 23*R*), and (1*R*, 5*S*, 7*R*, and 23*R*), respectively.

### Type B BPAPs

Compound **8** was obtained as pale yellow oil. The HRESIMS data at *m/z* 617.3488 [M - H]^−^ of **8** together with ^13^C-NMR and DEPT indicated molecular formula of C_38_H_50_O_7_. The ^1^H, ^13^C NMR, HSQC, and HMBC spectrum of **8** disclosed characteristic signals of the Type B BPAPs skeleton, including a methylene at *δ*_C_ 43.4 (C-6), a methine at *δ*_C_ 48.8 (C-7), three *sp* ^*3*^ quaternary carbon at *δ*_C_ 69.3 (C-1), 61.9 (C-5), and 49.7 (C-8), a conjugated carbonyl carbon at *δ*_C_ 192.5 (C-10), a non-conjugated carbonyl carbon at *δ*_C_ 208.4 (C-9), an enolized 1,3-diketo group at *δ*_C_ 173.2 (C-2), 119.1 (C-3), and 192.5 (C-4), and HMBC correlations from Me-37 and Me-38 to C-8 (*δ*_C_ 49.7) and C-1 (*δ*_C_ 69.3). The NMR data (Tables 1, 3) of **8** was highly similar to those of hyperibone I except for the replacement of a prenyl group at C-5 in hyperibone I by a *E*-5-methyl-2-(1-methylethenyl)-5-hydroperoxy-hex-3-enyl group in **8** (Matsuhisa et al., 2002). The key HMBC correlations from Me-30 and Me-31 to C-28 and C-29, from Me-25 to C-23, C-24, and C-26, and from H_2_-22 to C-4, C-5, C-23, C-24, and C-27 (Figure 2), together with ^1^H-^1^H COSY of H_2_-22/H-23/H-27/H-28 confirmed this conclusion. The double bond of Δ^27(28)^ was assigned the *E* configuration based on their coupling constant values 16.2 Hz (Zhang et al., 2016). By comparison of ^13^C-NMR data of **8** and hyperibone I suggested that the relative configurations C-1, C-5, and C-18 of **8** was consistent with hyperibone I, which was further confirmed by ROESY correlations H-18/H_3_-37 and H_3_-37/H_2_-32 (Figure 3). The determination of its absolute configuration will be discussed with compound **9**.

Compound **9** was obtained as a white amorphous powder. The HRESIMS data at *m/z* 587.37262 [M + H]^+^ of **9** together with ^13^C-NMR and DEPT indicated molecular formula of C_38_H_50_O_5_. The NMR data of **9** was highly similar to those of **8** (Tables 1, 3), except for the presence of a prenyl group attached to C-23 in **9**, instead of *E*-3-methyl-3-hydroperoxy-but-1-enyl group in **8**. The key HMBC correlations from Me-30 and Me-31 to C-28 (*δ*_C_ 123.1) and C-29 (*δ*_C_ 131.9) confirmed this conclusion. Compound **9** was also determined as *endo*-type B BPAPs based on the chemical shift of C-7 (*δ*_C_ 48.9) and C-38 (*δ*_C_ 26.8) and ROESY correlation between Me-37 and H_2_-32. The NMR difference between compounds **8** and **9** mainly lied in the chemical shift and splitting of H-18 [(*δ*_H_ 4.03, t, *J* = 10.8 Hz) in **9** (*δ*_H_ 4.68, dd, *J* = 10.8, 7.8 Hz) in **8**], suggesting the orientation of H-18 in **9** was opposite to **8**, which was further confirmed by ROESY correlation of Me-37/Me-21. As with the above discussion of compounds **3** and **5–7**, the relative configurations of compounds **8** and **9** could be determined as (1S*, 5R*, 7R*, 18R*, and 23R*) and (1S*, 5S*, 7R*, 18S*, and 23S*), respectively. Owing to the change of the priority order of functional groups at C-23, the relative configuration of C-23 of **8** changed from S* to R*. The calculated ECD spectrum of (1*R*, 5*R*, 7*S*, 18*R*, and 23*R*)-**9b** was in good agreement with the experimental ECD spectrum of **9**, establishing the absolute configuration of **9** as 1*R*, 5*R*, 7*S*, 18*R*, and 23*R* (Figure 4). Owing to the similarity of experimental ECD curves between **8** and **9**, the absolute configuration of **8** was defined as 1*R*, 5*S*, 7*S*, 18*S*, and 23*S*. Thus, the structures of **8** and **9** were established as depicted in Figure 1, and named as garcimultinones K and L, respectively.

Compound **10** was obtained as a white amorphous powder. The HRESIMS data at *m/z* 571.37780 [M + H]^+^ of **10**, together with ^13^C-NMR and DEPT indicated molecular formula of C_38_H_50_O_4_. Its NMR data were highly similar to those of isogarcinol (Marti et al., 2010; Gustafson et al., 1992), except for the presence of a benzoyl group in **10**, instead of 3,4-dihydroxybenzoyl group in isogarcinol. Thus, **10** was a 13,14-didehydoxy of isogarcinol, which was further confirmed by the HMBC correlations (Figure 2). In a previous report, 13,14-didehydoxyisogarcinol was isolated from the fruit of *G. multiflora* (Chen et al., 2009), which has been corrected to 13,14-didehydoxy-7-epi-isogarcinol (Yang et al., 2018). The relative configuration of **10** was deduced as the same as that of isogarcinol from the ROESY spectrum (Supplementary Figure S139, Supplementary information). In the experimental ECD spectrum, compound **10** showed positive Cotton effect (CE) at 220 nm and negative CE at 270 nm, establishing the absolute configuration of **10** as the same as that of isogarcinol (Socolsky and Plietker, 2015). This deduction was further confirmed by ECD calculations (Supplementary Figure S159, Supplementary information). Thus, the structures of **10** were established as 13,14-didehydoxyisogarcinol and named as garcimultinone M. Compared to **8** and **9**, compound **10** display the different side chain orientations of the bicyclo[3.3.1]nonane moiety. However, it is noteworthy that the corresponding compounds show the same CD spectrum. Compounds **8–9** are the presence of an enolized C-2 *via* ether ring closure and C-4 keto form, while compound **10** is the presence of C-2 keto form and an enolized C-4 *via* ether ring closure. These findings could imply that the position of an enolized 1,3-diketo group in the core structure might affect the molecular conformation and hence the ECD curves (Le et al., 2016; Sukandar et al., 2020).

### Caged PPAPs

The HRESIMS data of **11** displayed an [M + H]^+^ ion at *m/z* 619.3630 (calcd for 619.3629), corresponding to the molecular formula of C_38_H_50_O_7_. The ^1^H, ^13^C NMR, HSQC, and HMBC spectrum of **11** contained characteristic signals of a homo-adamantane PPAPs skeleton, including three non-conjugated carbonyls at *δ*_C_ 208.2 (C-4), 204.1 (C-2), and 204.3 (C-9), four quaternary carbons at *δ*_C_ 82.1 (C-1), 66.6 (C-3), 69.1 (C-5), and 48.7 (C-8), two methines at *δ*_C_ 43.3 (C-7) and 41.5 (C-33), two methylenes at *δ*_C_ 37.8 (C-6) and 28.8 (C-32), and HMBC correlations (Figure 2) from Me-37 and Me-38 to C-8 (*δ*_C_ 48.7), C-7 (*δ*_C_ 43.3), and C-1 (*δ*_C_ 82.1). Comparison of the NMR data of **11** with those of garcimultiflorone G disclosed that the planar structure of **11** was identical to that of garcimultiflorone G (Ting et al., 2014). Compared to garcimultiflorone G, the chemical shifts of C-6 and C-32 was shielded from *δ*_C_ 45.1 (C-6) and 31.6 (C-32) in garcimultiflorone G to *δ*_C_ 37.8 (C-6) and 28.8 (C-32) in **11**. Therefore, H-33 was determined as *β*-oriented. The Δ*δ*_C_ between Me-35 and Me-36 was about 10 ppm, suggesting the relative configuration of H-18 and H-33 taken as *cis*-oriented (Ye et al., 2019). This deduction was further confirmed by ROESY spectrum (Figure 3). Therefore, the relative configuration of **11** except C-23 can be determined as (1S*, 3S*, 5R*, 7R*, 18S*, and 33S*). The carbon skeleton of **11** was different from the abovementioned compounds, and this rule might be not suitable for determining the configuration of C-23. Thus, The ECD calculations for (1*S*, 3*S*, 5*R*, 7*R*, 18*S*, 23*S*, and 33*S*)-**11a**, (1*S*, 3*S*, 5*R*, 7*R*, 18*S*, 23*R*, and 33*S*)-**11b**, and their enantiomers **11a′** and **11b′** were performed using the TDDFT/ECD method at the B3LYP/6–31+G(d) level. As a result, the calculated ECD curves of **11a′** and **11b′** matched well the experimental ECD spectra of **11** (Figure 4). Consequently, the absolute configuration of **11** except C-23 was established, as shown in Figure 1, and named as garcimultinone N.

### MPAPs

Compound **12** was obtained as a white amorphous powder. The molecular formula of **12** was established as C_33_H_42_O_4_ by the ^13^C NMR, DEPT, and HRESIMS data at *m/z* [M + H]^+^ 503.3158 (calcd for C_33_H_43_O_4_, 503.3156). The ^1^H- and ^13^C-NMR spectra (Tables 1, 3) revealed the existence the characteristic signals of the phloroglucinol core, including one *sp* ^*3*^ quaternary carbon at *δ*_C_ 52.7 (C-2), an enolized 1,3-diketo group at *δ*_C_ 196.3 (C-1), 107.6 (C-6), and 189.0 (C-5), and an enolic moiety at *δ*_C_ 171.9 (C-3) and 116.6 (C-4). Comparison of the NMR data **12** with those of hypelodin A indicated that the *E*-4-methylpent-1,3-dienyl group attached to C-9 in hypelodin A was replaced by the methyl group at C-9 in **12** (Hashida et al., 2014), which was further confirmed by HMBC correlations from Me-37 and Me-38 to C-8 (*δ*_C_ 41.0) and C-9 (*δ*_C_ 85.6). In the ROESY spectrum of **12** (Figure 3), the correlations of H-8/H_2_-22, H-8/H_3_-37, and H_3_-38/H_2_-17 demonstrated that H-8, 2-prenyl, and CH_3_-37 were cofacial and arbitrarily assigned as *β*-oriented. The calculated ECD spectrum of (2*R* and 8*S*)-**12a** matched well with the experimental ECD spectrum of **12** (Figure 4). Thus, the absolute configuration of **12** was established as (2*R*, 8*S*), and compound **12** was named as garcimultinone O.

Compound **13** was obtained as a white amorphous powder. The molecular formula of **13** was established as C_38_H_50_O_4_ by the ^13^C NMR, DEPT, and HRESIMS data at *m/z* [M + H]^+^ 571.3780 (calcd for C_38_H_51_O_4_, 571.3782). Comparison of the NMR data **13** with those of **12** indicated the presence of an isogeranyl group at C-2 in **13** as opposed to a prenyl group at C-2 in **12**. However, the chemical shifts of C-2 and C-8 were shifted downfield from C-2 (*δ*_C_ 52.7) and C-8 (*δ*_C_ 41.0) in **12** to C-2 (*δ*_C_ 56.5) and C-8 (*δ*_C_ 44.2) in **13**, suggesting that 8-prenyl and 2-isogeranyl groups were taken as *cis* relationship (Xu et al., 2019). ROESY correlations H-17/H_2_-22/Me-38 supported this deduction (Supplementary Figure S139, Supplementary information). The relative configuration of C-23 remained undetermined. Thus, the ECD calculations for (2*R*, 8*R*, and 23*R*)-**13a**, (2*R*, 8*R*, and 23*S*)-**13b**, and their enantiomers **13a′** and **13b′** were carried out. These result showed that the calculated ECD curves of **13a′** and **13b′** were in good agreement with experimental ECD data (Supplementary Figure S160, Supplementary information). Thus, the absolute configuration of **13** except C-23 was established as (2*S* and 8*S*), and compound **13** was named as garcimultinone P.

Compound **14** was obtained as a yellow powder, which possessed a molecular formula of C_28_H_34_O_4_ as determined by the HRESIMS data at *m/z* 435.2532 [M + H]^+^ (calcd for C_28_H_35_O_4_, 435.2530) in accordance with its ^13^C NMR data. The ^1^H- and ^13^C-NMR spectra (Tables 1 and 3) showed the characteristic signals for the phloroglucinol core, including three *sp* ^*2*^ quaternary carbons at *δ*_C_ 104.6 (C-2), *δ*_C_ 100.5 (C-4), and *δ*_C_ 105.2 (C-6), three *sp* ^*2*^ oxygenated quaternary carbons at *δ*_C_ 161.2 (C-1), *δ*_C_ 160.3 (C-3), and *δ*_C_ 154.5 (C-5), which constructed six substituted benzene ring. The ^1^H- and ^13^C-NMR data of **14** were very similar to those of vismiaguianones A (Seo et al., 2000), except for the presence of an additional prenyl group in **14** and the chemical shift of C-8 being shifted upfield from C-8 (*δ*_C_ 68.7) in vismiaguianones A to C-8 (*δ*_C_ 40.9) in **14**. Thus, the hydroxy group in vismiaguianones A was replaced by the prenyl group in **14**. This deduction was subsequently confirmed by the correlations of H_2_-22 (*δ*_H_ 2.10, 1.68) with C-7 (*δ*_C_ 22.1) and C-8 (*δ*_C_ 40.9) in the HMBC spectrum (Figure 2). The absolute configuration of **14** was established as 8*S* by comparing the calculated and experimental ECD data (Supplementary Figure S161, Supplementary information), and compound **14** was named as garcimultinone Q.

The ten known analogues were identified as garcimultiflorone A (**15**) (Chen et al., 2009), hyperscabrone M (**16**) (Gao et al., 2016), 13,14-didehydoxy-7-epi-isogarcinol (**17**) (Chen et al., 2009), xerophenone C (**18**) (Thoison et al., 2005), garcimultiflorone G (**19**) (Ting et al., 2014), garcimultiflorone P (**20**) and garcimultiflorone N (**21**) (Wang et al., 2018), garciniagifolone A (**22**) (Shan et al., 2012), garcibracteatone (**23**) (Thoison et al., 2005), and nemorosonol (**24**) (Oya et al., 2015).

All isolated compounds were evaluated for their inhibitory effects against the human T98, HepG2, and MCF-7 cancer cell lines by the CCK-8 method. Compounds **2** and **7** displayed evident antiproliferative activity against three tested cell lines (Supplementary Table S1, Supplementary information). The IC_50_ values of antiproliferative activities of compound **2** on T98, HepG2, and MCF-7 cancer cell lines were 13.23 ± 4.24, 13.53 ± 0.17, and 9.81 ± 1.56 μM, respectively. Compound **7** showed antiproliferative activity against T98, HepG2, and MCF-7 cancer cell lines with IC_50_ values of 17.00 ± 2.75, 12.84 ± 1.59, and 15.68 ± 1.65 μM, respectively. However, the other compounds were inactive showing IC_50_ values in excess of 20 μM. According to the structure type and biological activity of the isolated compounds, it could be preliminarily inferred that 3,4-dihydroxybenzoyl substituents in the structures of BPAPs are important for their anticancer activities (Yang et al., 2018).

## Conclusion

In summary, the phytochemical investigation of the fruits of *G. multiflora* resulted in the isolation and structure elucidation of 24 structurally diverse polyprenylated acylphloroglucinols (PAPs) including 11 new PPAPs (**1**–**11**) and **3** new MPAPs (**12**–**14**). These PPAPs belong to three types including the bicyclic polyprenylated acylphloroglucinols (BPAPs, **1**–**10** and **15**–**18**), the caged PPAPs (**11** and **19**–**22**), and the complicated PPAPs (**23** and **24**). Interestingly, most of PAPs are linked with the isogeranyl or its derivatives. The BPAPs with 3,4-dihydroxybenzoyl were found to exhibit effectively antiproliferative activity. These findings indicated that the fruits of *G. multiflora* are an important source of structural diversity PAPs, which deserve further study.

## Data Availability

The original contributions presented in the study are included in the article/Supplementary Material; further inquiries can be directed to the corresponding authors.
