# Supplementary material for: Polyprenylated Acylphloroglucinols With Different Carbon Skeletons From the Fruits of Garcinia multiflora
Source: Front Chem. 2021 Oct 26;9:756452. doi: 10.3389/fchem.2021.756452 (PMC8576638; doi:10.3389/fchem.2021.756452)
Supplement: Supplementary file 1 [file DataSheet1.PDF]

Supplementary Information

Polyprenylated Acylphloroglucinols With Different Carbon Skeletons From the Fruits of *Garcinia multiflora*

Haida Teng[a,1], *Qingqing Li*[b,1], *Ziyu Ma*[a], *Xueni Li*[b], *Wenli Xie*[b], *Yu Chen*[a*], *Guangzhong Yang*[b*]

[a] *College of Chemistry and Material Sciences, South-Central University for Nationalities, Wuhan 430074, P. R. China*

[b] *School of Pharmaceutical Sciences, South-Central University for Nationalities, Wuhan 430074, P. R. China*

*Corresponding authors. Tel./fax: +86 27 6784 1196
E-mail address: chenyuwh888@126.com (Y. Chen); yanggz888@126.com (G. Yang)

[1] These authors contributed equally to this work.

Contents

Figure S1: ^{1}H-NMR (600 MHz, $CDCl_3$) spectrum of compound 1 ....S7

Figure S2: ^{13}C-NMR (150 MHz, $CDCl_3$) spectrum of compound 1 ....S7

Figure S3: ^{13}C-NMR-DEPT (θ=135°) spectrum of compound 1 ....S8

Figure S4: HSQC spectrum of compound 1 ....S8

Figure S5: HMBC spectrum of compound 1 ....S9

Figure S6: ^{1}H-^{1}H COSY spectrum of compound 1 ....S9

Figure S7: ROESY spectrum of compound 1 ....S10

Figure S8: UV spectrum of compound 1 ....S10

Figure S9: HR-ESI-MS of compound 1 ....S11

Figure S10: CD spectrum of compound 1 ....S11

Figure S11: ^{1}H-NMR (600 MHz, $CDCl_3$) spectrum of compound 2 ....S12

Figure S12: ^{13}C-NMR (150 MHz, $CDCl_3$) spectrum of compound 2 ....S12

Figure S13: ^{13}C-NMR-DEPT (θ=135°) spectrum of compound 2 ....S13

Figure S14: HSQC spectrum of compound 2 ....S13

Figure S15: HMBC spectrum of compound 2 ....S14

Figure S16: ^{1}H-^{1}H COSY spectrum of compound 2 ....S14

Figure S17: ROESY spectrum of compound 2 ....S15

Figure S18: UV spectrum of compound 2 ....S15

Figure S19: HR-ESI-MS of compound 2 ....S16

Figure S20: CD spectrum of compound 2 ....S16

Figure S21: ^{1}H-NMR (600 MHz, $CDCl_3$) spectrum of compound 3 ....S17

Figure S22: The ^{13}C-NMR (150 MHz, $CDCl_3$) spectrum of compound 3 ....S17

Figure S23: ^{13}C-NMR-DEPT (θ=135°) spectrum of compound 3 ....S18

Figure S24: HSQC spectrum of compound 3 ....S18

Figure S25: HMBC spectrum of compound 3 ....S19

Figure S26: ^{1}H-^{1}H COSY spectrum of compound 3 ....S19

Figure S27: ROESY spectrum of compound 3 .... **S20**

Figure S28: UV spectrum of compound 3 .... **S20**

Figure S29: HR-ESI-MS of compound 3 .... **S21**

Figure S30: CD spectrum of compound 3 .... **S21**

Figure S31: ^{1}H-NMR (600 MHz, $CDCl_3$) spectrum of compound 4 .... **S22**

Figure S32: The ^{13}C-NMR (150 MHz, $CDCl_3$) spectrum of compound 4 .... **S22**

Figure S33: ^{13}C-NMR-DEPT (θ=135°) spectrum of compound 4 .... **S23**

Figure S34: HSQC spectrum of compound 4 .... **S23**

Figure S35: HMBC spectrum of compound 4 .... **S24**

Figure S36: ROESY spectrum of compound 4 .... **S24**

Figure S37: UV spectrum of compound 4 .... **S25**

Figure S38: HR-ESI-MS of compound 4 .... **S25**

Figure S39: CD spectrum of compound 4 .... **S26**

Figure S40: ^{1}H-NMR (600 MHz, $CDCl_3$) spectrum of compound 5 .... **S26**

Figure S41: ^{13}C-NMR (150 MHz, $CDCl_3$) spectrum of compound 5 .... **S27**

Figure S42: ^{13}C-NMR-DEPT (θ=135°) spectrum of compound 5 .... **S27**

Figure S43: HSQC spectrum of compound 5 .... **S28**

Figure S44: HMBC spectrum of compound 5 .... **S28**

Figure S45: ROESY spectrum of compound 5 .... **S29**

Figure S46: UV spectrum of compound 5 .... **S29**

Figure S47: HR-ESI-MS of compound 5 .... **S30**

Figure S48: CD spectrum of compound 5 .... **S30**

Figure S49: ^{1}H-NMR (600 MHz, $CDCl_3$) spectrum of compound 6 .... **S31**

Figure S50: ^{13}C-NMR (150 MHz, $CDCl_3$) spectrum of compound 6 .... **S31**

Figure S51: ^{13}C-NMR-DEPT (θ=135°) spectrum of compound 6 .... **S32**

Figure S52: HSQC spectrum of compound 6 .... **S32**

Figure S53: HMBC spectrum of compound 6 .... **S33**

Figure S54: ROESY spectrum of compound 6 .... **S33**

Figure S55: UV spectrum of compound 6 .... **S34**

Figure S56: HR-ESI-MS of compound 6 .... S34

Figure S57: CD spectrum of compound 6 .... S35

Figure S58: ^{1}H-NMR (600 MHz, $CDCl_3$) spectrum of compound 7 .... S35

Figure S59: The ^{13}C-NMR (150 MHz, $CDCl_3$) spectrum of compound 7 .... S36

Figure S60: ^{13}C-NMR-DEPT (θ=135°) spectrum of compound 7 .... S36

Figure S61: HSQC spectrum of compound 7 .... S37

Figure S62: HMBC spectrum of compound 7 .... S37

Figure S63: ROESY spectrum of compound 7 .... S38

Figure S64: UV spectrum of compound 7 .... S38

Figure S65: HR-ESI-MS of compound 7 .... S39

Figure S66: CD spectrum of compound 7 .... S39

Figure S67: ^{1}H-NMR (600 MHz, $CDCl_3$) spectrum of compound 8 .... S40

Figure S68: ^{13}C-NMR (150 MHz, $CDCl_3$) spectrum of compound 8 .... S40

Figure S69: ^{13}C-NMR-DEPT (θ=135°) spectrum of compound 8 .... S41

Figure S70: HSQC spectrum of compound 8 .... S41

Figure S71: HMBC spectrum of compound 8 .... S42

Figure S72: ^{1}H-^{1}H COSY spectrum of compound 8 .... S42

Figure S73: ROESY spectrum of compound 8 .... S43

Figure S74: UV spectrum of compound 8 .... S43

Figure S75: HR-ESI-MS of compound 8 .... S44

Figure S76: CD spectrum of compound 8 .... S44

Figure S77: ^{1}H-NMR (600 MHz, $CDCl_3$) spectrum of compound 9 .... S45

Figure S78: ^{13}C-NMR (150 MHz, $CDCl_3$) spectrum of compound 9 .... S45

Figure S79: ^{13}C-NMR-DEPT (θ=135°) spectrum of compound 9 .... S46

Figure S80: HSQC spectrum of compound 9 .... S46

Figure S81: HMBC spectrum of compound 9 .... S47

Figure S82: ^{1}H-^{1}H COSY spectrum of compound 9 .... S47

Figure S83: ROESY spectrum of compound 9 .... S48

Figure S84: UV spectrum of compound 9 .... S48

Figure S85: HR-ESI-MS of compound 9 .... S49

Figure S86: CD spectrum of compound 9 .... S49

Figure S87: ^{1}H-NMR (600 MHz, $CDCl_3$) spectrum of compound 10 .... S50

Figure S88: ^{13}C-NMR (150 MHz, $CDCl_3$) spectrum of compound 10 .... S50

Figure S89: ^{13}C-NMR-DEPT (θ=135°) spectrum of compound 10 .... S51

Figure S90: HSQC spectrum of compound 10 .... S51

Figure S91: HMBC spectrum of compound 10 .... S52

Figure S92: ^{1}H-^{1}H COSY spectrum of compound 10 .... S52

Figure S93: ROESY spectrum of compound 10 .... S53

Figure S94: UV spectrum of compound 10 .... S53

Figure S95: HR-ESI-MS of compound 10 .... S54

Figure S96: CD spectrum of compound 10 .... S54

Figure S97: ^{1}H-NMR (600 MHz, $CDCl_3$) spectrum of compound 11 .... S55

Figure S98: ^{13}C-NMR (150 MHz, $CDCl_3$) spectrum of compound 11 .... S55

Figure S99: ^{13}C-NMR-DEPT (θ=135°) spectrum of compound 11 .... S56

Figure S100: HSQC spectrum of compound 11 .... S56

Figure S101: HMBC spectrum of compound 11 .... S57

Figure S102: ROESY spectrum of compound 11 .... S57

Figure S103: UV spectrum of compound 11 .... S58

Figure S104: HR-ESI-MS of compound 11 .... S58

Figure S105: CD spectrum of compound 11 .... S59

Figure S106: ^{1}H-NMR (600 MHz, $CDCl_3$) spectrum of compound 12 .... S59

Figure S107: ^{13}C-NMR (150 MHz, $CDCl_3$) spectrum of compound 12 .... S60

Figure S108: ^{13}C-NMR-DEPT (θ=135°) spectrum of compound 12 .... S60

Figure S109: HSQC spectrum of compound 12 .... S61

Figure S110: HMBC spectrum of compound 12 .... S61

Figure S111: ^{1}H-^{1}H COSY spectrum of compound 12 .... S62

Figure S112: ROESY spectrum of compound 12 .... S62

Figure S113: UV spectrum of compound 12 .... S63

Figure S114: HR-ESI-MS of compound 12..........S63

Figure S115: CD spectrum of compound 12..........S64

Figure S116: ^{1}H-NMR (600 MHz, $CDCl_3$) spectrum of compound 13..........S64

Figure S117: ^{13}C-NMR (150 MHz, $CDCl_3$) spectrum of compound 13..........S65

Figure S118: ^{13}C-NMR-DEPT (θ=135°) spectrum of compound 13..........S65

Figure S119: HSQC spectrum of compound 13..........S66

Figure S120: HMBC spectrum of compound 13..........S66

Figure S121: ^{1}H-^{1}H COSY spectrum of compound 13..........S67

Figure S122: ROESY spectrum of compound 13..........S67

Figure S123: UV spectrum of compound 13..........S68

Figure S124: HR-ESI-MS of compound 13..........S68

Figure S125: CD spectrum of compound 13..........S69

Figure S126: ^{1}H-NMR (600 MHz, $CDCl_3$) spectrum of compound 14..........S69

Figure S127: ^{13}C-NMR (150 MHz, $CDCl_3$) spectrum of compound 14..........S70

Figure S128: ^{13}C-NMR-DEPT (θ=135°) spectrum of compound 14..........S70

Figure S129: HSQC spectrum of compound 14..........S71

Figure S130: HMBC spectrum of compound 14..........S71

Figure S131: ROESY spectrum of compound 14..........S72

Figure S132: UV spectrum of compound 14..........S72

Figure S133: HR-ESI-MS of compound 14..........S73

Figure S134: CD spectrum of compound 14..........S73

Table S1. Antiproliferative activities of compounds 1-24..........S74

1. Computational methods..........S79

2. Energies and Coordinates..........S87

3. Experimental and Computed NMR Chemical Shifts..........S168

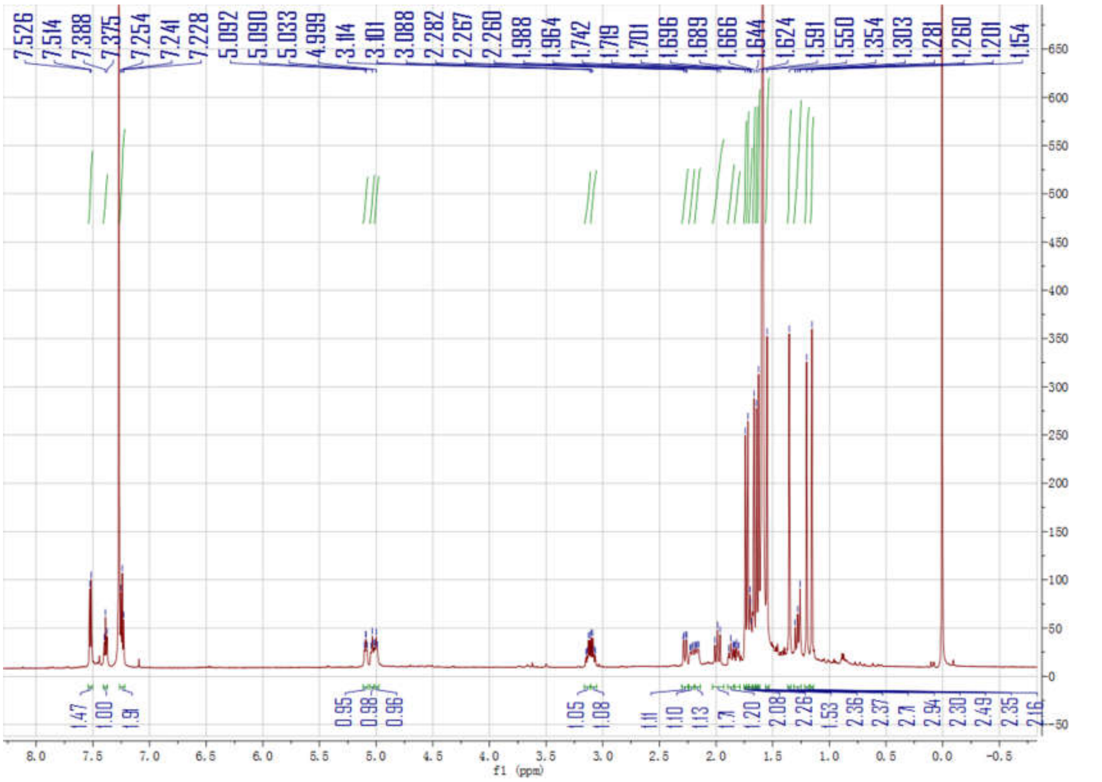

Figure S1: ^{1}H-NMR (600 MHz, $CDCl_3$) spectrum of compound 1

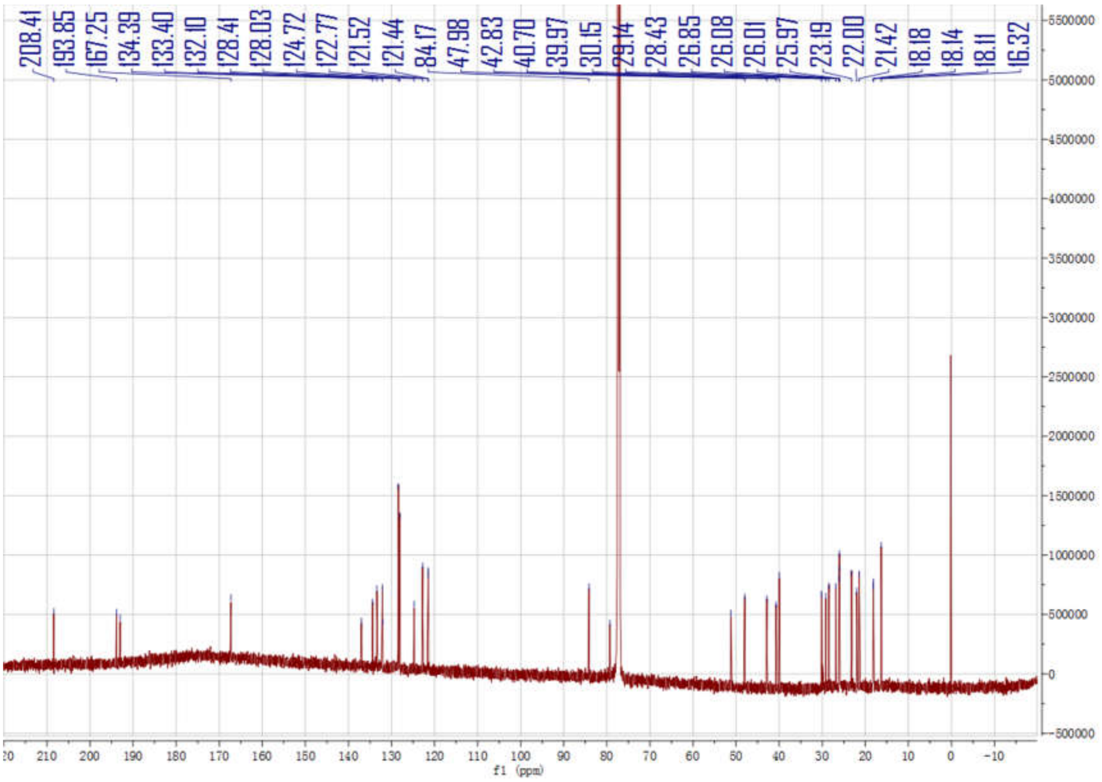

Figure S2: ^{13}C-NMR (150 MHz, $CDCl_3$) spectrum of compound 1

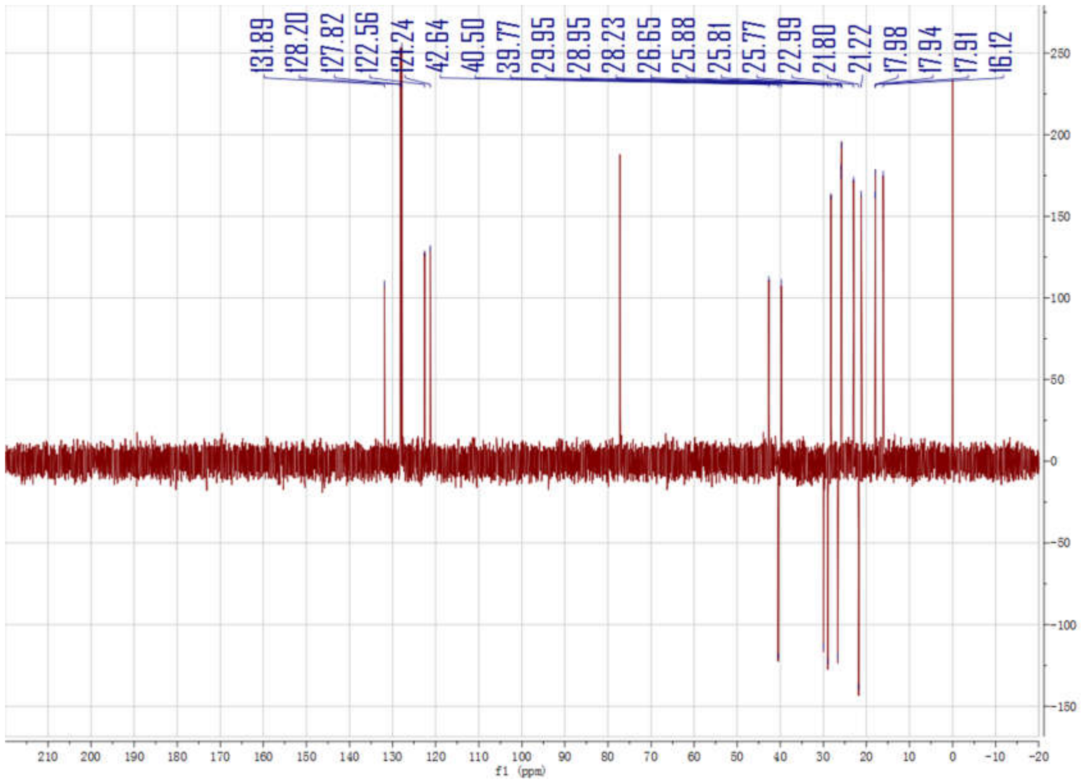

Figure S3: ^{13}C-NMR-DEPT (θ=135°) spectrum of compound 1

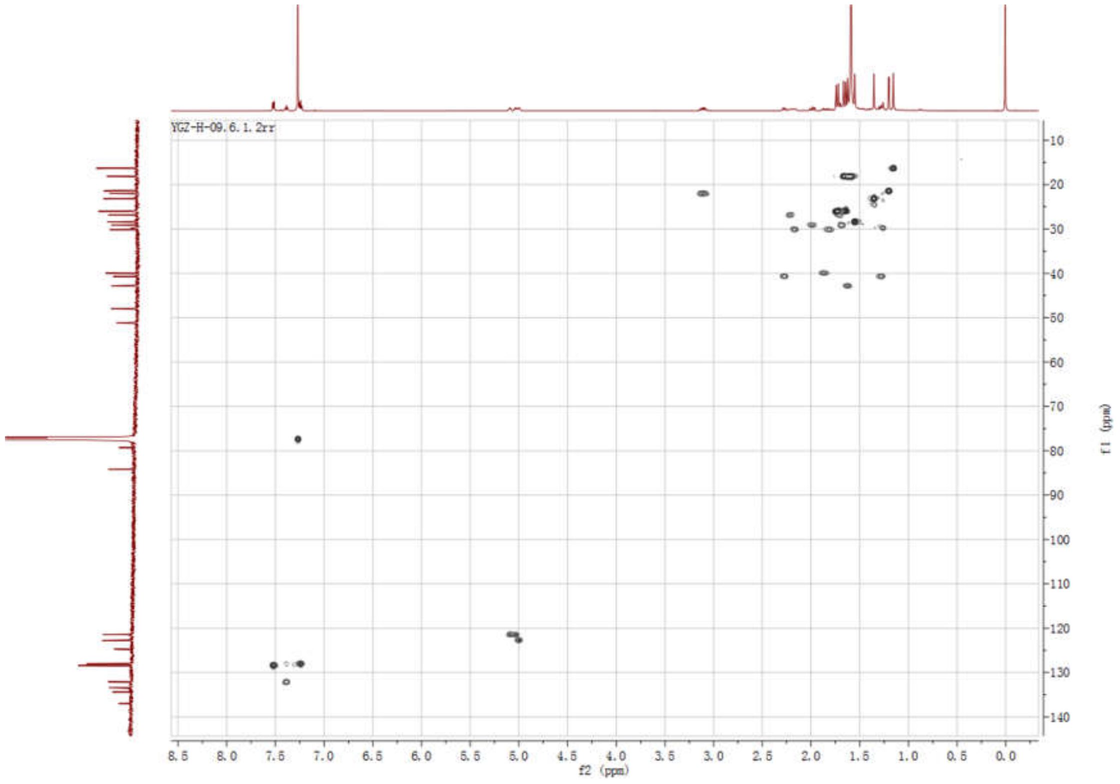

Figure S4: HSQC spectrum of compound 1

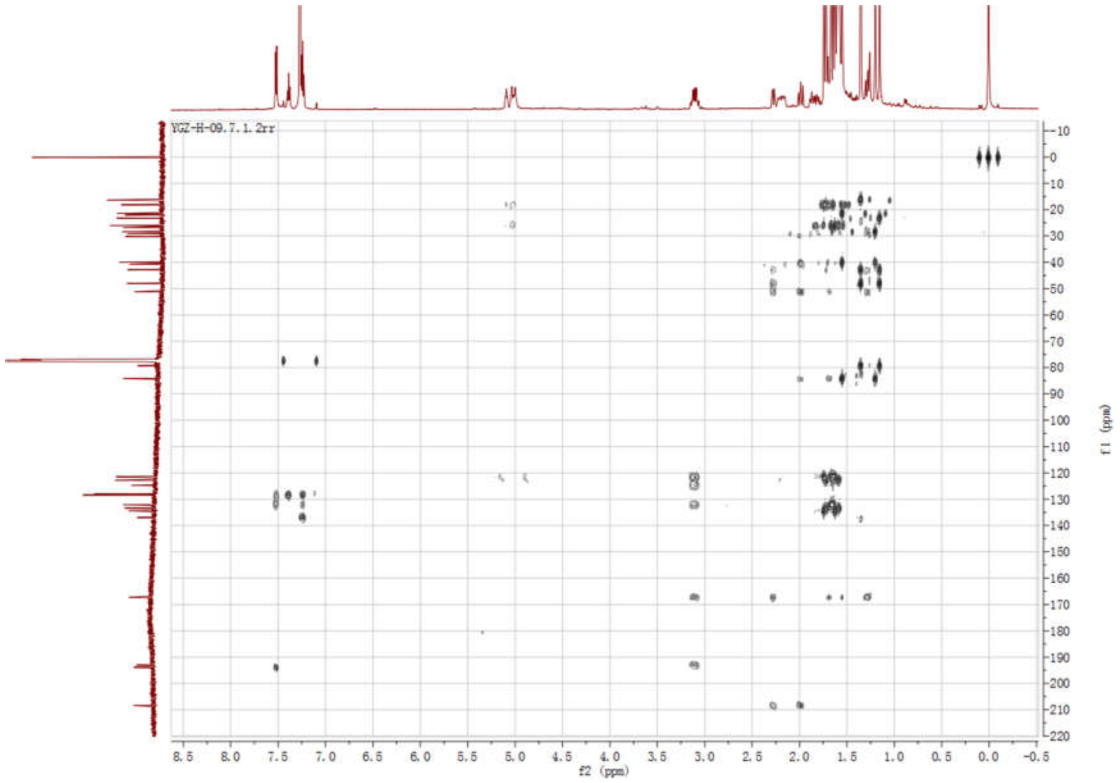

Figure S5: HMBC spectrum of compound 1

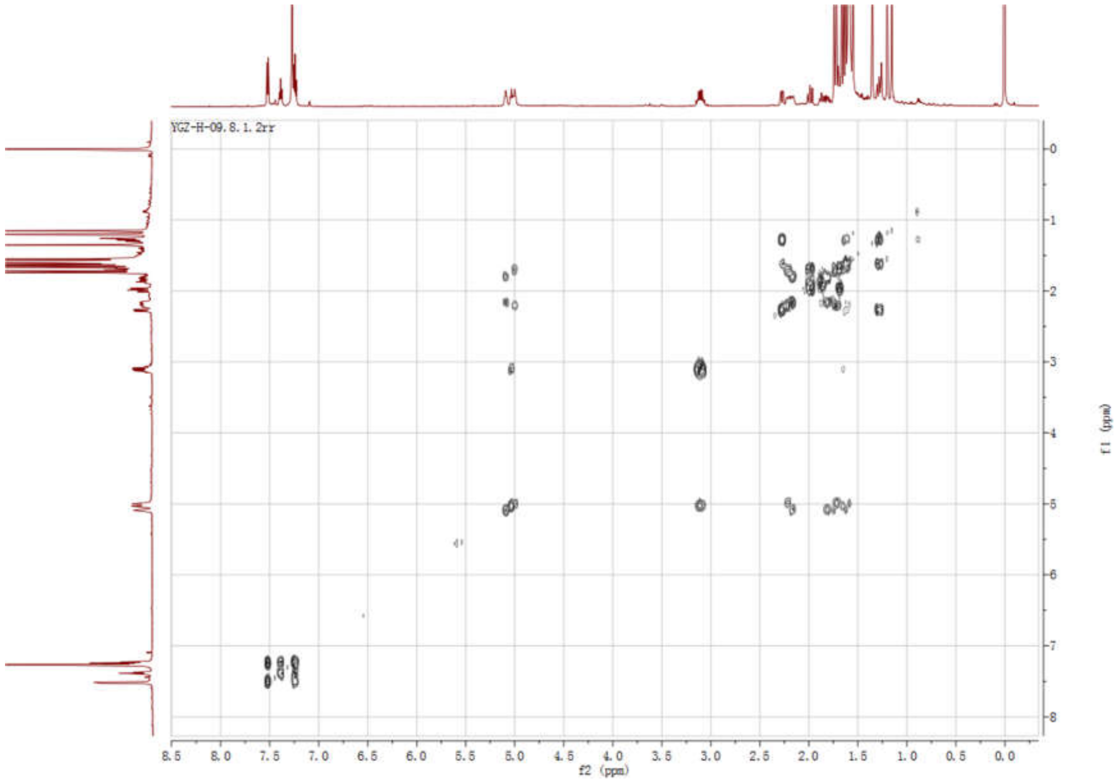

Figure S6: ^{1}H-^{1}H COSY spectrum of compound 1

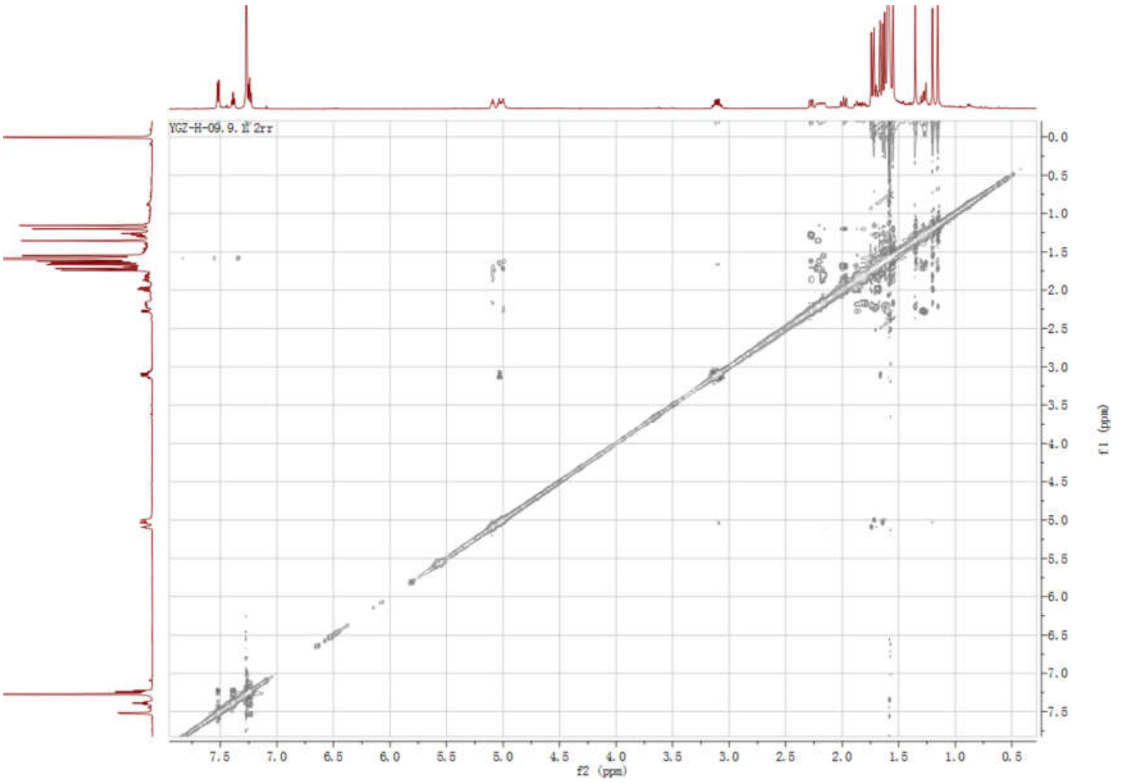

Figure S7: ROESY spectrum of compound 1

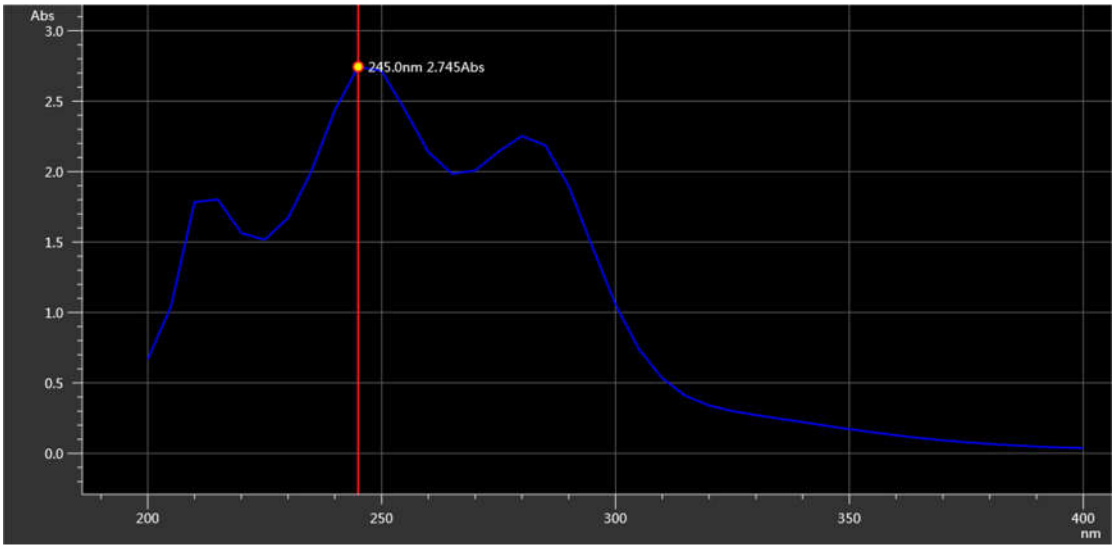

Figure S8: UV spectrum of compound 1

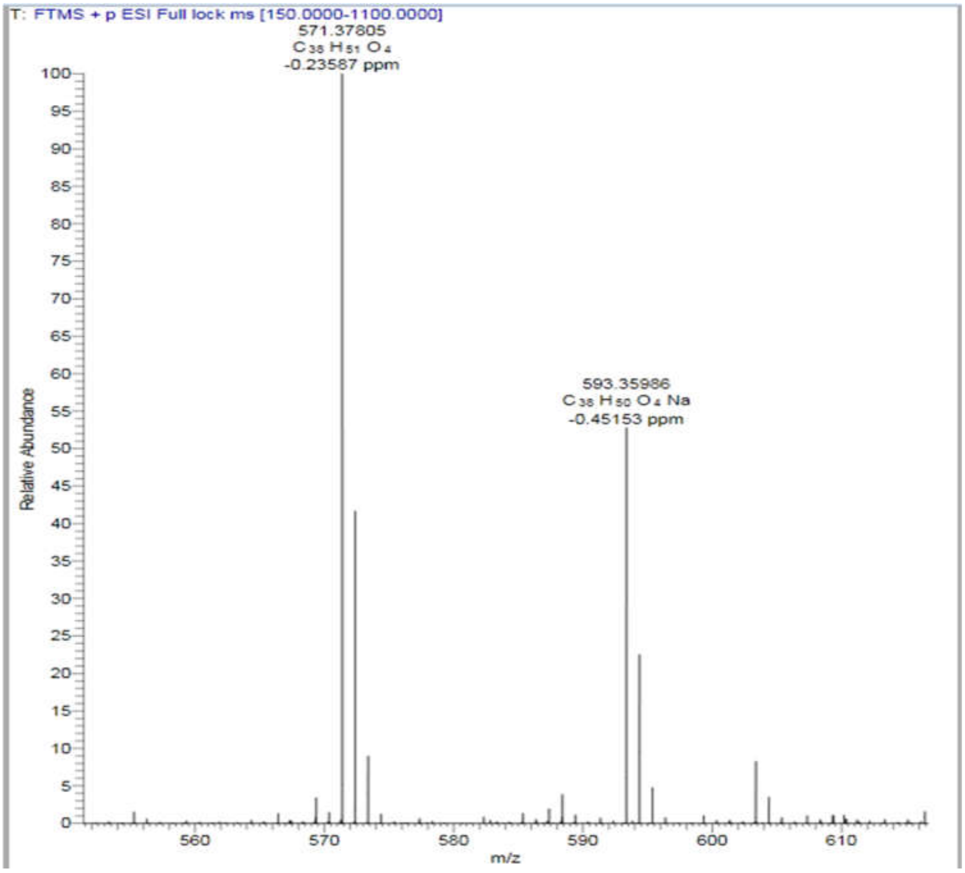

Figure S9: HR-ESI-MS of compound 1

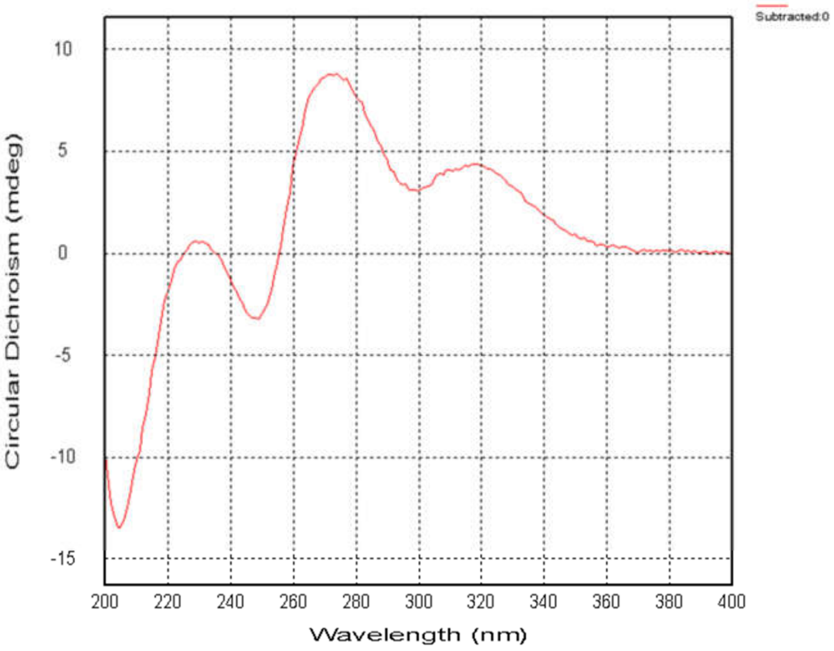

Figure S10: CD spectrum of compound 1

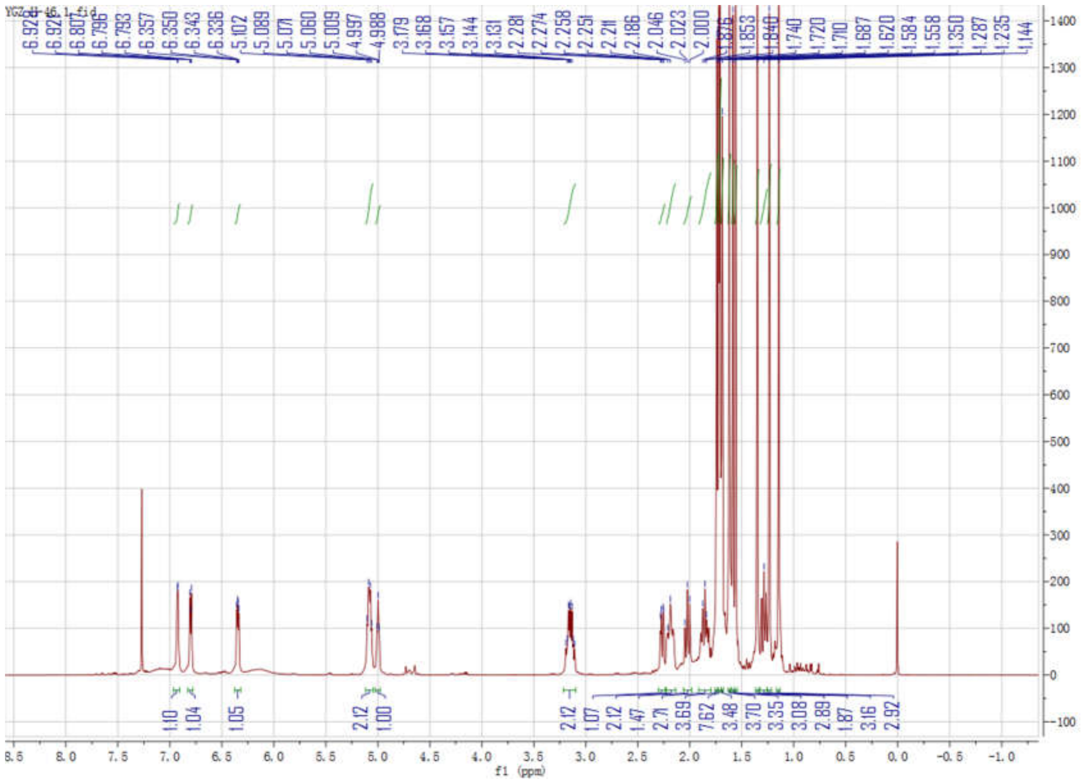

Figure S11: ^{1}H-NMR (600 MHz, $CDCl_3$) spectrum of compound 2

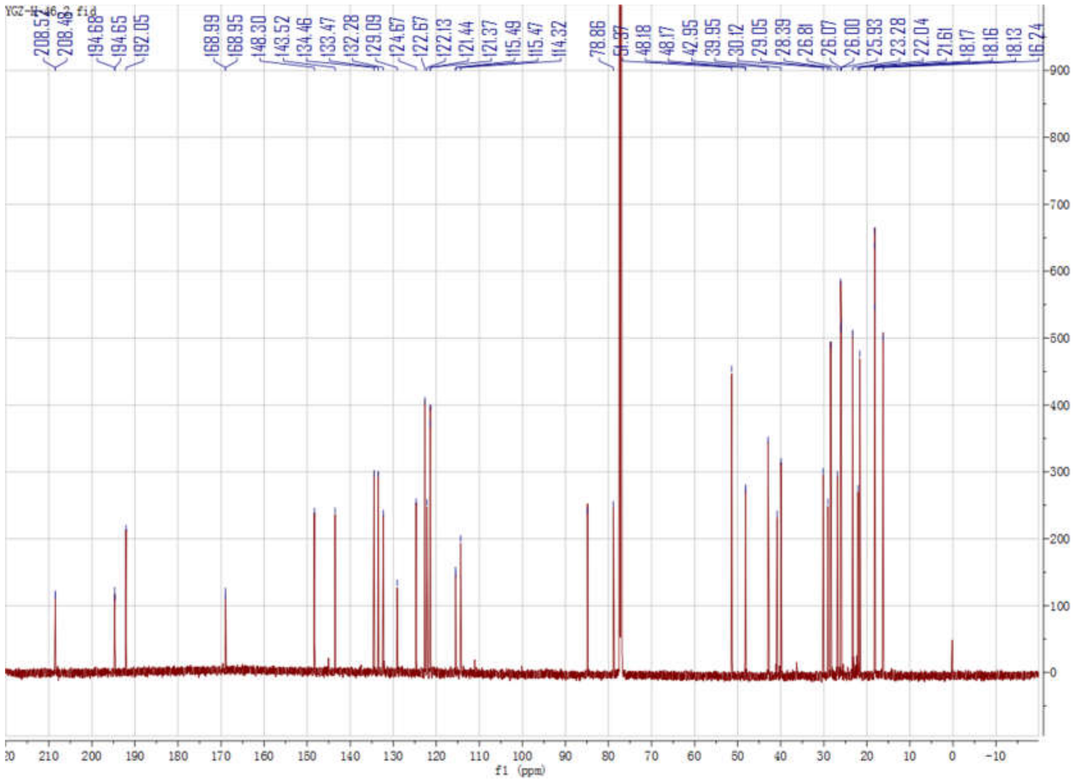

Figure S12: ^{13}C-NMR (150 MHz, $CDCl_3$) spectrum of compound 2

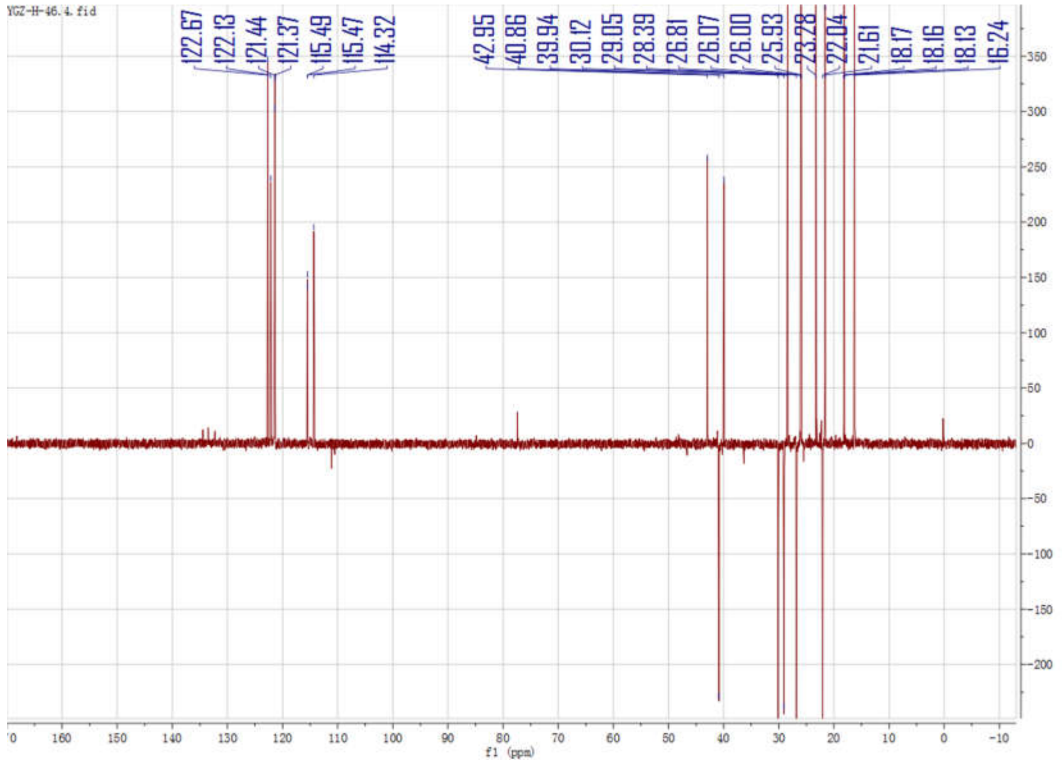

Figure S13: ^{13}C-NMR-DEPT (θ=135°) spectrum of compound 2

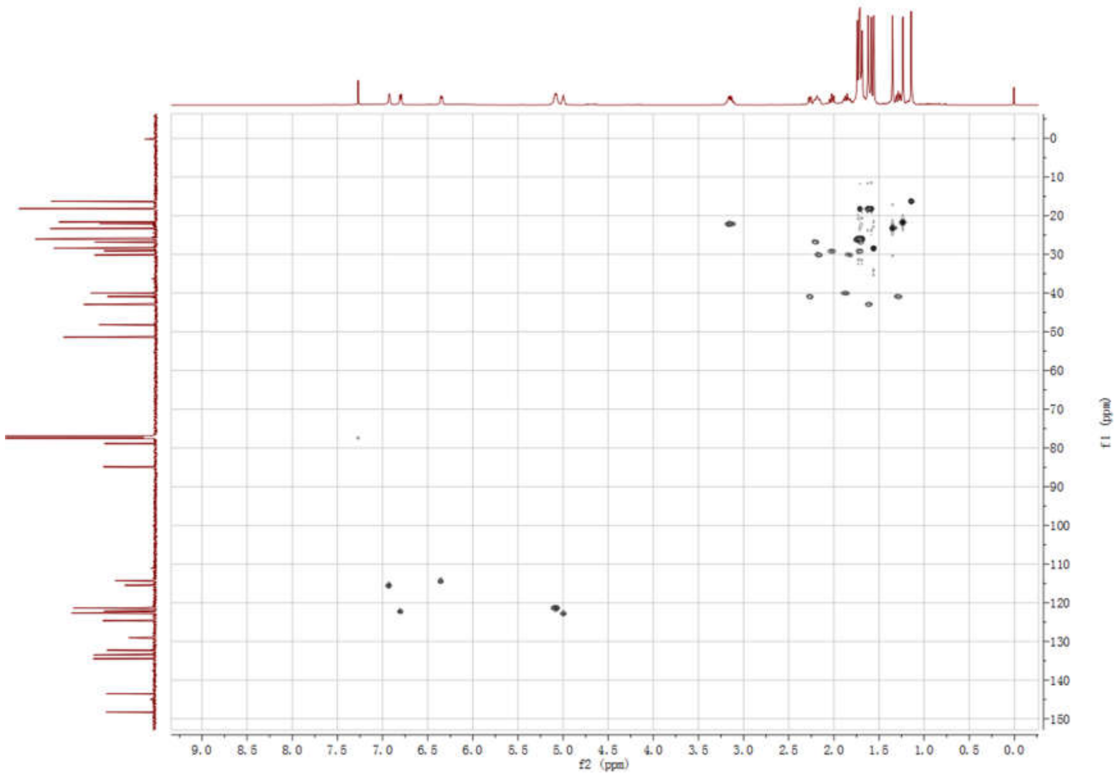

Figure S14: HSQC spectrum of compound 2

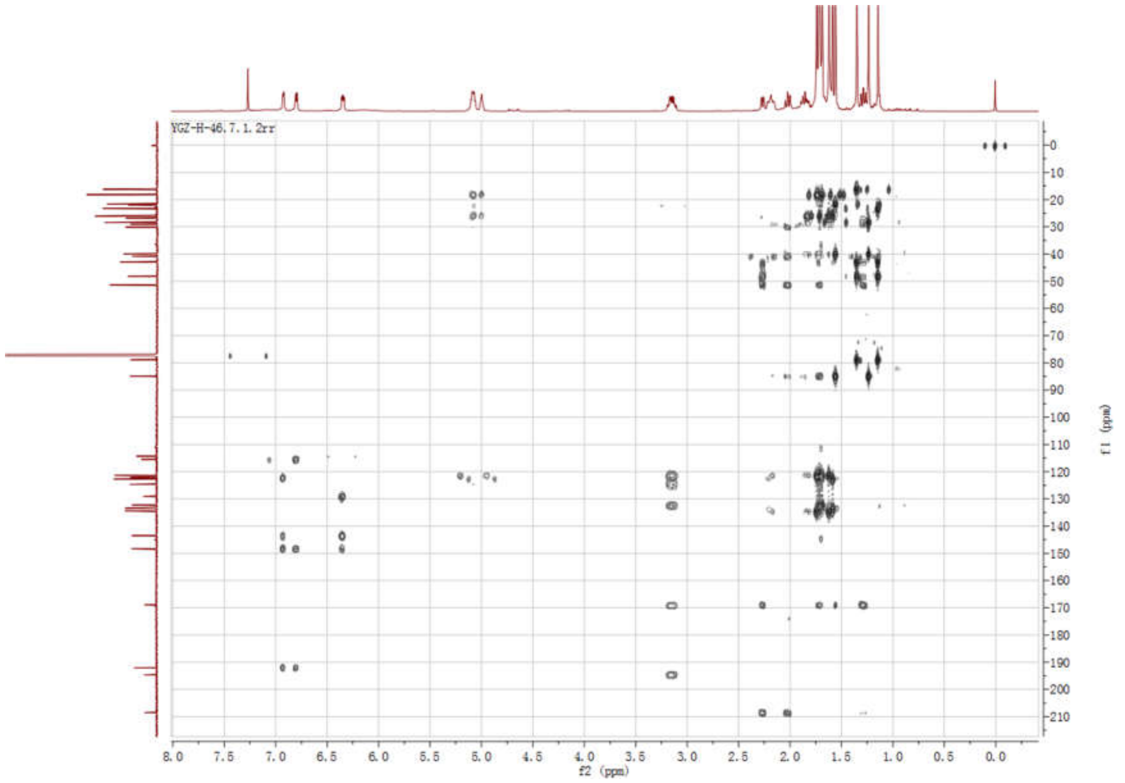

Figure S15: HMBC spectrum of compound 2

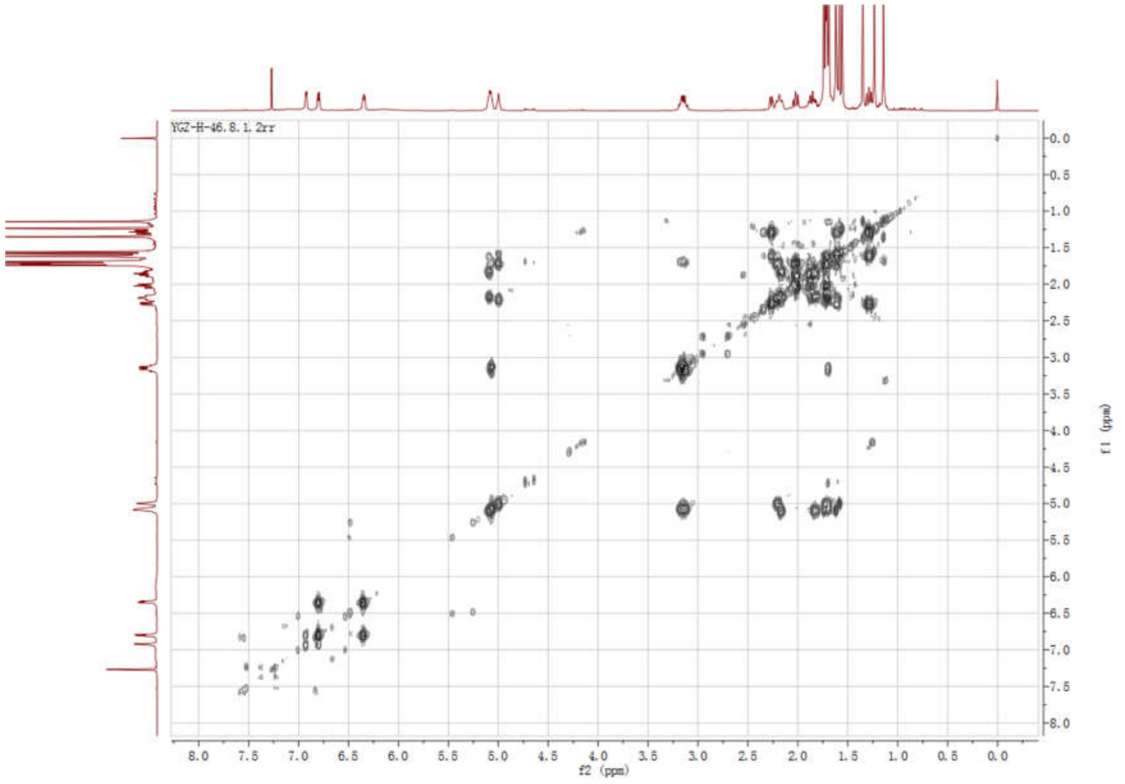

Figure S16: ^{1}H-^{1}H COSY spectrum of compound 2

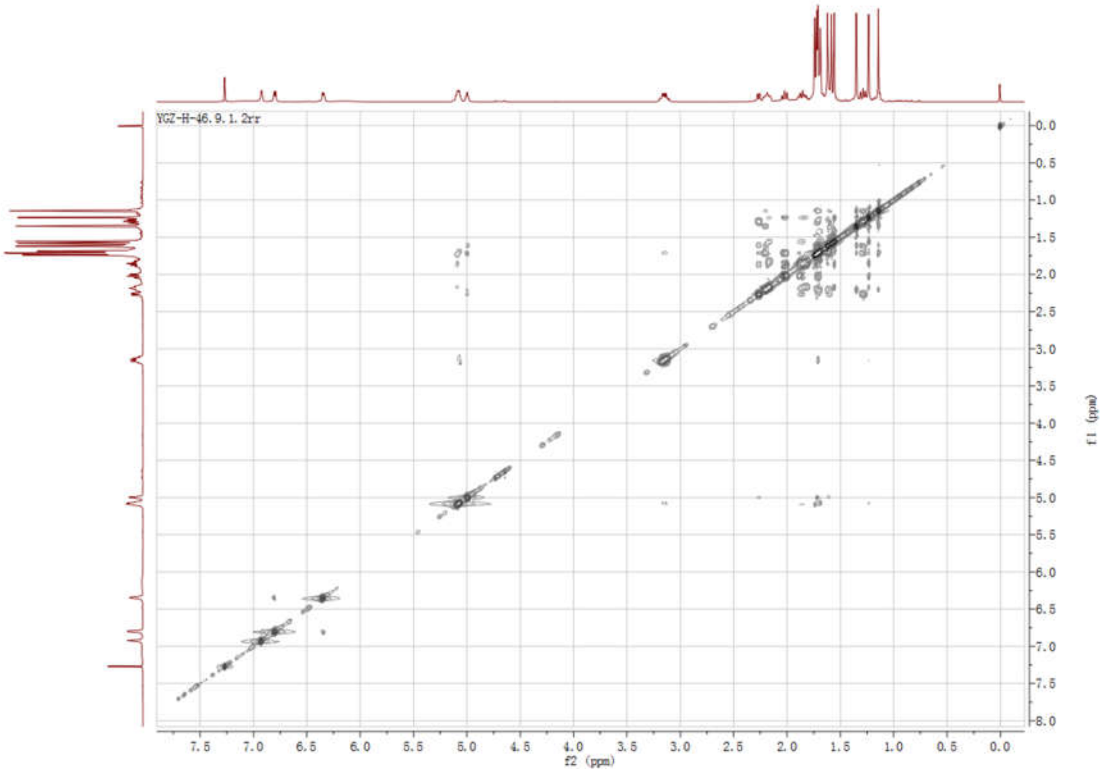

Figure S17: ROESY spectrum of compound 2

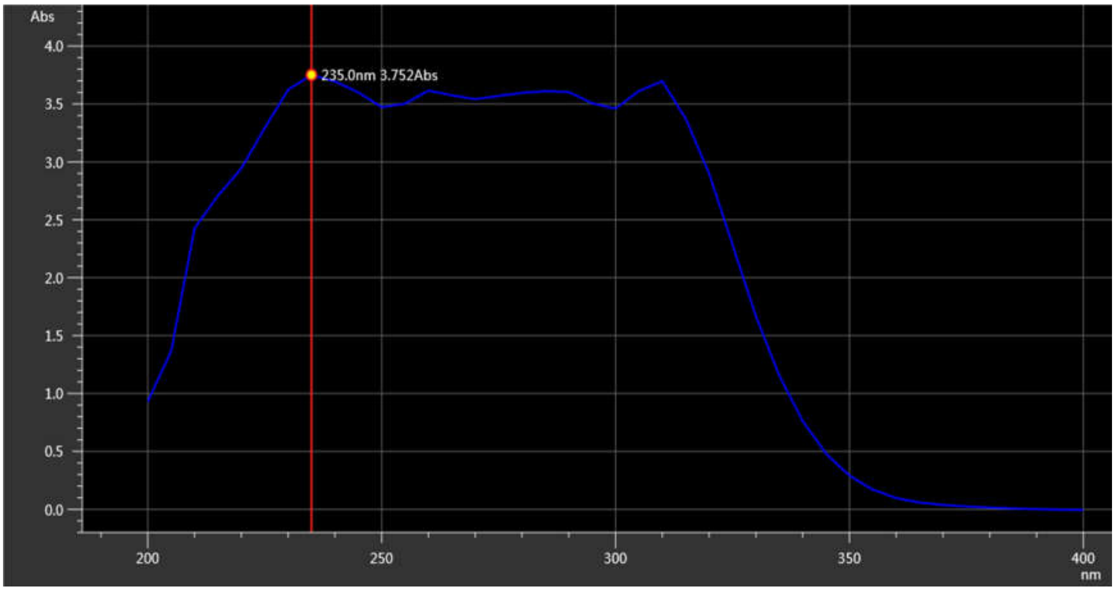

Figure S18: UV spectrum of compound 2

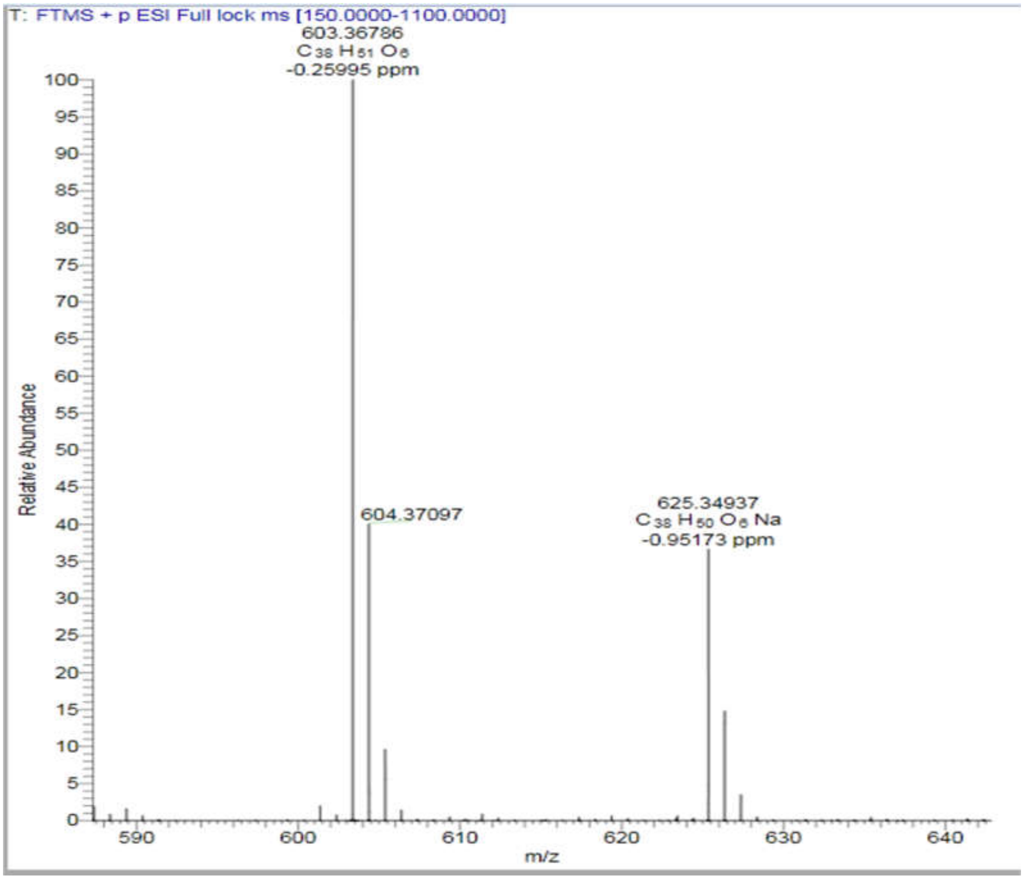

Figure S19: HR-ESI-MS of compound 2

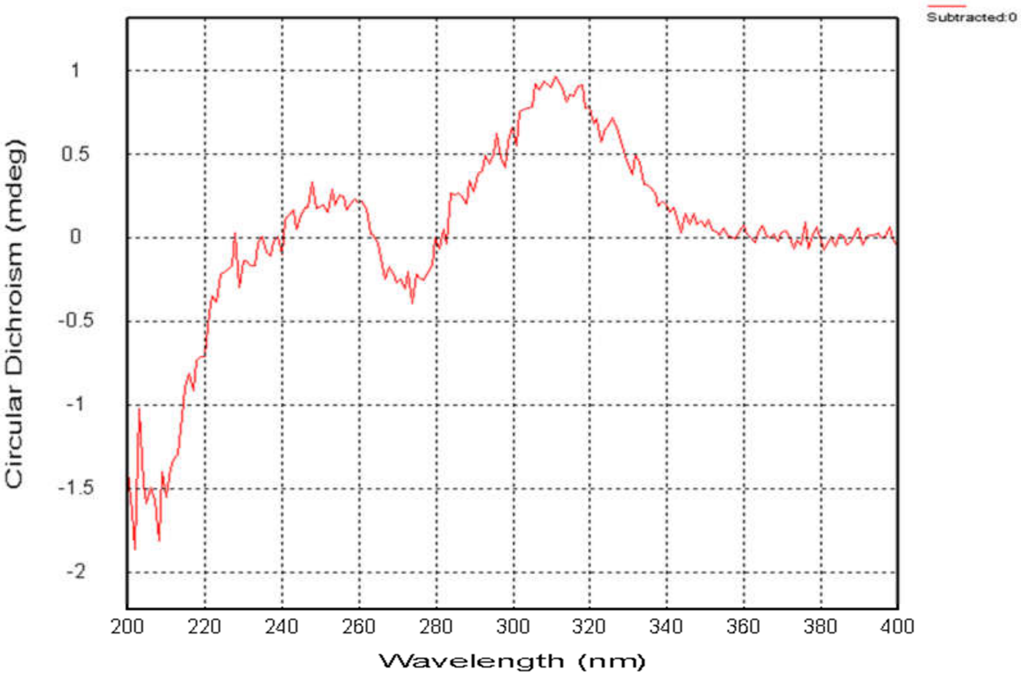

Figure S20: CD spectrum of compound 2

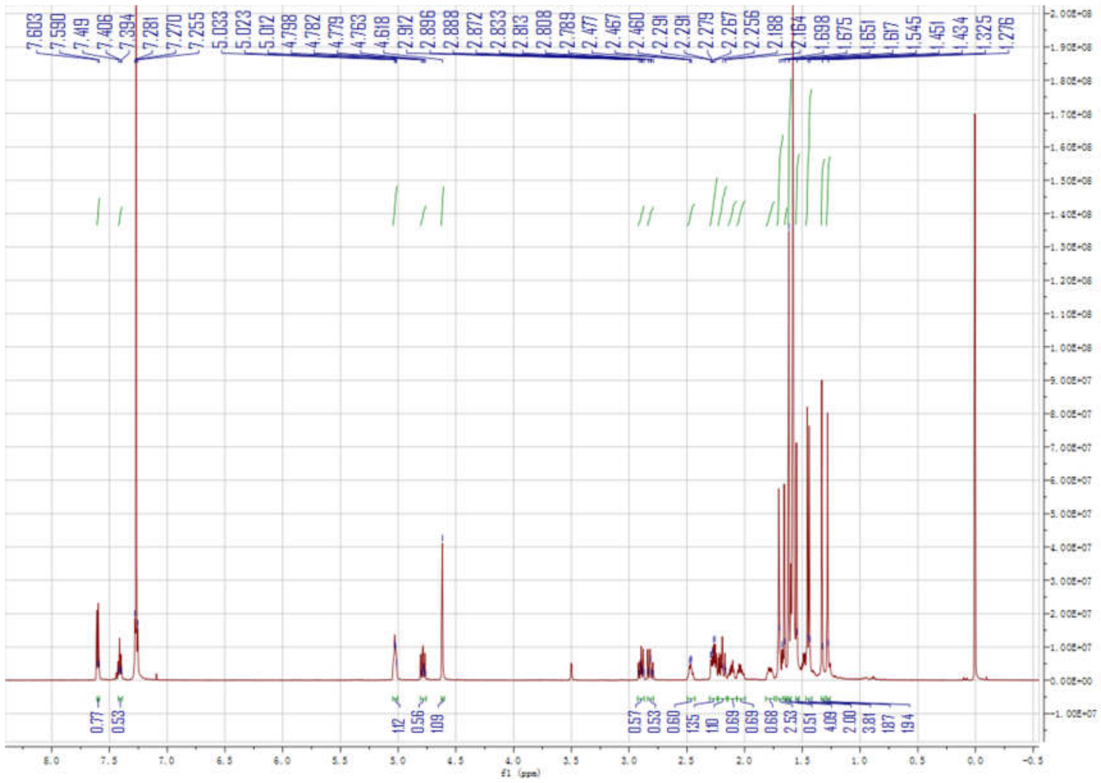

Figure S21: ^{1}H-NMR (600 MHz, $CDCl_3$) spectrum of compound 3

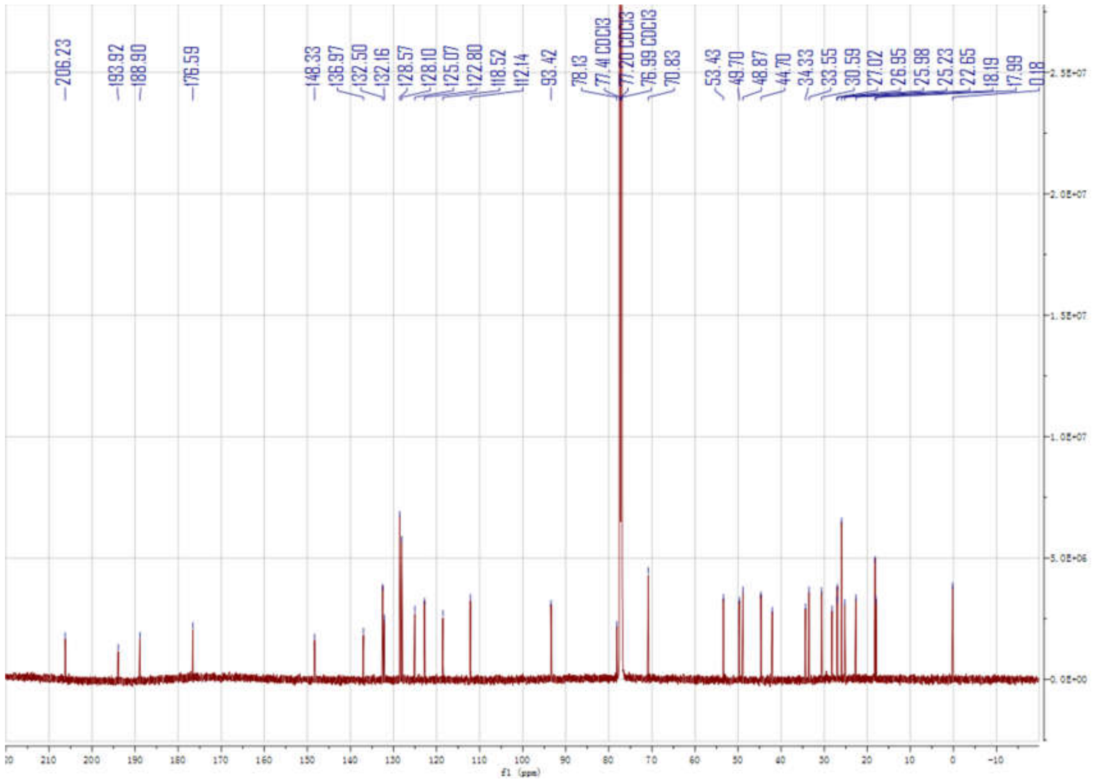

Figure S22: The ^{13}C-NMR (150 MHz, $CDCl_3$) spectrum of compound 3

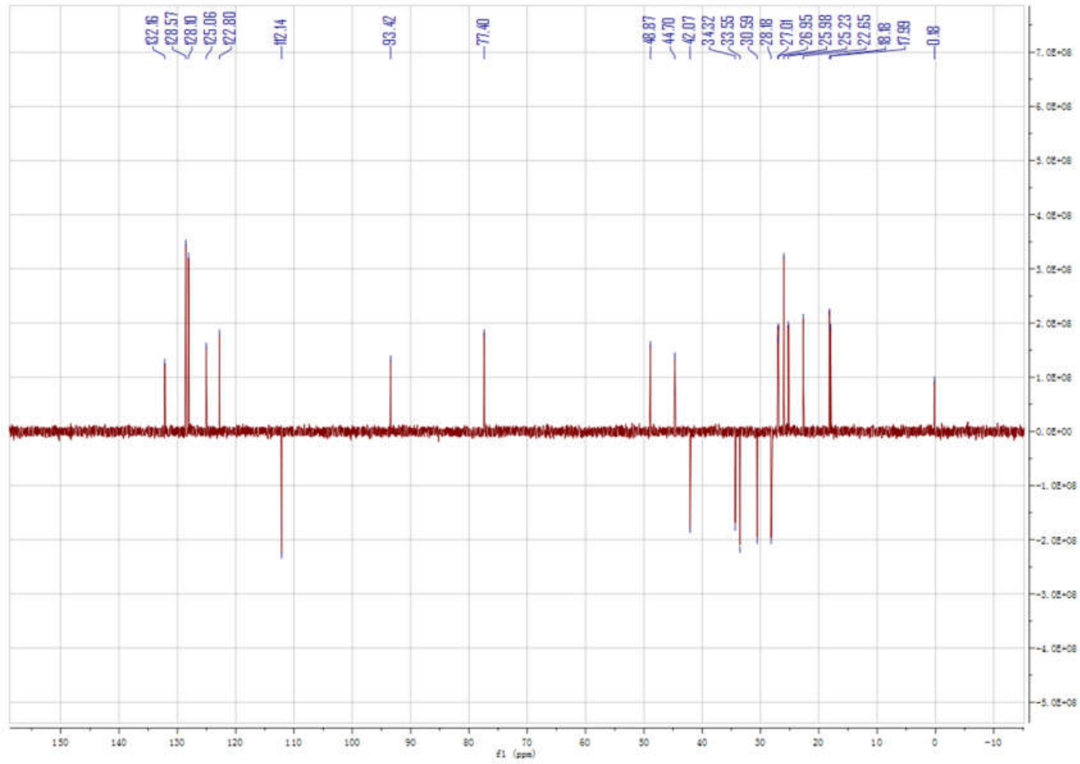

Figure S23: ^{13}C-NMR-DEPT (θ=135°) spectrum of compound 3

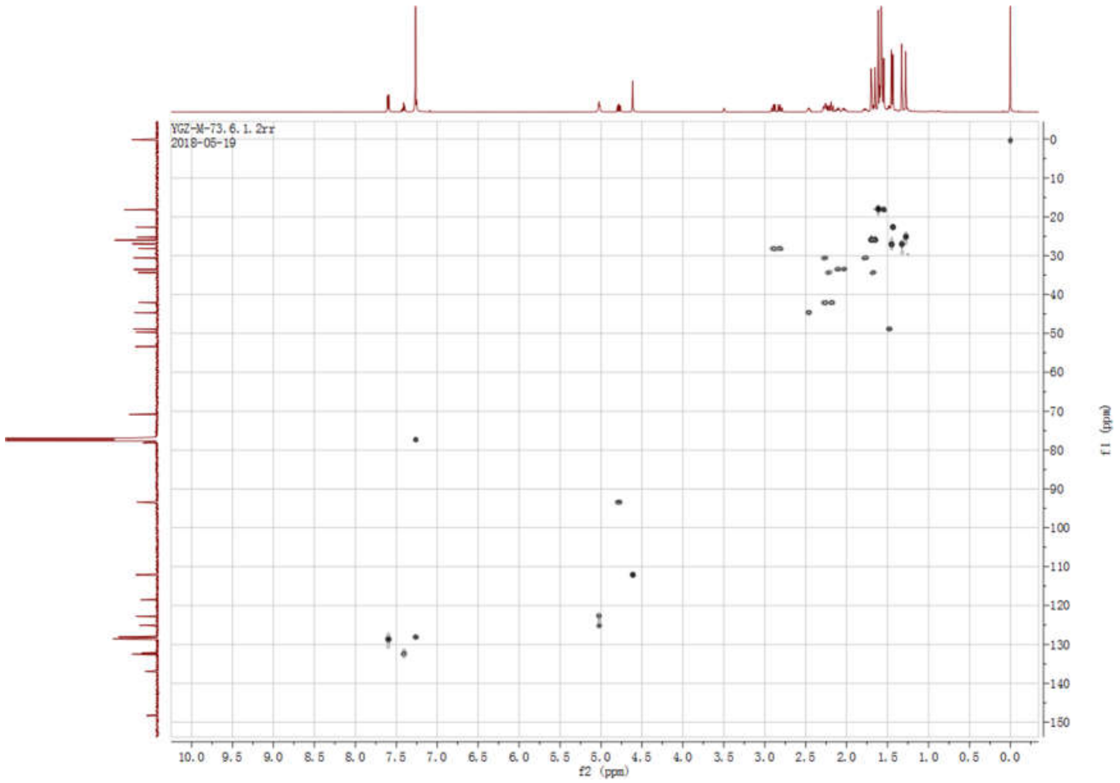

Figure S24: HSQC spectrum of compound 3

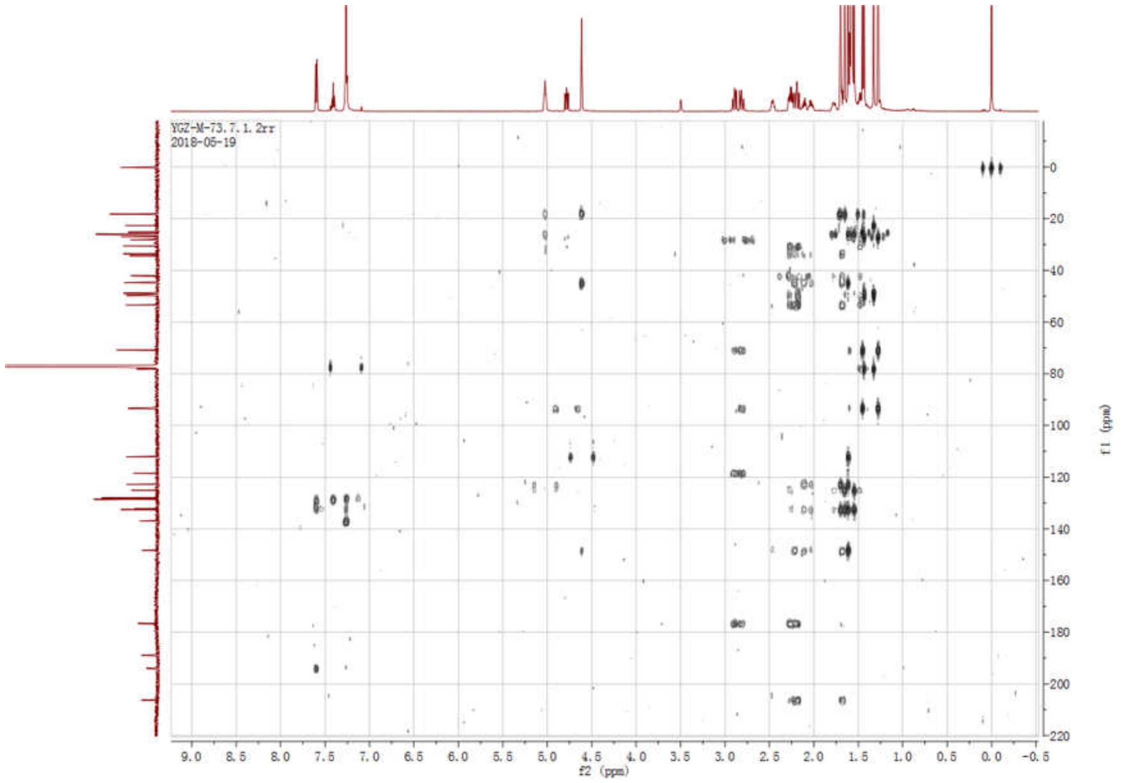

Figure S25: HMBC spectrum of compound 3

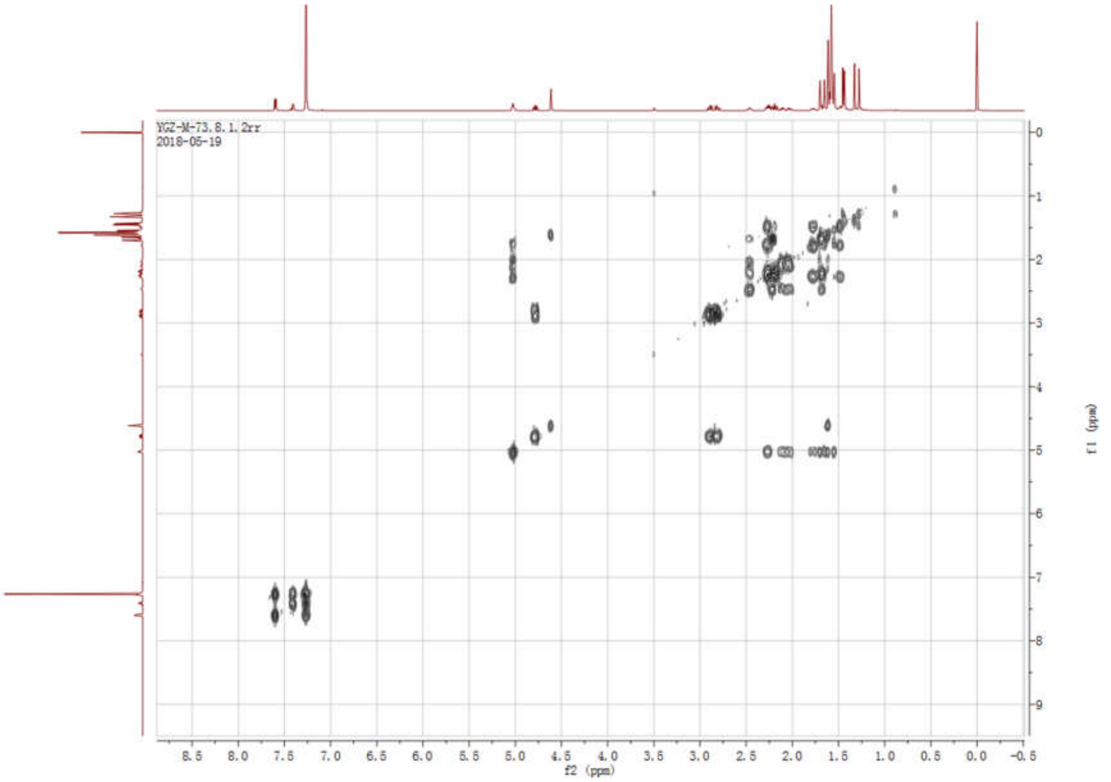

Figure S26: ^{1}H-^{1}H COSY spectrum of compound 3

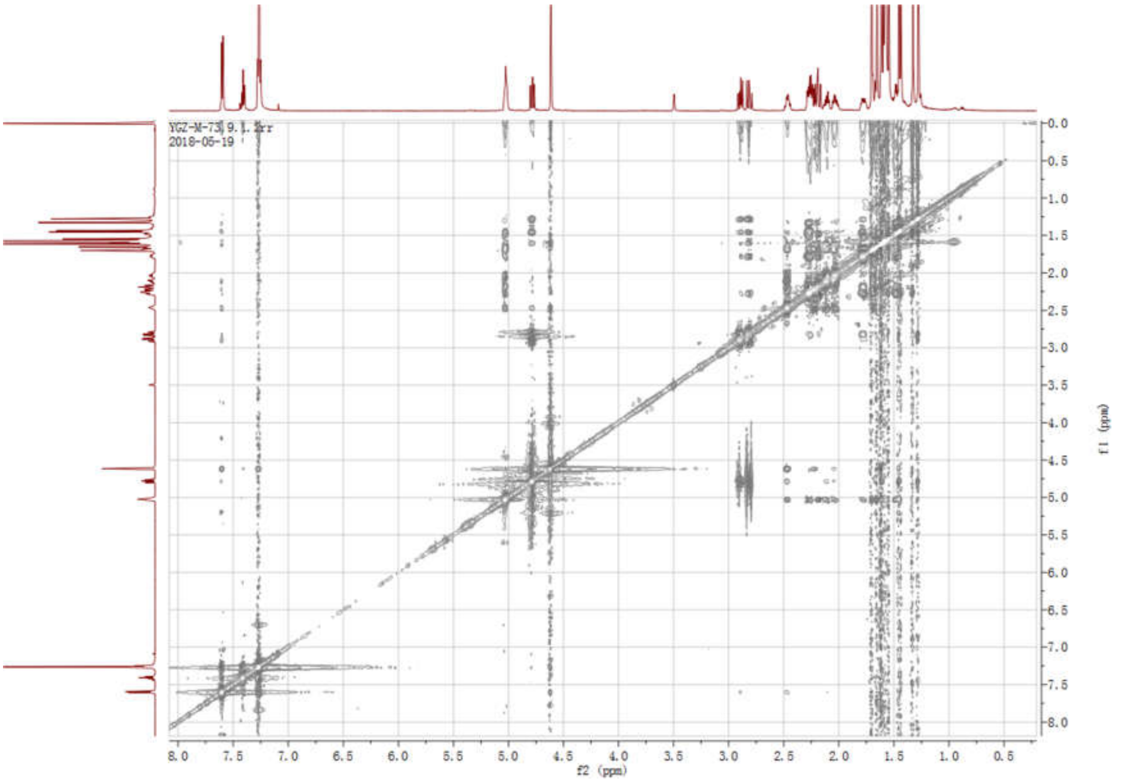

Figure S27: ROESY spectrum of compound 3

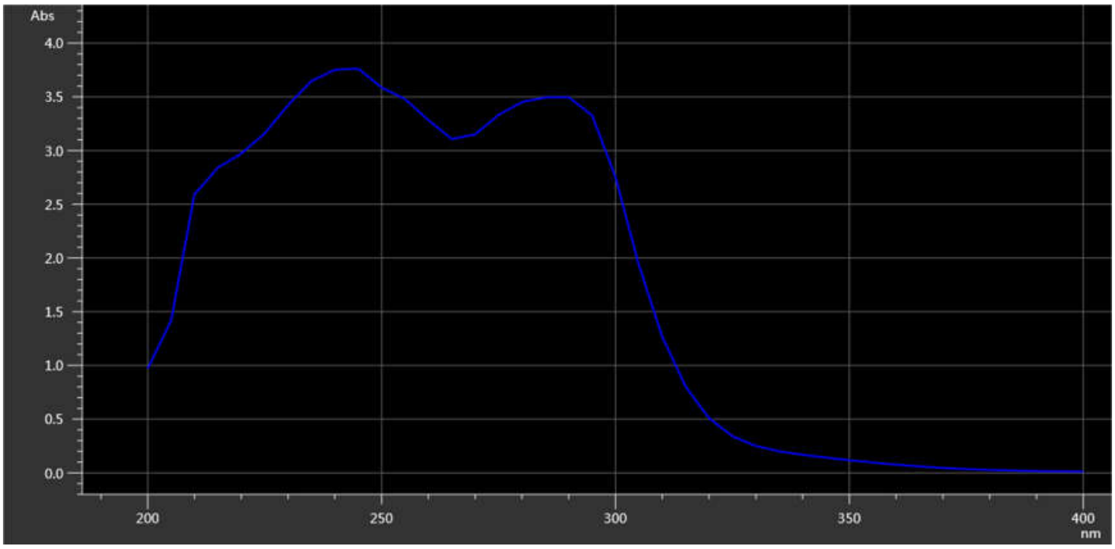

Figure S28: UV spectrum of compound 3

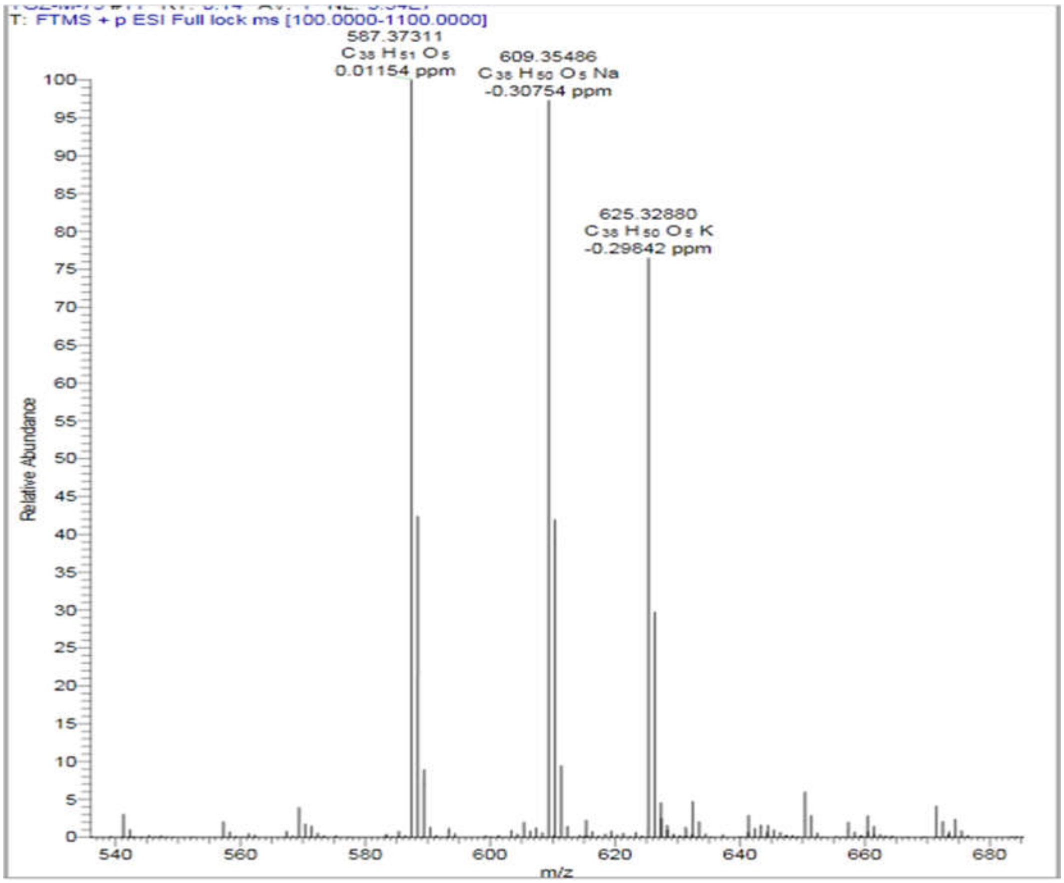

Figure S29: HR-ESI-MS of compound 3

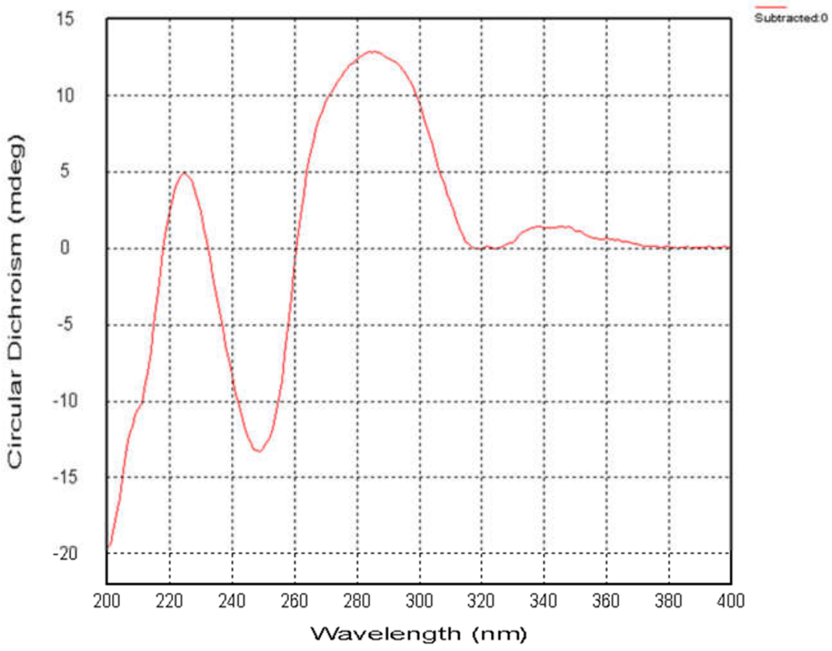

Figure S30: CD spectrum of compound 3

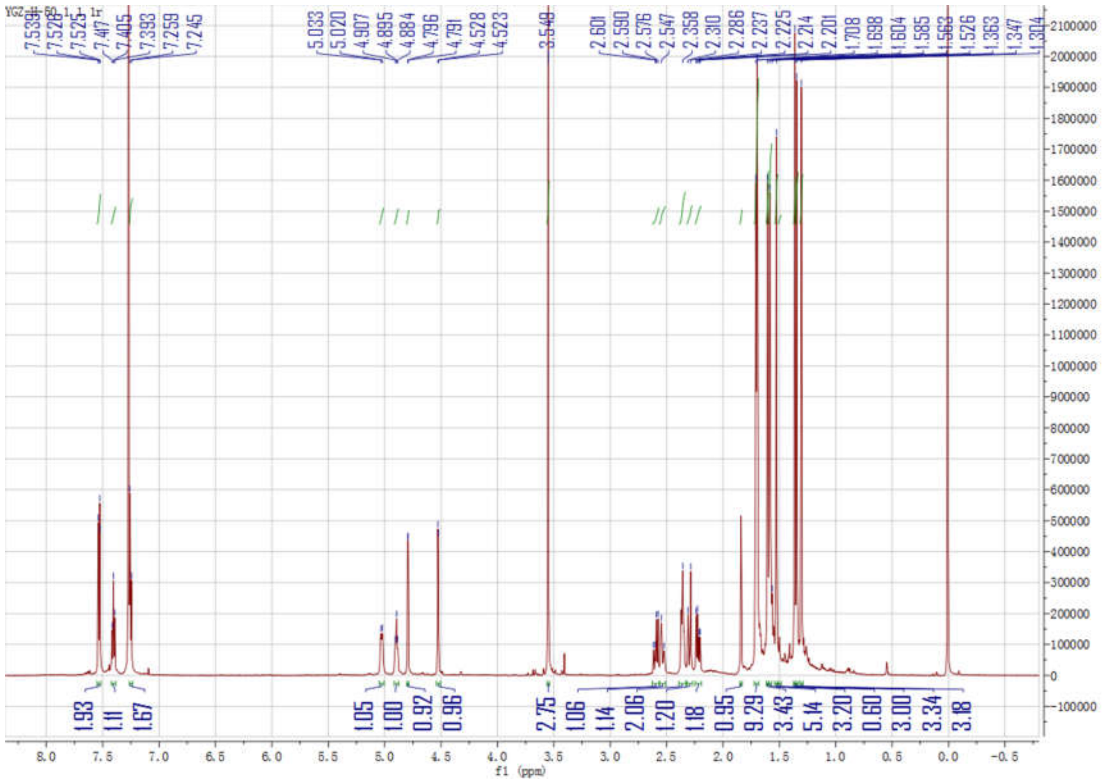

Figure S31: ^{1}H-NMR (600 MHz, $CDCl_3$) spectrum of compound 4

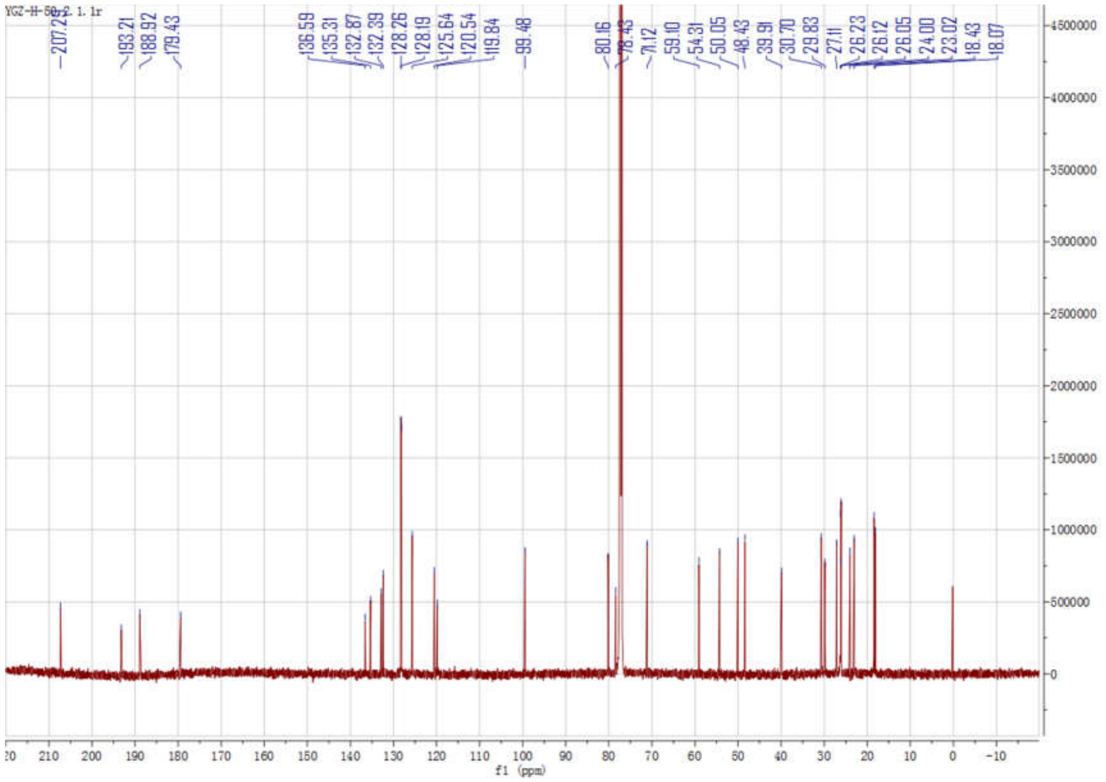

Figure S32: The ^{13}C-NMR (150 MHz, $CDCl_3$) spectrum of compound 4

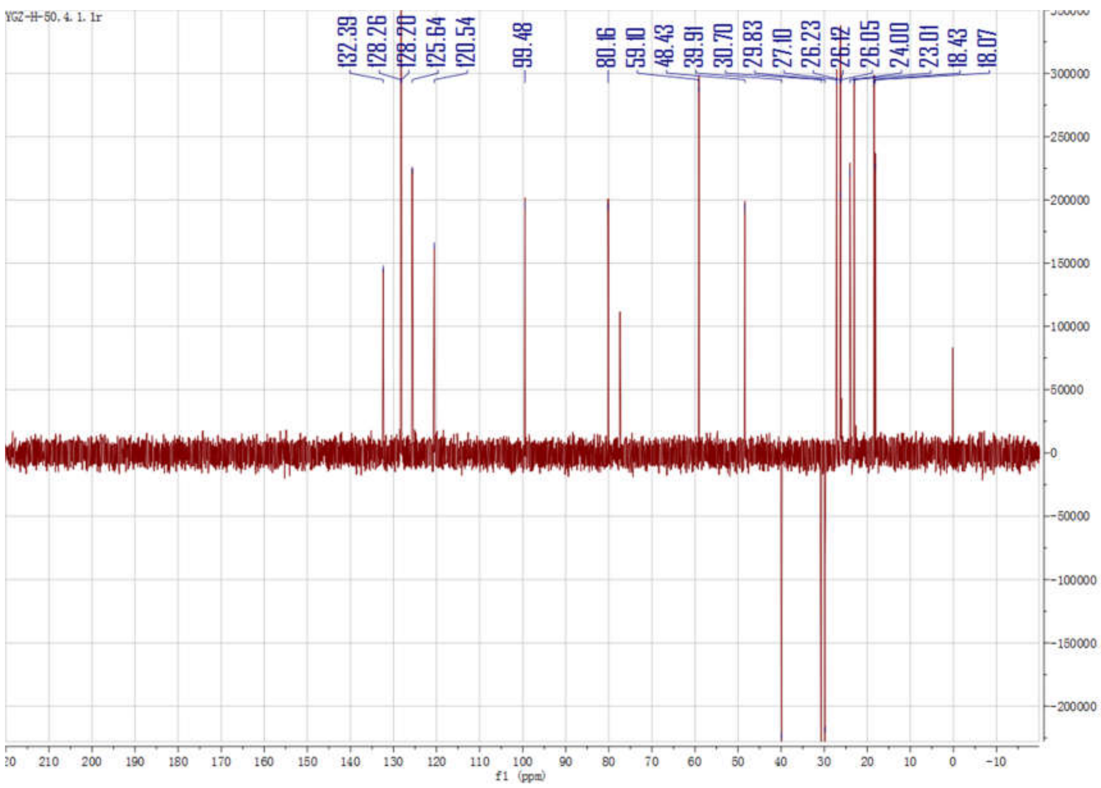

Figure S33: ^{13}C-NMR-DEPT (θ=135°) spectrum of compound 4

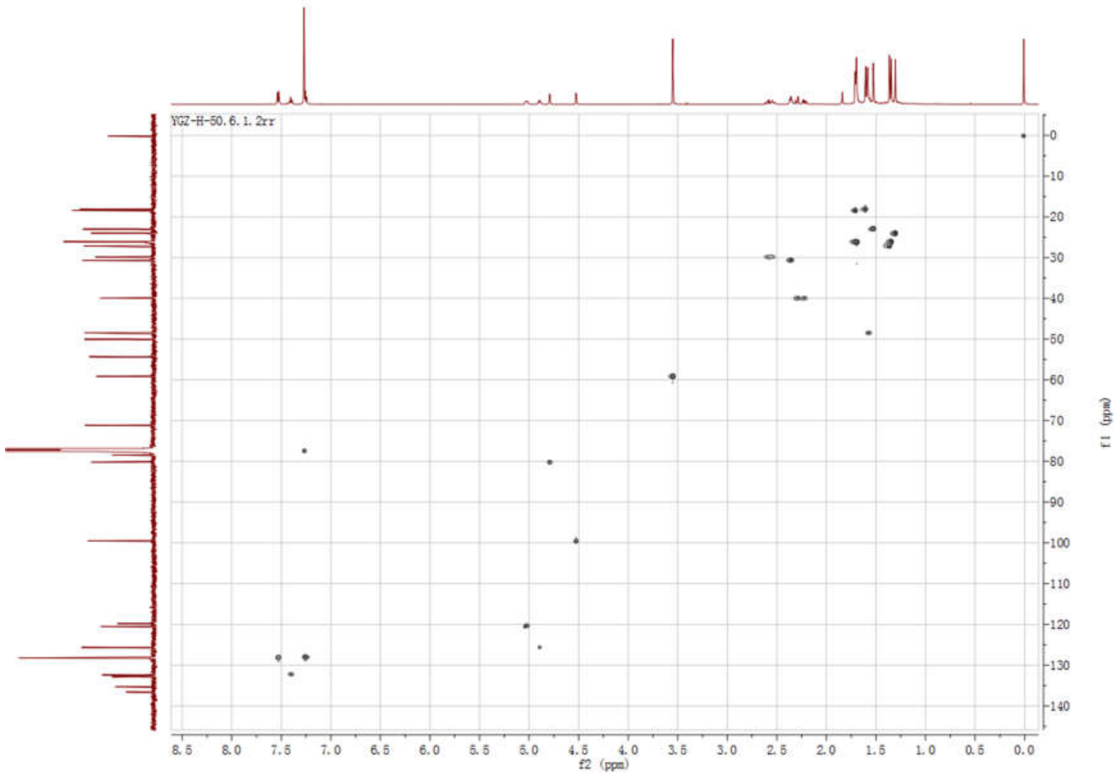

Figure S34: HSQC spectrum of compound 4

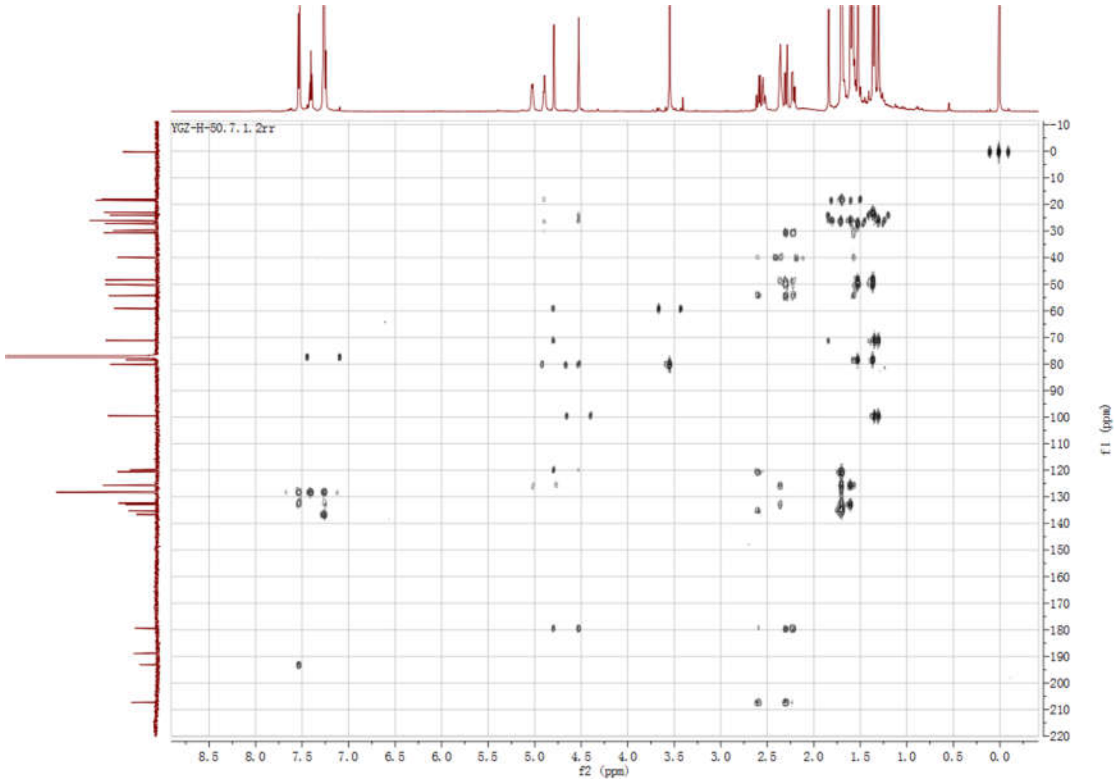

Figure S35: HMBC spectrum of compound 4

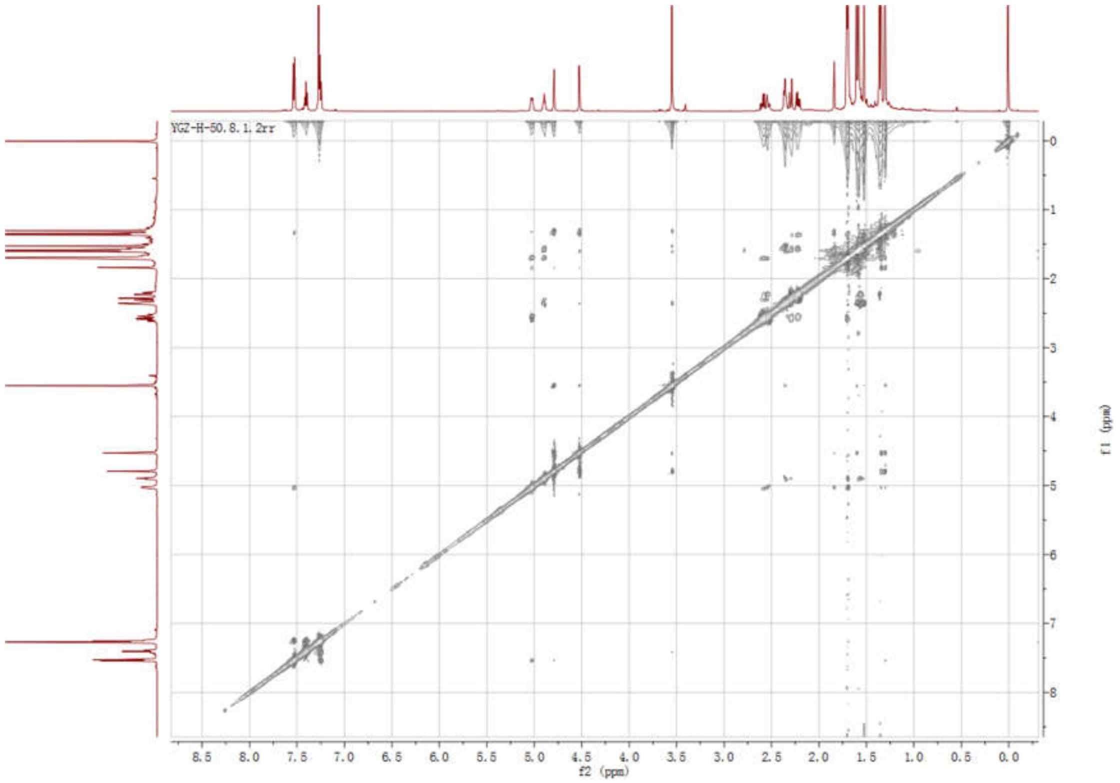

Figure S36: ROESY spectrum of compound 4

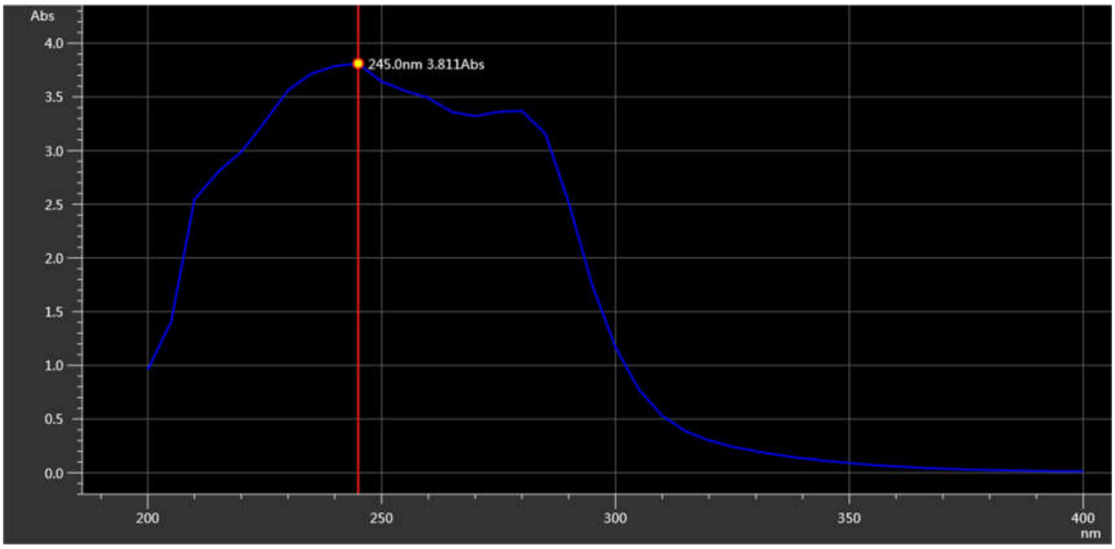

Figure S37: UV spectrum of compound 4

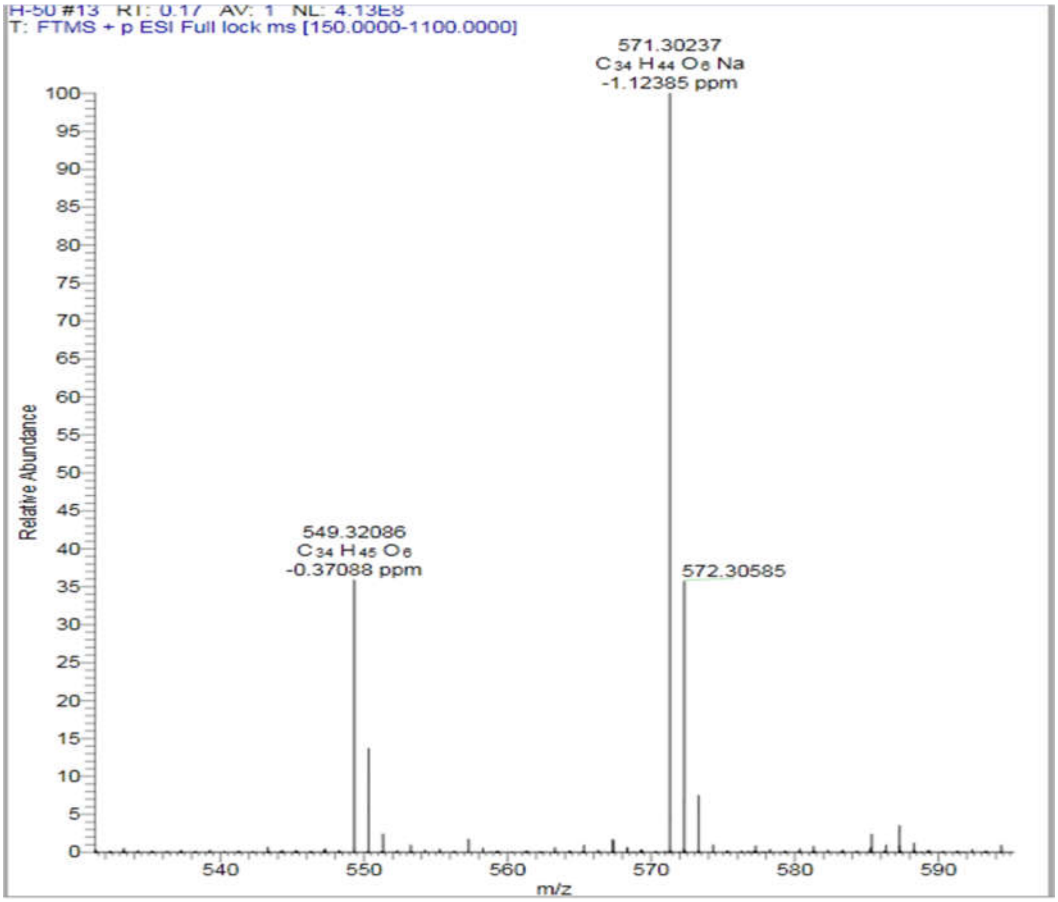

Figure S38: HR-ESI-MS of compound 4

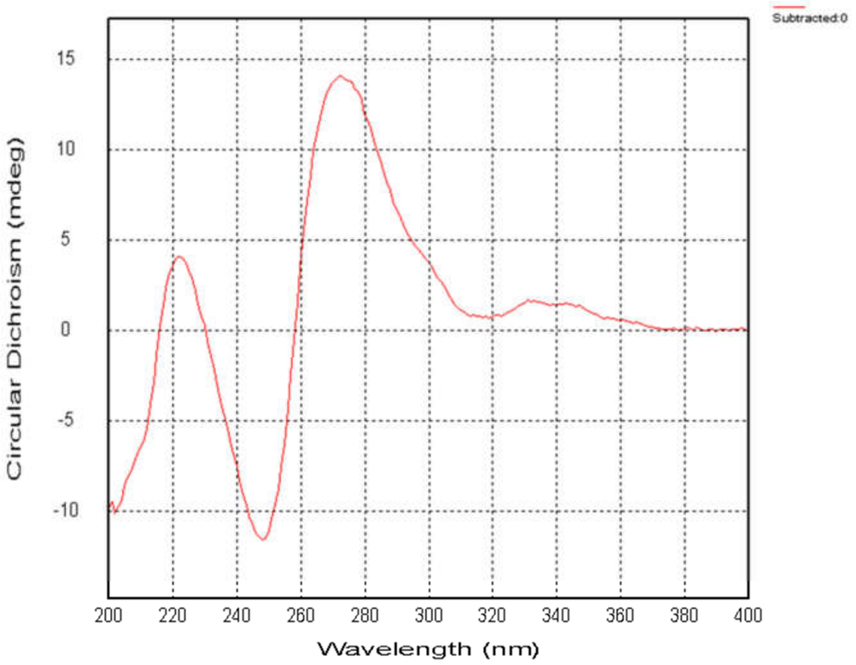

Figure S39: CD spectrum of compound 4

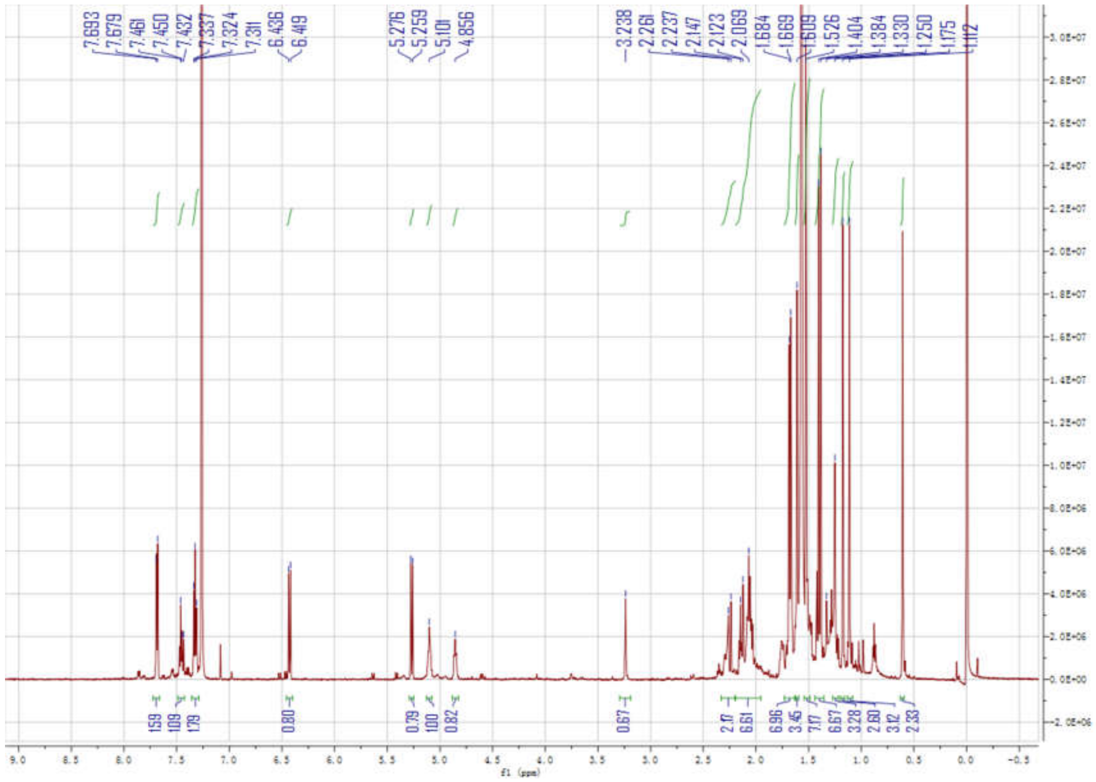

Figure S40: ^{1}H-NMR (600 MHz, $CDCl_3$) spectrum of compound 5

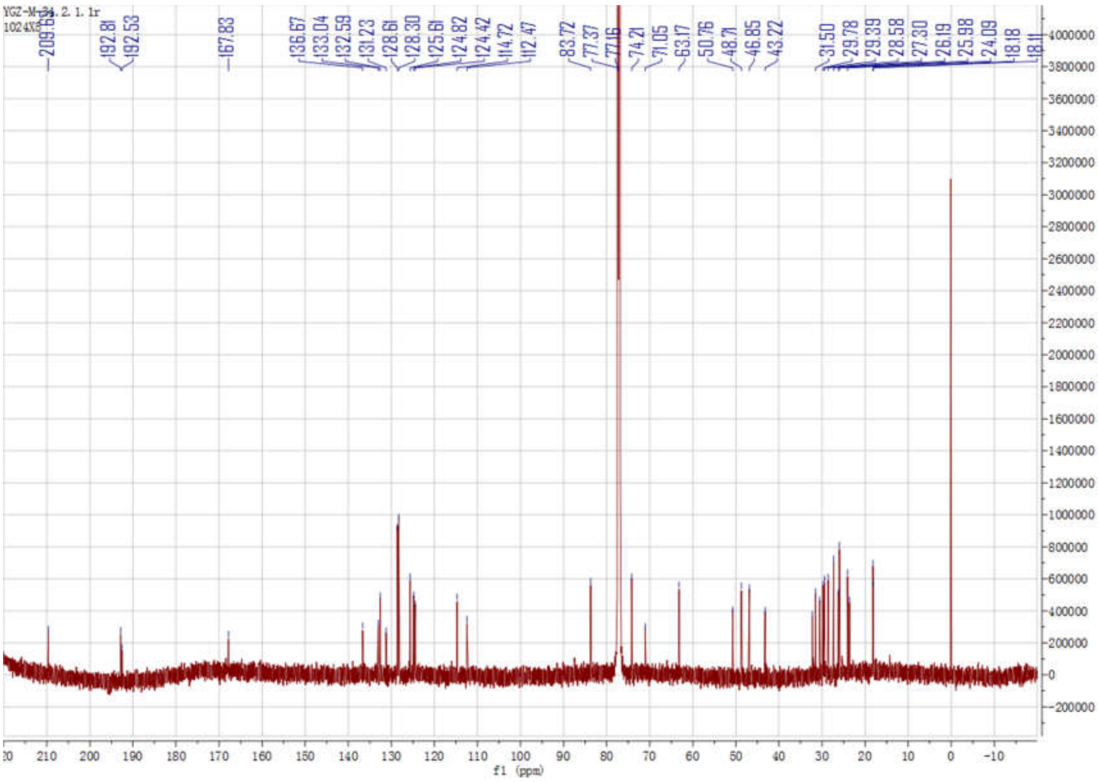

Figure S41: ^{13}C-NMR (150 MHz, $CDCl_3$) spectrum of compound 5

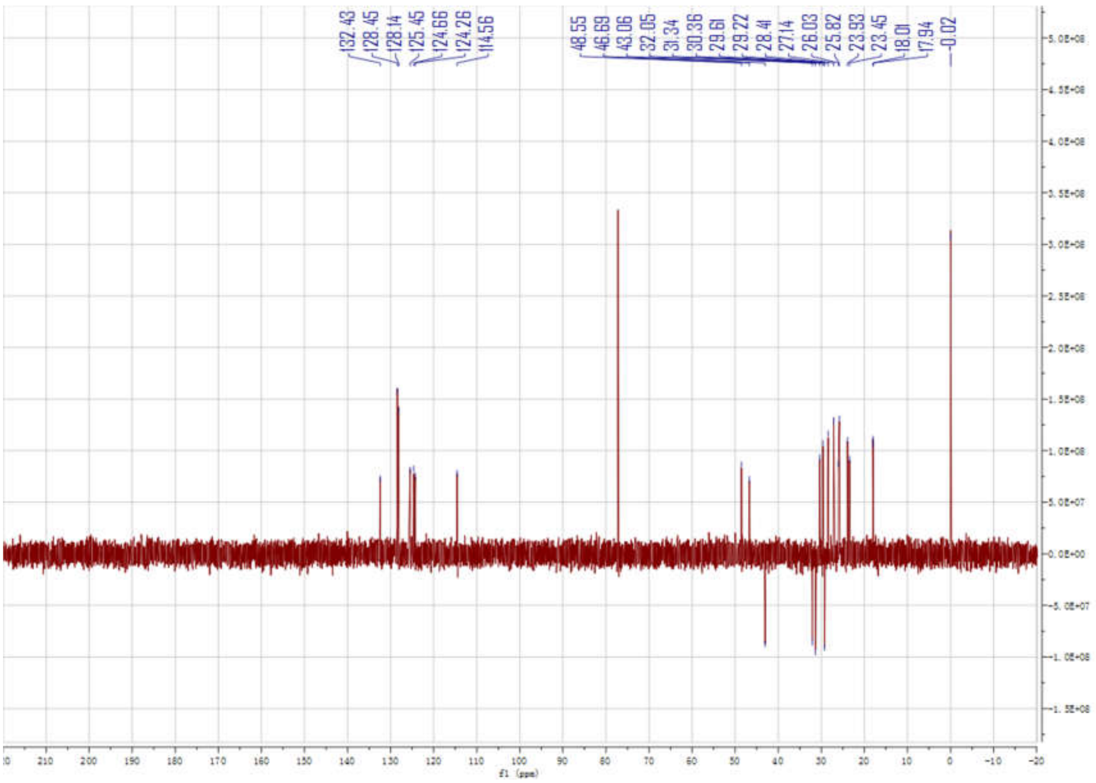

Figure S42: ^{13}C-NMR-DEPT (θ=135°) spectrum of compound 5

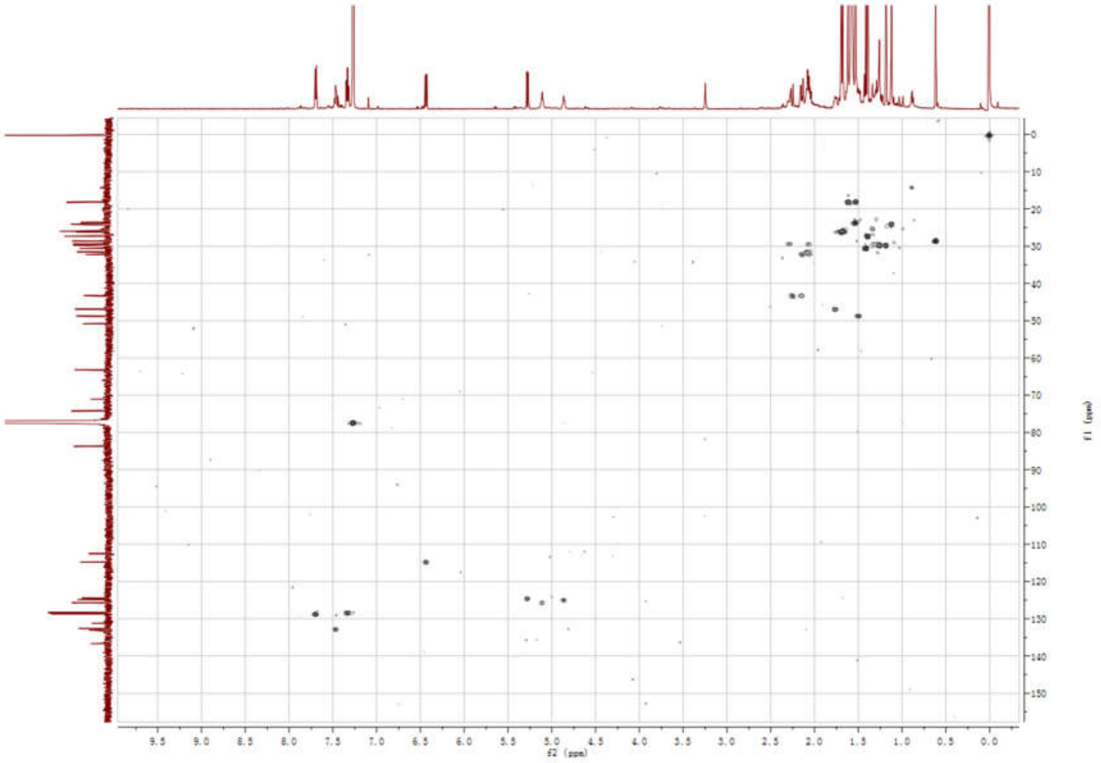

Figure S43: HSQC spectrum of compound 5

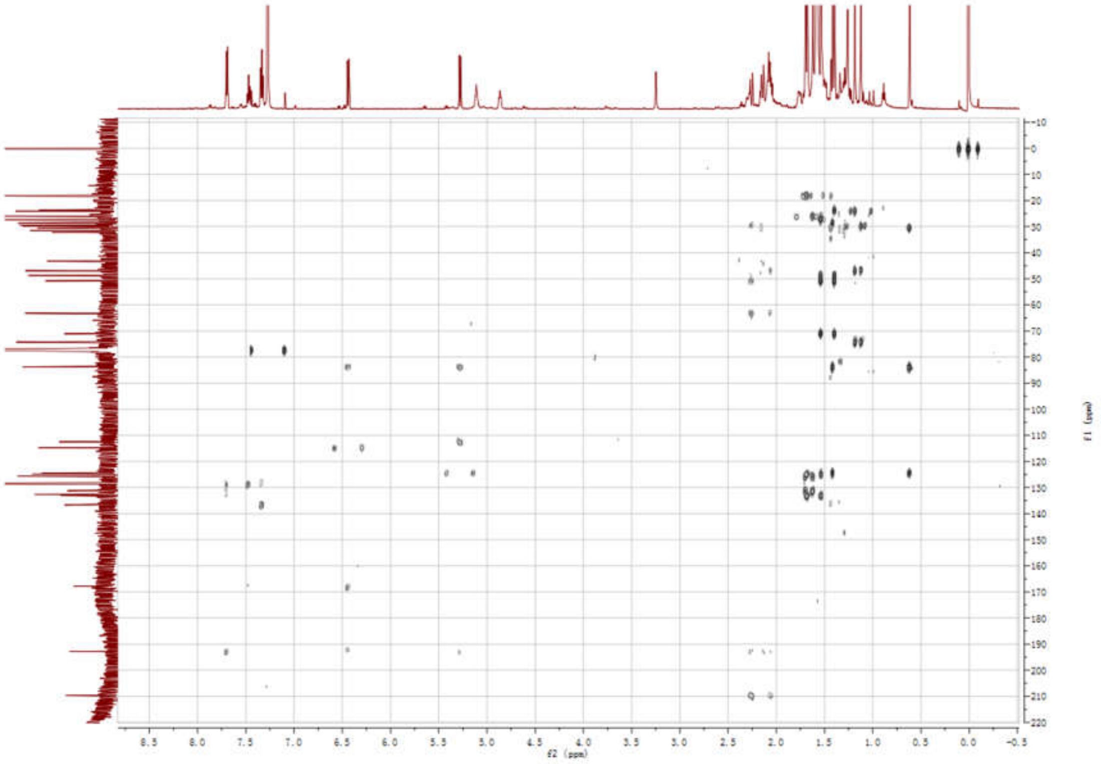

Figure S44: HMBC spectrum of compound 5

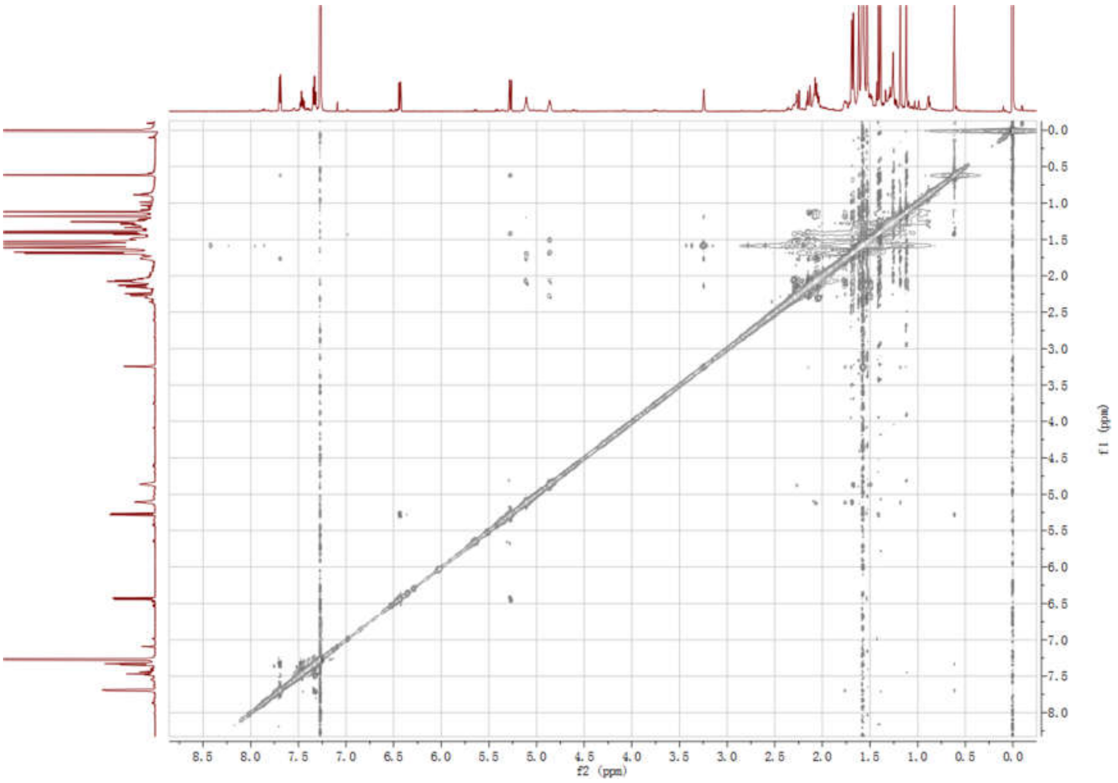

Figure S45: ROESY spectrum of compound 5

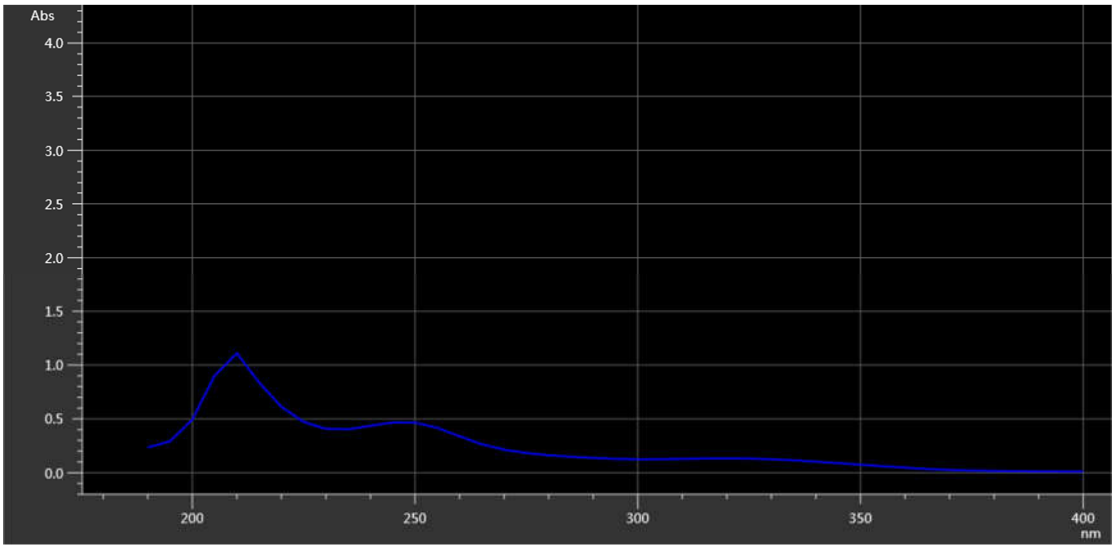

Figure S46: UV spectrum of compound 5

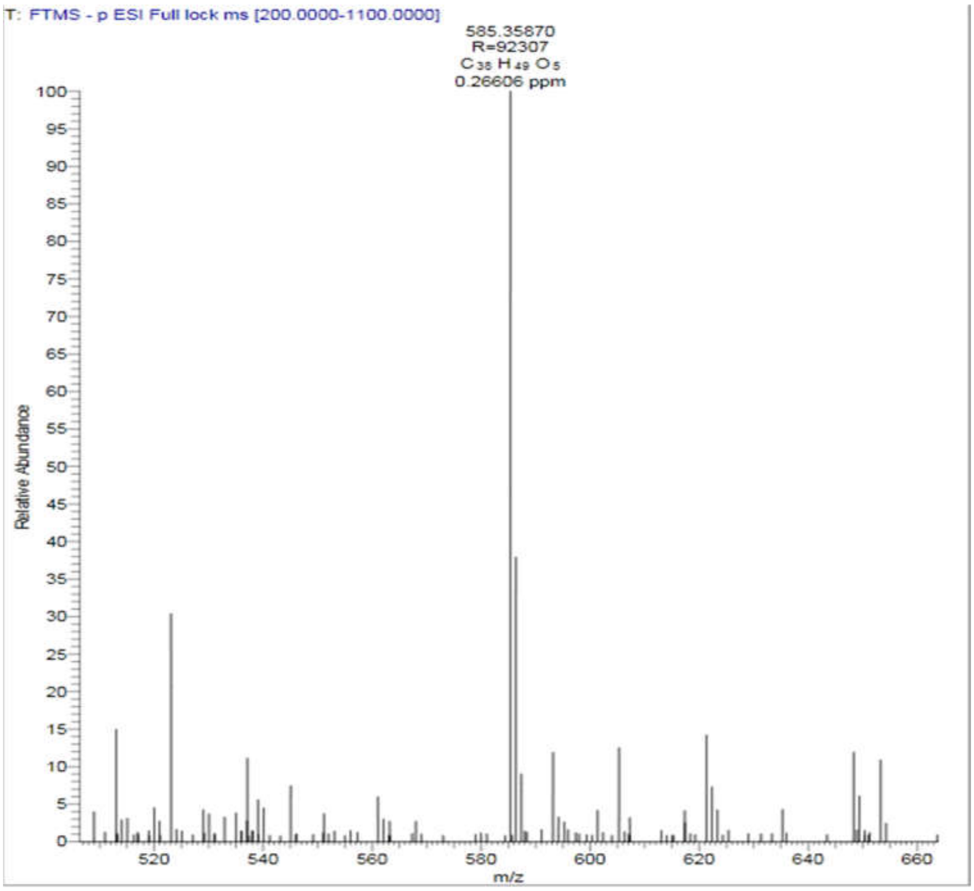

Figure S47: HR-ESI-MS of compound 5

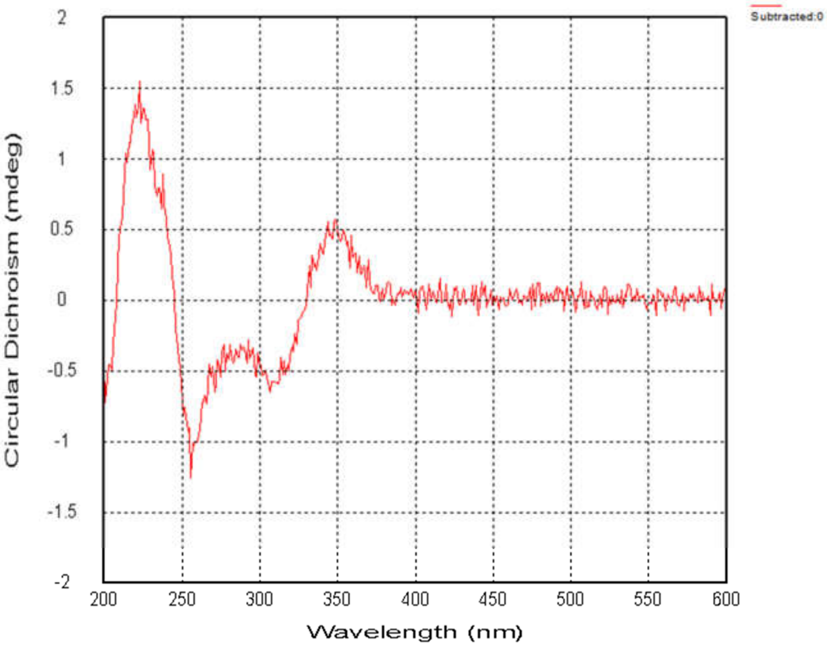

Figure S48: CD spectrum of compound 5

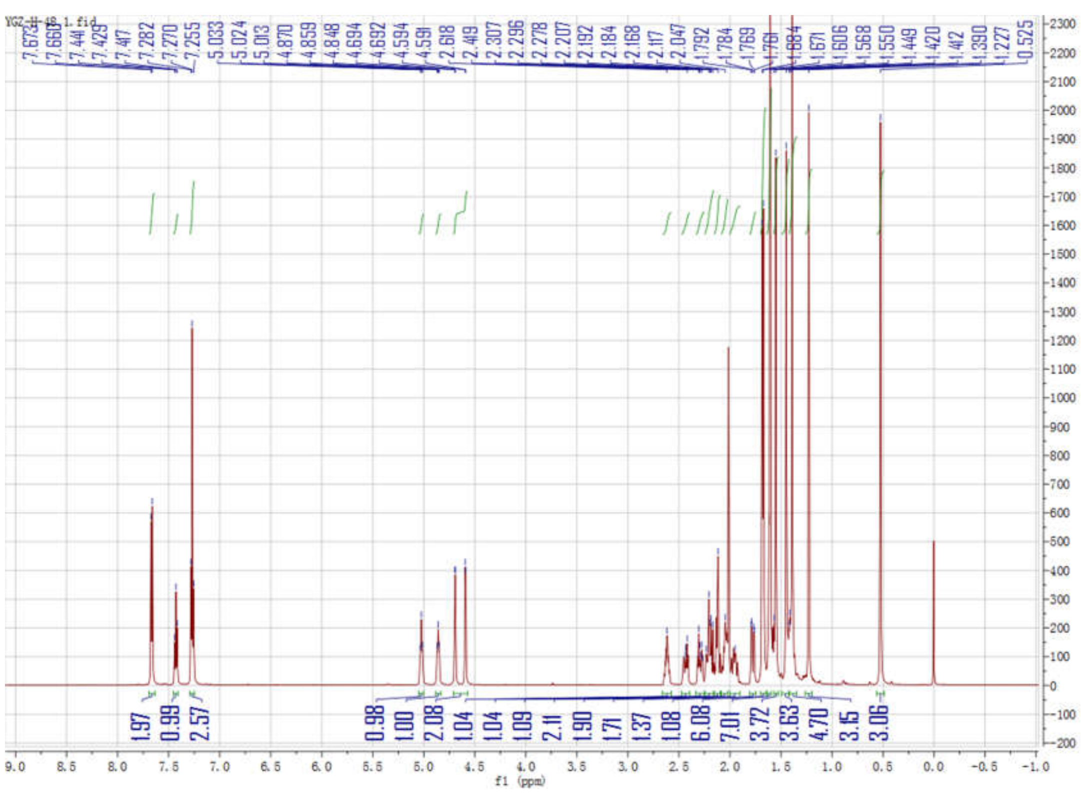

Figure S49: ^{1}H-NMR (600 MHz, $CDCl_3$) spectrum of compound 6

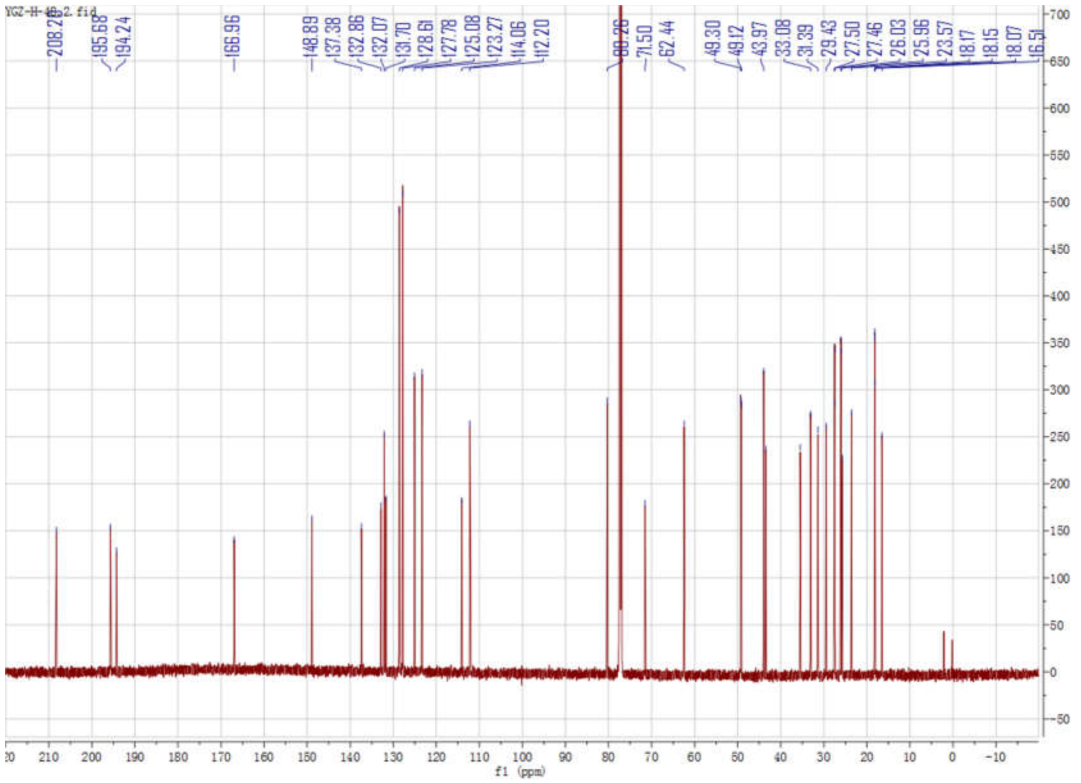

Figure S50: ^{13}C-NMR (150 MHz, $CDCl_3$) spectrum of compound 6

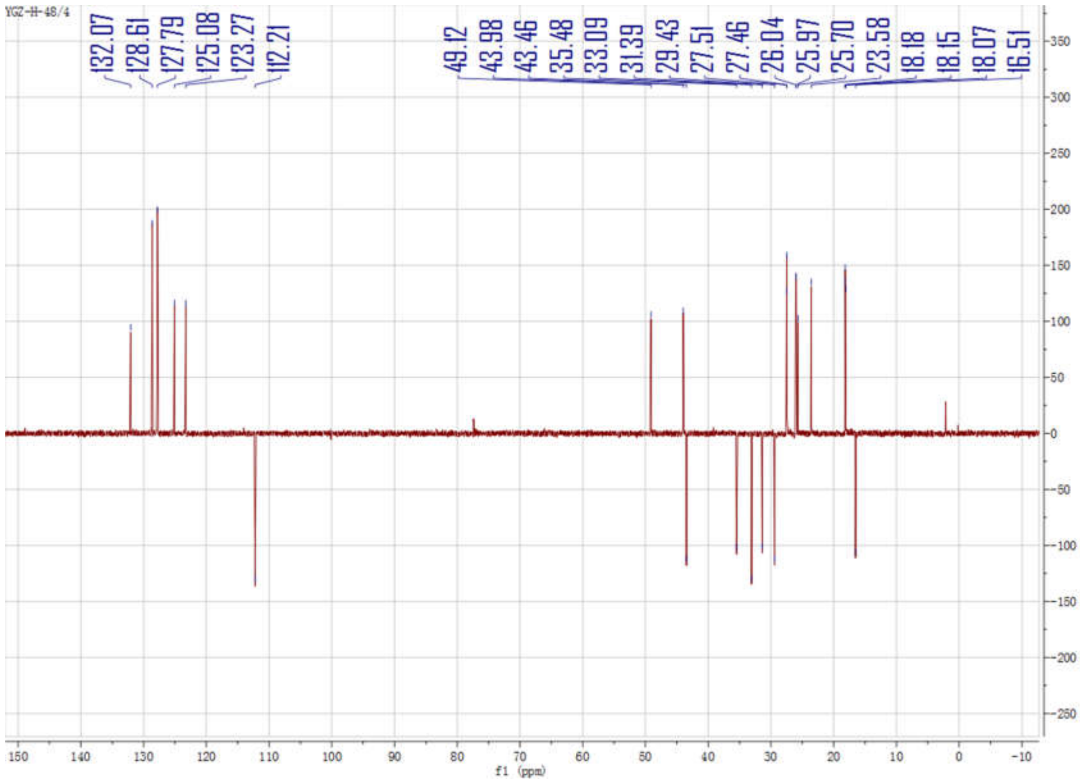

Figure S51: ^{13}C-NMR-DEPT (θ=135°) spectrum of compound 6

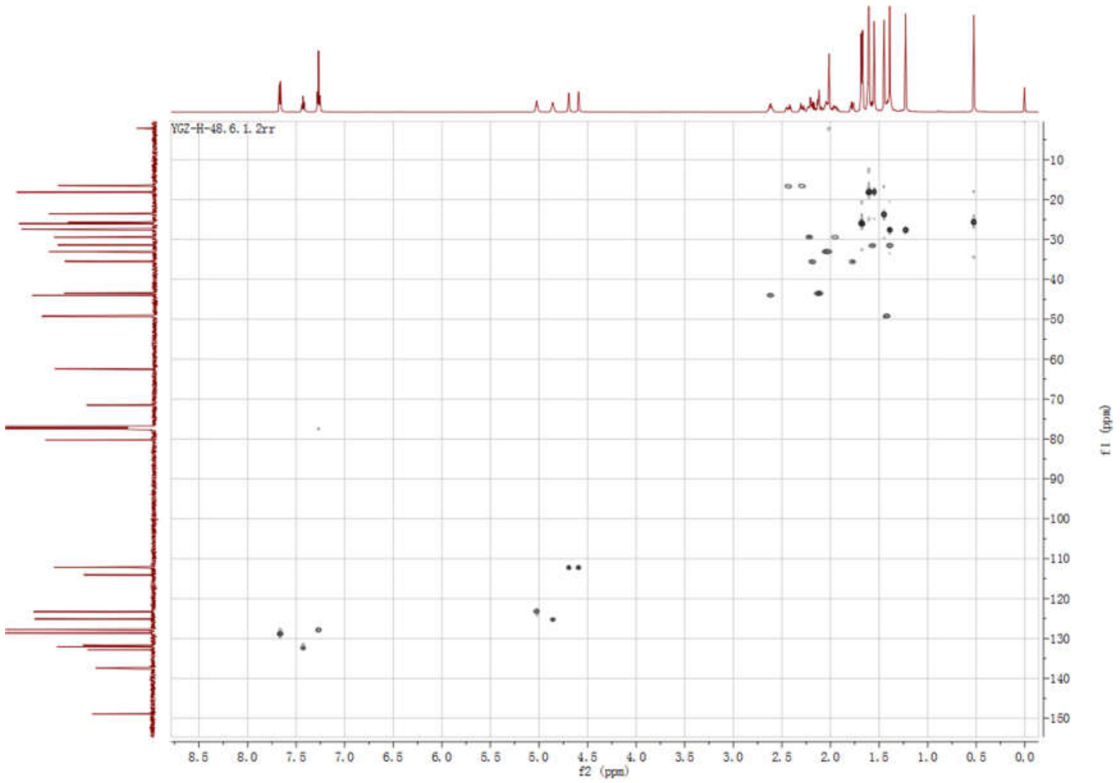

Figure S52: HSQC spectrum of compound 6

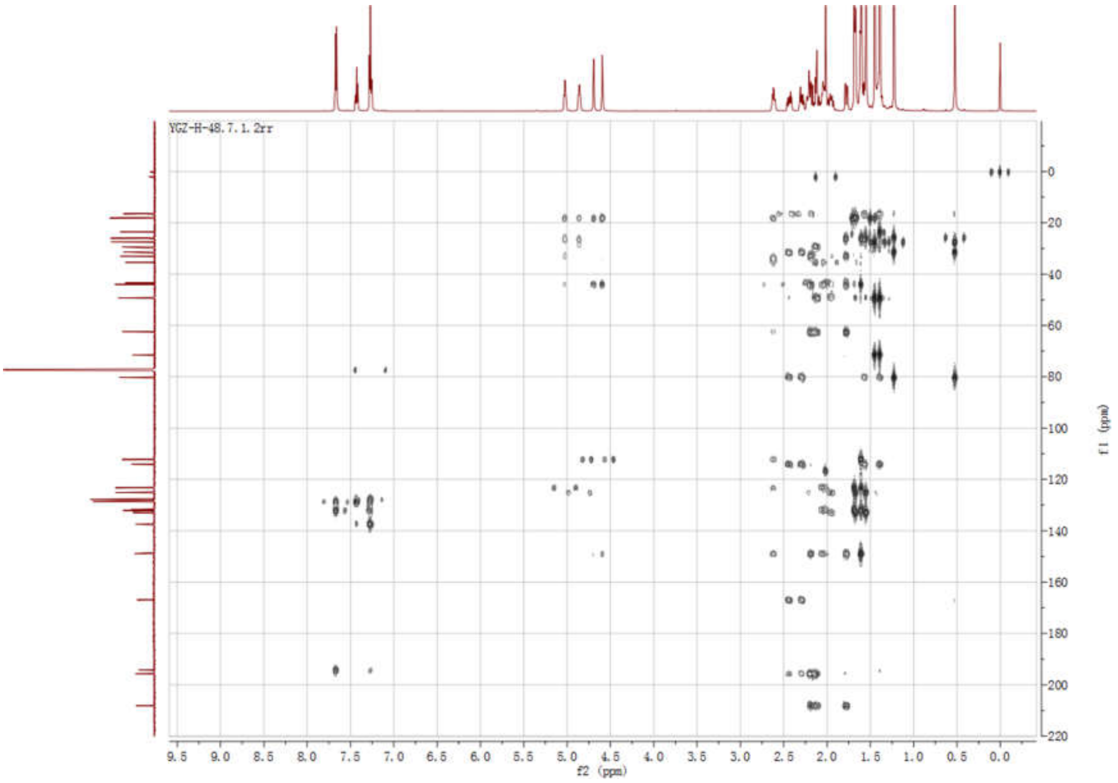

Figure S53: HMBC spectrum of compound 6

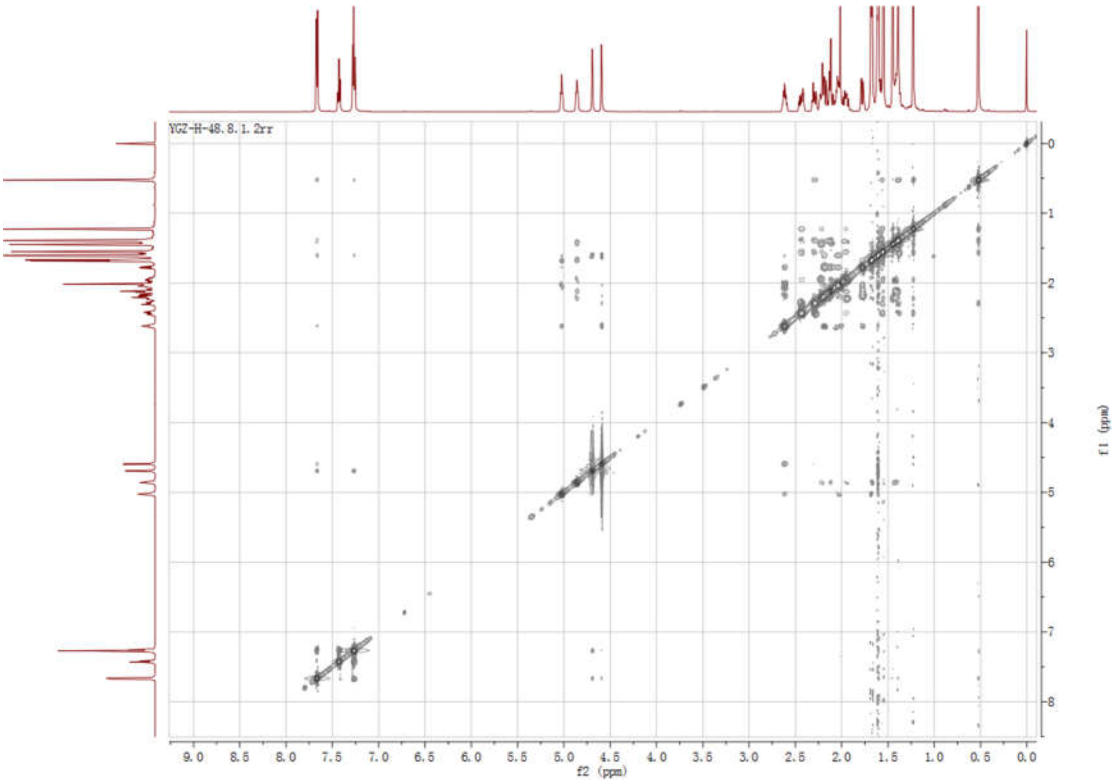

Figure S54: ROESY spectrum of compound 6

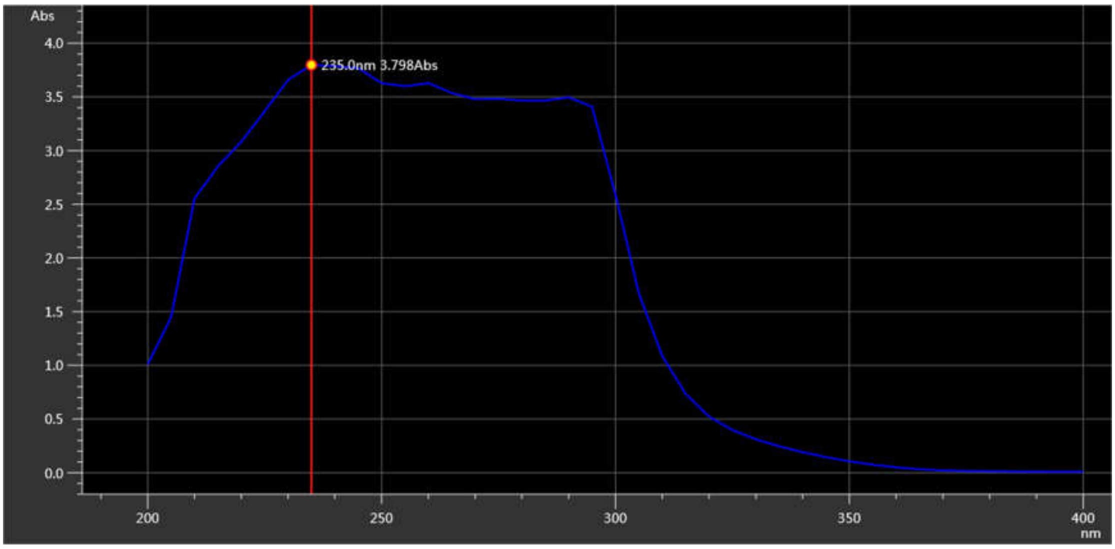

Figure S55: UV spectrum of compound 6

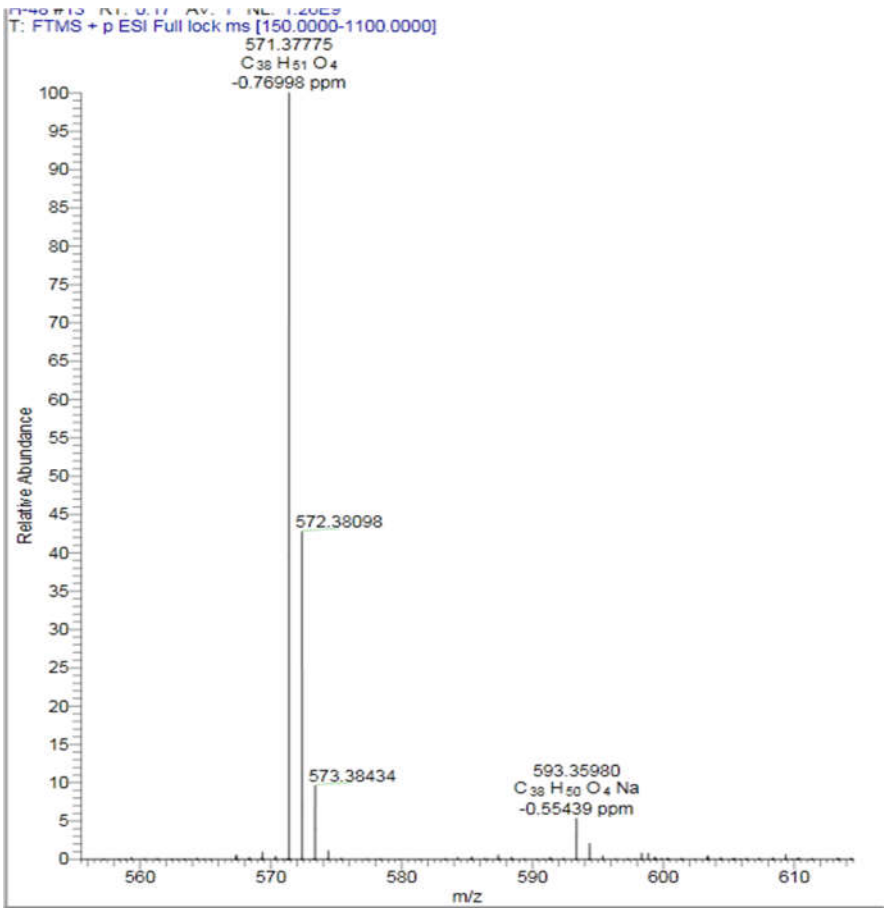

Figure S56: HR-ESI-MS of compound 6

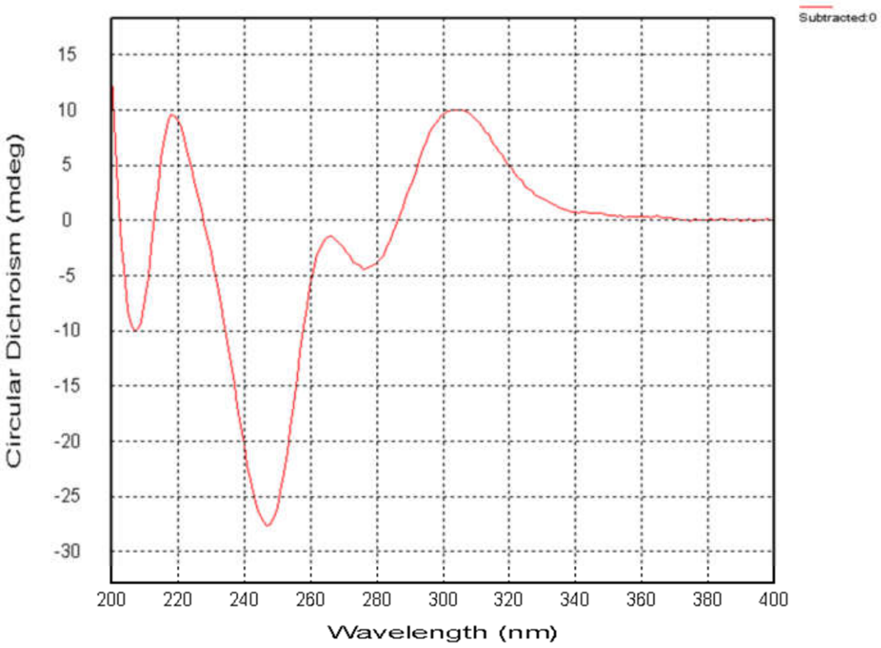

Figure S57: CD spectrum of compound 6

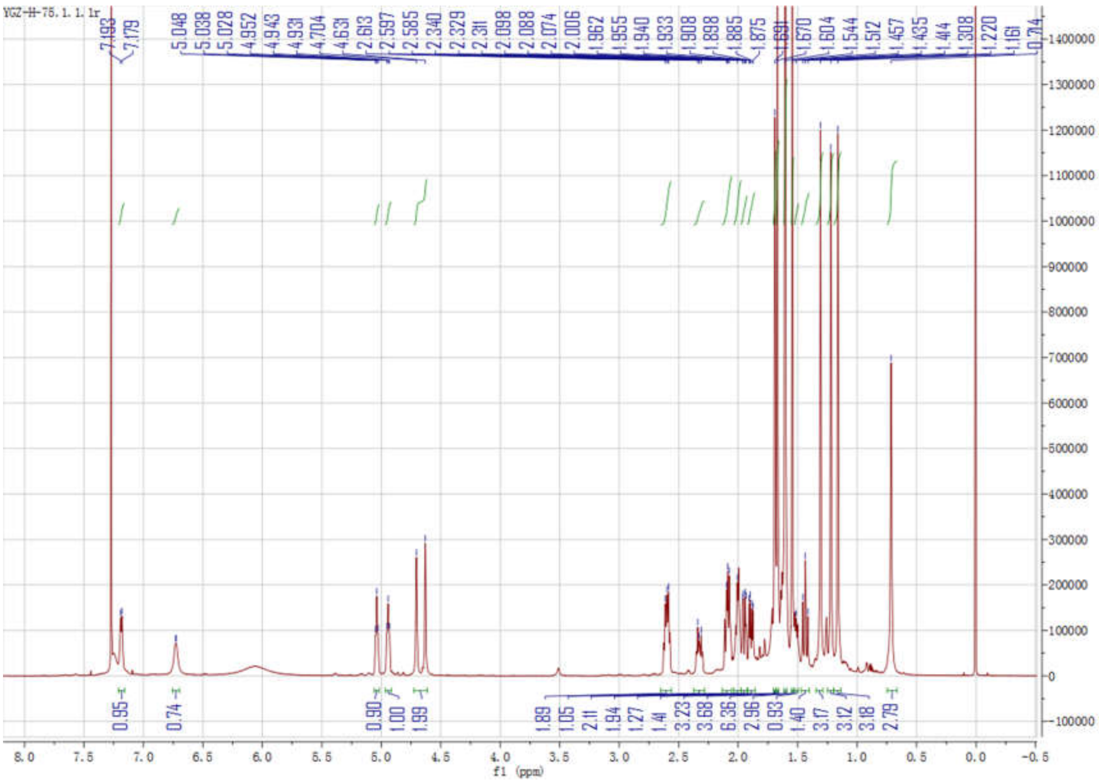

Figure S58: ^{1}H-NMR (600 MHz, $CDCl_3$) spectrum of compound 7

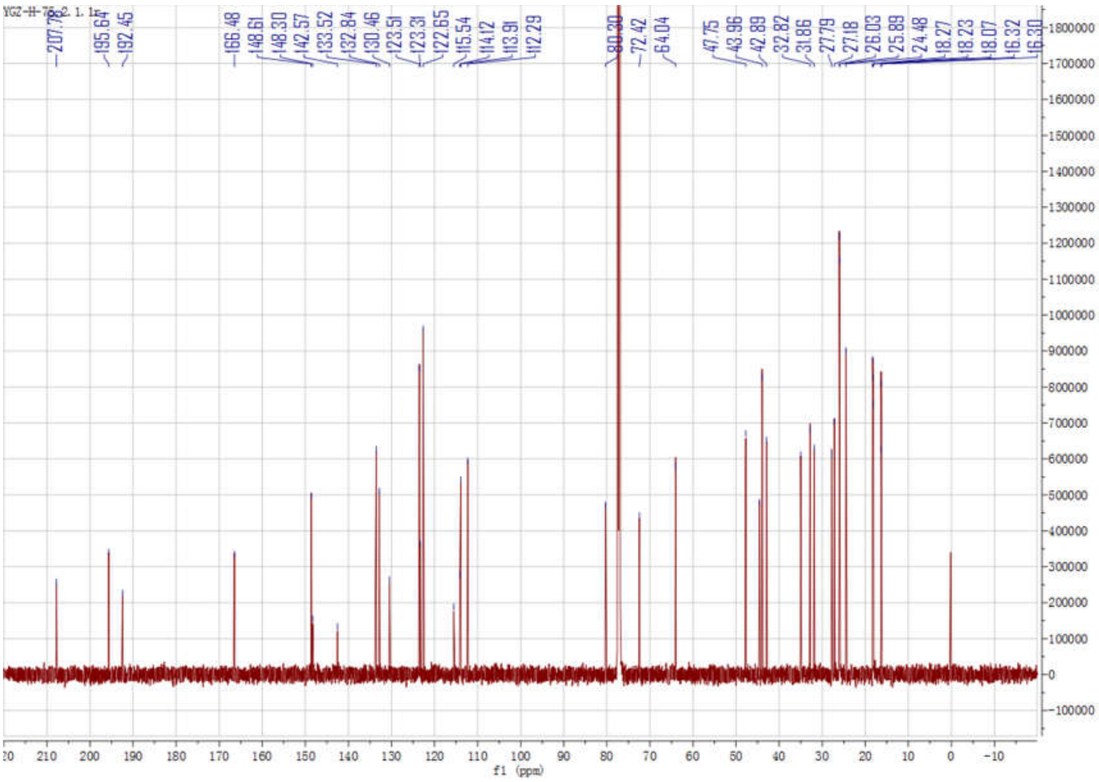

Figure S59: The ^{13}C-NMR (150 MHz, $CDCl_3$) spectrum of compound 7

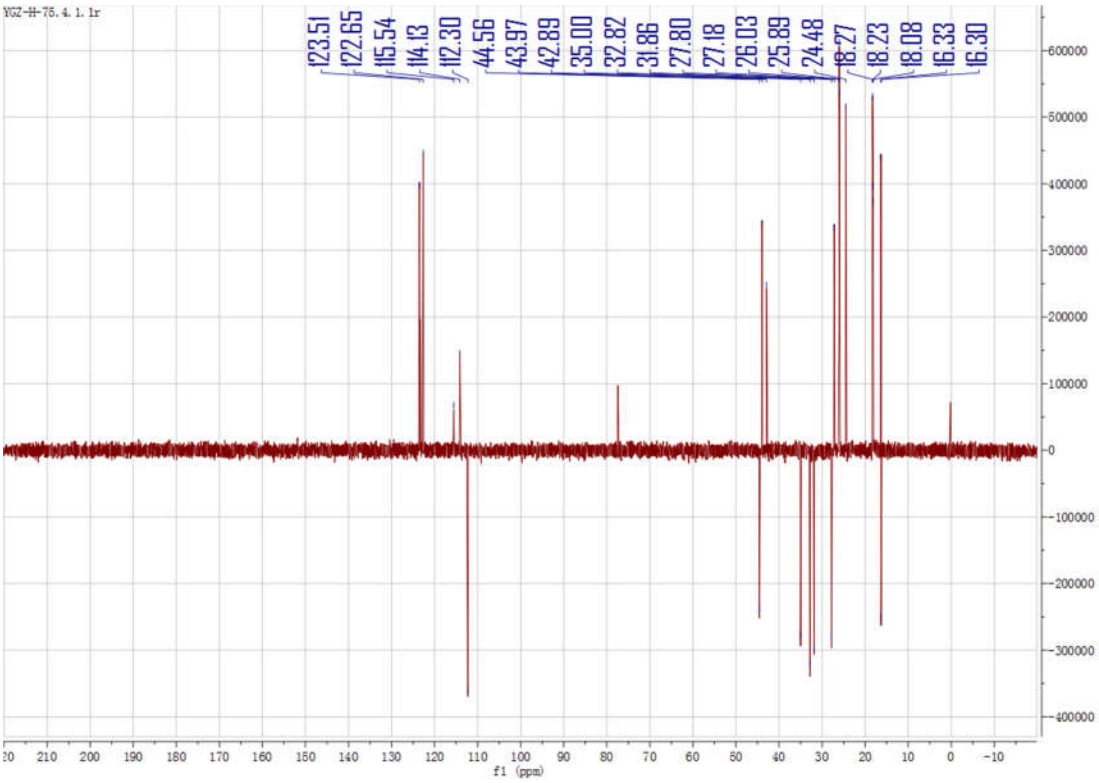

Figure S60: ^{13}C-NMR-DEPT (θ=135°) spectrum of compound 7

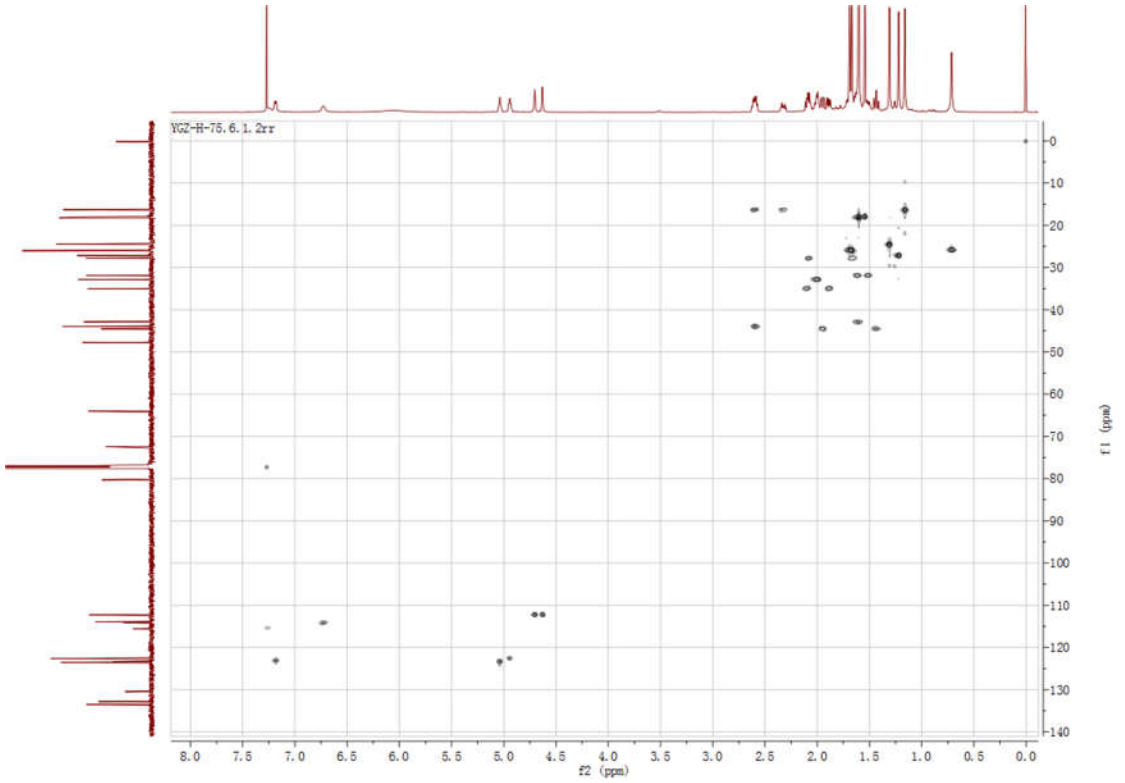

Figure S61: HSQC spectrum of compound 7

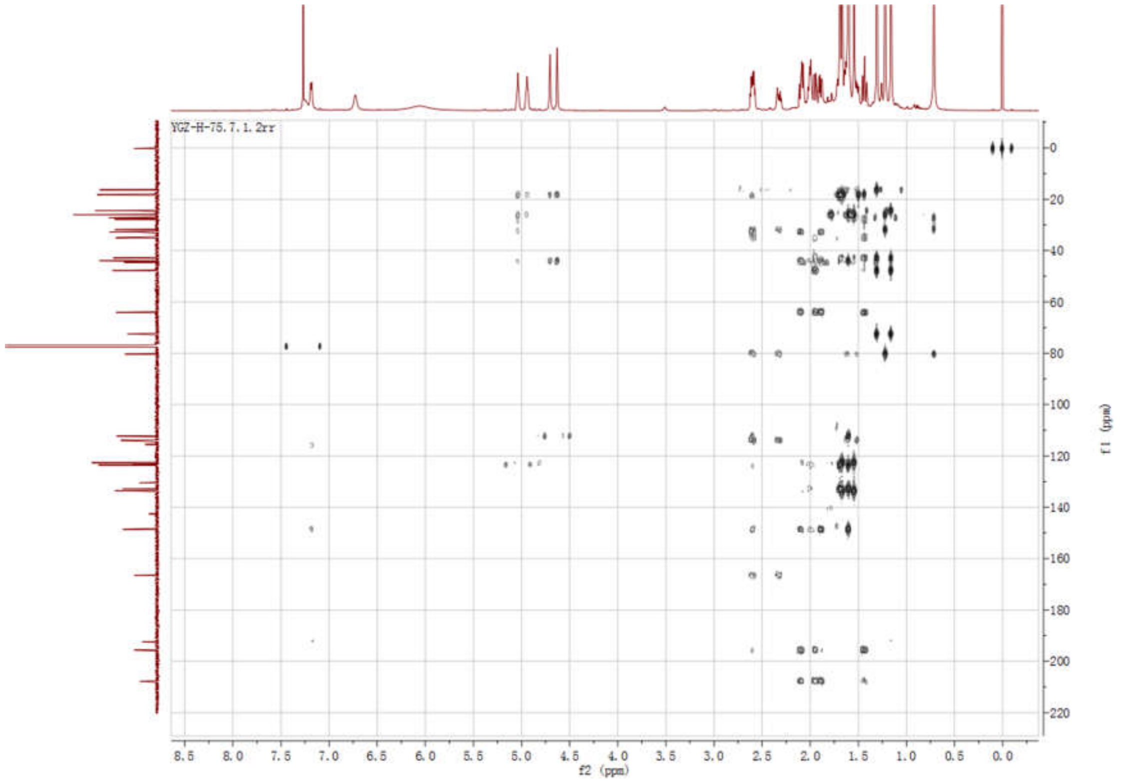

Figure S62: HMBC spectrum of compound 7

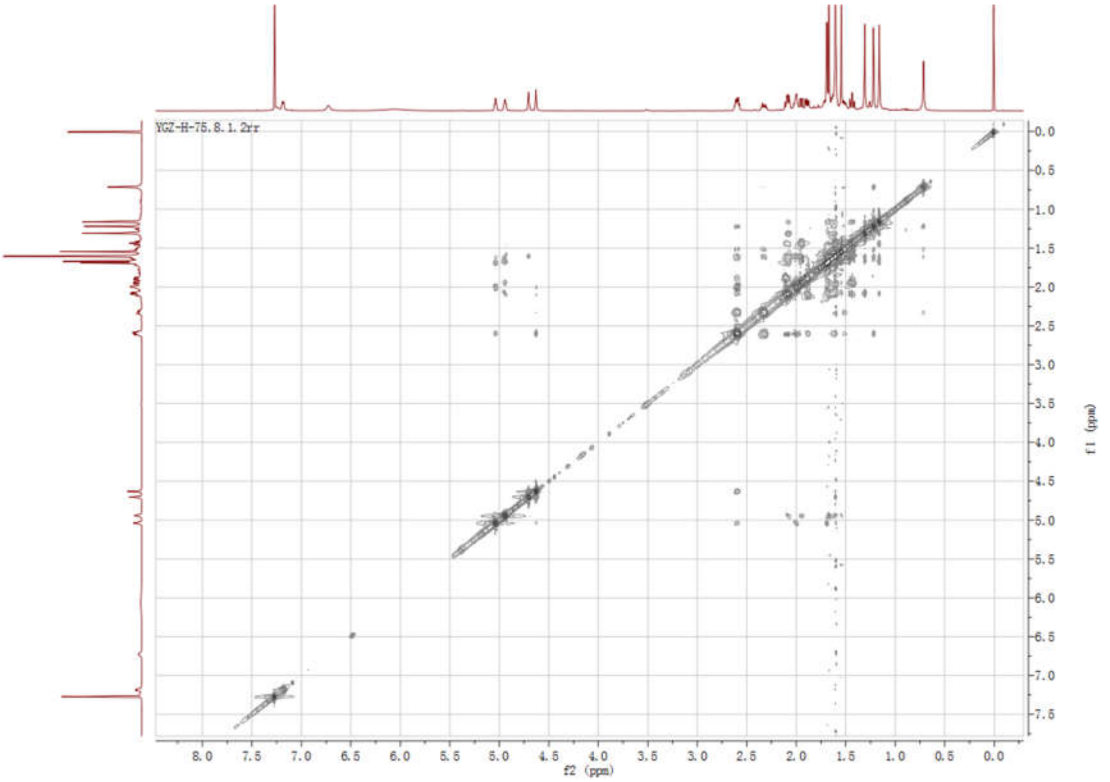

Figure S63: ROESY spectrum of compound 7

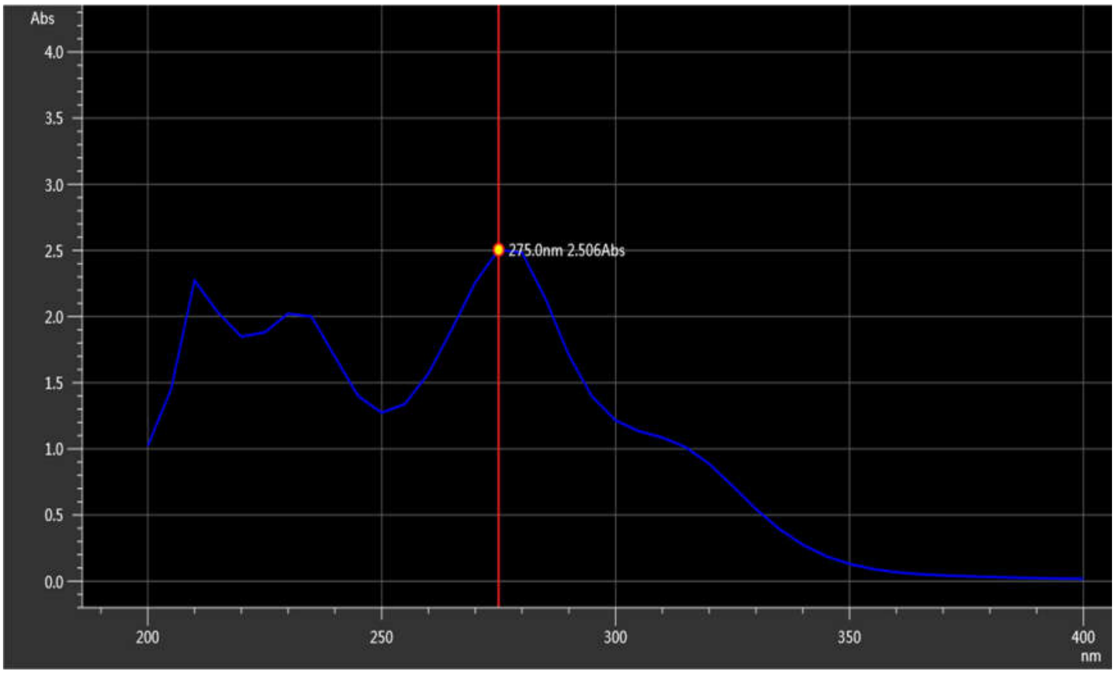

Figure S64: UV spectrum of compound 7

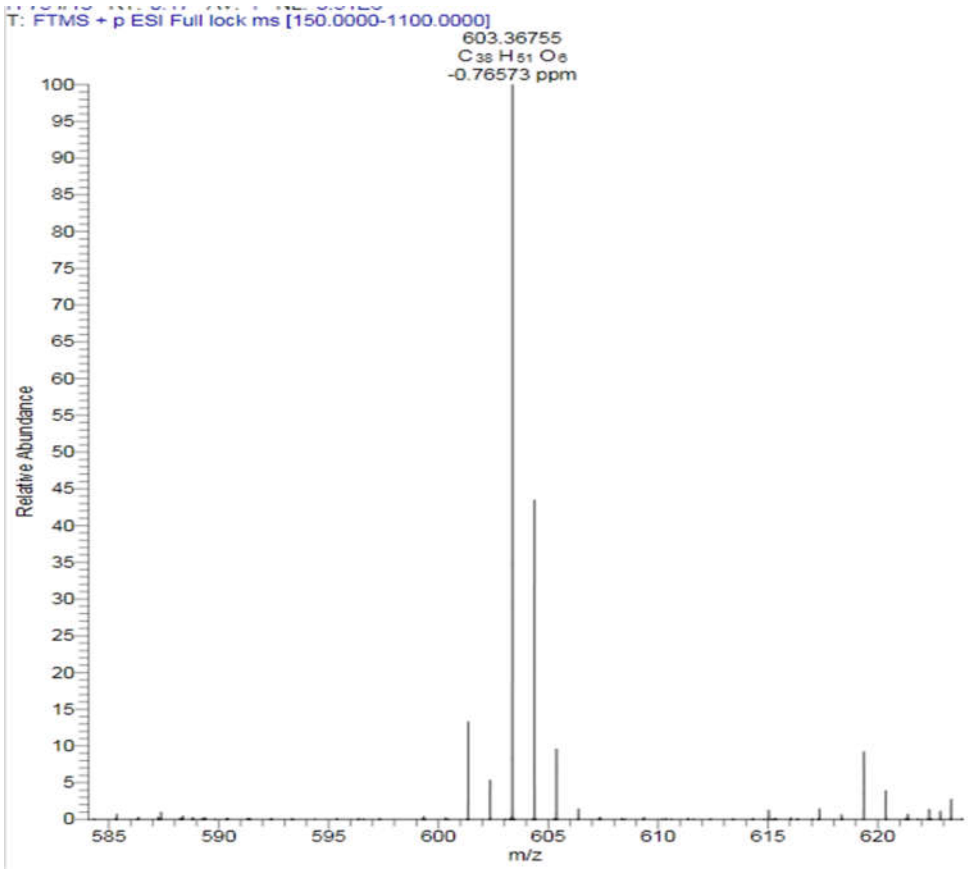

Figure S65: HR-ESI-MS of compound 7

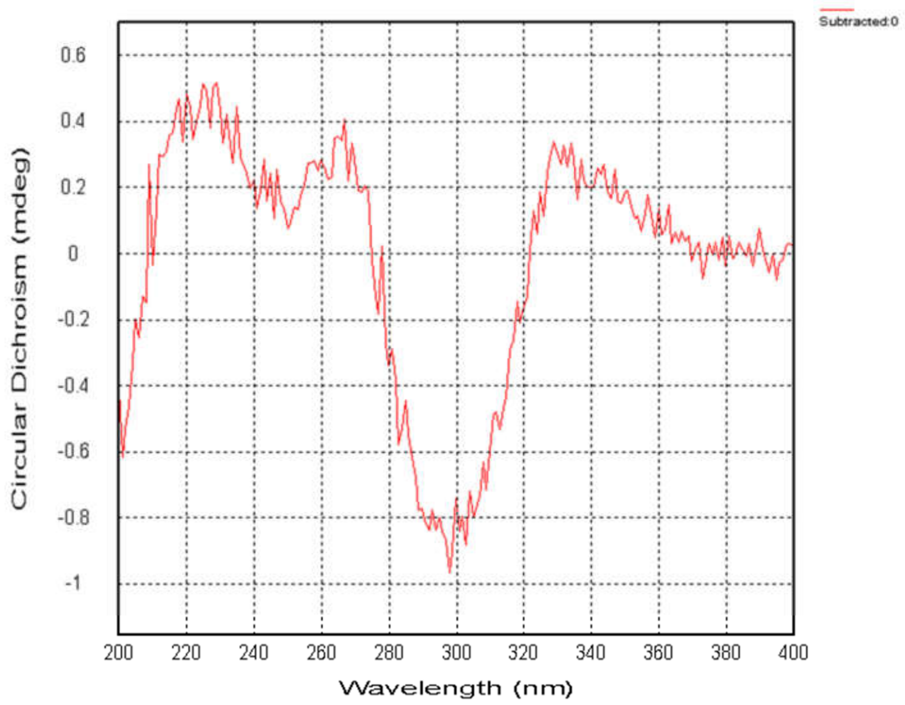

Figure S66: CD spectrum of compound 7

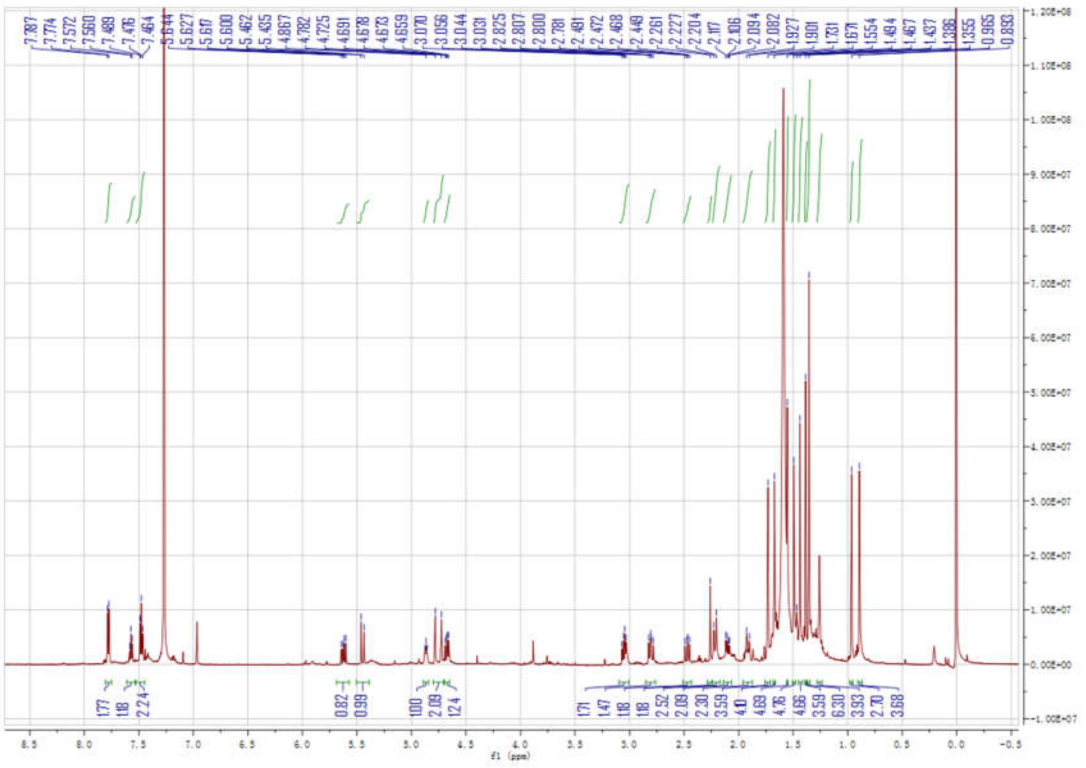

Figure S67: ^{1}H-NMR (600 MHz, $CDCl_3$) spectrum of compound 8

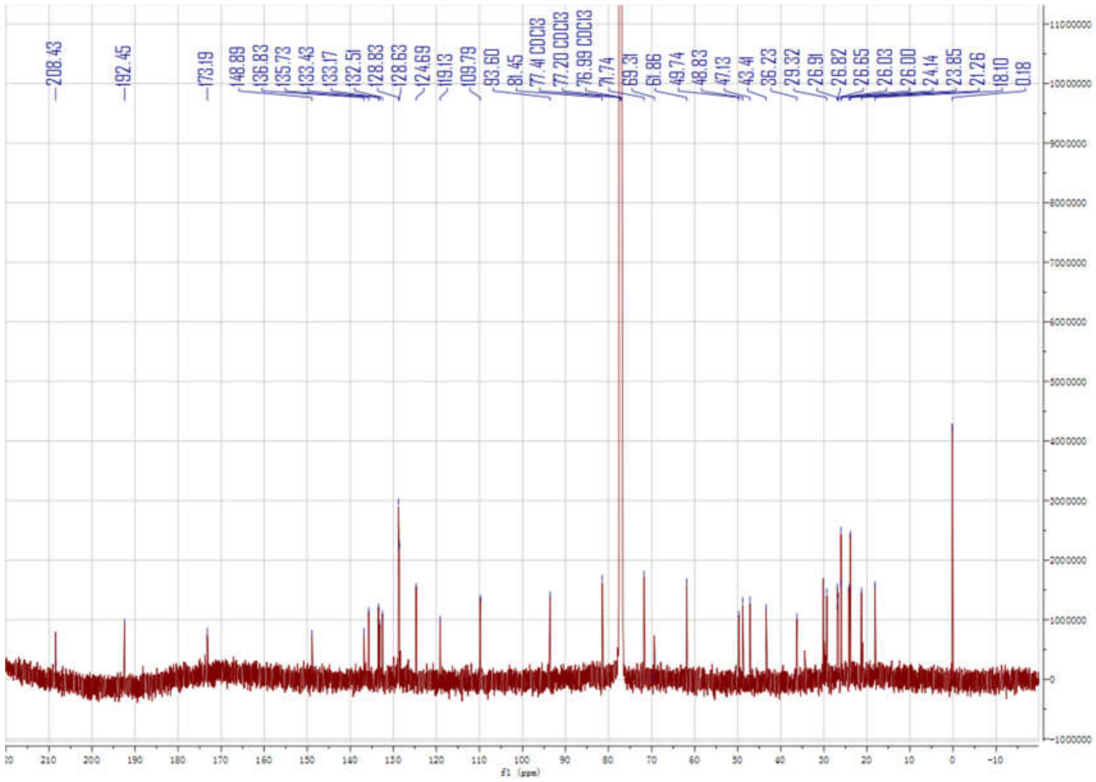

Figure S68: ^{13}C-NMR (150 MHz, $CDCl_3$) spectrum of compound 8

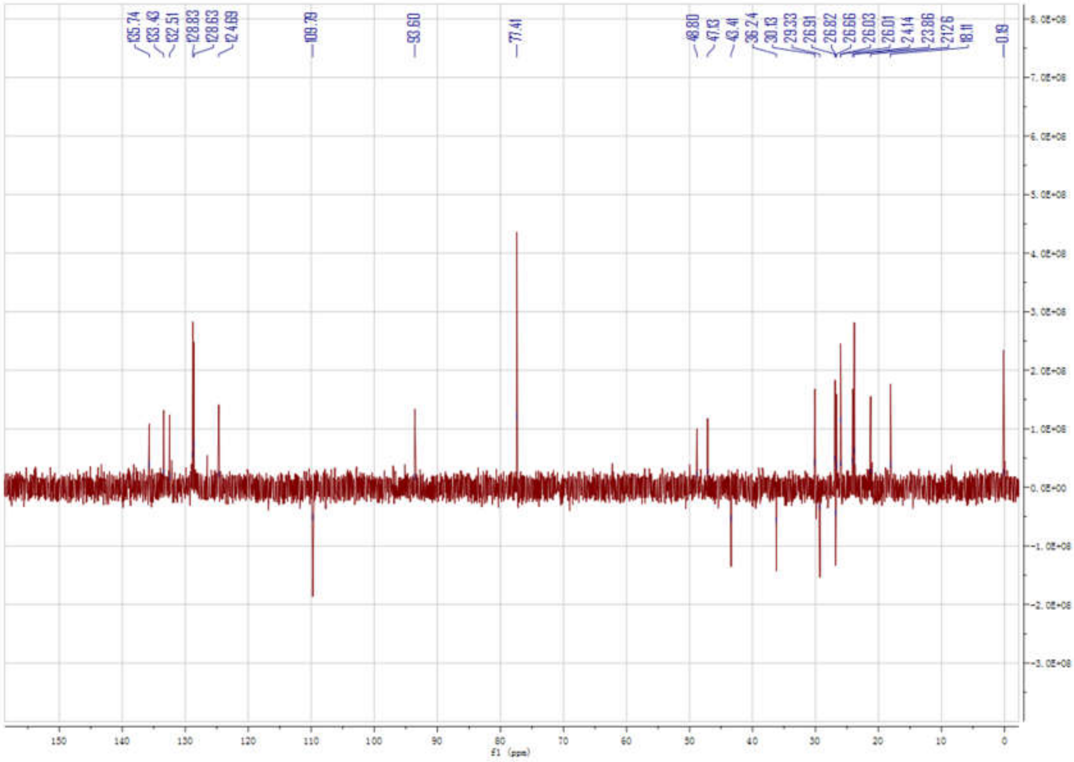

Figure S69: ^{13}C-NMR-DEPT (θ=135°) spectrum of compound 8

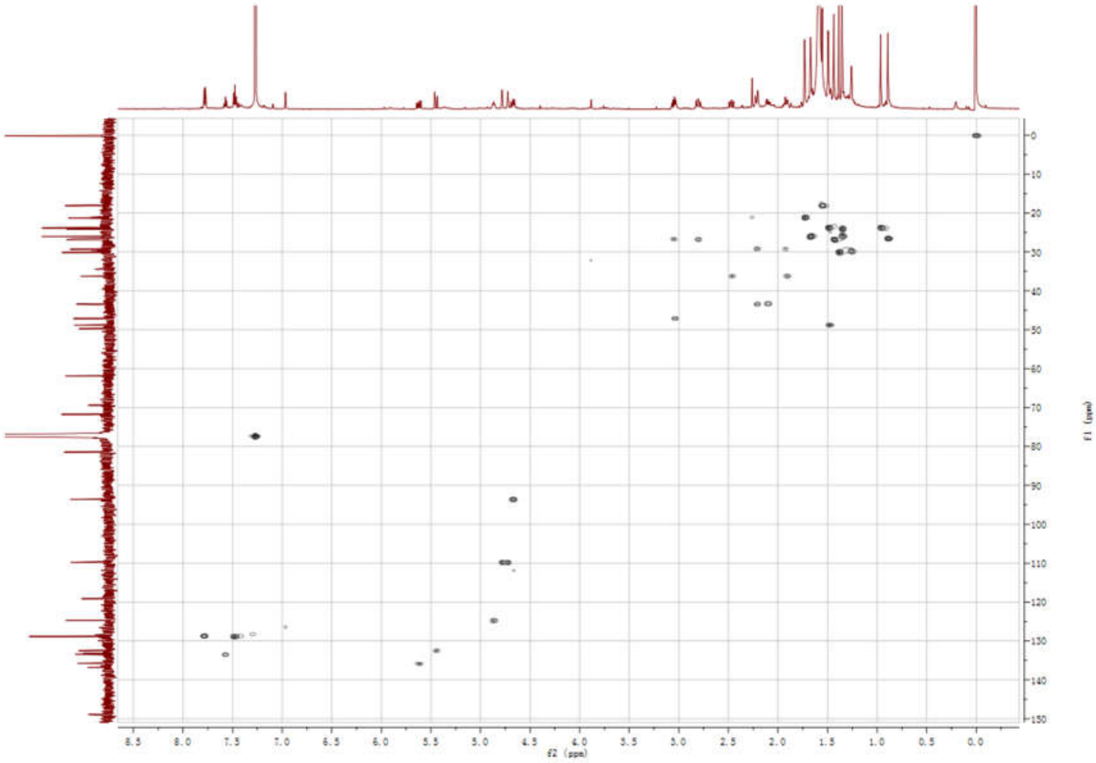

Figure S70: HSQC spectrum of compound 8

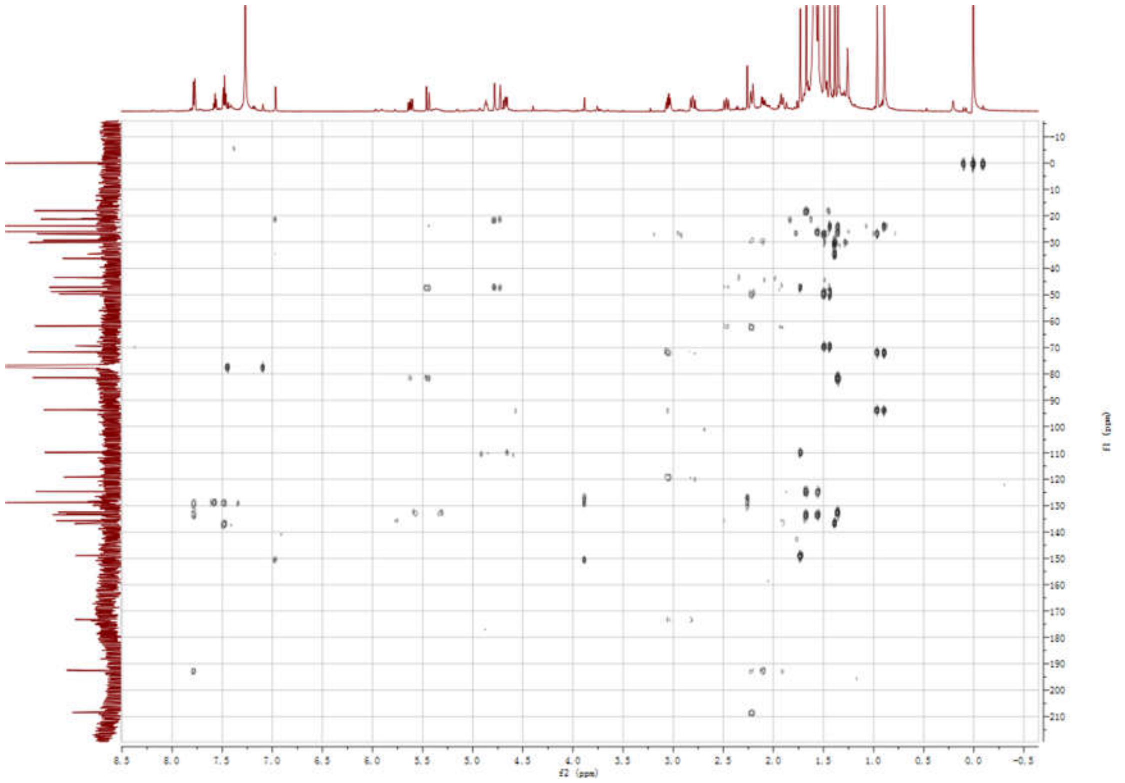

Figure S71: HMBC spectrum of compound 8

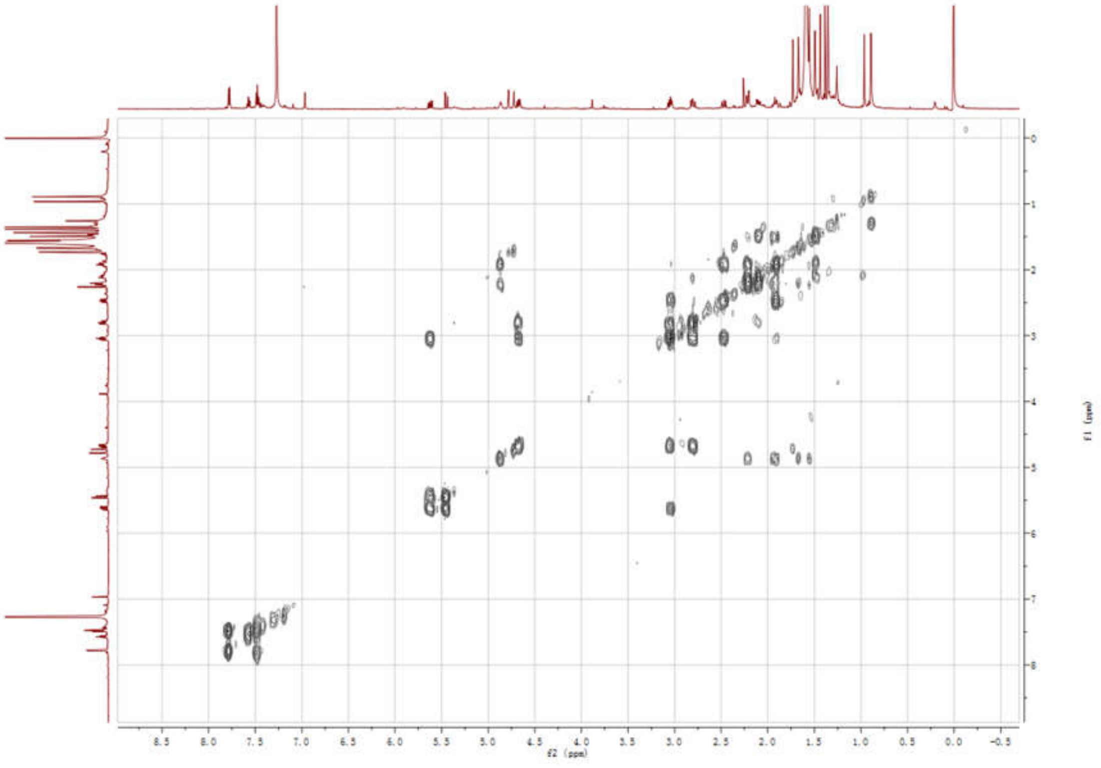

Figure S72: ^1H-^1H COSY spectrum of compound 8

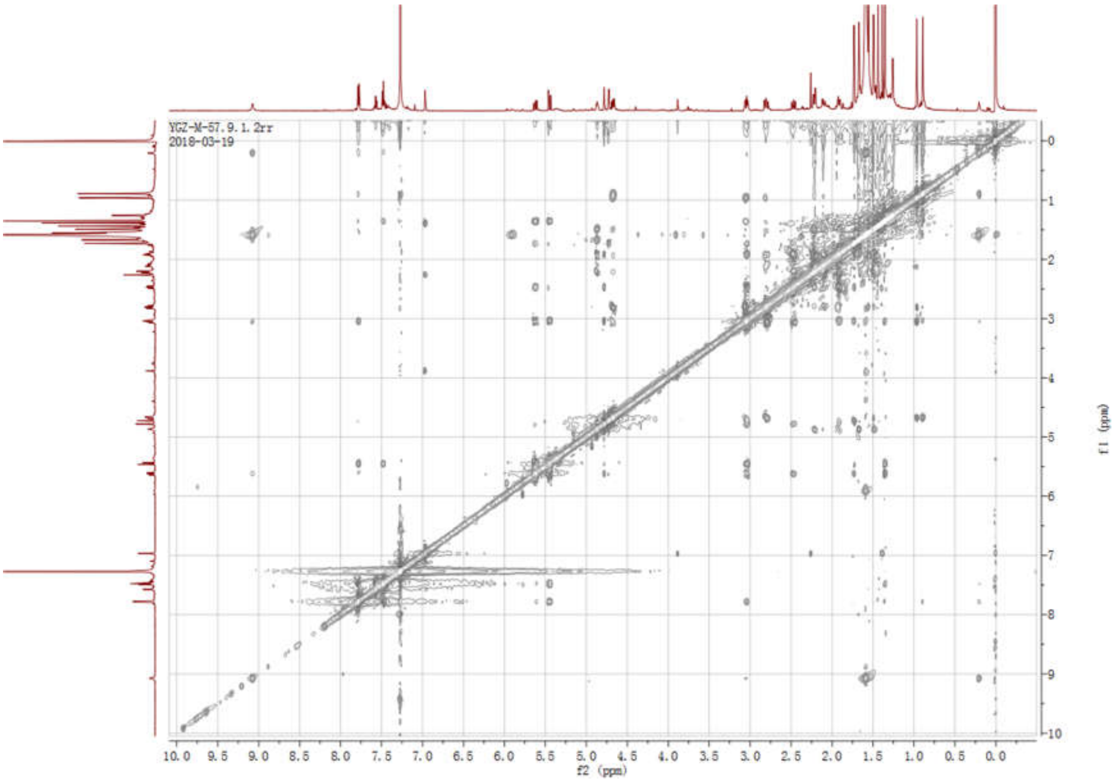

Figure S73: ROESY spectrum of compound 8

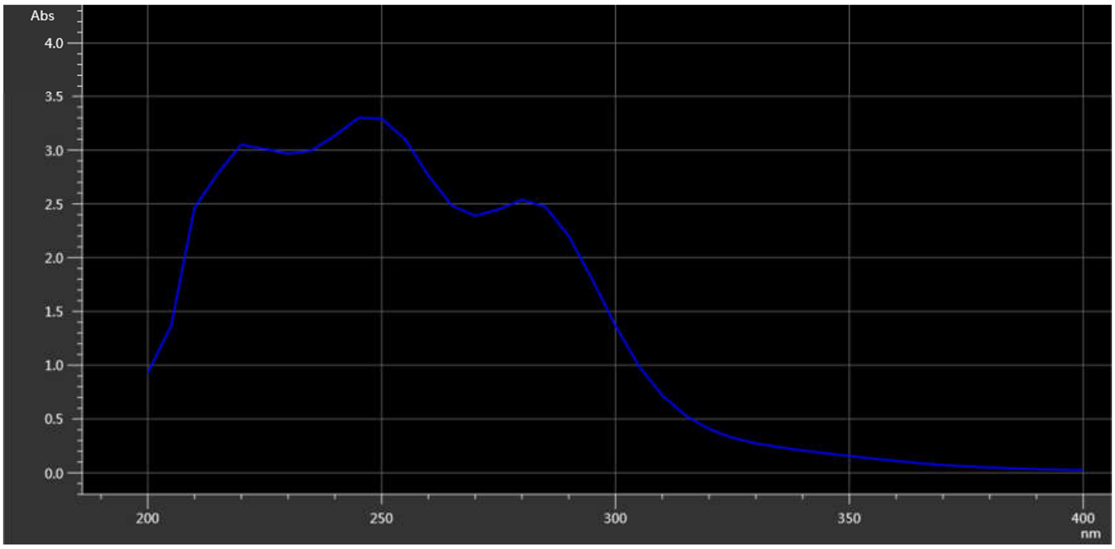

Figure S74: UV spectrum of compound 8

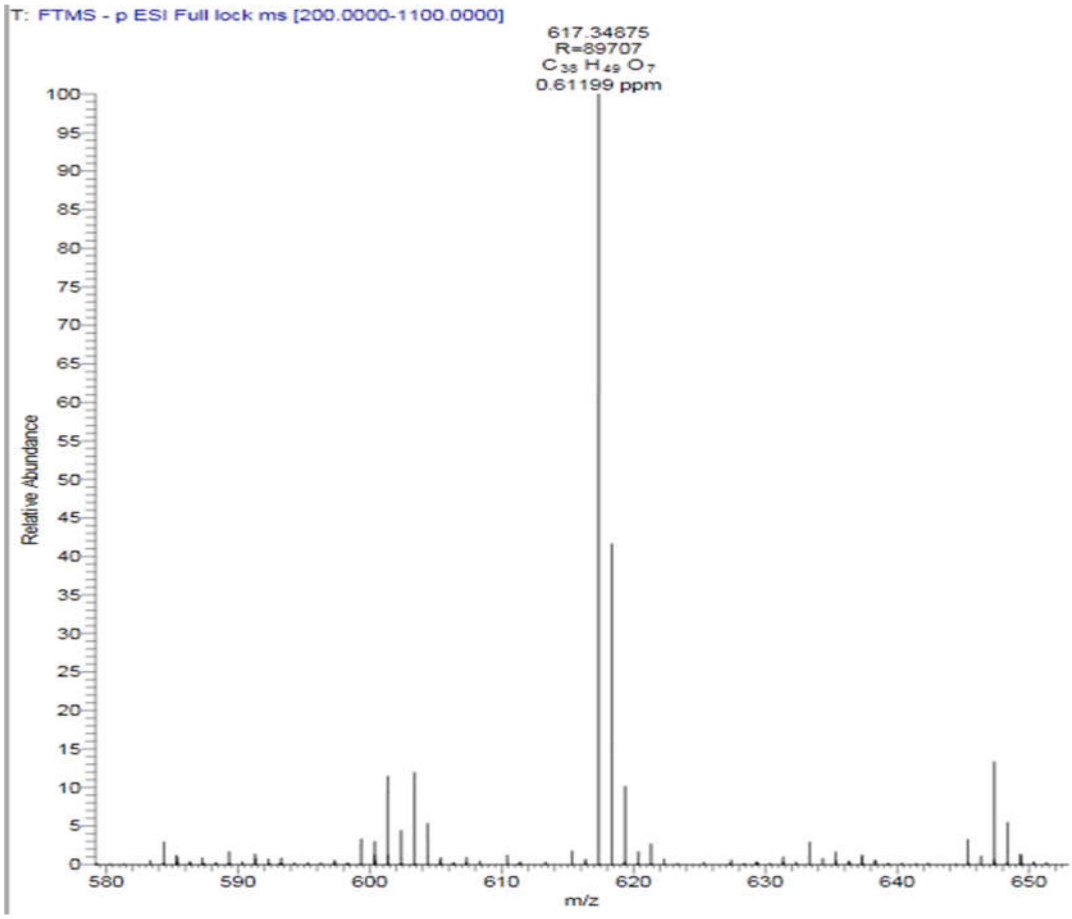

Figure S75: HR-ESI-MS of compound 8

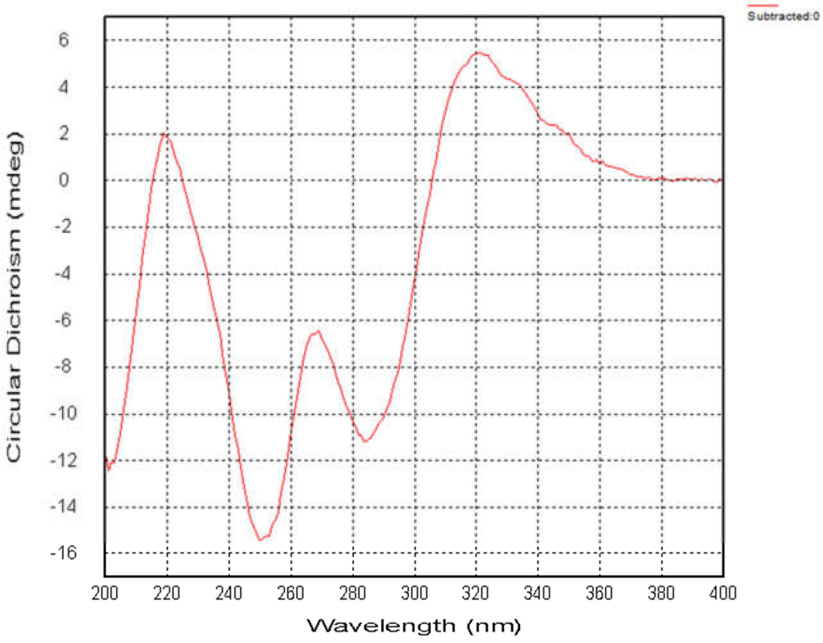

Figure S76: CD spectrum of compound 8

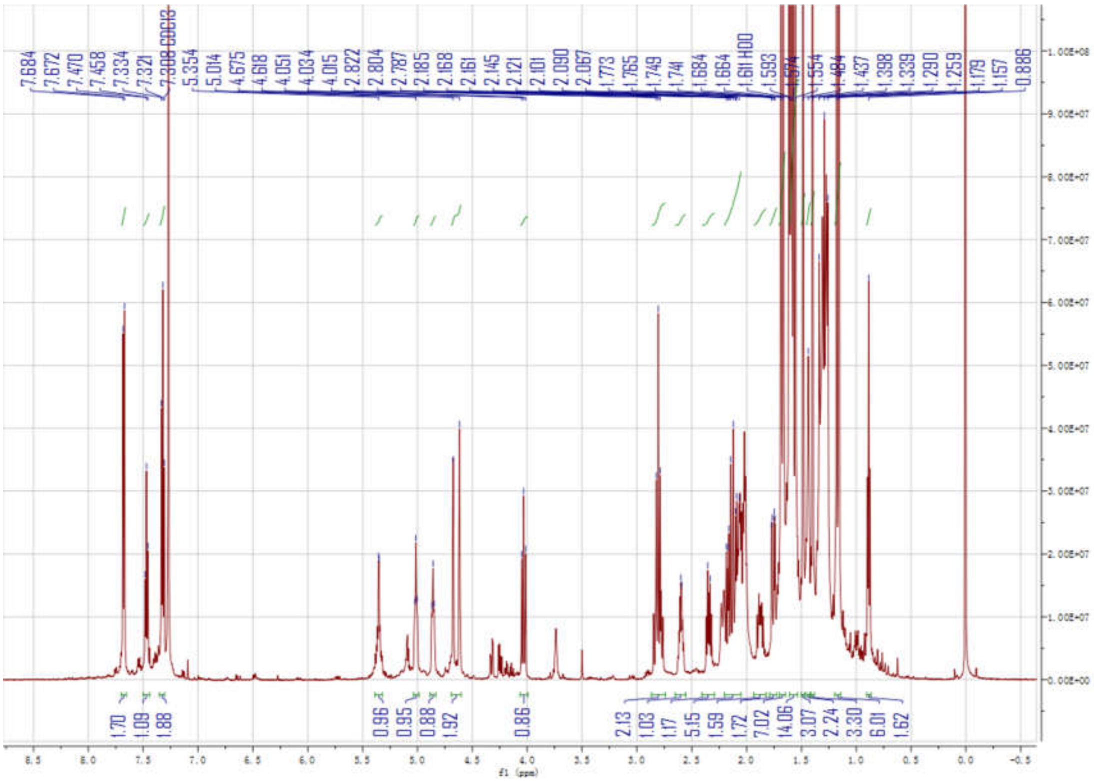

Figure S77: ^{1}H-NMR (600 MHz, $CDCl_3$) spectrum of compound 9

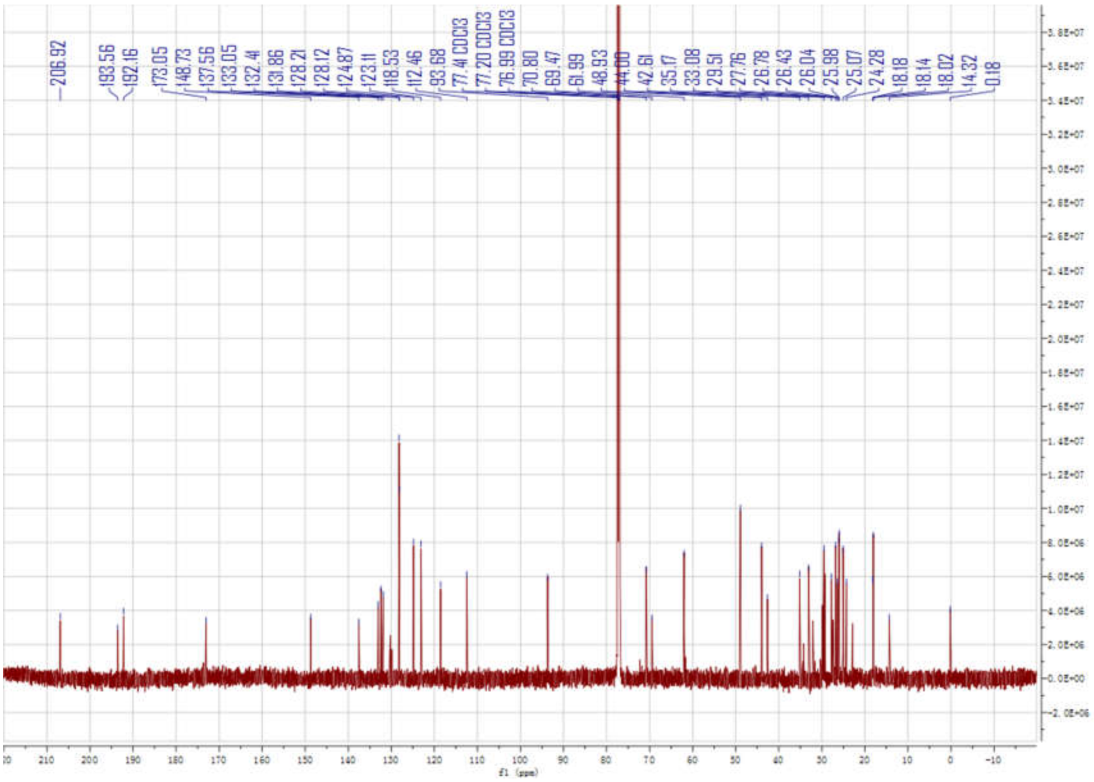

Figure S78: ^{13}C-NMR (150 MHz, $CDCl_3$) spectrum of compound 9

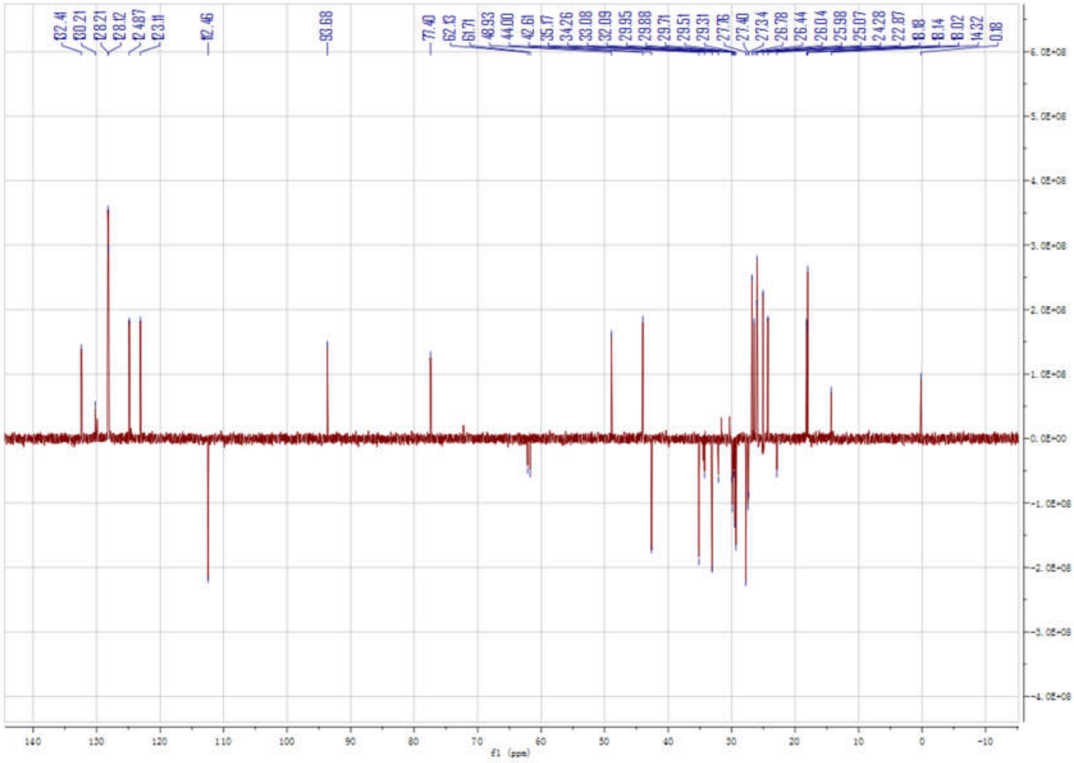

Figure S79: ^{13}C-NMR-DEPT (θ=135°) spectrum of compound 9

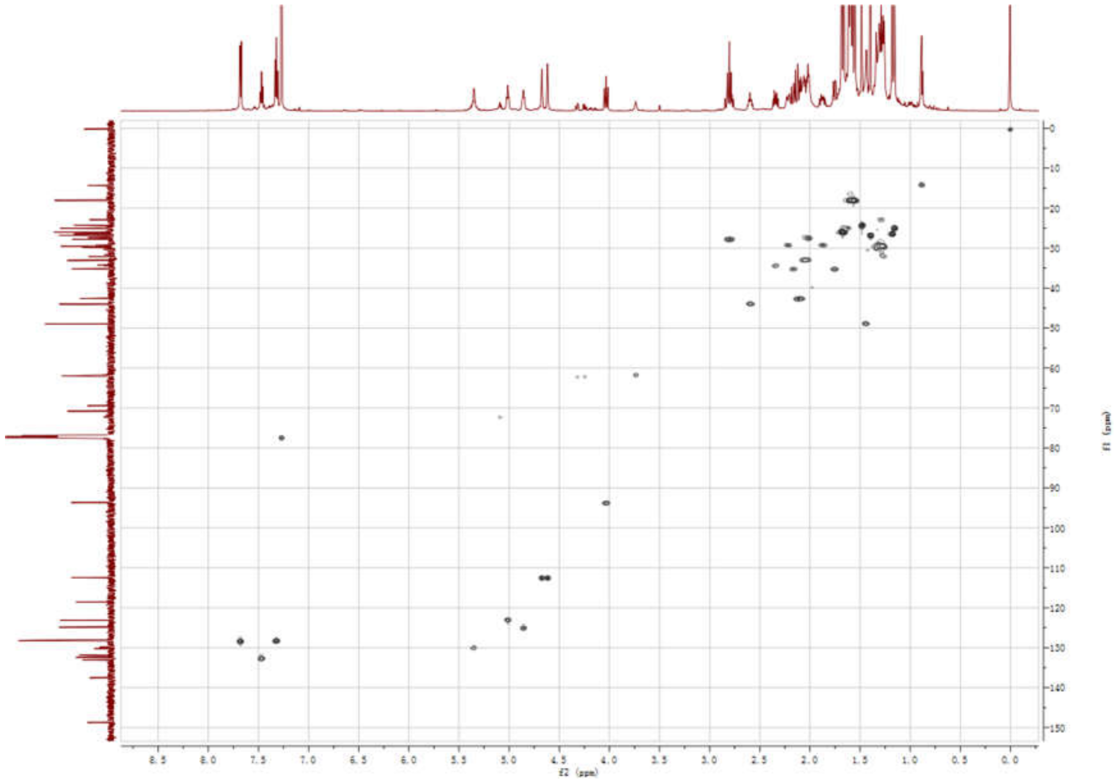

Figure S80: HSQC spectrum of compound 9

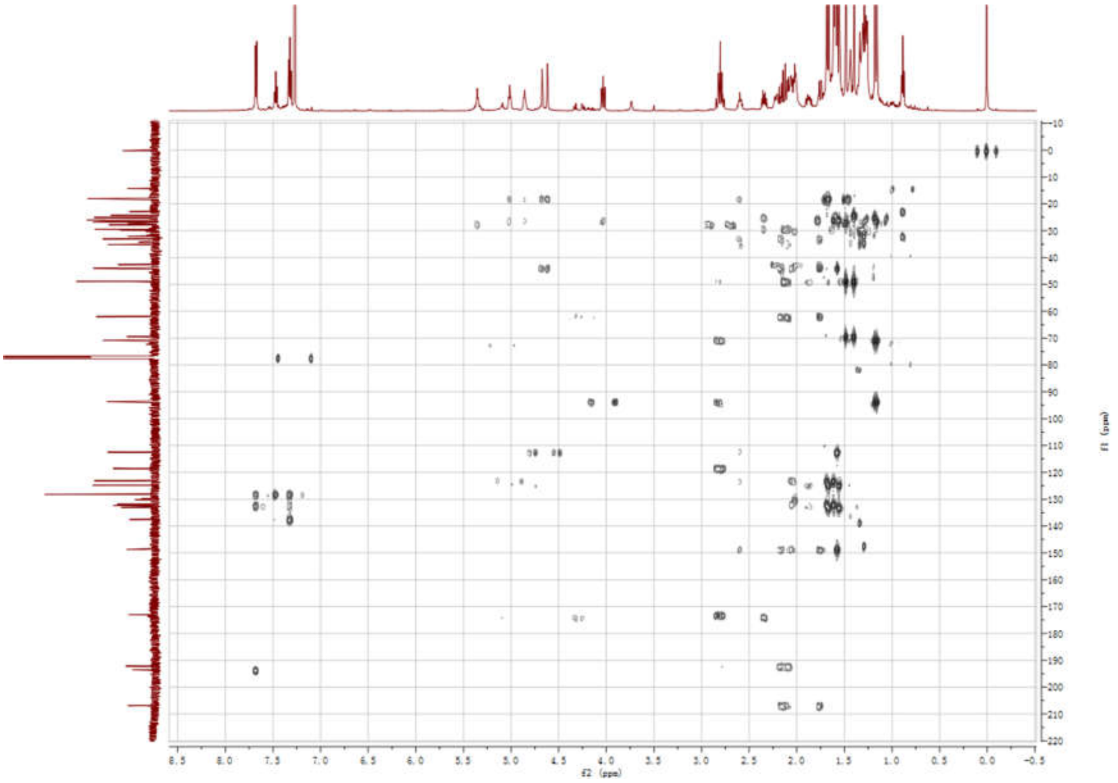

Figure S81: HMBC spectrum of compound 9

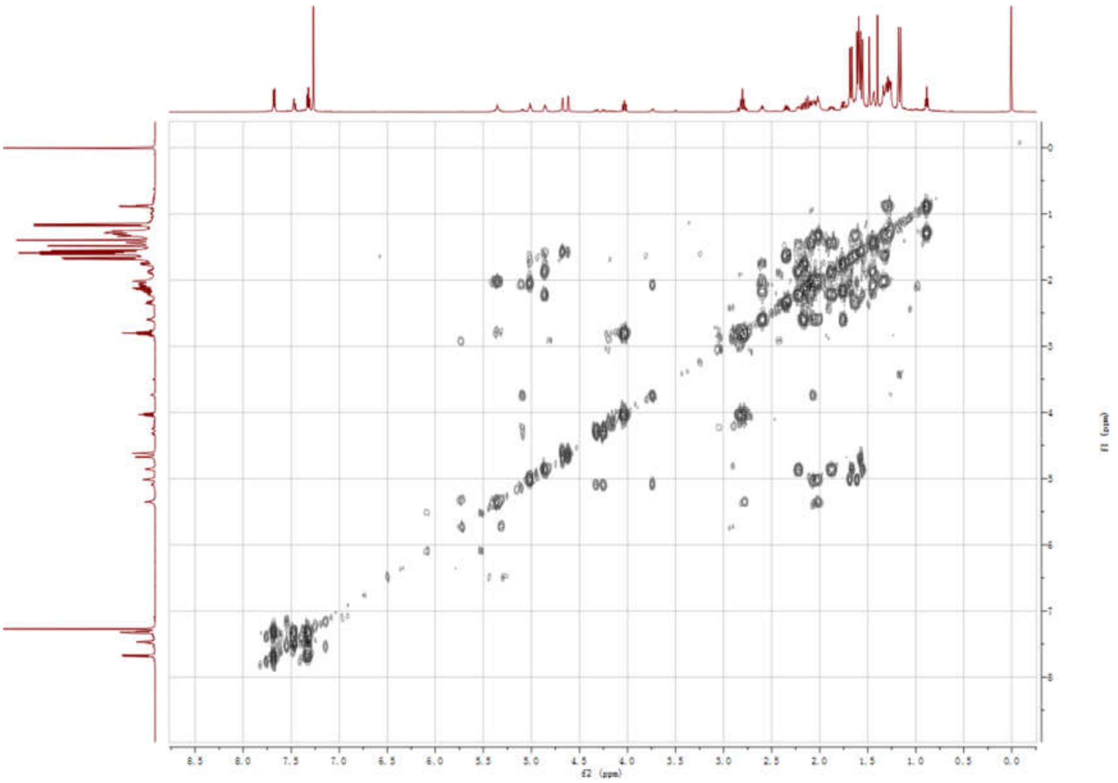

Figure S82: ^{1}H-^{1}H COSY spectrum of compound 9

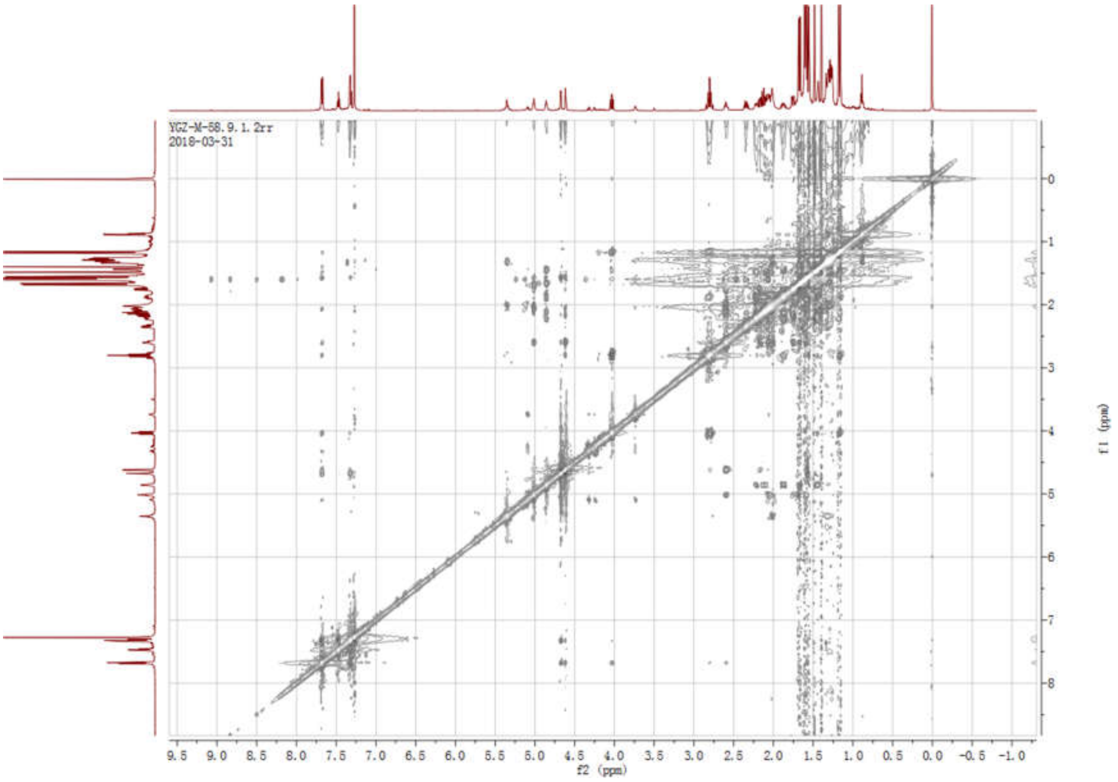

Figure S83: ROESY spectrum of compound 9

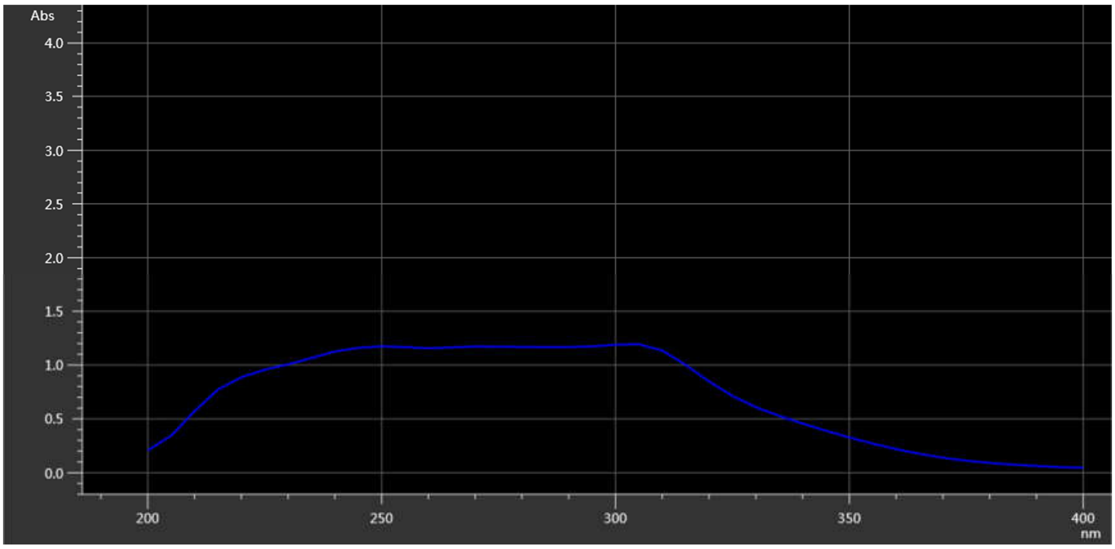

Figure S84: UV spectrum of compound 9

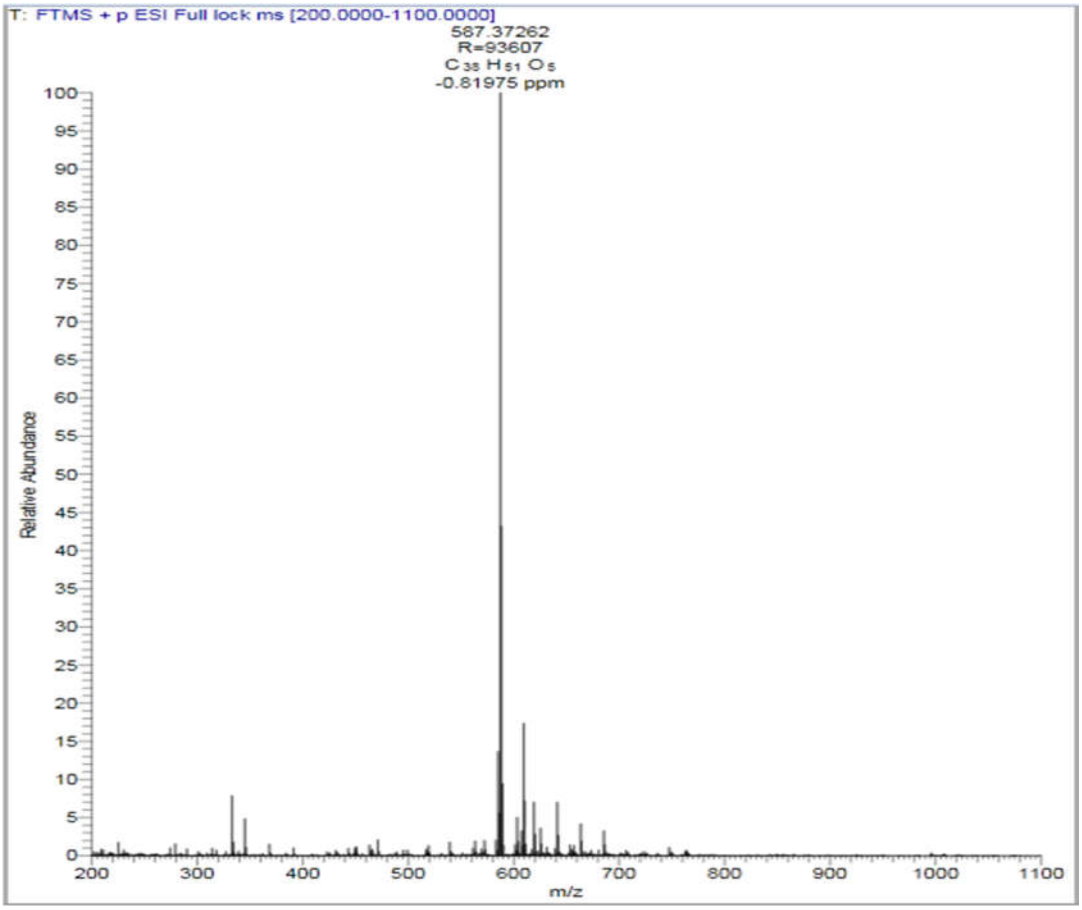

Figure S85: HR-ESI-MS of compound 9

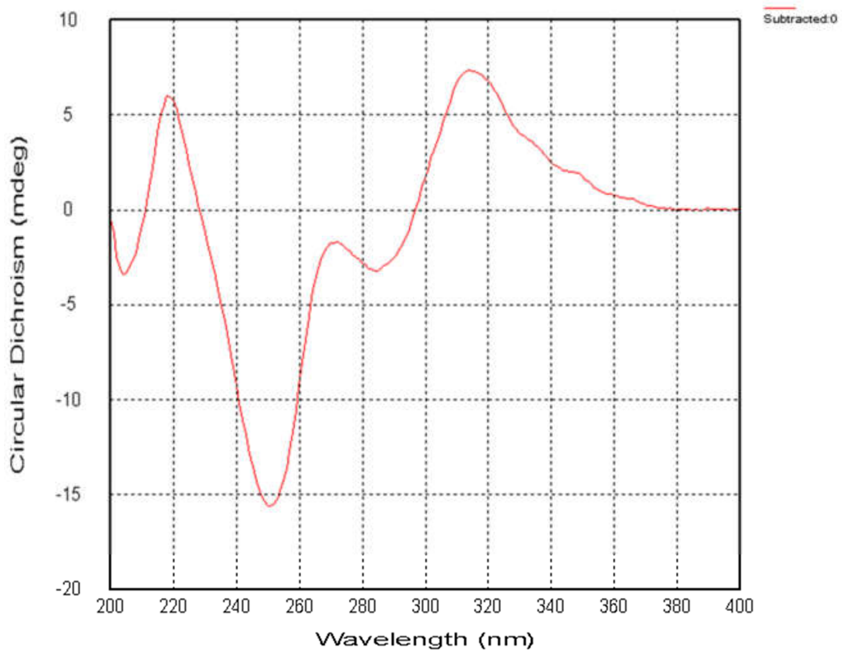

Figure S86: CD spectrum of compound 9

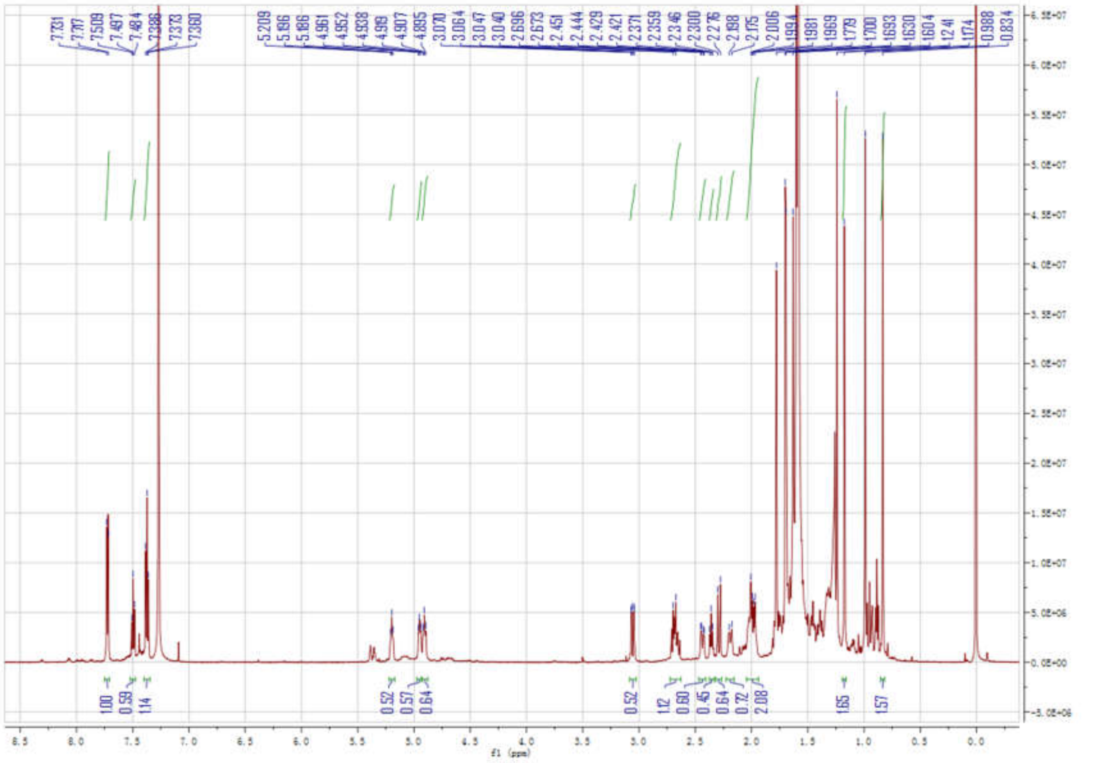

Figure S87: ^{1}H-NMR (600 MHz, $CDCl_3$) spectrum of compound 10

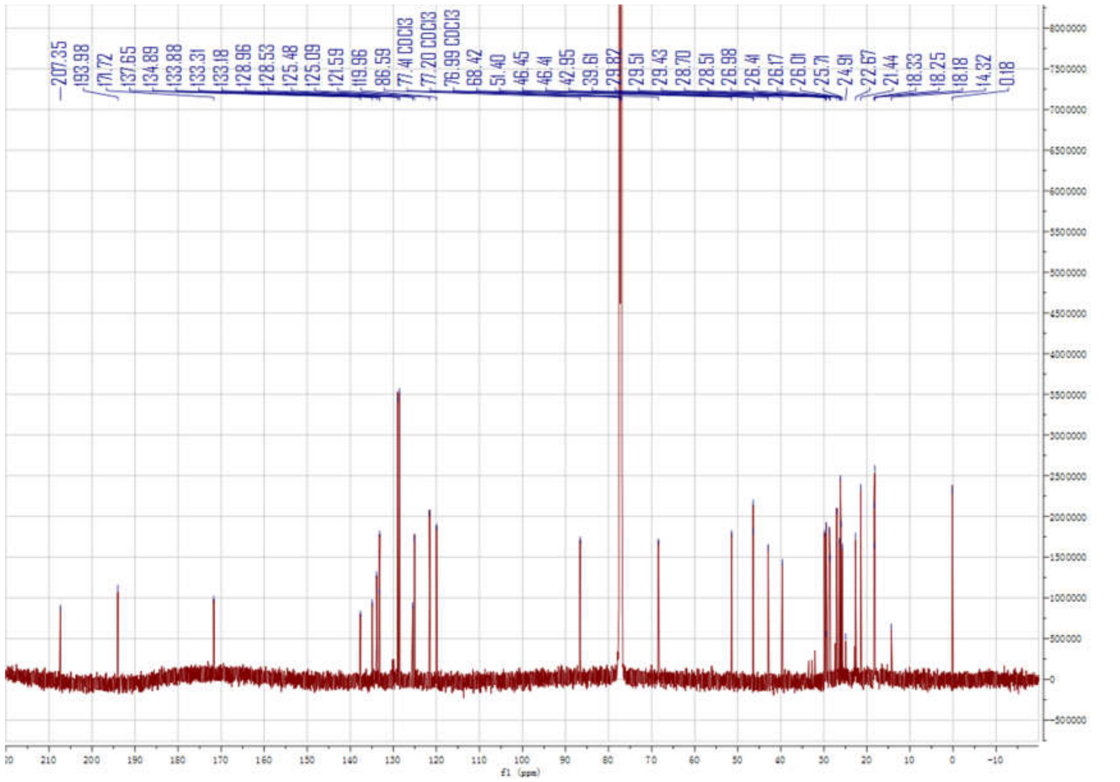

Figure S88: ^{13}C-NMR (150 MHz, $CDCl_3$) spectrum of compound 10

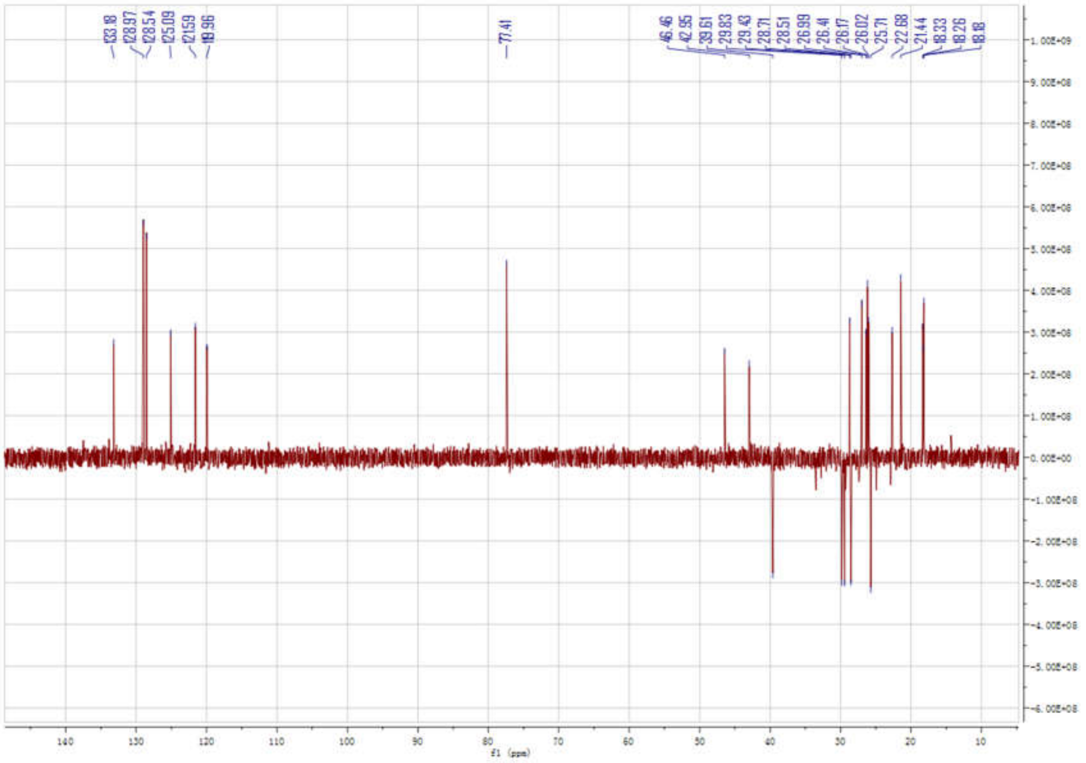

Figure S89: ^{13}C-NMR-DEPT (θ=135°) spectrum of compound 10

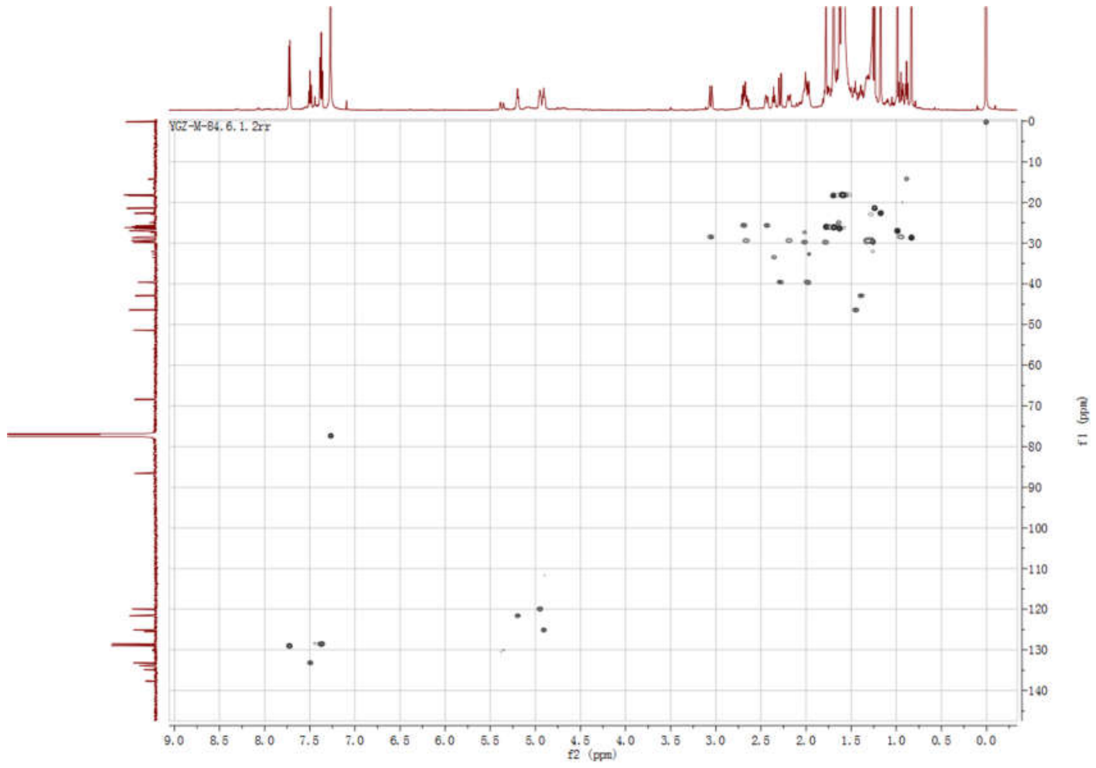

Figure S90: HSQC spectrum of compound 10

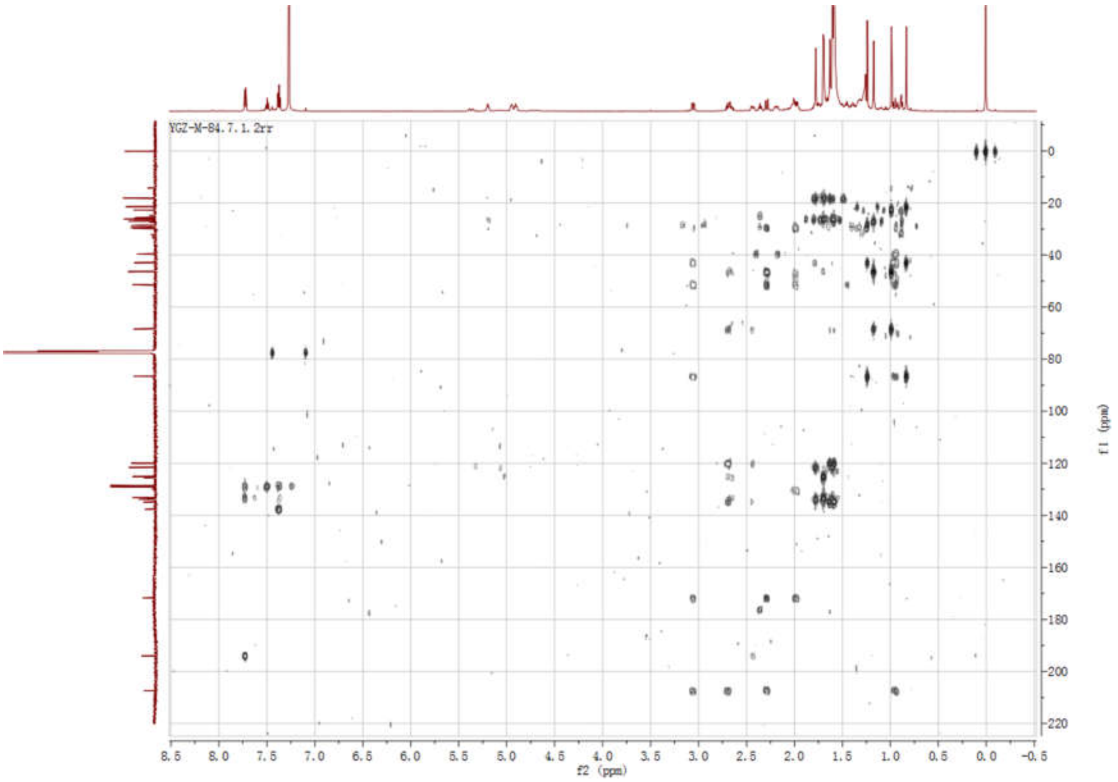

Figure S91: HMBC spectrum of compound 10

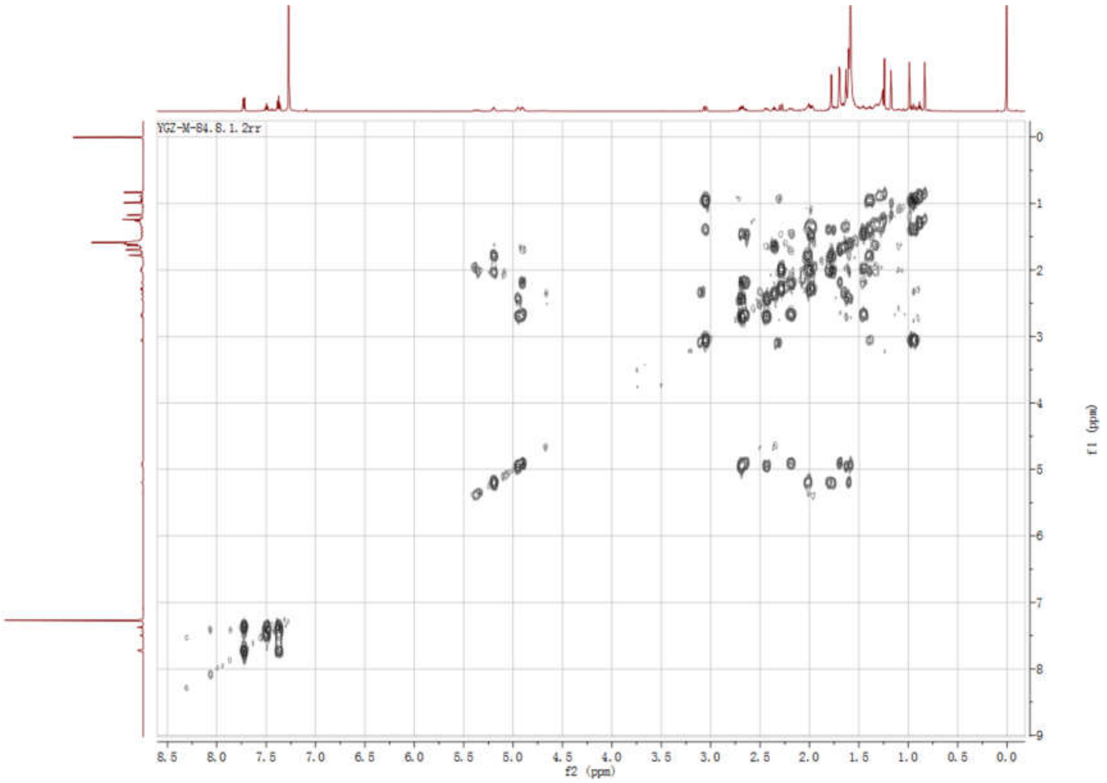

Figure S92: ^{1}H-^{1}H COSY spectrum of compound 10

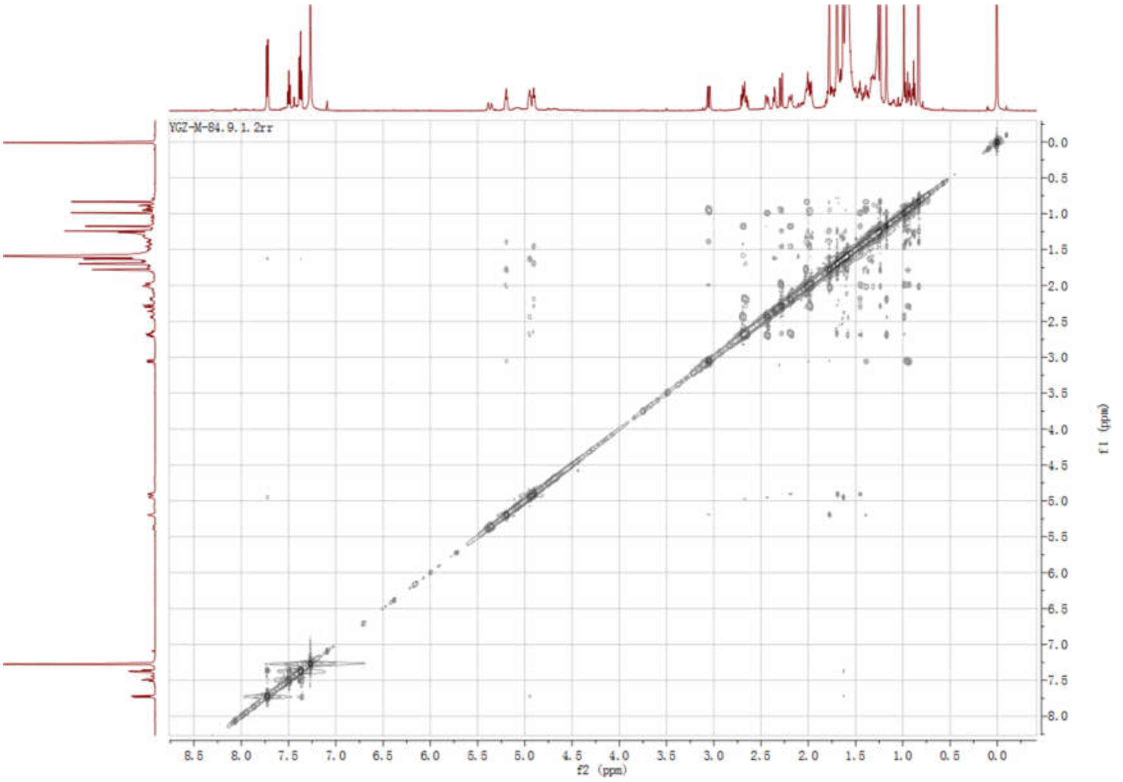

Figure S93: ROESY spectrum of compound 10

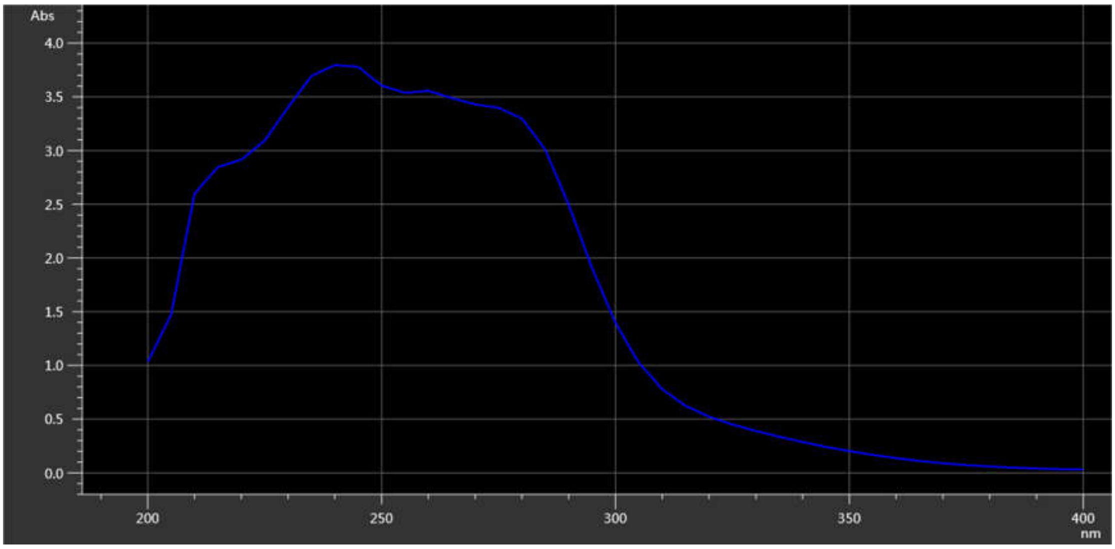

Figure S94: UV spectrum of compound 10

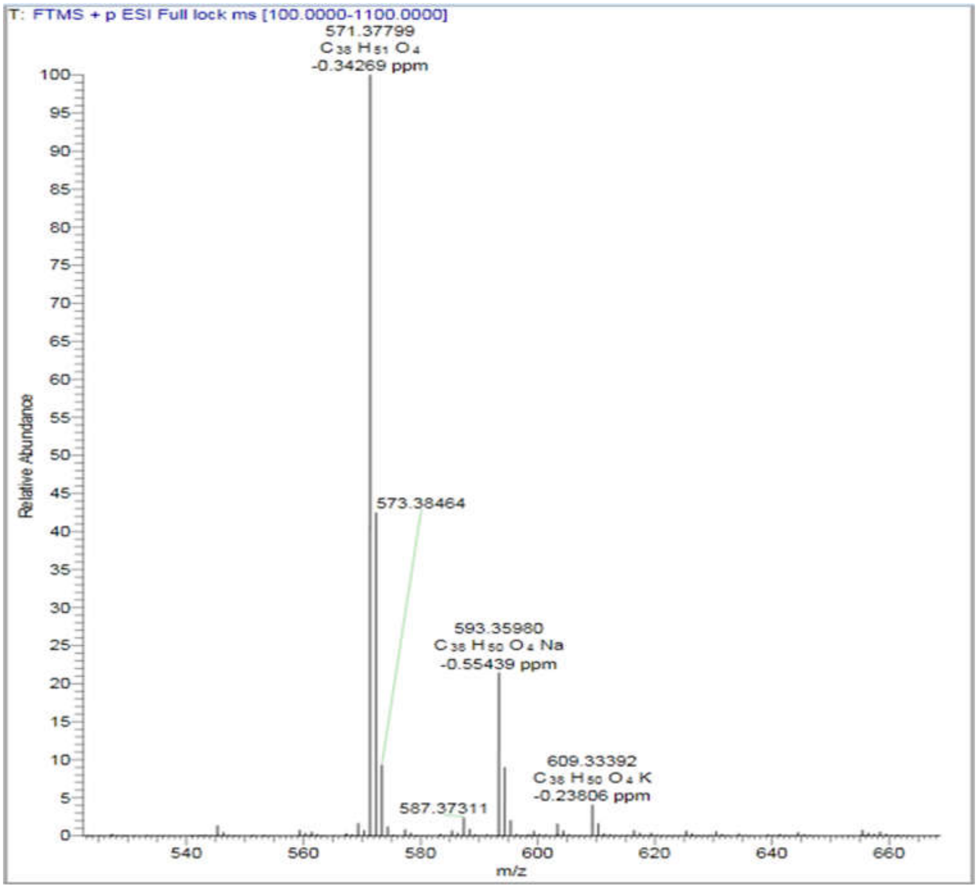

Figure S95: HR-ESI-MS of compound 10

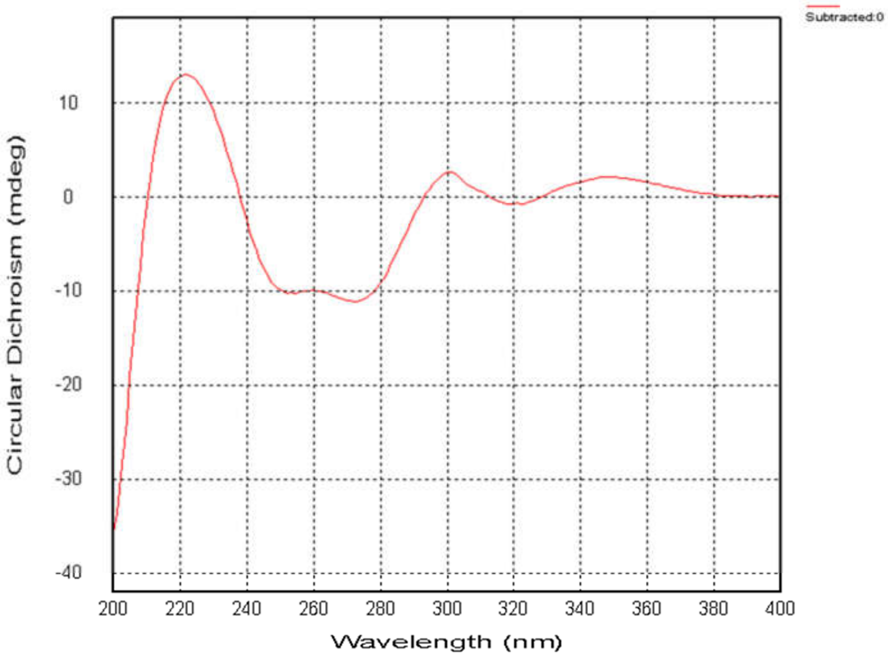

Figure S96: CD spectrum of compound 10

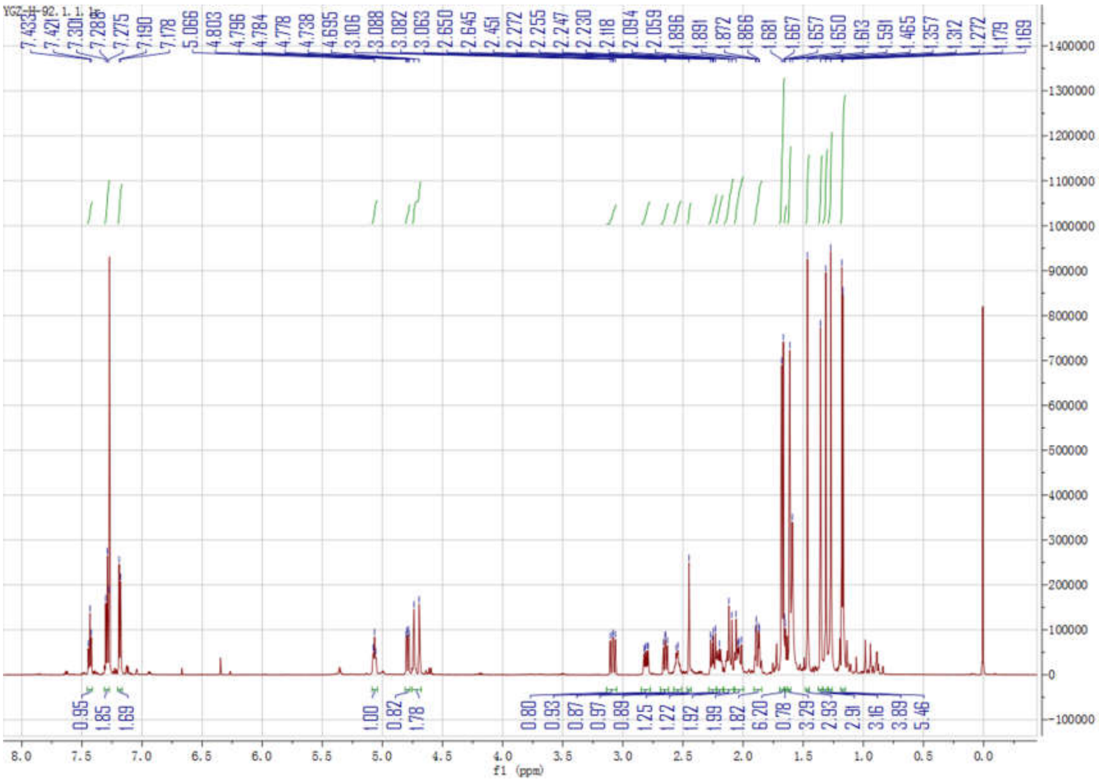

Figure S97: ^{1}H-NMR (600 MHz, $CDCl_3$) spectrum of compound 11

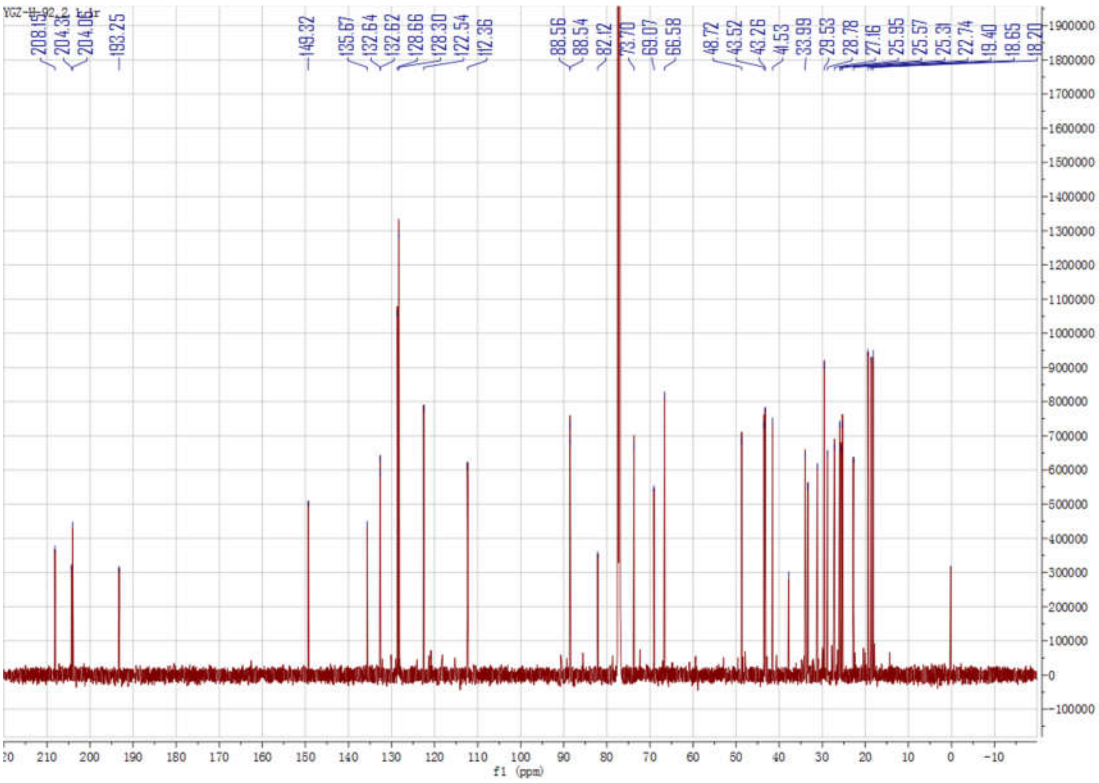

Figure S98: ^{13}C-NMR (150 MHz, $CDCl_3$) spectrum of compound 11

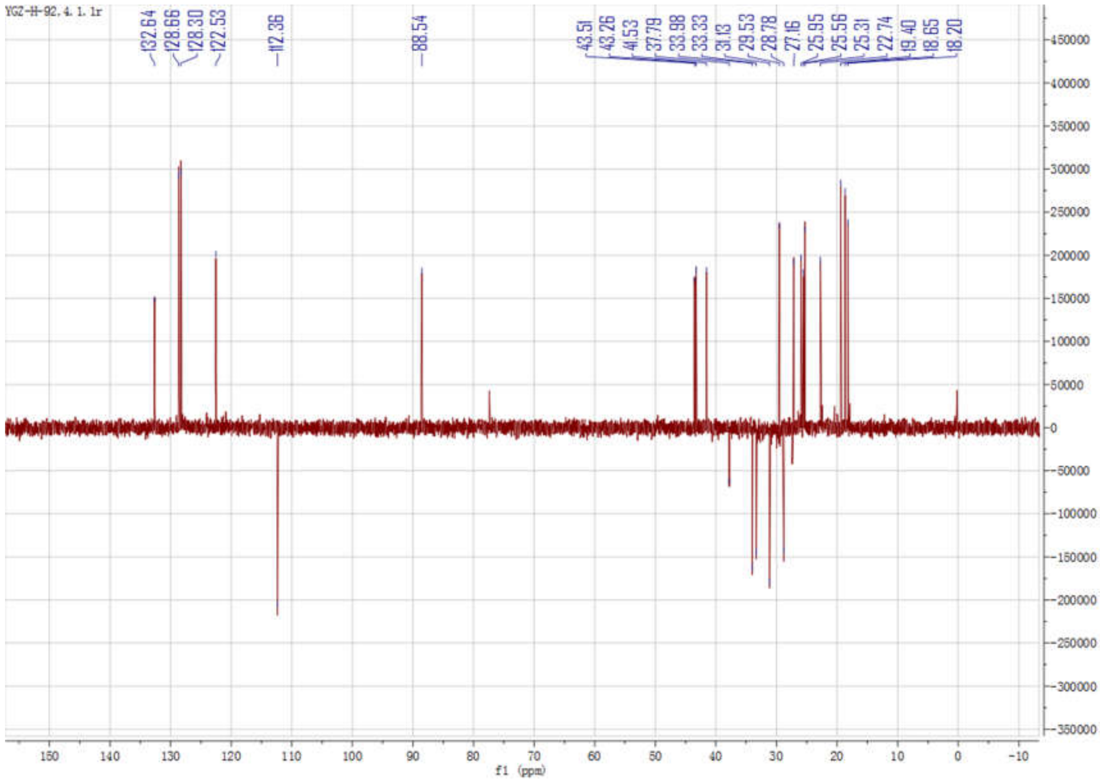

Figure S99: ^{13}C-NMR-DEPT (θ=135°) spectrum of compound 11

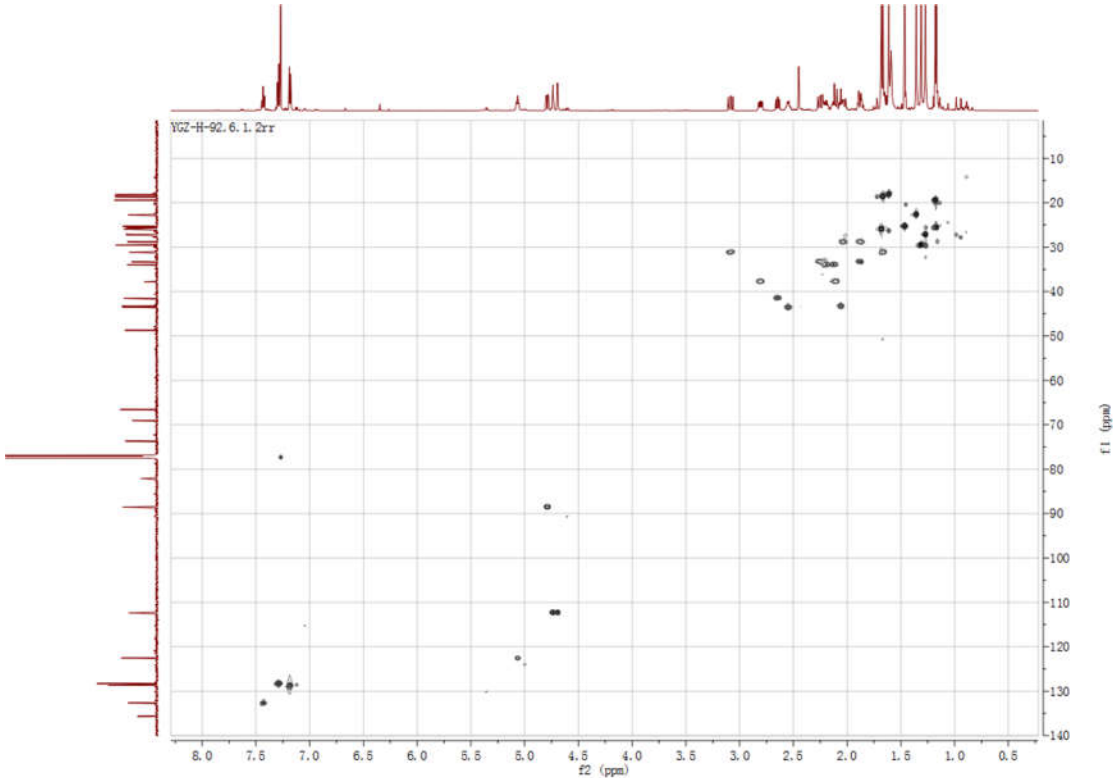

Figure S100: HSQC spectrum of compound 11

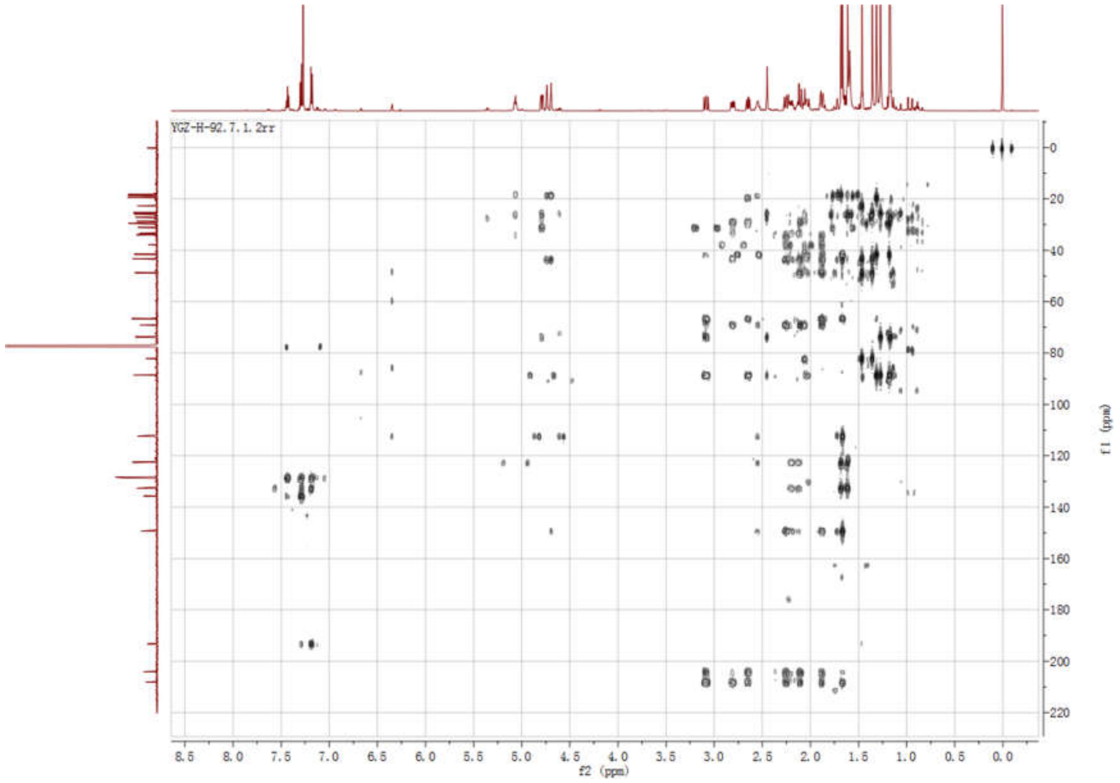

Figure S101: HMBC spectrum of compound 11

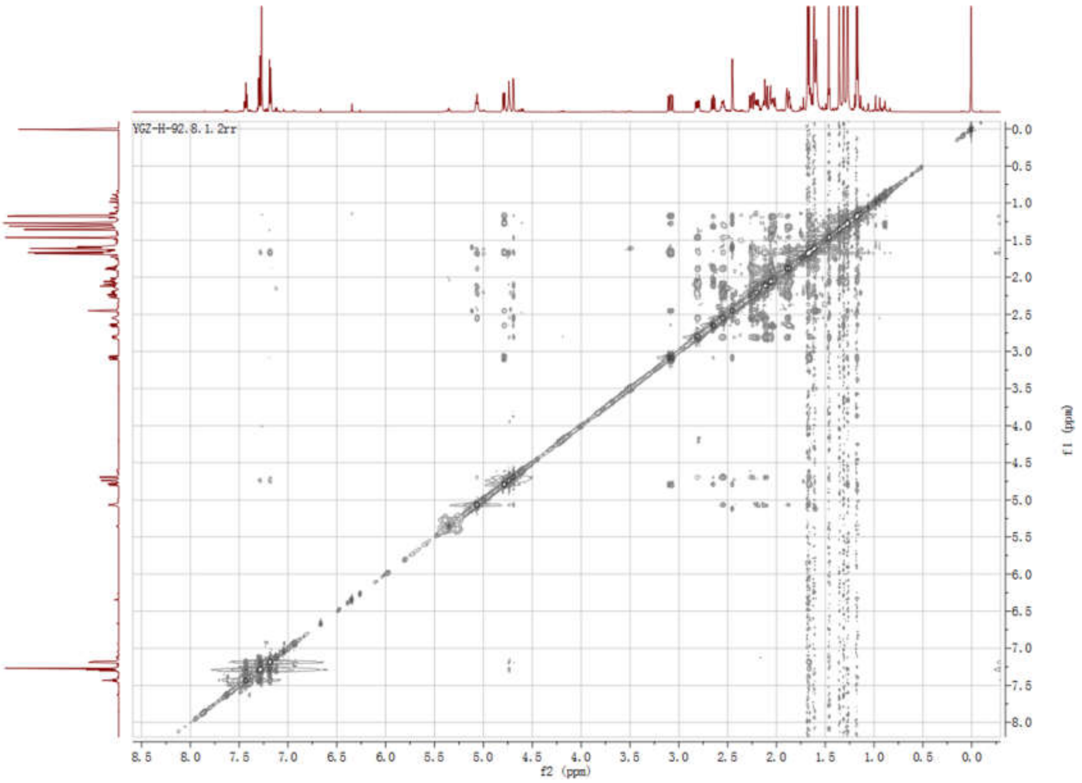

Figure S102: ROESY spectrum of compound 11

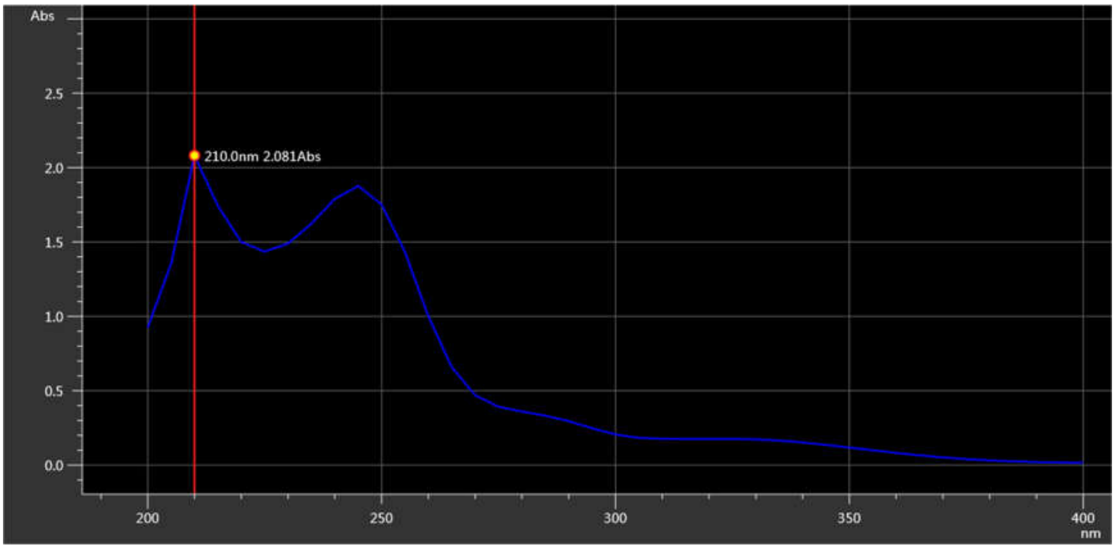

Figure S103: UV spectrum of compound 11

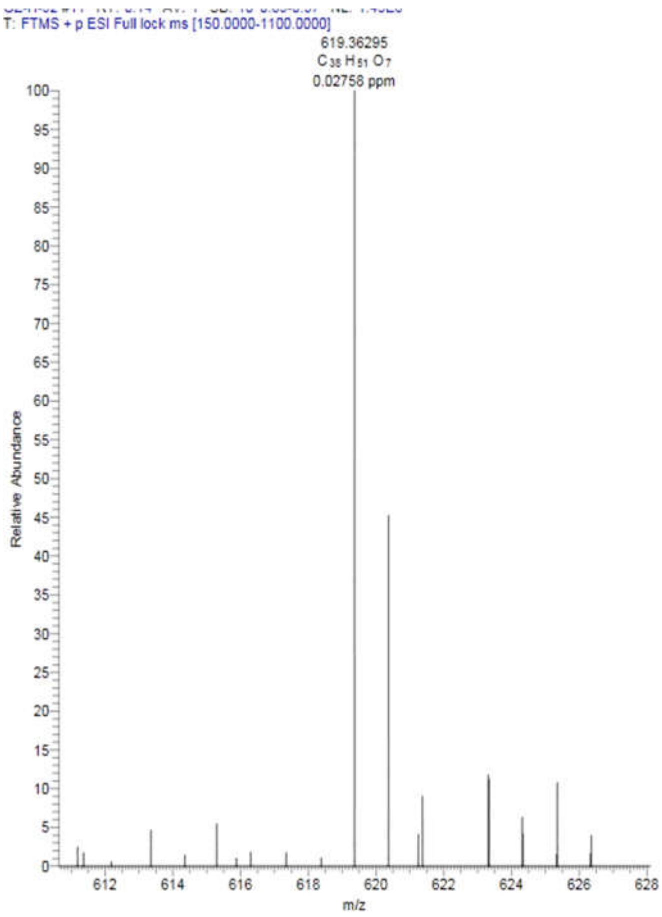

Figure S104: HR-ESI-MS of compound 11

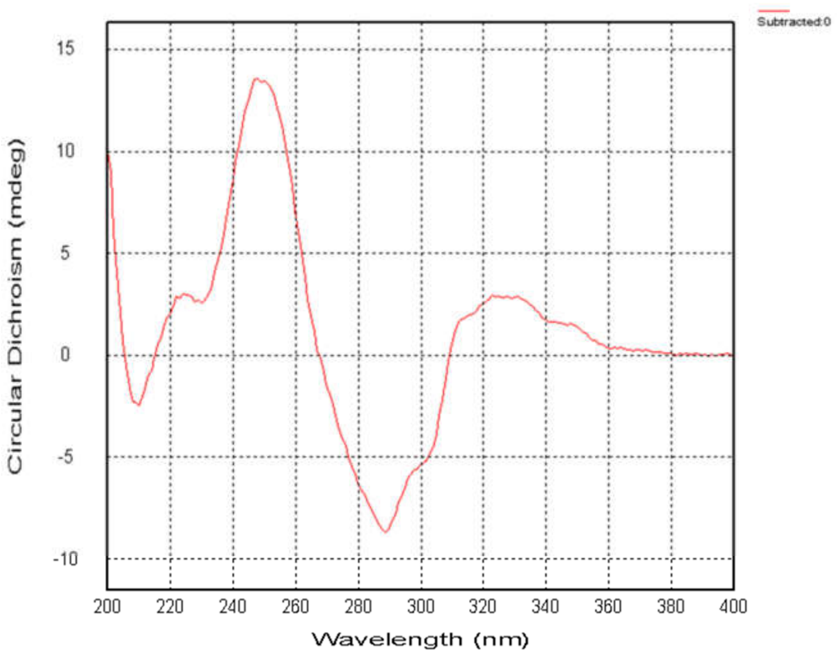

Figure S105: CD spectrum of compound 11

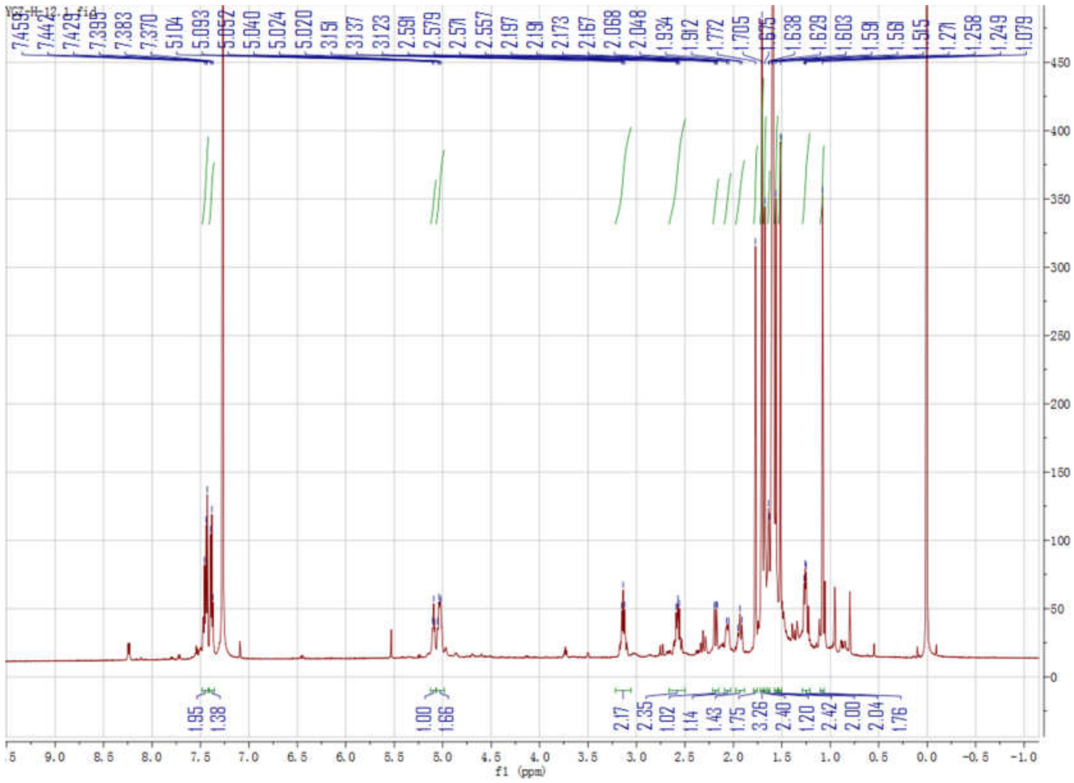

Figure S106: ^{1}H-NMR (600 MHz, $CDCl_3$) spectrum of compound 12

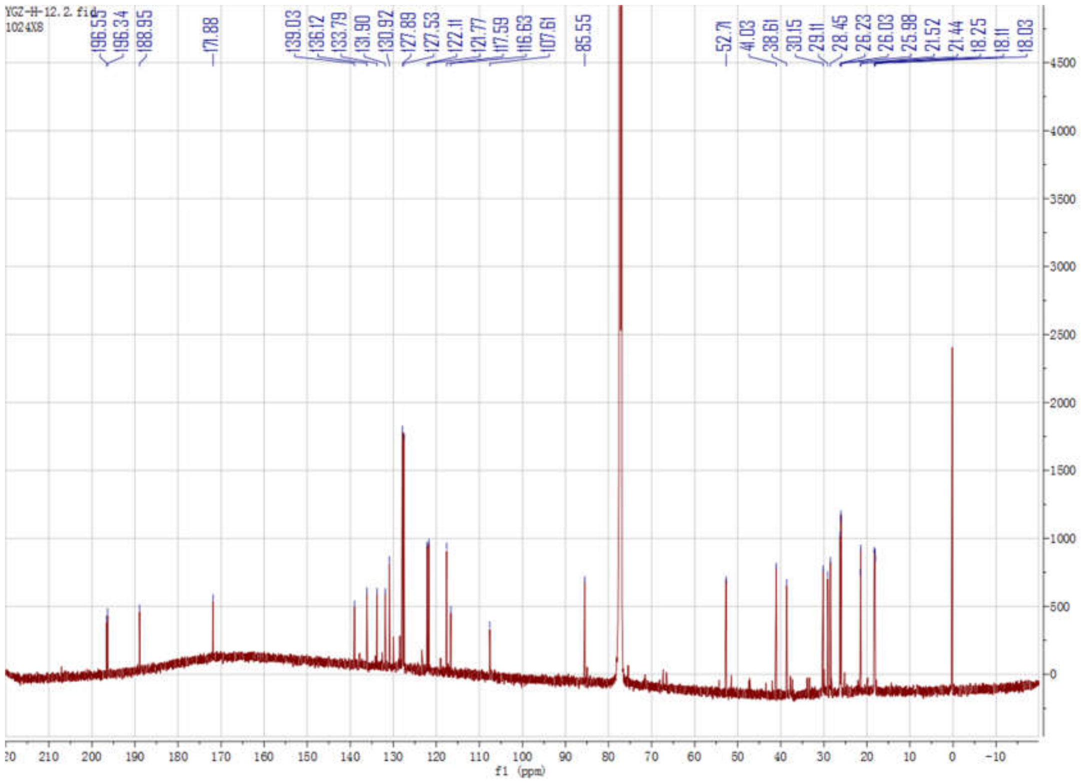

Figure S107: ^{13}C-NMR (150 MHz, $CDCl_3$) spectrum of compound 12

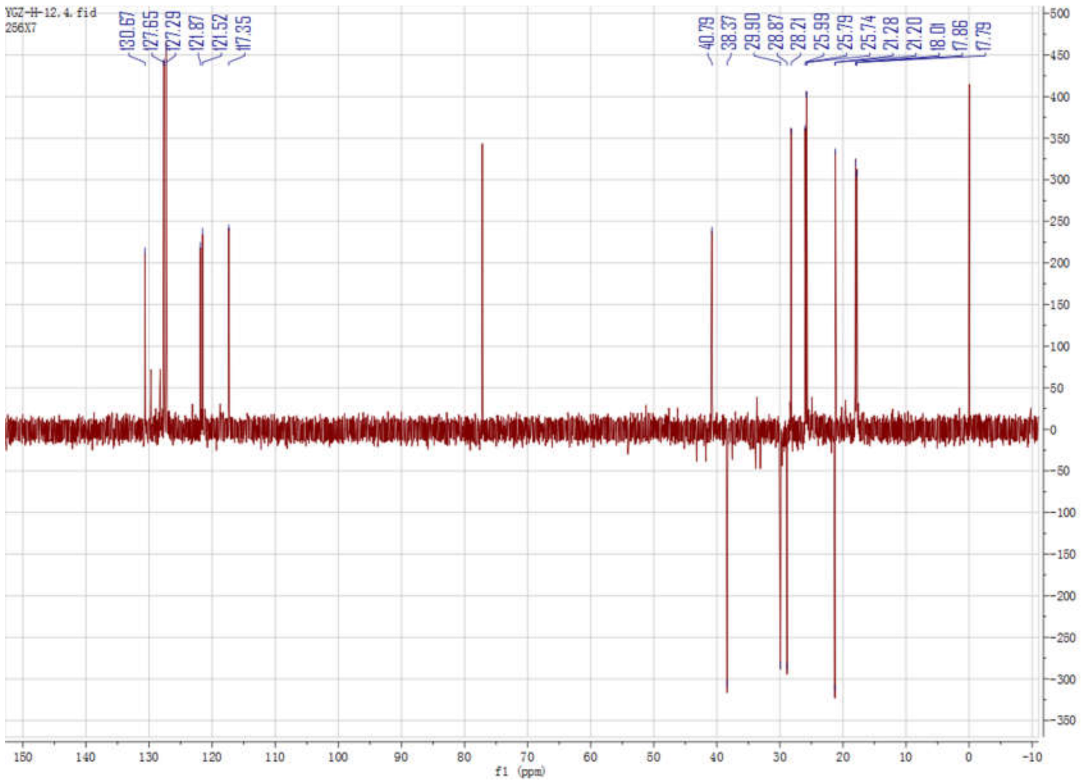

Figure S108: ^{13}C-NMR-DEPT (θ=135°) spectrum of compound 12

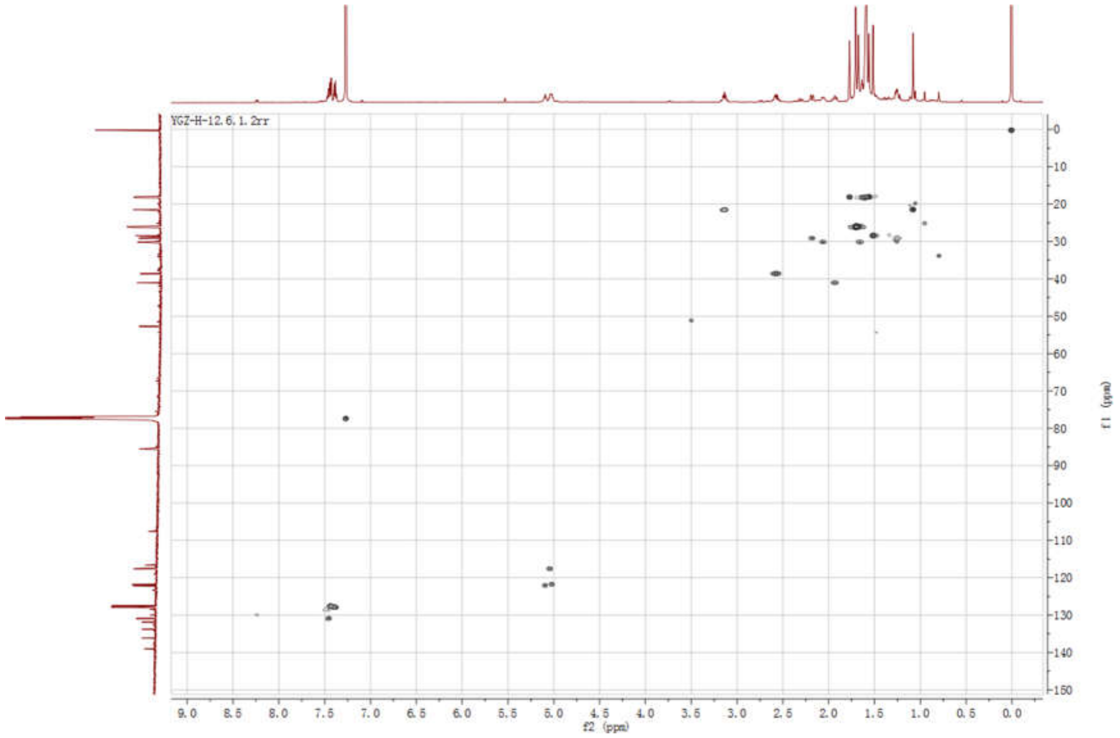

Figure S109: HSQC spectrum of compound 12

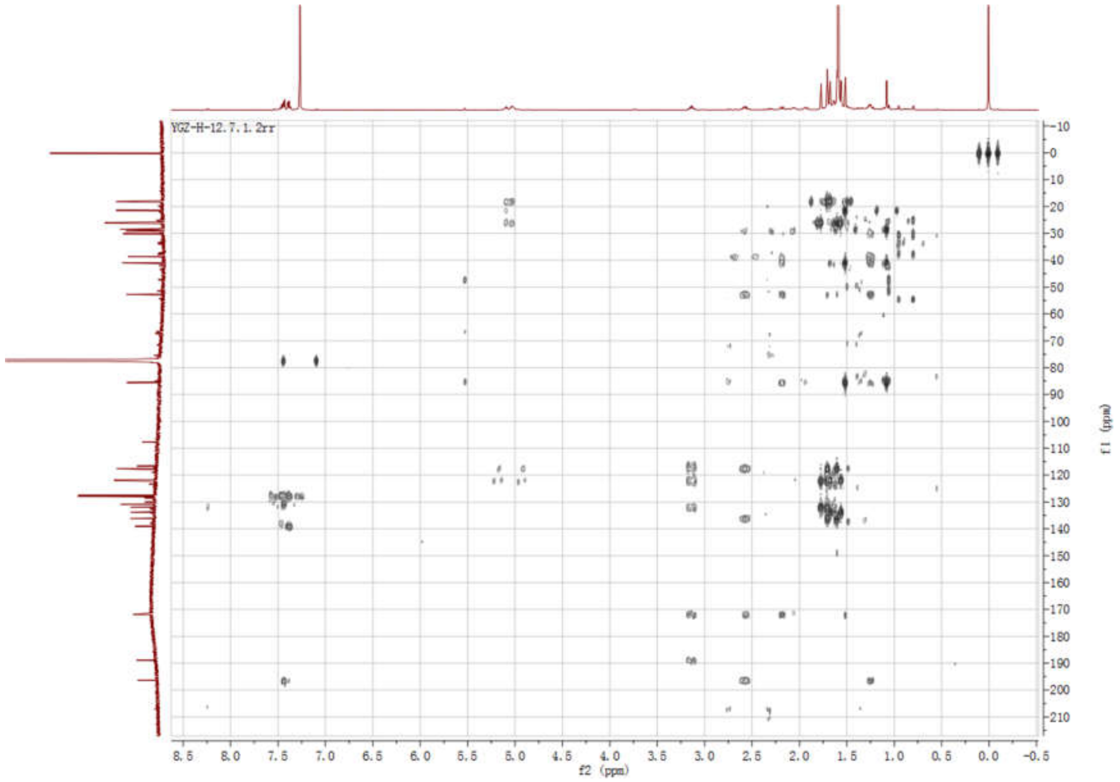

Figure S110: HMBC spectrum of compound 12

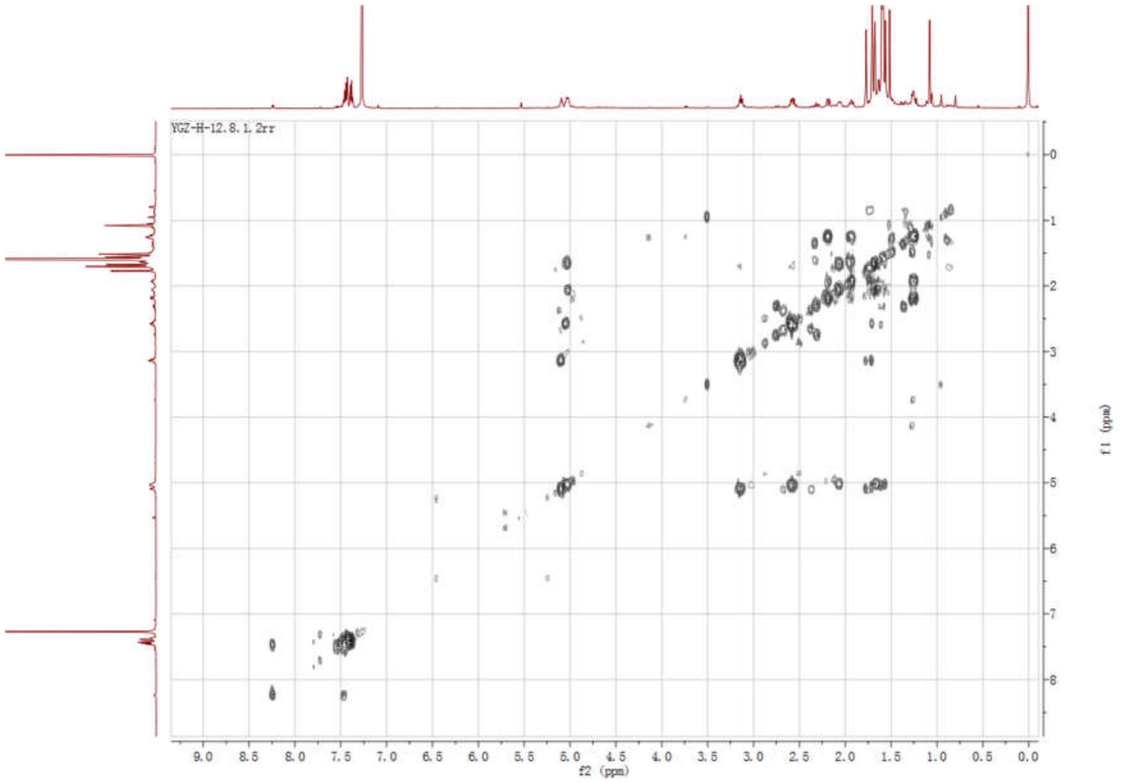

Figure S111: ^{1}H-^{1}H COSY spectrum of compound 12

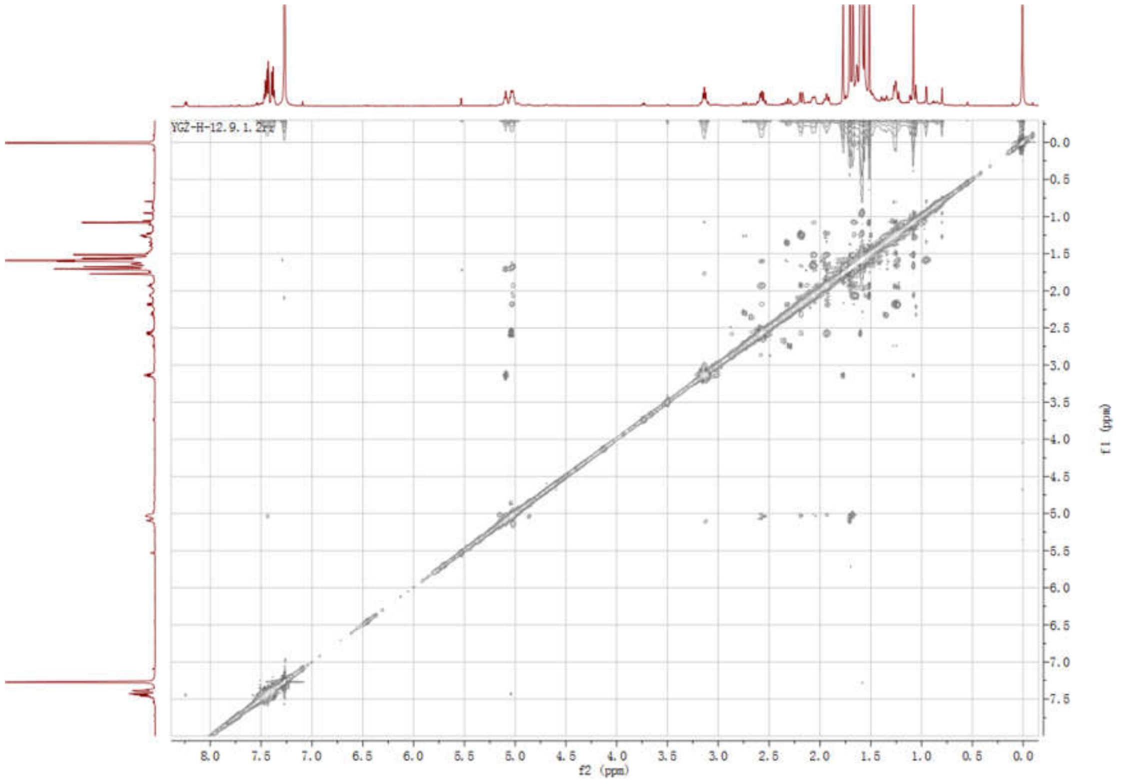

Figure S112: ROESY spectrum of compound 12

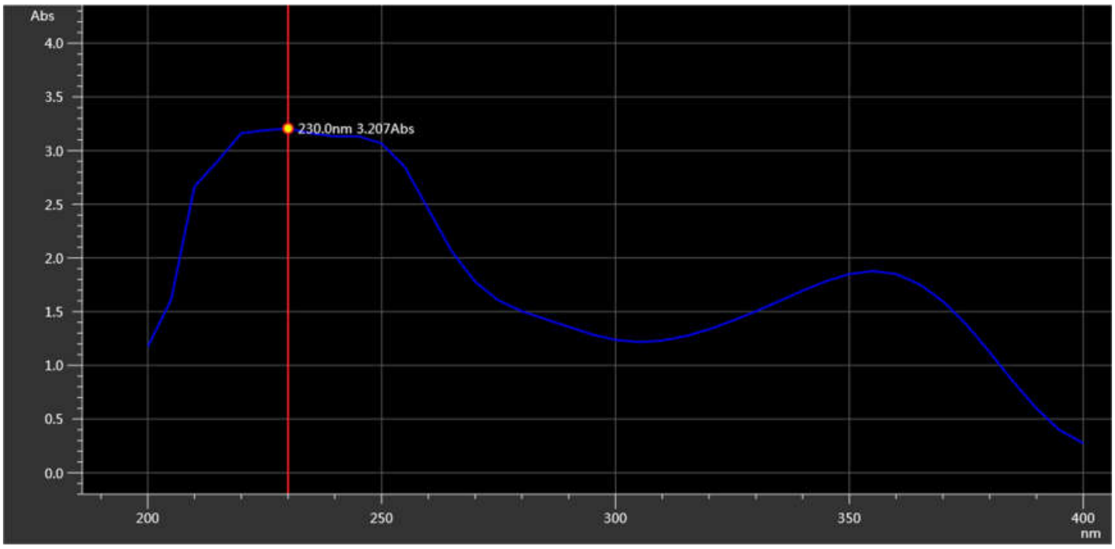

Figure S113: UV spectrum of compound 12

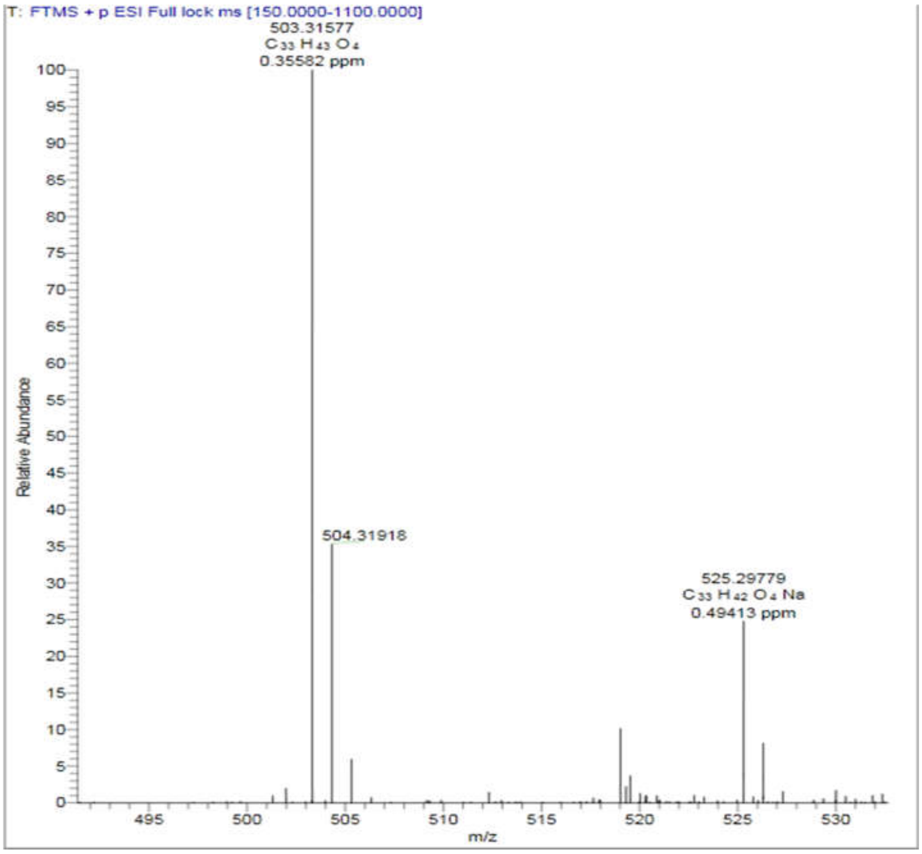

Figure S114: HR-ESI-MS of compound 12

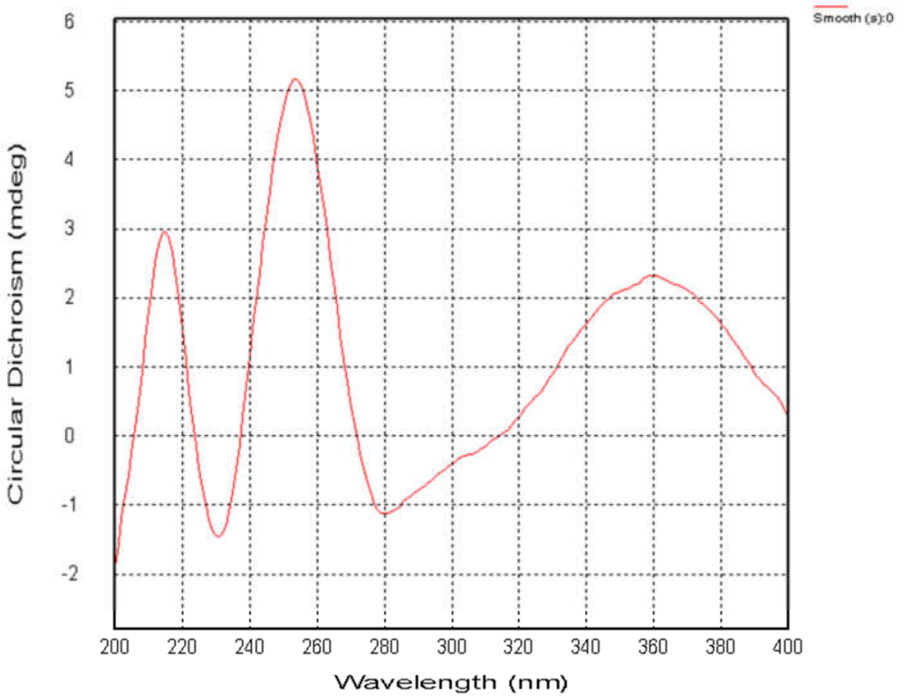

Figure S115: CD spectrum of compound 12

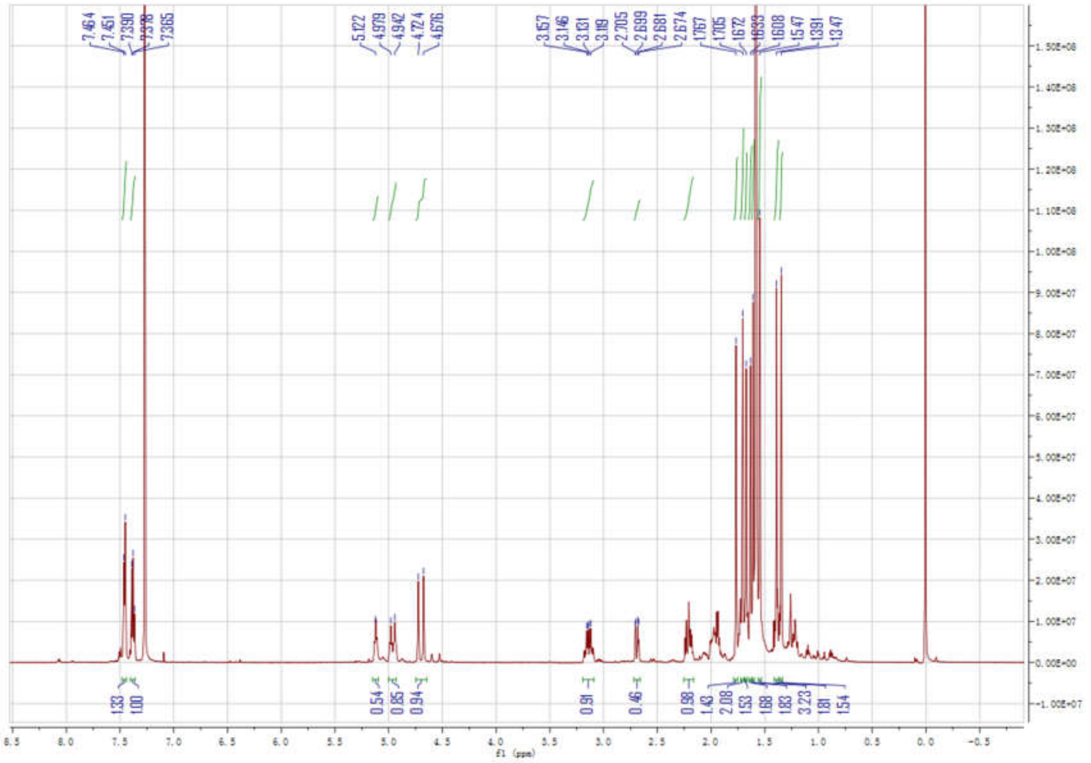

Figure S116: ^{1}H-NMR (600 MHz, $CDCl_3$) spectrum of compound 13

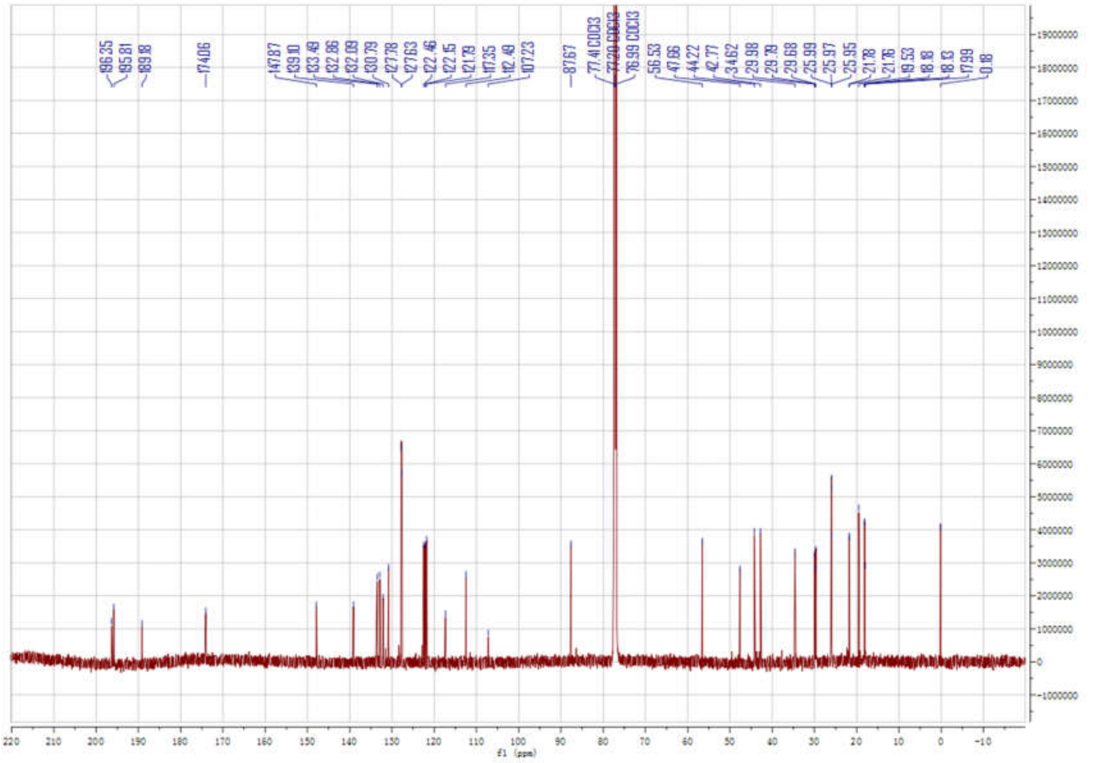

Figure S117: ^{13}C-NMR (150 MHz, CDCl$_3$) spectrum of compound 13

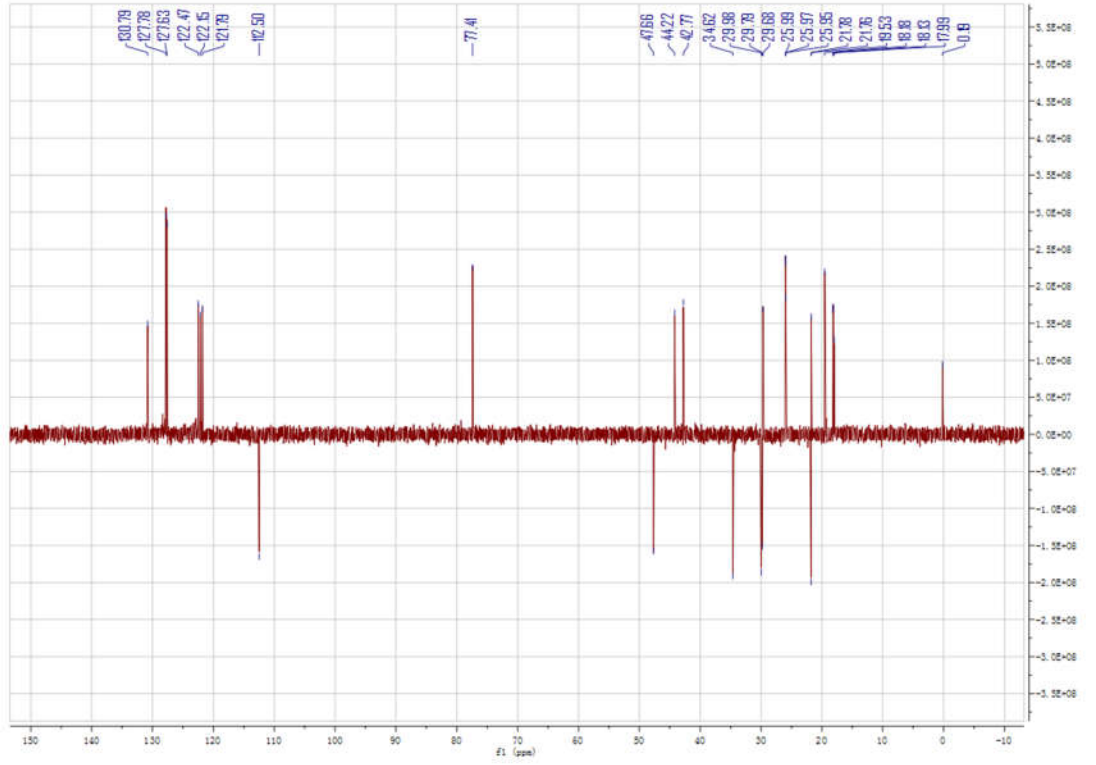

Figure S118: ^{13}C-NMR-DEPT (θ=135°) spectrum of compound 13

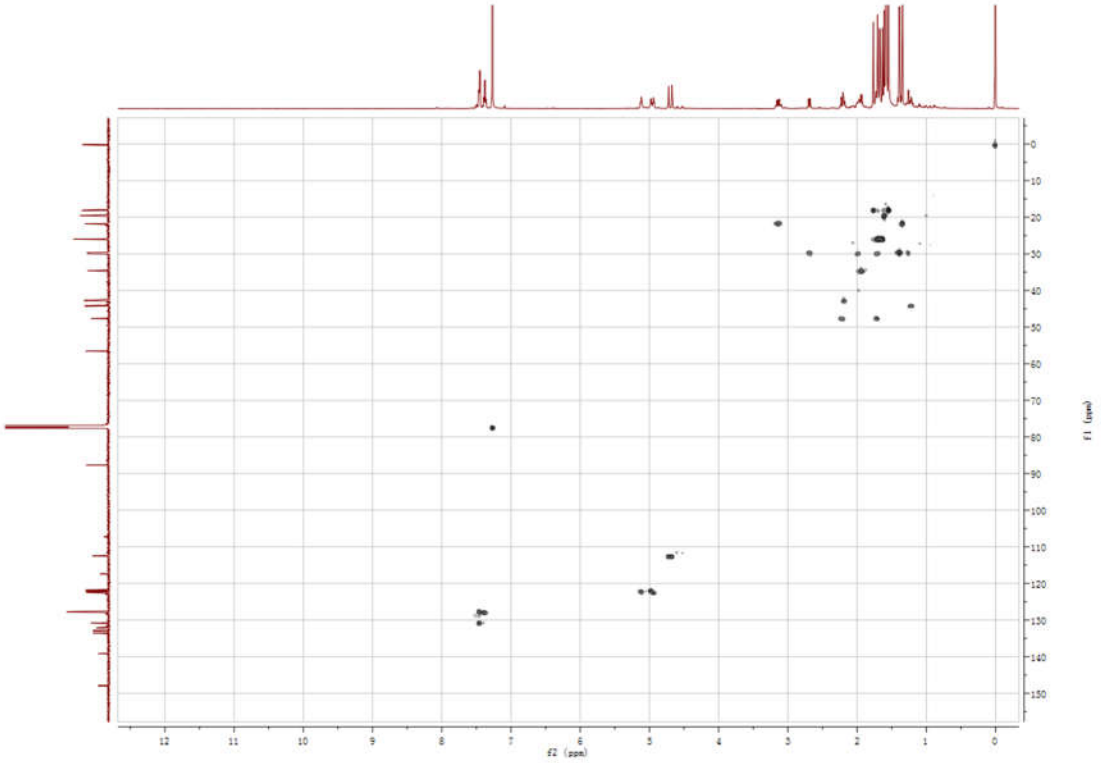

Figure S119: HSQC spectrum of compound 13

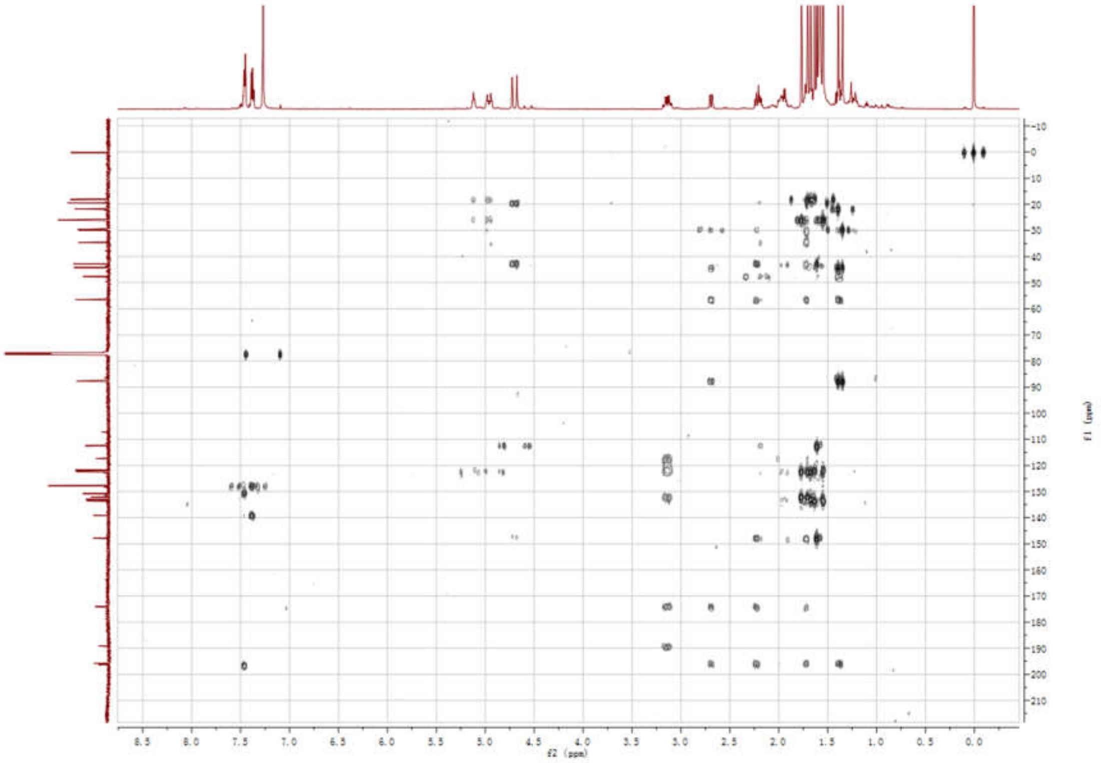

Figure S120: HMBC spectrum of compound 13

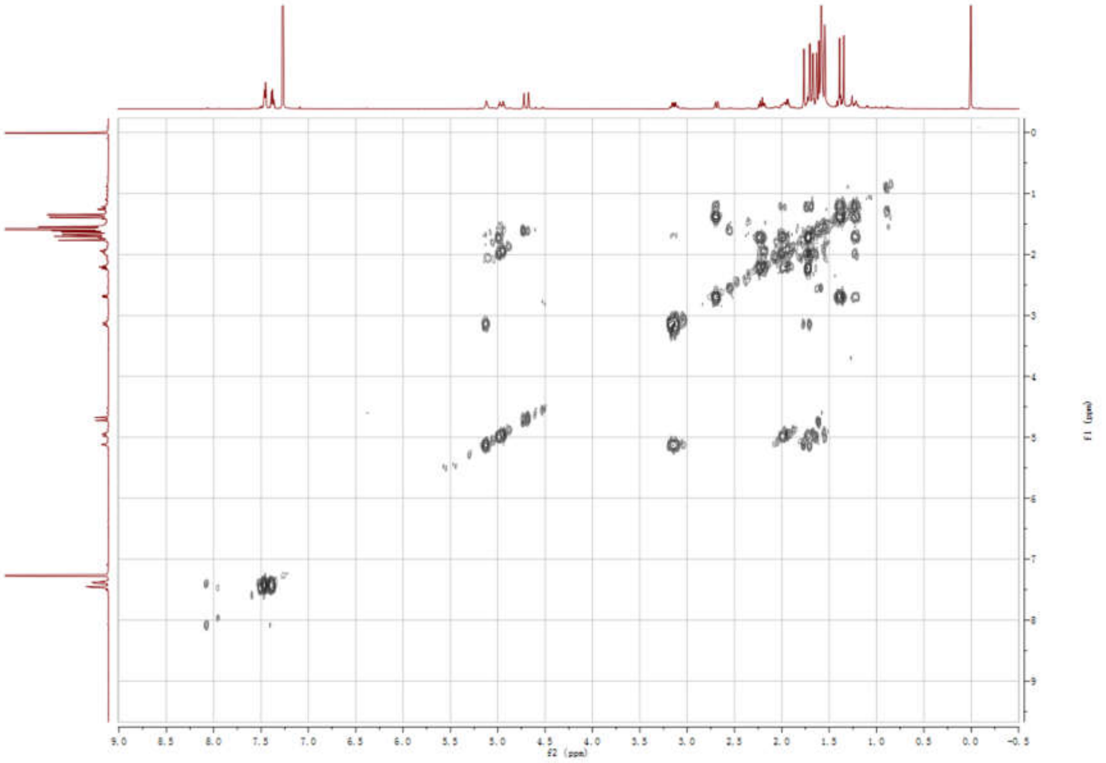

Figure S121: ^{1}H-^{1}H COSY spectrum of compound 13

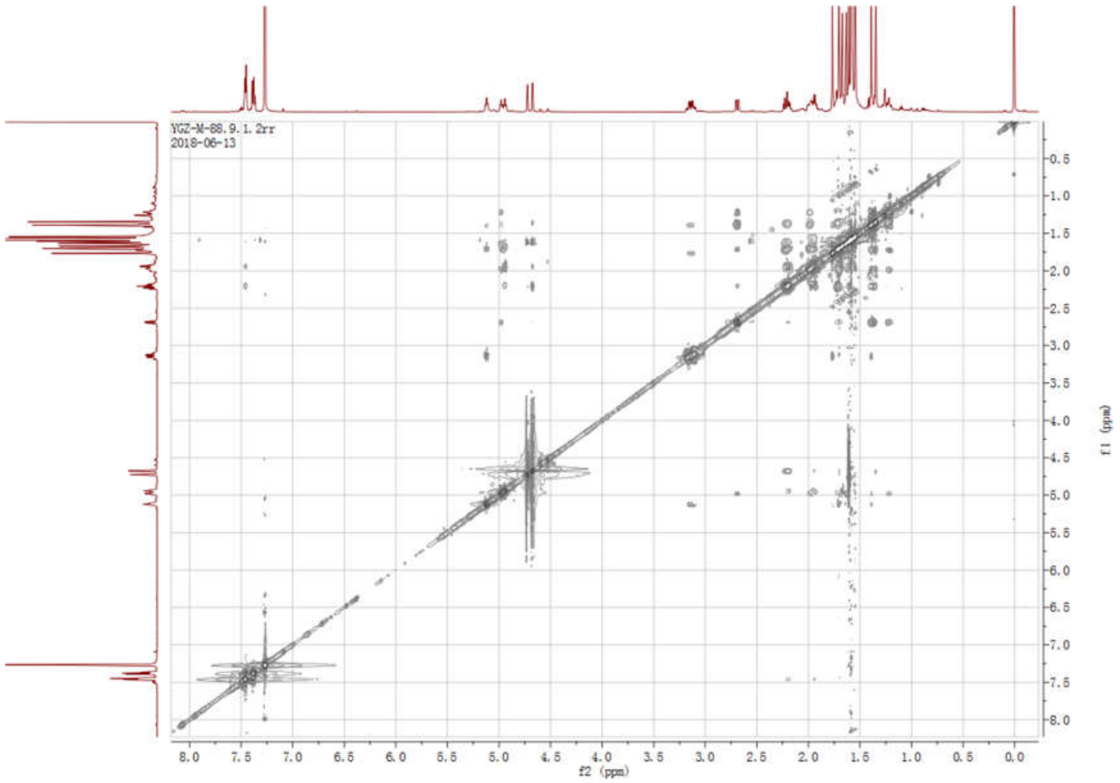

Figure S122: ROESY spectrum of compound 13

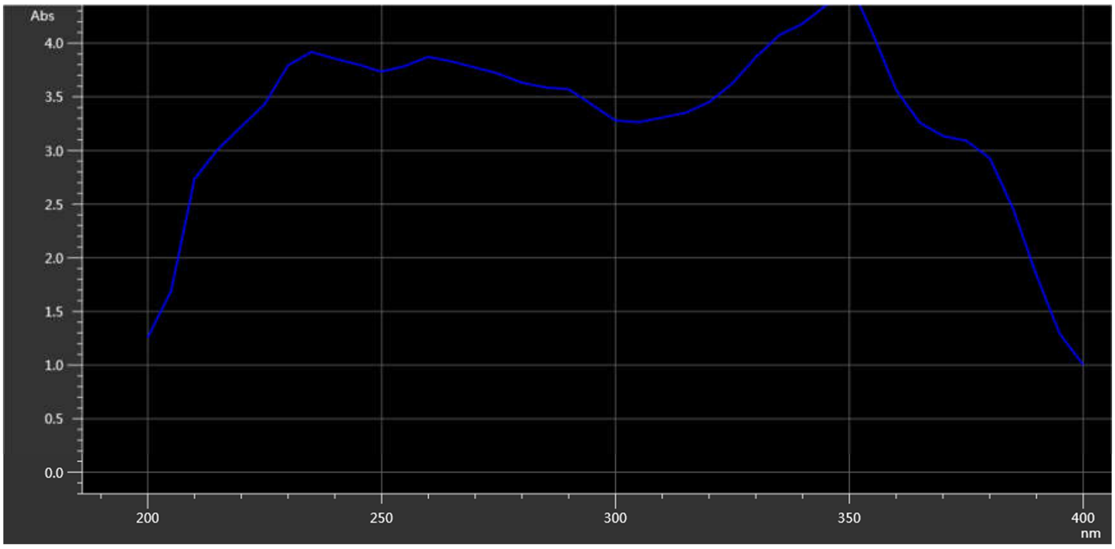

Figure S123: UV spectrum of compound 13

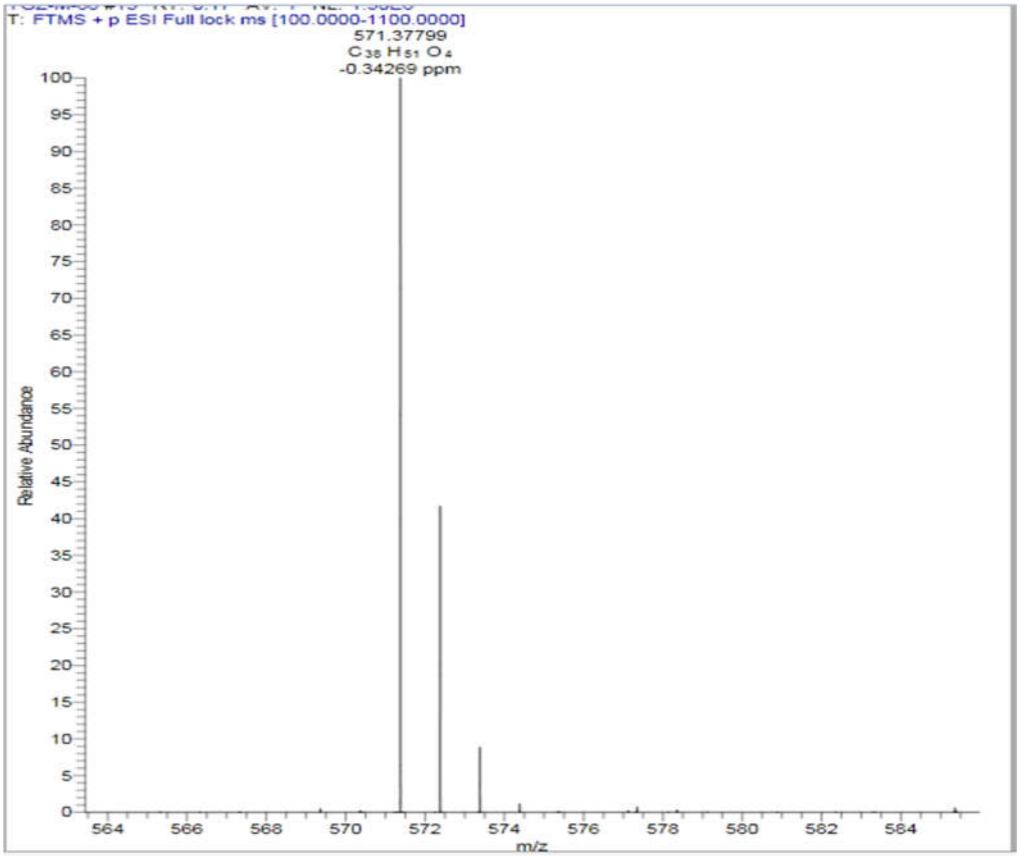

Figure S124: HR-ESI-MS of compound 13

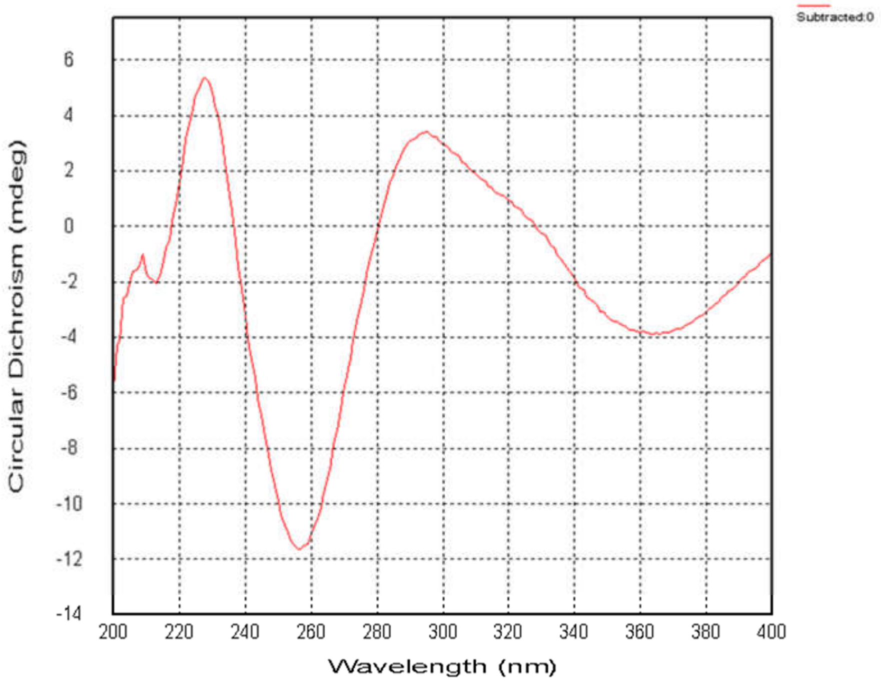

Figure S125: CD spectrum of compound 13

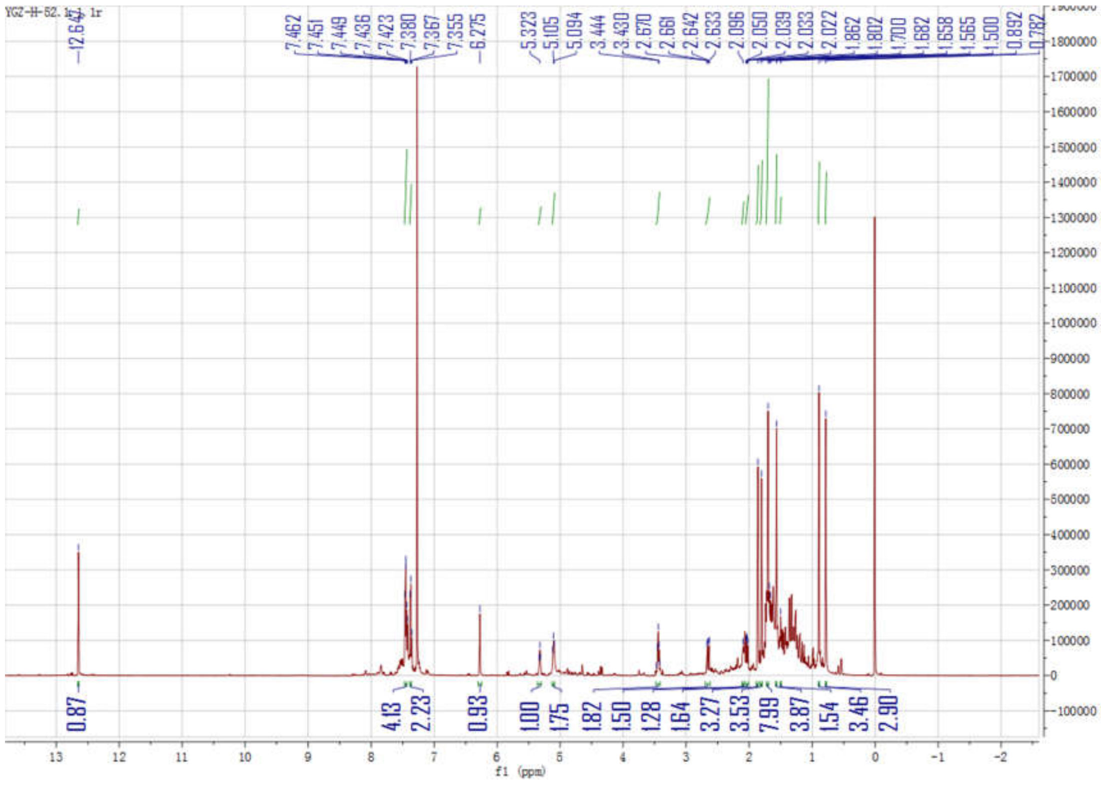

Figure S126: ^{1}H-NMR (600 MHz, $CDCl_3$) spectrum of compound 14

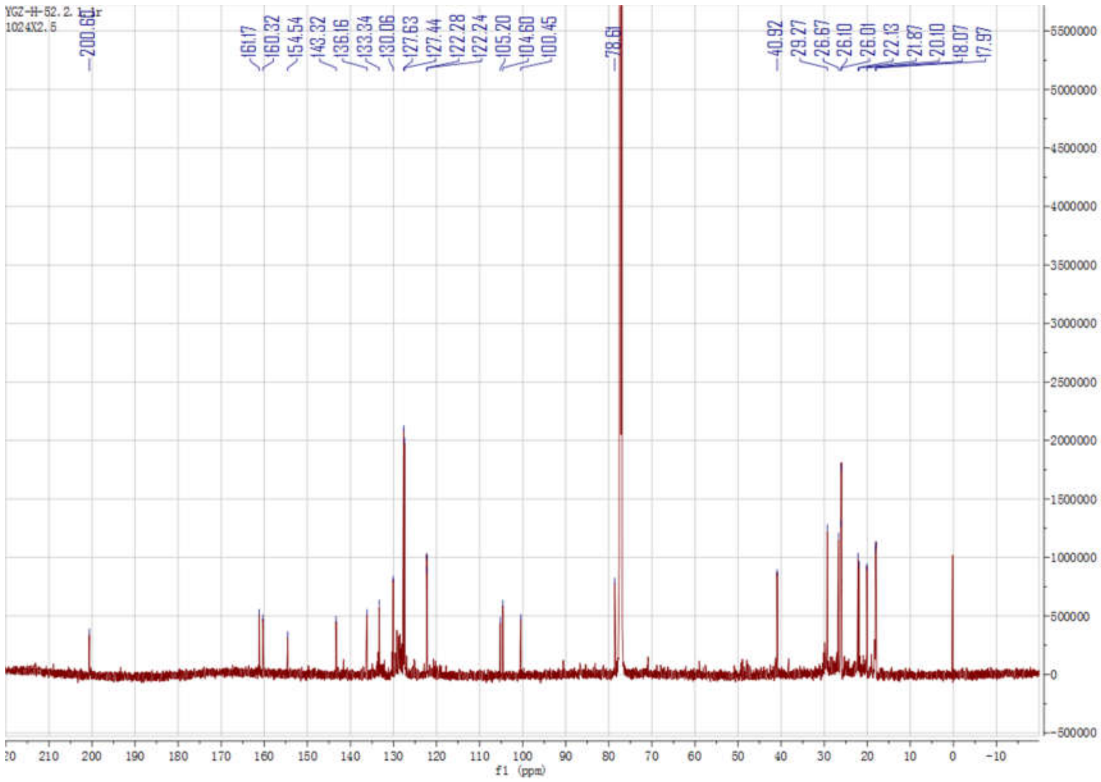

Figure S127: ^{13}C-NMR (150 MHz, CDCl$_3$) spectrum of compound 14

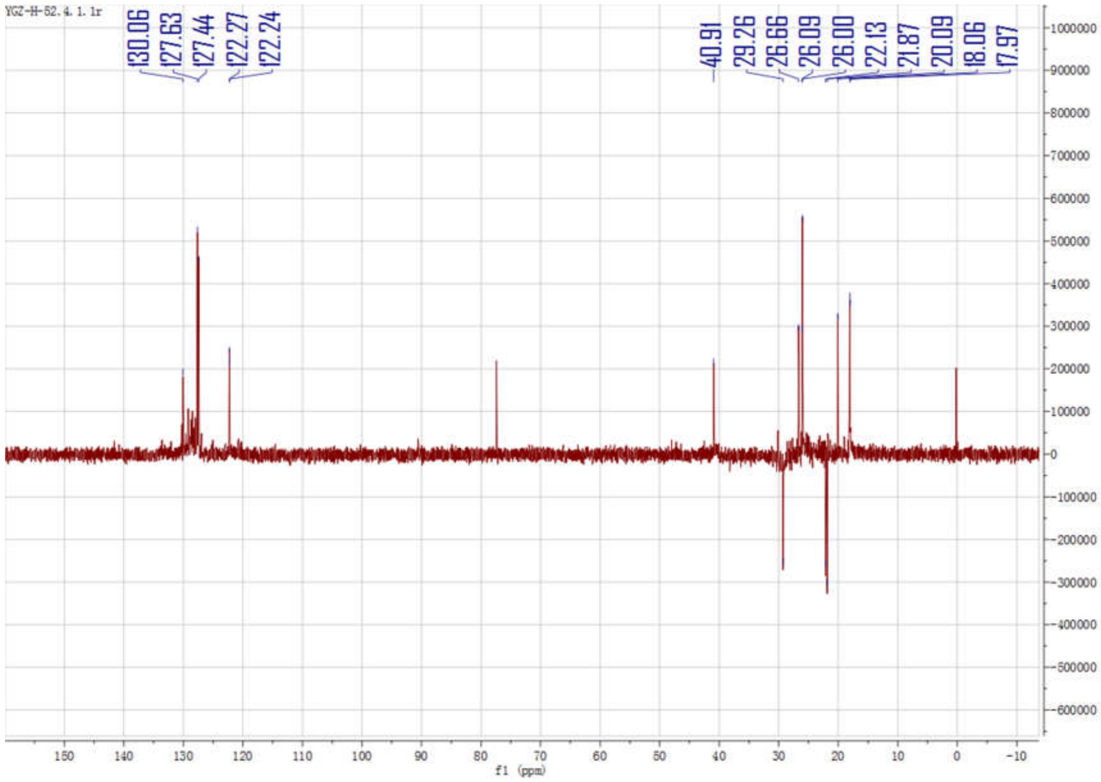

Figure S128: ^{13}C-NMR-DEPT (θ=135°) spectrum of compound 14

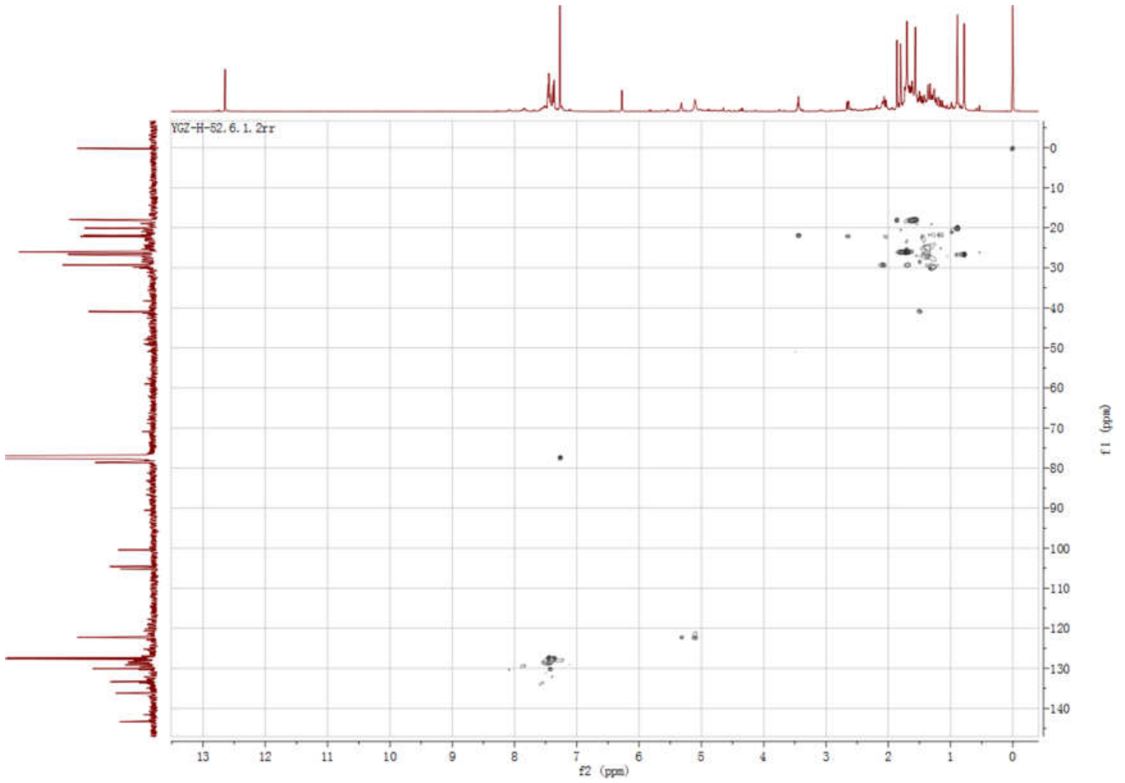

Figure S129: HSQC spectrum of compound 14

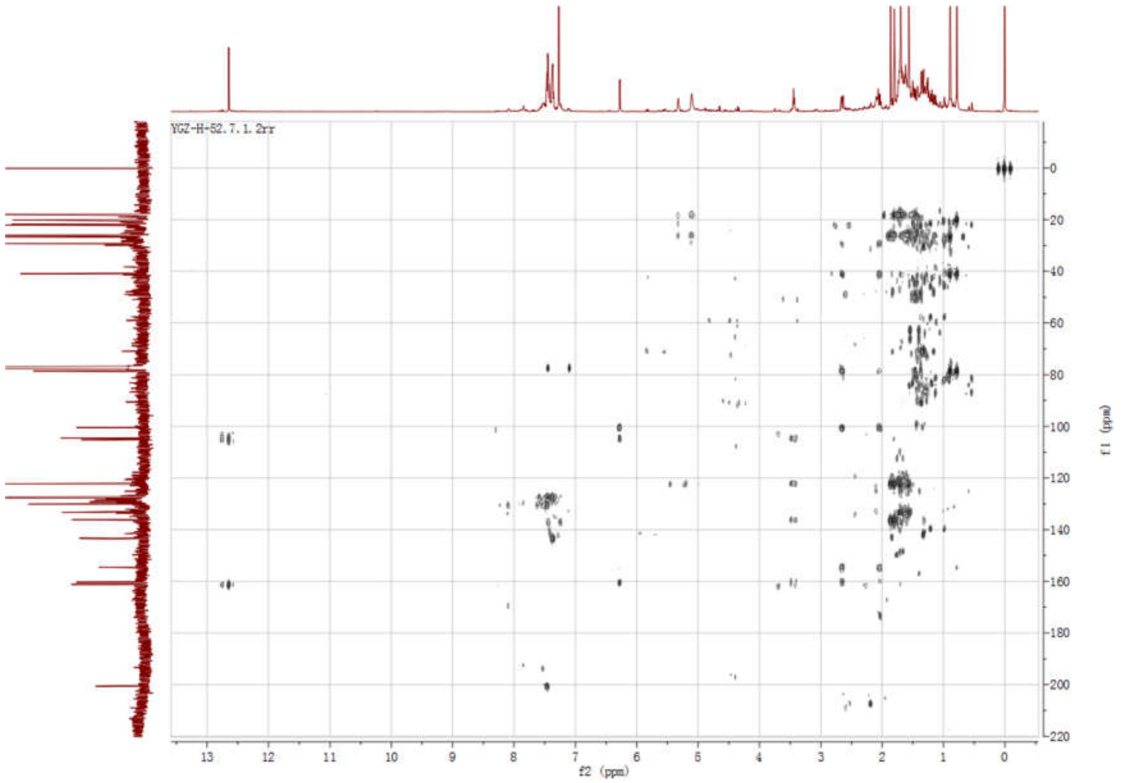

Figure S130: HMBC spectrum of compound 14

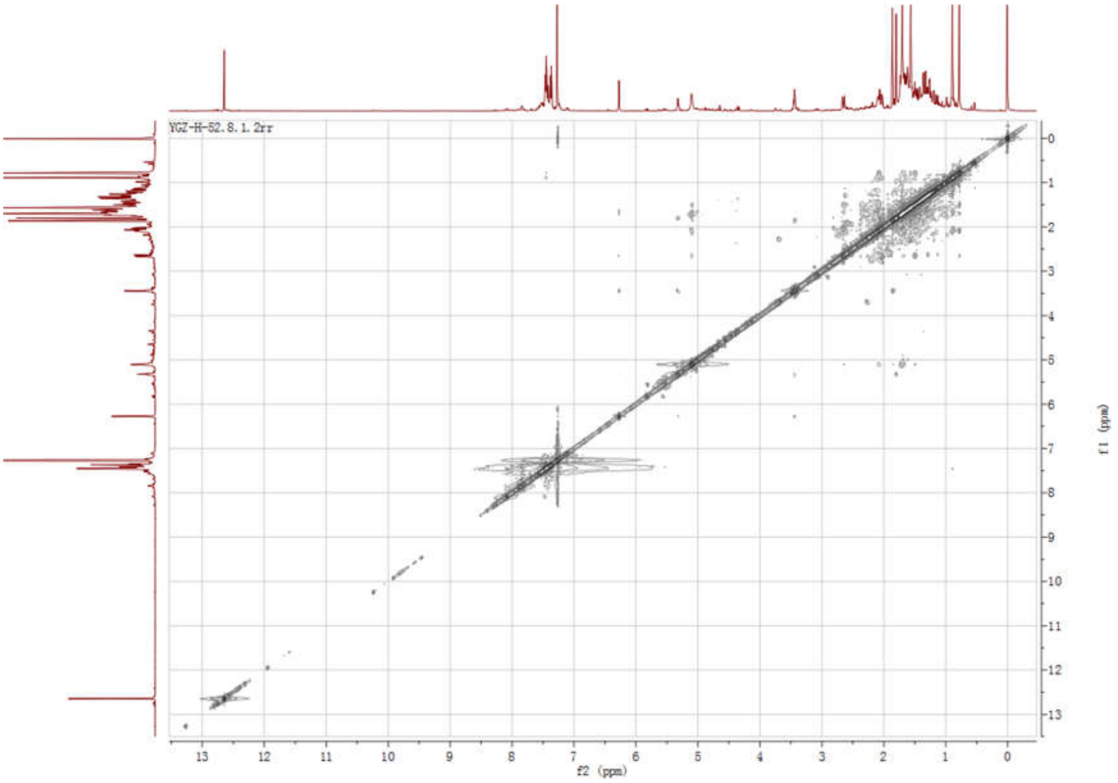

Figure S131: ROESY spectrum of compound 14

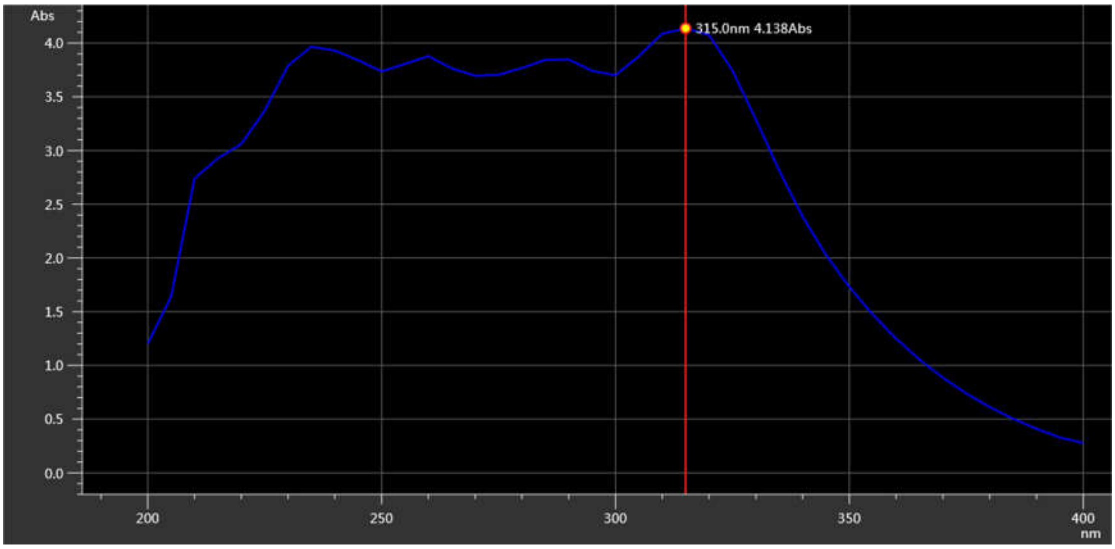

Figure S132: UV spectrum of compound 14

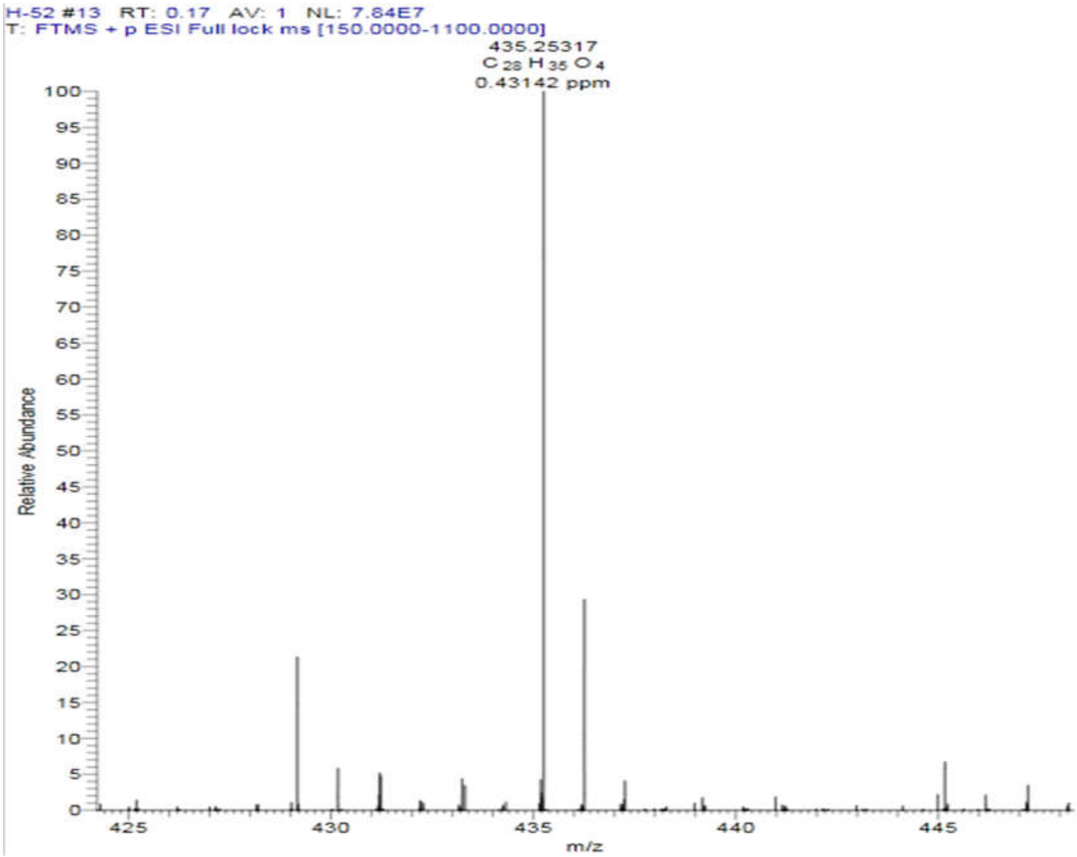

Figure S133: HR-ESI-MS of compound 14

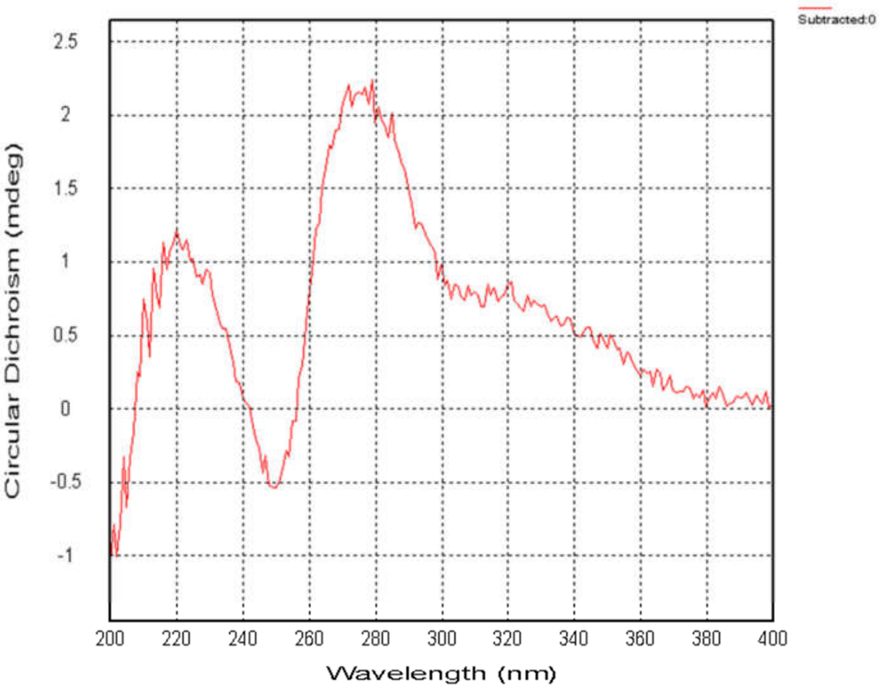

Figure S134: CD spectrum of compound 14

Table S1. Antiproliferative activities of compounds **1-24** (IC_{50}, μM)

No.	T98	HepG2	MCF-7	No.	T98	HepG2	MCF-7
Cisplatin	14.16±0.33	8.5±0.18	17.61±0.48	Cisplatin	14.16±0.33	8.5±0.18	17.61±0.48
**1**	>20	>20	>20	**13**	>20	>20	>20
**2**	13.23±4.24	13.53±0.17	9.81±1.56	**14**	>20	>20	>20
**3**	>20	>20	>20	**15**	>20	>20	>20
**4**	>20	>20	>20	**16**	>20	>20	>20
**5**	>20	>20	>20	**17**	>20	>20	>20
**6**	>20	>20	>20	**18**	>20	>20	>20
**7**	17.00±2.75	12.84±1.59	15.68±1.65	**19**	>20	>20	>20
**8**	>20	>20	>20	**20**	>20	>20	>20
**9**	>20	>20	>20	**21**	>20	>20	>20
**10**	>20	>20	>20	**22**	>20	>20	>20
**11**	>20	>20	>20	**23**	>20	>20	>20
**12**	>20	>20	>20	**24**	>20	>20	>20

BPAPs

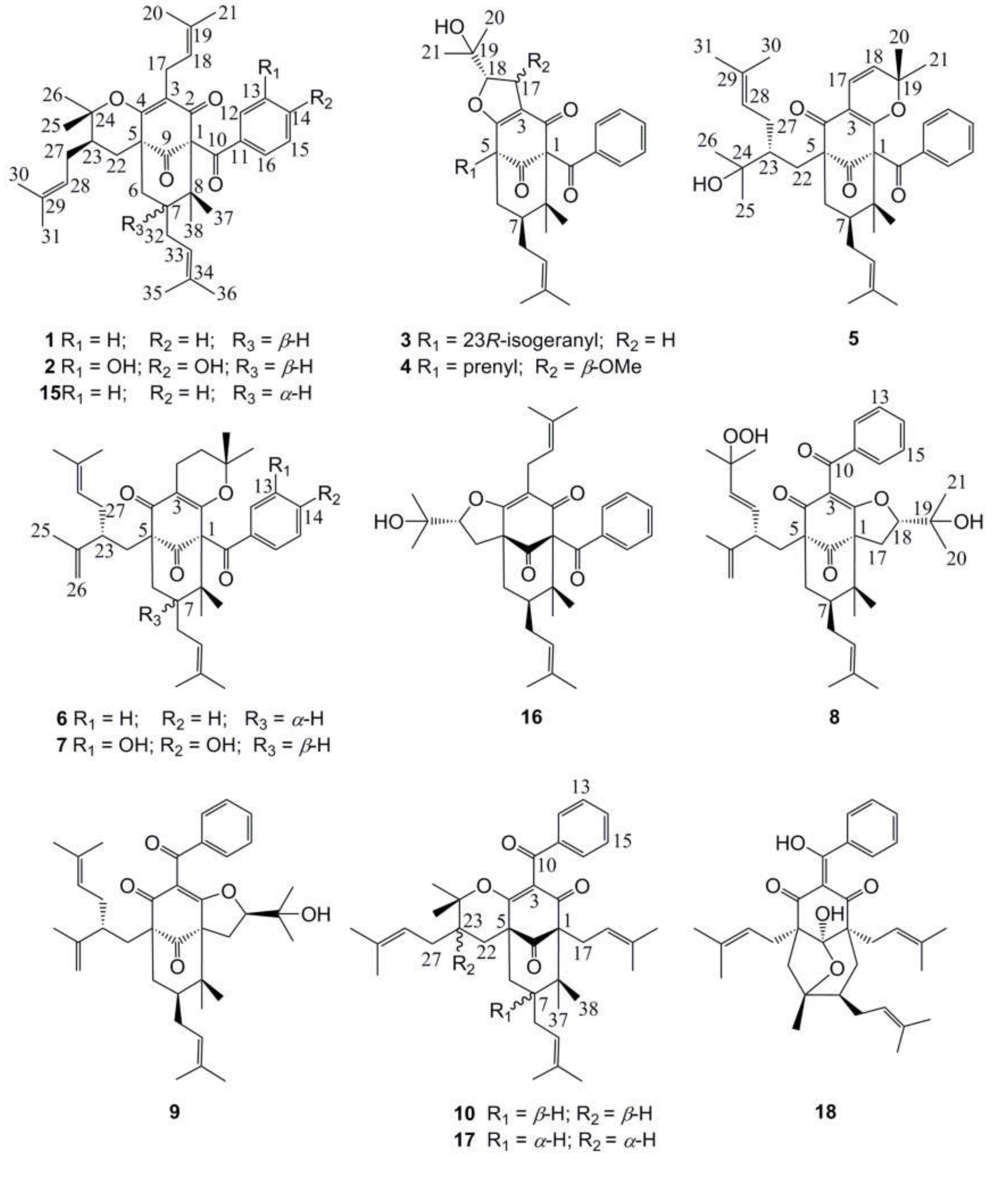

Figure S135 Chemical structures of BPAPs (1-10 and 15-18) from *G. multiflora*.

Caged (**11**,**19**-**22**), Complicated (**23**,**24**) PPAPs

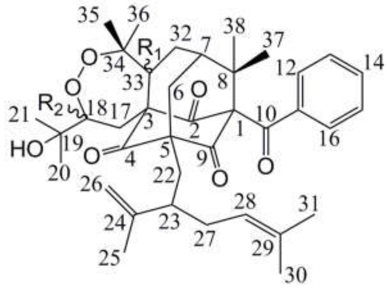

11 R_1 = *β*-H R_2 = *β*-H
19 R_1 = *α*-H R_2 = *α*-H

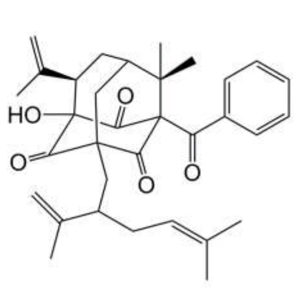

20

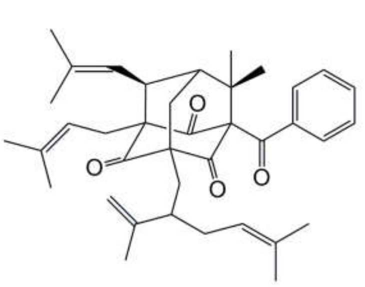

21

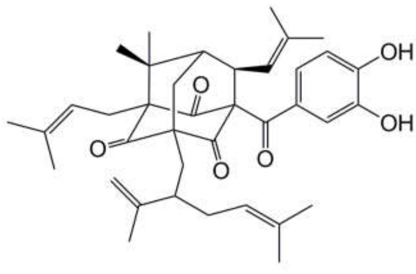

22

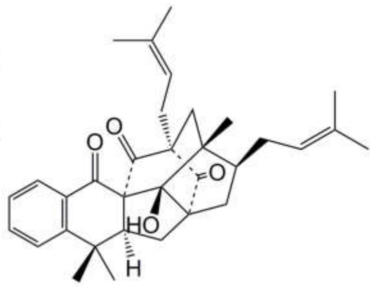

23

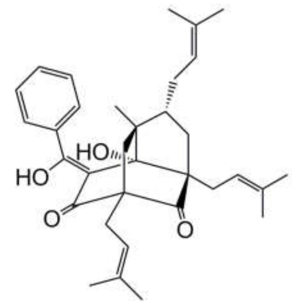

24

MPAPs

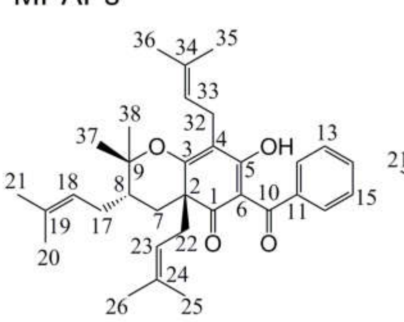

12

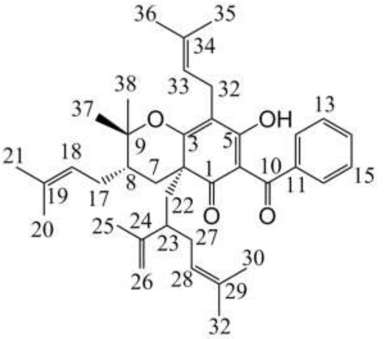

13

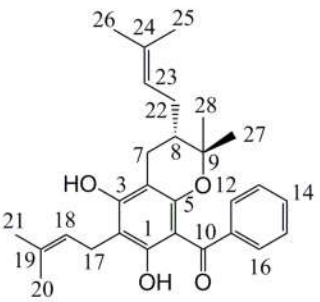

14

Figure S136 Chemical structures of PAPs (11-14 and 19-24) from *G. multiflora*.

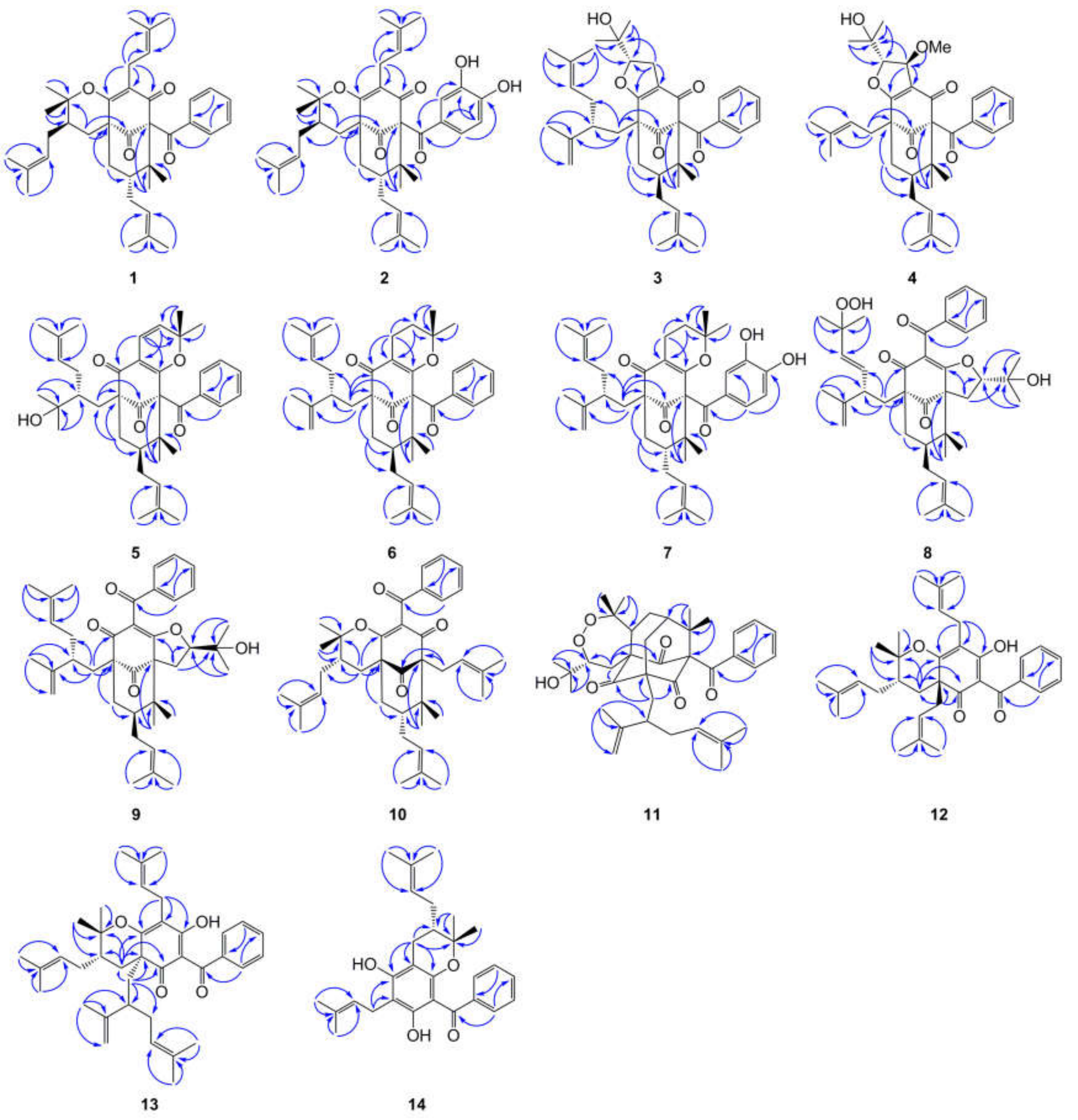

Figure S137 HMBC correlations for compounds 1-14.

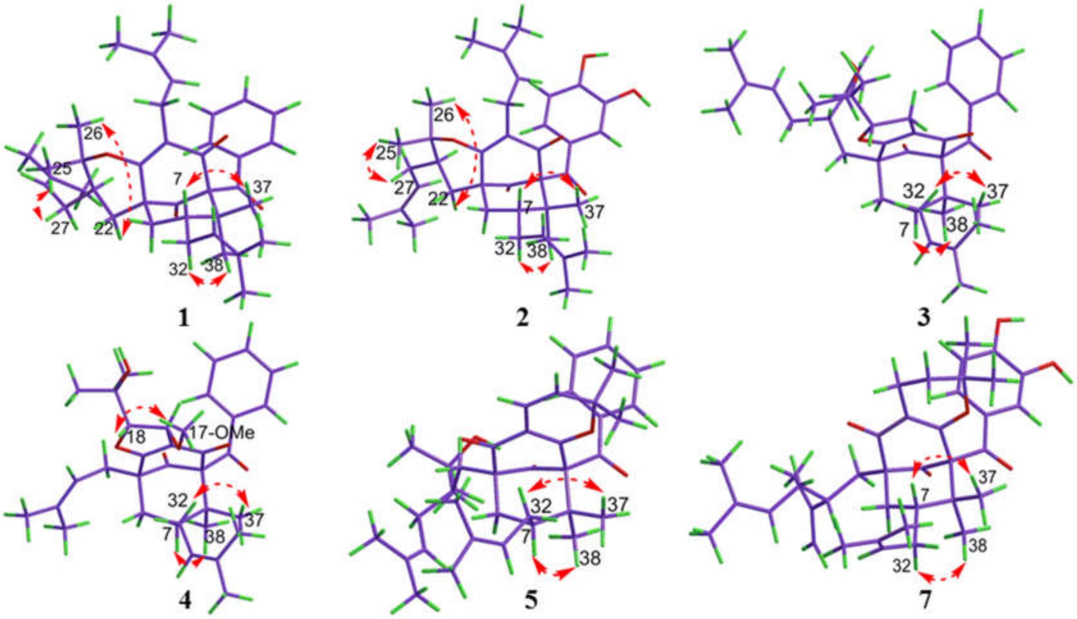

Figure S138 ROESY correlations for compounds 1-5 and 7.

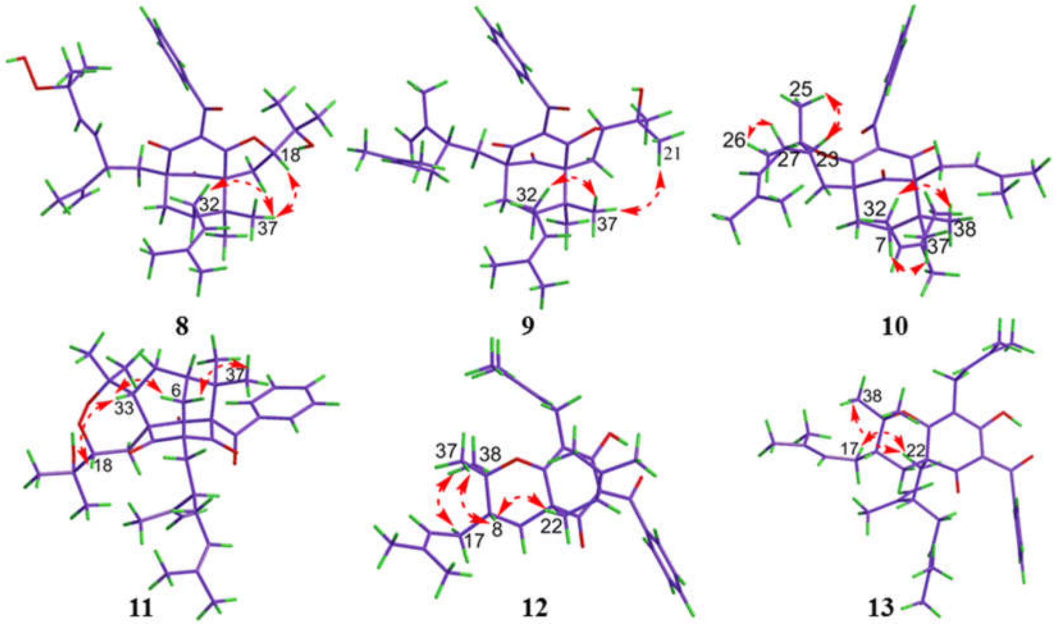

Figure S139 ROESY correlations for compounds 8-13.

1. Computational methods

1. 1 Conformational analysis

Conformational analysis for compounds **1-14** (**Figure S140-S153**) were performed in Yinfo Cloud Platform (http://cloud.yinfotek.com/) using Stochastic or Custom algorithm by Confab [1] at MMFF94 force field with RMSD threshold of 0.5 Å and energy window of 7 kcal/mol.

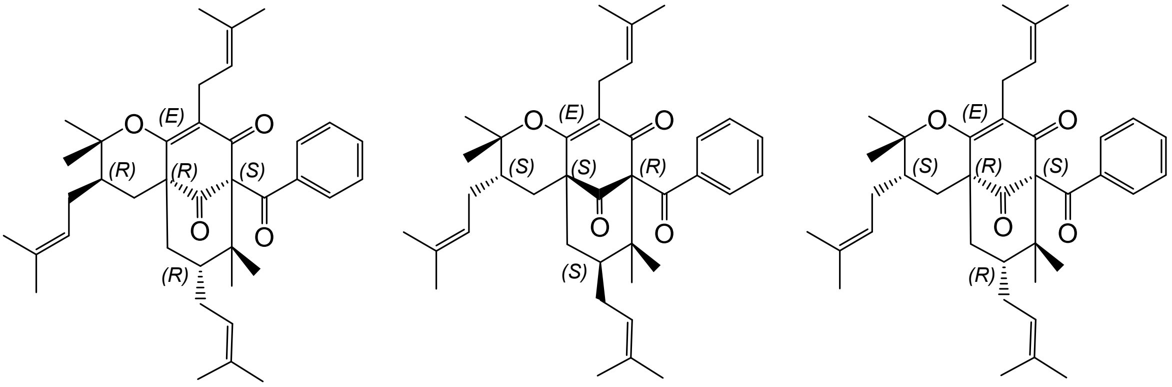

1a **1a'** **1b**

Figure S140 Chemical structure of compounds **1**.

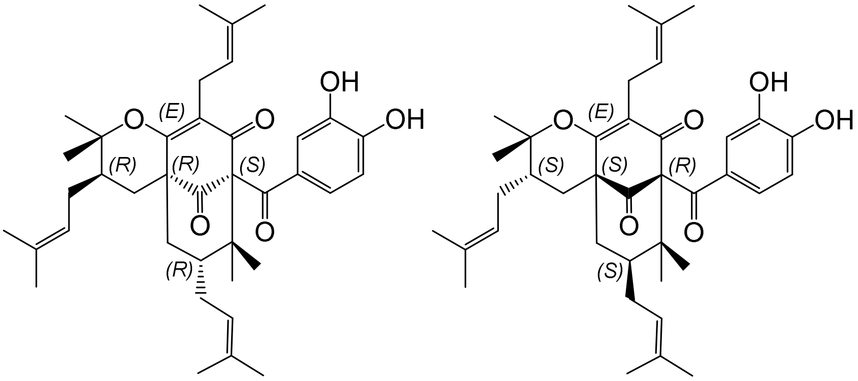

2a **2b**

Figure S141 Chemical structure of compounds **2**.

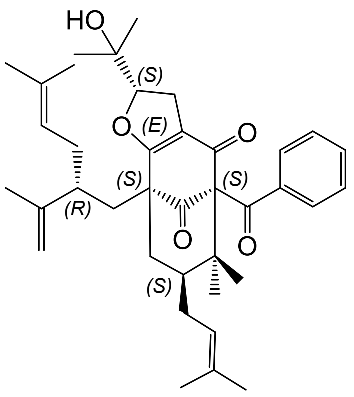

3a

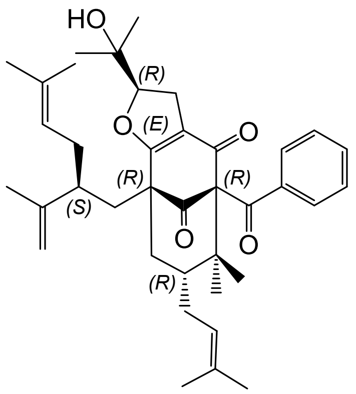

3b

Figure S142 Chemical structure of compounds **3**.

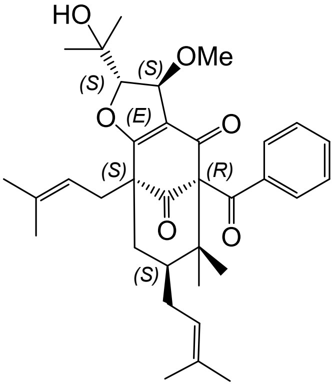

4a

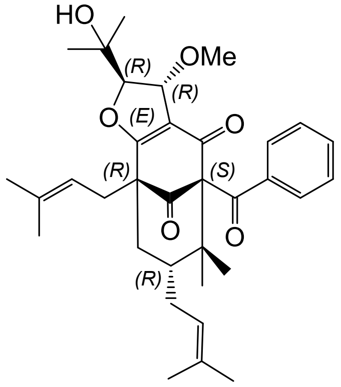

4b

Figure S143 Chemical structure of compounds **4**.

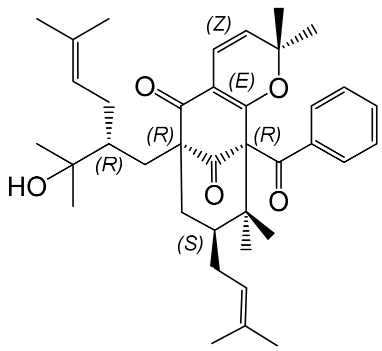

5a

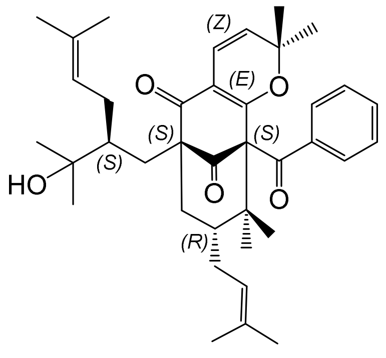

5b

Figure S144 Chemical structure of compounds **5**.

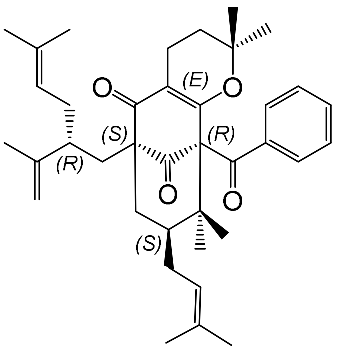

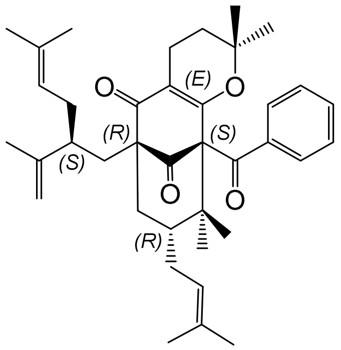

6a **6b**

Figure S145 Chemical structure of compounds **6**.

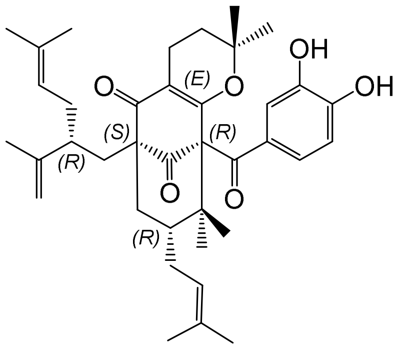

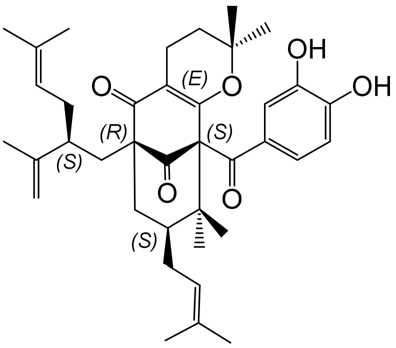

7a **7b**

Figure S146 Chemical structure of compounds **7**.

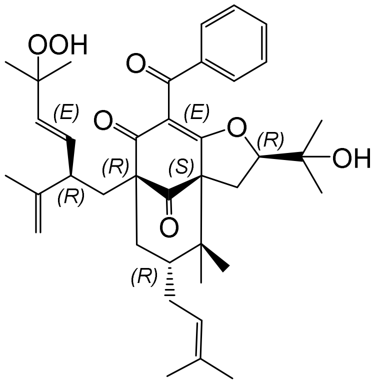

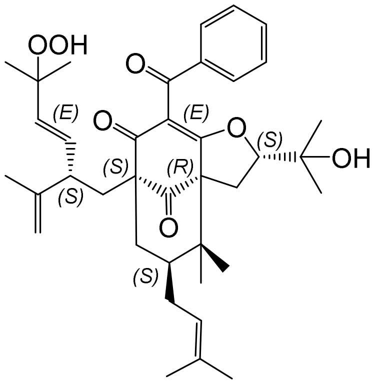

8a **8b**

Figure S147 Chemical structure of compounds **8**.

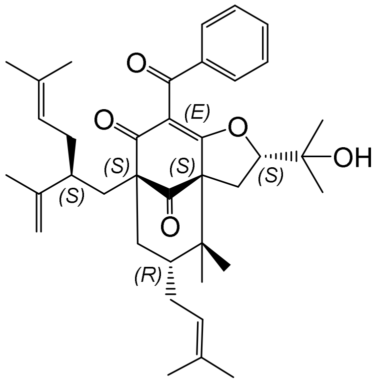

9a

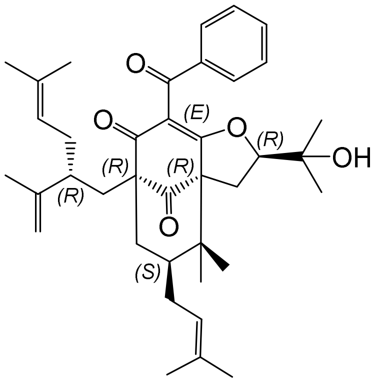

9b

Figure S148 Chemical structure of compounds **9**.

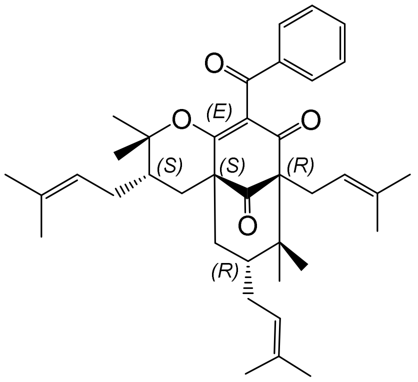

10a

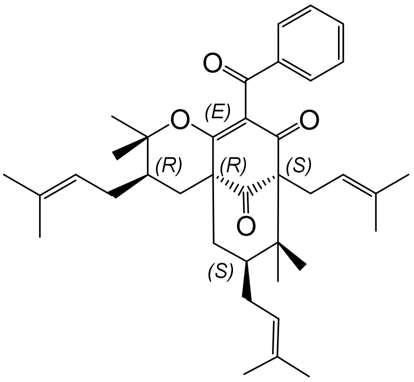

10b

Figure S149 Chemical structure of compounds **10**.

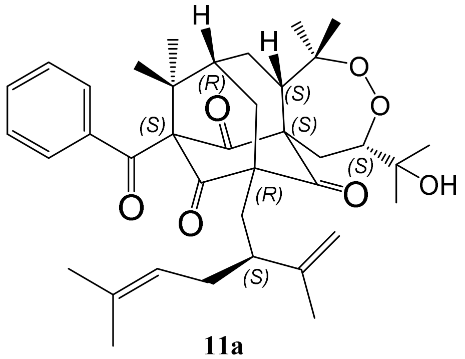

11a

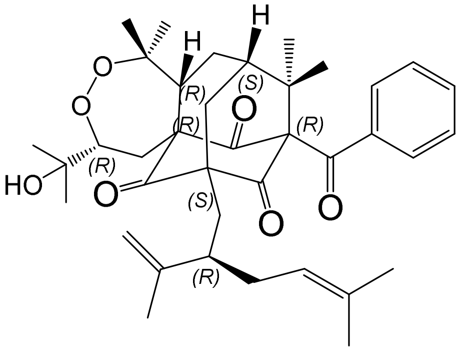

11a'

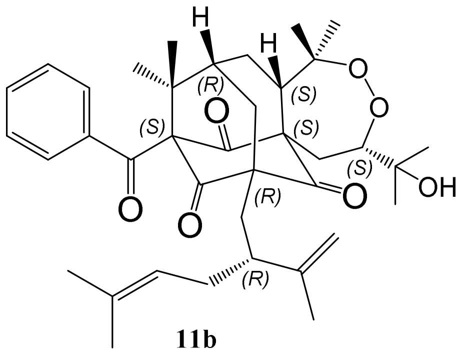

11b

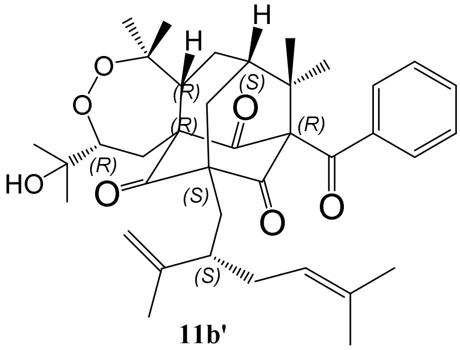

11b'

Figure S150 Chemical structure of compounds **11**.

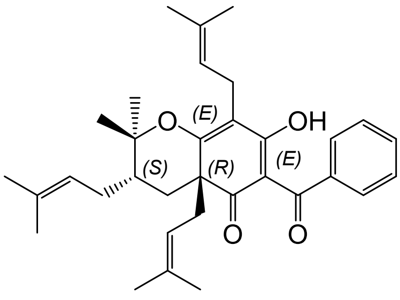

12a

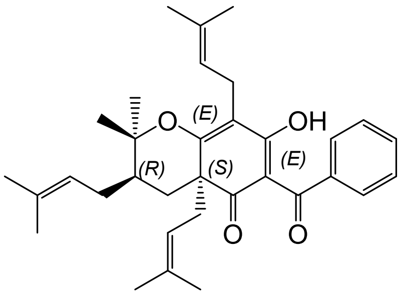

12b

Figure S151 Chemical structure of compounds **12**.

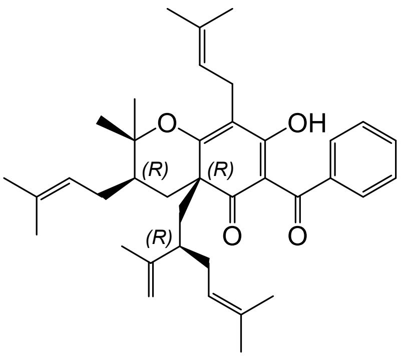

13a

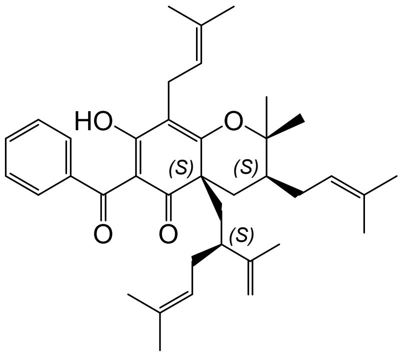

13a'

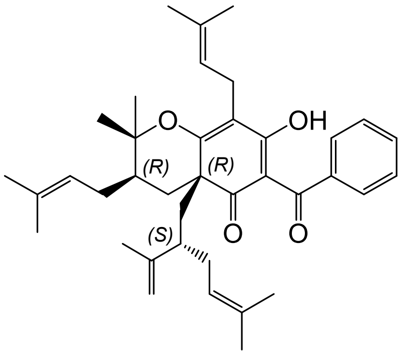

13b

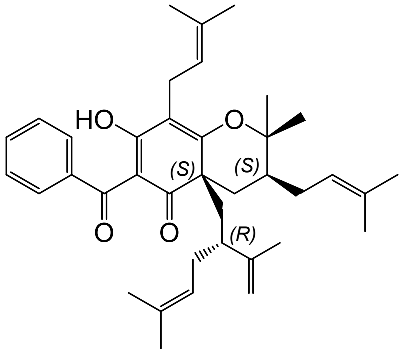

13b'

Figure S152 Chemical structure of compounds **13**.

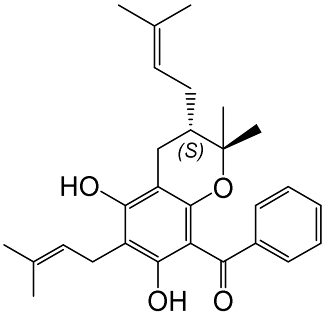

14a

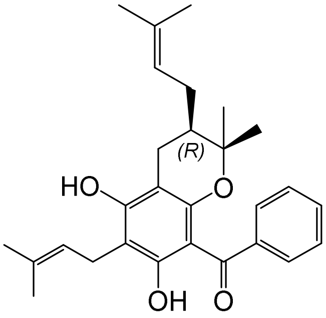

14b

Figure S153 Chemical structure of compounds **14**.

1. 2**NMR calculation**

The theoretical calculations were carried out using Gaussian 09 [2]. At first, all conformers were optimized at PM6. Room-temperature equilibrium populations were calculated according to Boltzmann distribution law (eq. 1), based on which

dominative conformers of population over 1% were kept. The chosen conformers were further optimized at B3LYP/6-31G(d,p) in gas phase (**Table S2**). Vibrational frequency analysis confirmed the stable structures. NMR calculations were carried out following the protocol adapted from Michael *et al.* [3] (**Table S3**) using the Gauge-Including Atomic Orbitals (GIAO) method at mPW1PW91/6-311+G(2d,p) level in $CDCl_3$ simulated by the IEFPCM model. The TMS-corrected NMR chemical shift values were averaged according to Boltzmann distribution and fitted to the experimental values by linear regression. The calculated ^{13}C- and ^{1}H-NMR chemical shift values of TMS in $CDCl_3$ were 77.2 and 7.27 ppm respectively. To confirm the conclusions of NMR calculations, DP4+ analysis was also performed.

$$\frac{N_i}{N} = \frac{g_i e^{-\frac{E_i}{k_B T}}}{\sum g_i e^{-\frac{E_i}{k_B T}}} \qquad \textbf{(1)}$$

where N_i is the number of conformer i with energy E_i and degeneracy g_i at temperature T, and k_B is Boltzmann constant.

1. 3 ECD calculation

The ECD calculations were conducted at B3LYP/6-311G(d,p) level in methanol with IEFPCM model using Time-dependent Density functional theory (TD-DFT). Rotatory strengths for 30 excited states were calculated. The ECD spectrum was simulated using the ECD/UV analysis tool in Yinfo Cloud Platform (https://cloud.yinfotek.com/) by overlapping Gaussian functions for each transition according to (eq. 2).

$$\Delta\varepsilon(E) = \frac{1}{2.297\times10^{-39}} \times \frac{1}{\sqrt{2\pi\sigma}} \sum_{i}^{A} \Delta E_i R_i e^{-\left(\frac{E-E_i}{2\sigma}\right)^2} \qquad \textbf{(2)}$$

where σ represents the width of the band at $1/e$ height, while ΔE_i and R_i are the excitation energies and rotatory strengths for transition *i*, respectively.

The spectrum of the enantiomers were produced directly by mirror inversion about the horizontal axis.

1. 4 References

1. Noel M OBoyle, Tim V, ermeersch, Christopher J Flynn, Anita R Maguire Maguire, and Geoffey R Hutchison. Confab-systematic generation of diverse low-energy conformers. *Journal of Cheminformatics*, 3:3–8, March 2011.
2. M. J. Frisch, G. W. Trucks, H. B. Schlegel, G. E. Scuseria, M. A. Robb, J. R. Cheeseman, G. Scalmani, V. Barone, B. Mennucci, G. A. Petersson, H. Nakatsuji, M. Caricato, X. Li, H. P. Hratchian, A. F. Izmaylov, J. Bloino, G. Zheng, J. L. Sonnenberg, M. Hada, M. Ehara, K. Toyota, R. Fukuda, J. Hasegawa, M. Ishida, T. Nakajima, Y. Honda, O. Kitao, H. Nakai, T. Vreven, J. A. Montgomery, Jr., J. E. Peralta, F. Ogliaro, M. Bearpark, J. J. Heyd, E. Brothers, K. N. Kudin, V. N. Staroverov, R. Kobayashi, J. Normand, K. Raghavachari, A. Rendell, J. C. Burant, S. S. Iyengar, J. Tomasi, M. Cossi, N. Rega, J. M. Millam, M. Klene, J. E. Knox, J. B. Cross, V. Bakken, C. Adamo, J. Jaramillo, R. Gomperts, R. E. Stratmann, O. Yazyev, A. J. Austin, R. Cammi, C. Pomelli, J. W. Ochterski, R. L. Martin, K. Morokuma, V. G. Zakrzewski, G. A. Voth, P. Salvador, J. J. Dannenberg, S. Dapprich, A. D. Daniels, O. Farkas, J. B. Foresman, J. V. Ortiz, J. Cioslowski, and D. J. Fox. Gaussian 09 Revision D.01. Gaussian Inc. Wallingford CT 2009.
3. Michael W. Lodewyk, Matthew R. Siebert, and Dean J. Tantillo. Computational Prediction of 1H and 13C Chemical Shifts: A Useful Tool for Natural Product, Mechanistic, and Synthetic Organic Chemistry. *Chem. Rev.*, 2012, 112 (3), 1839–1862.

2. Energies and Coordinates

2. 1 Energies at B3LYP theory level

Table S2 Energies of configurations **1-14**.

Configuration	Conformer	Structure	E (Hartree)	E (kcal/mol)	Population (%)
1a	8	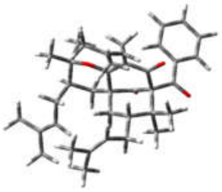	-1779.475857	-1116637.95	66.31
1a	9	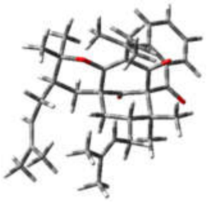	-1779.474872	-1116637.332	23.34
1a	10	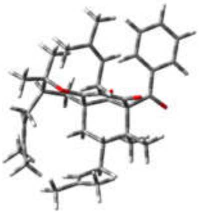	-1779.474106	-1116636.851	10.36
1b	2	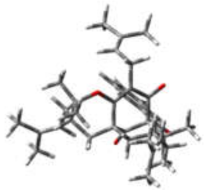	-1779.082633	-1116391.198	82.96
1b	3	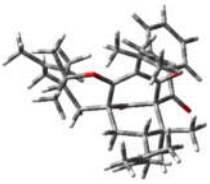	-1779.081138	-1116390.260	17.04
2a	12	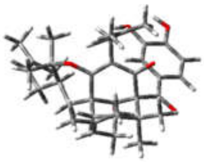	-1929.970863	-1211074.991	82.60

2a	13	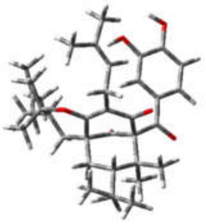	-1929.969394	-1211074.069	17.40
3a	1	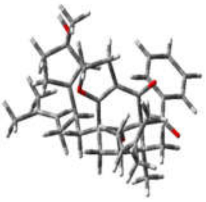	-1854.701818	-1163842.953	100.00
4a	2	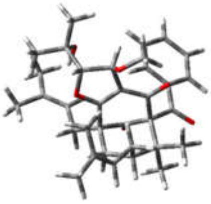	-1773.876498	-1113124.299	85.41
4a	4	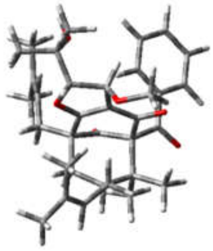	-1773.874829	-1113123.252	14.59
5a	2	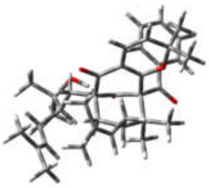	-1854.712748	-1163849.812	14.69
5a	3	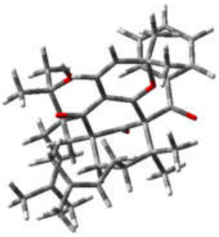	-1854.714409	-1163850.854	85.31
6a	1	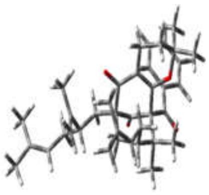	-1779.465762	-1116631.615	100.00

7a	1	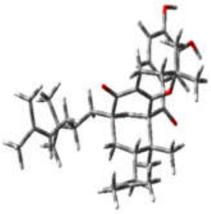	-1929.969744	-1211074.289	23.90
7a	2	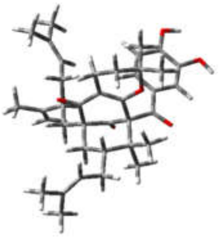	-1929.974672	-1211077.382	76.10
8a	1	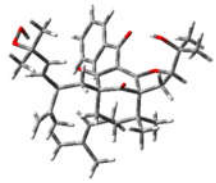	-2004.6851	-1257958.883	29.41
8a	2	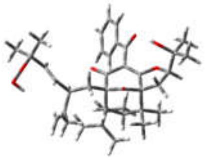	-2004.685927	-1257959.402	70.59
9a	1	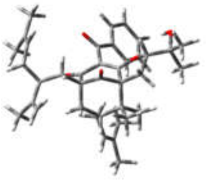	-1854.701499	-1163842.753	1.60
9a	3	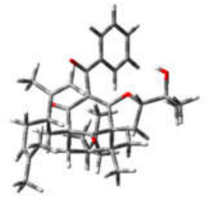	-1854.705390	-1163845.194	98.40
10a	1	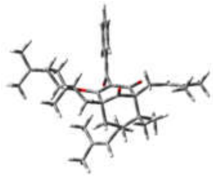	-1779.470384	-1116634.516	87.21
10a	3	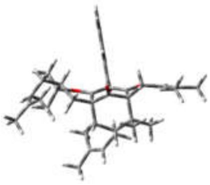	-1779.468559	-1116633.37	12.60

11a	1	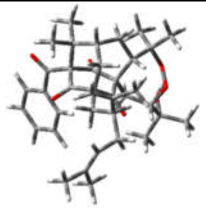	-2005.109059	-1258224.921	100
11b	1	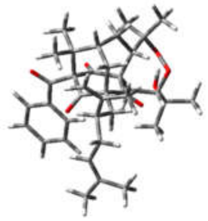	-2005.109768	-1258225.366	42.75
11b	3	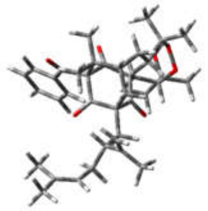	-2005.109614	-1258225.269	36.30
11b	11	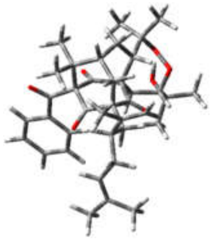	-2005.109095	-1258224.944	20.95
12a	2	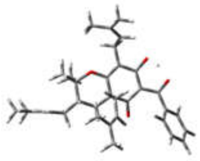	-1583.750963	-993818.7261	53.84
12a	3	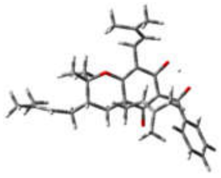	-1583.749786	-993817.987	15.45
12a	6	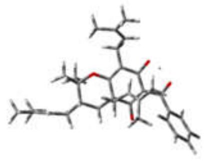	-1583.749786	-993817.9869	15.55
13a	1	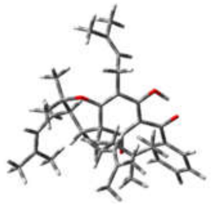	-1779.468982	-1116633.636	61.54

13a	2	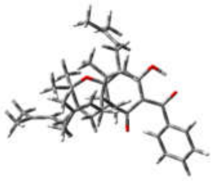	-1779.468538	-1116633.357	38.46
13b	1	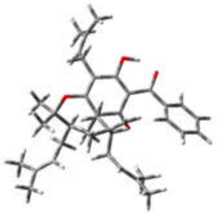	-1779.469034	-1116633.669	100
14a	3	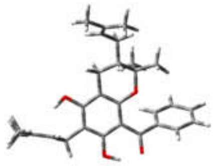	-1388.724283	-871437.6373	84.53
14a	4	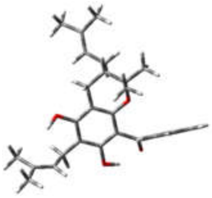	-1388.722679	-871436.6310	15.47

2. 2Coordinates at B3LYP theory level

Table S3 Standard orientations of configurations **1-14**.

Conformer 1a-8

Center	Atomic	Atomic	Coordinates (Angstroms)		
Number	Number	Type	X	Y	Z
1	6	0	0.157979	-0.863082	-0.090520
2	6	0	-1.956511	0.085777	-1.179634
3	6	0	-2.099971	1.021617	0.045091
4	6	0	-1.166988	0.915254	1.162102
5	6	0	-0.145657	0.008973	1.120942
6	6	0	-1.149355	-1.154076	-0.826529
7	8	0	-1.476679	-2.272369	-1.182925
8	6	0	1.005279	-0.027928	-1.116007
9	1	0	1.926004	0.304531	-0.633823

10	1	0	1.302143	-0.715890	-1.916048
11	6	0	-1.105934	0.850383	-2.334295
12	6	0	0.297715	1.195007	-1.720911
13	6	0	1.271076	1.896585	-2.709932
14	1	0	1.606179	1.185790	-3.470043
15	1	0	0.727976	2.686286	-3.241971
16	6	0	2.443095	2.531111	-2.005556
17	1	0	2.165037	3.297589	-1.279706
18	6	0	3.754094	2.283232	-2.161341
19	6	0	4.782325	3.051913	-1.364742
20	1	0	4.318589	3.769602	-0.682539
21	1	0	5.463082	3.601245	-2.028526
22	1	0	5.410608	2.372745	-0.773674
23	6	0	4.337260	1.262501	-3.107878
24	1	0	3.584343	0.723036	-3.685034
25	1	0	4.931063	0.523518	-2.554482
26	1	0	5.024502	1.742548	-3.816473
27	6	0	-1.822825	2.152295	-2.749304
28	1	0	-2.852010	1.954672	-3.045071
29	1	0	-1.313379	2.595921	-3.608906
30	1	0	-1.828716	2.888551	-1.942236
31	6	0	-0.951148	-0.057792	-3.575628
32	1	0	-0.350032	0.457155	-4.329837
33	1	0	-1.923267	-0.277805	-4.013484
34	1	0	-0.458383	-1.007685	-3.352143
35	6	0	-3.398339	-0.336821	-1.611779
36	6	0	0.860909	-2.181731	0.300572
37	1	0	0.091242	-2.894310	0.605756
38	1	0	1.319558	-2.615895	-0.591058
39	6	0	1.910404	-2.064041	1.425699
40	1	0	2.099780	-3.087364	1.773835
41	8	0	-3.804301	-0.127791	-2.744558
42	6	0	-4.316720	-1.015193	-0.628815
43	6	0	-3.970313	-1.361588	0.686957
44	1	0	-2.985621	-1.145350	1.082181
45	6	0	-4.891823	-1.992088	1.521829
46	1	0	-4.605495	-2.251988	2.536105
47	6	0	-6.174645	-2.287590	1.057182
48	1	0	-6.890547	-2.778103	1.709787
49	6	0	-6.532749	-1.948775	-0.250615

50	1	0	-7.528673	-2.174494	-0.619304
51	6	0	-5.612179	-1.319931	-1.083219
52	1	0	-5.878572	-1.052655	-2.099264
53	8	0	-3.038995	1.821252	0.054207
54	6	0	-0.889951	3.250282	2.062216
55	1	0	-1.434916	3.764746	1.271592
56	6	0	-1.385745	1.847715	2.339937
57	1	0	-2.465286	1.893823	2.527725
58	1	0	-0.911608	1.423840	3.224848
59	6	0	3.292754	-1.503319	0.984557
60	1	0	3.219779	-0.422894	0.813479
61	1	0	3.984076	-1.623834	1.821755
62	6	0	1.290035	-1.308550	2.624826
63	8	0	0.713843	-0.026119	2.157294
64	6	0	3.867805	-2.162224	-0.245032
65	1	0	3.520896	-1.766717	-1.198908
66	6	0	4.752391	-3.172765	-0.292602
67	6	0	5.236725	-3.714577	-1.616369
68	1	0	4.782430	-3.193012	-2.463255
69	1	0	5.009137	-4.784427	-1.710737
70	1	0	6.327496	-3.622917	-1.702530
71	6	0	5.342226	-3.857256	0.916113
72	1	0	4.994863	-3.442291	1.864093
73	1	0	6.437968	-3.794655	0.898391
74	1	0	5.095304	-4.926688	0.907955
75	6	0	0.113191	3.910524	2.662277
76	6	0	0.976290	3.354885	3.768543
77	1	0	2.037982	3.453030	3.508235
78	1	0	0.781685	2.302962	3.980913
79	1	0	0.833637	3.925510	4.695793
80	6	0	0.453865	5.324823	2.254013
81	1	0	-0.203293	5.691941	1.460824
82	1	0	1.490879	5.393232	1.899367
83	1	0	0.374635	6.010888	3.107786
84	6	0	0.158840	-2.105552	3.289993
85	1	0	0.559931	-3.023345	3.729625
86	1	0	-0.628396	-2.384815	2.586089
87	1	0	-0.293642	-1.511391	4.088287
88	6	0	2.301542	-0.877630	3.685913
89	1	0	3.025610	-0.164798	3.288049

90	1	0	2.841531	-1.750508	4.063784
91	1	0	1.783235	-0.406690	4.525369
92	1	0	0.120036	1.915357	-0.913030

Conformer 1a-9

Center	Atomic	Atomic	Coordinates (Angstroms)		
Number	Number	Type	X	Y	Z
1	6	0	0.024426	-0.976874	0.288495
2	6	0	-1.813378	-0.078355	-1.253673
3	6	0	-1.938580	1.139791	-0.306738
4	6	0	-1.130179	1.209070	0.907099
5	6	0	-0.253631	0.205356	1.205786
6	6	0	-1.234189	-1.279986	-0.523709
7	8	0	-1.687443	-2.403617	-0.653135
8	6	0	1.086830	-0.521689	-0.778946
9	1	0	1.986348	-0.183780	-0.259798
10	1	0	1.371465	-1.411435	-1.352027
11	6	0	-0.749503	0.273514	-2.429188
12	6	0	0.620117	0.592575	-1.732272
13	6	0	1.766896	0.941424	-2.716161
14	1	0	2.090640	0.045093	-3.253733
15	1	0	1.379828	1.630767	-3.476497
16	6	0	2.933910	1.606596	-2.025841
17	1	0	2.653744	2.313163	-1.242186
18	6	0	4.243650	1.477624	-2.292998
19	6	0	5.270374	2.275681	-1.524077
20	1	0	5.858430	2.912878	-2.197793
21	1	0	5.987949	1.613724	-1.021770
22	1	0	4.808148	2.915790	-0.767545
23	6	0	4.818820	0.588019	-3.367788
24	1	0	4.073232	-0.047106	-3.849768
25	1	0	5.603964	-0.058530	-2.957123
26	1	0	5.295793	1.193773	-4.149787
27	6	0	-1.226601	1.508046	-3.224538
28	1	0	-2.245798	1.372295	-3.582359
29	1	0	-0.588168	1.651459	-4.100133
30	1	0	-1.187657	2.419802	-2.623687
31	6	0	-0.603312	-0.923786	-3.395941
32	1	0	0.132411	-0.680198	-4.167122
33	1	0	-1.549616	-1.137028	-3.890231
34	1	0	-0.268262	-1.837238	-2.897732

35	6	0	-3.250281	-0.423875	-1.764099
36	6	0	0.465332	-2.236250	1.070359
37	1	0	-0.439905	-2.751880	1.399046
38	1	0	0.960586	-2.923248	0.378680
39	6	0	1.380201	-1.992542	2.290875
40	1	0	1.297340	-2.891647	2.915982
41	8	0	-3.507910	-0.444013	-2.957813
42	6	0	-4.347048	-0.744757	-0.782940
43	6	0	-4.190064	-0.784828	0.611481
44	1	0	-3.233332	-0.573377	1.072582
45	6	0	-5.267841	-1.096821	1.438980
46	1	0	-5.127457	-1.122484	2.515015
47	6	0	-6.520013	-1.375103	0.887760
48	1	0	-7.357635	-1.618226	1.534528
49	6	0	-6.690153	-1.339245	-0.498953
50	1	0	-7.660824	-1.554193	-0.935213
51	6	0	-5.613970	-1.027190	-1.323871
52	1	0	-5.733604	-0.996953	-2.400704
53	8	0	-2.753459	2.017939	-0.598819
54	6	0	-0.587141	3.645920	1.237196
55	1	0	-0.988859	4.006913	0.291201
56	6	0	-1.303081	2.432250	1.789466
57	1	0	-2.375737	2.655443	1.838148
58	1	0	-0.965462	2.195069	2.798133
59	6	0	2.884342	-1.871555	1.937731
60	1	0	3.080950	-0.979103	1.338039
61	1	0	3.440049	-1.733305	2.873696
62	6	0	0.810523	-0.841940	3.154694
63	8	0	0.514647	0.333363	2.304216
64	6	0	3.406925	-3.119368	1.269854
65	1	0	3.049246	-4.047941	1.719476
66	6	0	4.272133	-3.231504	0.249462
67	6	0	4.710538	-4.590469	-0.243567
68	1	0	4.236987	-5.401871	0.315951
69	1	0	5.799135	-4.707019	-0.159011
70	1	0	4.468638	-4.719956	-1.306649
71	6	0	4.902579	-2.070677	-0.478291
72	1	0	4.521809	-1.098774	-0.160390
73	1	0	4.742399	-2.160582	-1.559882
74	1	0	5.990235	-2.070587	-0.327145

75	6	0	0.454267	4.307201	1.766993
76	6	0	1.138526	3.949220	3.063377
77	1	0	2.217864	3.829641	2.903973
78	1	0	0.763969	3.026992	3.508949
79	1	0	1.024229	4.757584	3.797591
80	6	0	1.031573	5.517022	1.069783
81	1	0	0.499437	5.750058	0.143326
82	1	0	2.091204	5.362524	0.826618
83	1	0	0.990558	6.401954	1.718707
84	6	0	-0.500515	-1.247820	3.843451
85	1	0	-0.308795	-2.043384	4.568943
86	1	0	-1.248907	-1.610435	3.134684
87	1	0	-0.921868	-0.390874	4.375666
88	6	0	1.785404	-0.290288	4.192569
89	1	0	2.656579	0.168492	3.721547
90	1	0	2.125863	-1.093371	4.852348
91	1	0	1.287505	0.467138	4.803785
92	1	0	0.461358	1.498264	-1.134824

Conformer 1a-10

Center	Atomic	Atomic	Coordinates (Angstroms)		
Number	Number	Type	X	Y	Z
1	6	0	-0.513356	0.089962	-0.895666
2	6	0	1.116958	-1.745341	-0.170196
3	6	0	1.504316	-0.852596	1.033003
4	6	0	0.917389	0.475769	1.176159
5	6	0	0.027669	0.935416	0.247805
6	6	0	0.558108	-0.915595	-1.313973
7	8	0	0.875639	-1.105082	-2.475083
8	6	0	-1.693108	-0.789584	-0.338978
9	1	0	-2.447268	-0.125347	0.087795
10	1	0	-2.158603	-1.282912	-1.199884
11	6	0	-0.091480	-2.738644	0.273000
12	6	0	-1.295453	-1.833786	0.719489
13	6	0	-2.555611	-2.636583	1.156968
14	1	0	-2.994257	-3.127820	0.280393
15	1	0	-2.250367	-3.434481	1.836944
16	6	0	-3.619554	-1.785397	1.810554
17	1	0	-4.316674	-1.299301	1.129259
18	6	0	-3.797996	-1.566871	3.124172
19	6	0	-4.922156	-0.687223	3.617206

20	1	0	-5.520953	-0.287624	2.794095
21	1	0	-4.533762	0.158143	4.200281
22	1	0	-5.590236	-1.243006	4.288325
23	6	0	-2.935166	-2.150581	4.216052
24	1	0	-3.543614	-2.736194	4.917336
25	1	0	-2.470842	-1.349967	4.806113
26	1	0	-2.136753	-2.793367	3.841251
27	6	0	0.358193	-3.621868	1.456919
28	1	0	1.284826	-4.144495	1.225455
29	1	0	-0.402423	-4.379527	1.663128
30	1	0	0.508787	-3.035422	2.366566
31	6	0	-0.495783	-3.646694	-0.910264
32	1	0	-1.306425	-4.311159	-0.599526
33	1	0	0.343724	-4.265649	-1.221885
34	1	0	-0.845364	-3.085842	-1.780985
35	6	0	2.415531	-2.476977	-0.643732
36	6	0	-0.940036	0.946985	-2.109795
37	1	0	-0.049545	1.127120	-2.716263
38	1	0	-1.607204	0.351423	-2.738685
39	6	0	-1.612977	2.298229	-1.782480
40	1	0	-1.527737	2.907992	-2.691793
41	8	0	2.474083	-3.696060	-0.688085
42	6	0	3.625097	-1.680312	-1.057462
43	6	0	4.764495	-2.413521	-1.433478
44	1	0	4.714556	-3.495837	-1.403606
45	6	0	5.929897	-1.767749	-1.834919
46	1	0	6.799822	-2.351132	-2.120638
47	6	0	5.978741	-0.371504	-1.869223
48	1	0	6.886482	0.135542	-2.182133
49	6	0	4.854917	0.369738	-1.499150
50	1	0	4.884126	1.454516	-1.523605
51	6	0	3.687660	-0.278369	-1.096984
52	1	0	2.836444	0.327997	-0.813796
53	8	0	2.334974	-1.289062	1.833496
54	6	0	2.657759	2.048178	2.099743
55	1	0	3.468798	1.411913	1.746059
56	6	0	1.363381	1.312187	2.363122
57	1	0	0.563389	1.998341	2.641106
58	1	0	1.515128	0.623056	3.202993
59	6	0	-3.131790	2.197548	-1.489257

60	1	0	-3.316952	1.671306	-0.549198
61	1	0	-3.511968	3.216525	-1.342365
62	6	0	-0.789526	3.047284	-0.708056
63	8	0	-0.515687	2.151375	0.438250
64	6	0	-3.890788	1.575576	-2.635443
65	1	0	-3.559915	1.905463	-3.622246
66	6	0	-4.924468	0.719799	-2.594956
67	6	0	-5.575241	0.234814	-3.869028
68	1	0	-6.638751	0.506396	-3.896320
69	1	0	-5.535075	-0.860192	-3.938602
70	1	0	-5.094203	0.650816	-4.758514
71	6	0	-5.547815	0.181269	-1.331262
72	1	0	-5.524502	-0.916135	-1.323725
73	1	0	-6.606377	0.466625	-1.275798
74	1	0	-5.061086	0.539932	-0.422804
75	6	0	2.918185	3.355511	2.260784
76	6	0	1.930314	4.381518	2.759920
77	1	0	2.281609	4.827128	3.699847
78	1	0	0.932860	3.975376	2.935135
79	1	0	1.838458	5.208545	2.043947
80	6	0	4.291116	3.908193	1.955787
81	1	0	4.974148	3.132678	1.598298
82	1	0	4.736144	4.371418	2.846388
83	1	0	4.238342	4.696039	1.192774
84	6	0	0.566736	3.513939	-1.256752
85	1	0	0.414365	4.281395	-2.021080
86	1	0	1.137707	2.698596	-1.707676
87	1	0	1.166215	3.941880	-0.449455
88	6	0	-1.515554	4.227117	-0.064900
89	1	0	-1.821930	4.943585	-0.832202
90	1	0	-0.847041	4.737690	0.633197
91	1	0	-2.400913	3.902181	0.484511
92	1	0	-0.968887	-1.292598	1.614567

Conformer 1b-2

Center	Atomic	Atomic	Coordinates (Angstroms)		
Number	Number	Type	X	Y	Z
1	6	0	-0.943146	0.717044	-0.489075
2	6	0	-0.254472	-1.740787	-0.764899
3	6	0	-0.037754	-1.795830	0.768836

4	6	0	-0.173731	-0.576460	1.564562
5	6	0	-0.539479	0.599243	0.973487
6	6	0	-0.120825	-0.315943	-1.280265
7	8	0	0.508844	-0.035502	-2.282417
8	6	0	-2.434474	0.263146	-0.668371
9	1	0	-2.701272	0.474663	-1.712074
10	1	0	-3.071755	0.904081	-0.049843
11	6	0	-1.775348	-2.201826	-1.096029
12	6	0	-2.733540	-1.207722	-0.347314
13	1	0	-2.564587	-1.356554	0.727065
14	6	0	-4.245280	-1.478246	-0.584929
15	1	0	-4.512164	-1.228739	-1.616434
16	1	0	-4.433757	-2.552781	-0.468902
17	6	0	-5.125722	-0.744462	0.394472
18	1	0	-4.917242	-0.977728	1.441088
19	6	0	-6.122435	0.121397	0.149345
20	6	0	-6.913085	0.727672	1.285531
21	1	0	-6.855725	1.825379	1.268152
22	1	0	-6.556779	0.383896	2.261679
23	1	0	-7.979399	0.473255	1.204816
24	6	0	-6.569303	0.563261	-1.223421
25	1	0	-6.537376	1.658556	-1.307125
26	1	0	-7.613513	0.271392	-1.402860
27	1	0	-5.964456	0.147494	-2.032390
28	6	0	-2.019716	-3.637492	-0.583481
29	1	0	-2.995279	-3.993238	-0.931053
30	1	0	-2.004836	-3.689550	0.508468
31	1	0	-1.265279	-4.322245	-0.968380
32	6	0	-2.025786	-2.162600	-2.621186
33	1	0	-1.373476	-2.870089	-3.131296
34	1	0	-1.855456	-1.173863	-3.058668
35	1	0	-3.062306	-2.446384	-2.830437
36	6	0	0.841323	-2.640694	-1.433356
37	6	0	-0.775034	2.151633	-1.053569
38	1	0	-1.768401	2.572740	-1.233673
39	1	0	-0.289658	2.081297	-2.029249
40	6	0	0.013649	3.108482	-0.141594
41	1	0	-0.150402	4.127197	-0.517506
42	8	0	0.530274	-3.565568	-2.163395
43	6	0	2.303744	-2.367171	-1.195697

44	6	0	3.211815	-3.195216	-1.877624
45	1	0	2.813188	-3.971436	-2.521222
46	6	0	4.584300	-3.023021	-1.731155
47	1	0	5.270685	-3.672891	-2.267372
48	6	0	5.077644	-2.017281	-0.896186
49	1	0	6.149614	-1.880093	-0.779766
50	6	0	4.187196	-1.190154	-0.210721
51	1	0	4.559970	-0.406851	0.443333
52	6	0	2.811528	-1.362932	-0.359008
53	1	0	2.150151	-0.711787	0.199518
54	8	0	0.275947	-2.875031	1.266856
55	6	0	1.570303	-0.200228	3.359513
56	1	0	1.706296	0.880881	3.317529
57	6	0	0.164283	-0.664875	3.044580
58	1	0	-0.543162	-0.042686	3.605438
59	1	0	0.015274	-1.699446	3.357705
60	6	0	1.549318	2.862548	-0.184324
61	1	0	2.038395	3.583078	0.475881
62	1	0	1.775796	1.870653	0.230698
63	6	0	-0.593663	3.064025	1.281355
64	8	0	-0.605471	1.680527	1.776536
65	6	0	2.136154	2.955399	-1.572035
66	1	0	2.003466	2.069208	-2.190025
67	6	0	2.791158	3.996104	-2.111968
68	6	0	3.322687	3.919026	-3.524064
69	1	0	4.412497	4.061994	-3.544472
70	1	0	2.894709	4.712664	-4.153002
71	1	0	3.097516	2.955748	-3.991303
72	6	0	3.069066	5.305023	-1.412526
73	1	0	4.148917	5.506576	-1.380681
74	1	0	2.692372	5.340769	-0.387147
75	1	0	2.616748	6.141505	-1.963541
76	6	0	2.637169	-0.951203	3.675616
77	6	0	2.638152	-2.458135	3.765695
78	1	0	3.478003	-2.869045	3.189528
79	1	0	1.725939	-2.911750	3.375630
80	1	0	2.783936	-2.786328	4.805127
81	6	0	3.966186	-0.301443	3.984562
82	1	0	4.314218	-0.574556	4.990965
83	1	0	3.913831	0.791067	3.930453

84	1	0	4.744000	-0.643094	3.286844
85	6	0	-2.040373	3.580542	1.322849
86	1	0	-2.699816	3.043016	0.638583
87	1	0	-2.069689	4.644005	1.060565
88	1	0	-2.441560	3.466561	2.334595
89	6	0	0.228197	3.819378	2.331569
90	1	0	0.346293	4.868466	2.037888
91	1	0	1.218573	3.378982	2.462890
92	1	0	-0.284015	3.789417	3.298290

Conformer 1b-3

Center	Atomic	Atomic	Coordinates (Angstroms)		
Number	Number	Type	X	Y	Z
1	6	0	0.134504	0.631892	-1.053048
2	6	0	0.189123	-1.873116	-0.502874
3	6	0	-0.357875	-1.516347	0.902234
4	6	0	-0.511042	-0.115154	1.291871
5	6	0	-0.249548	0.884773	0.399165
6	6	0	0.922781	-0.687940	-1.109828
7	8	0	1.999179	-0.795450	-1.666504
8	6	0	-1.165005	0.369016	-1.893775
9	1	0	-0.856626	0.327061	-2.946672
10	1	0	-1.816123	1.243649	-1.791607
11	6	0	-1.057445	-2.184951	-1.492215
12	6	0	-1.955197	-0.896772	-1.531489
13	1	0	-2.365321	-0.758109	-0.523845
14	6	0	-3.189926	-1.017279	-2.474089
15	1	0	-2.846913	-1.082048	-3.514595
16	1	0	-3.708967	-1.955576	-2.263993
17	6	0	-4.156442	0.138805	-2.362261
18	1	0	-3.921578	1.010126	-2.974977
19	6	0	-5.260818	0.208873	-1.601694
20	6	0	-6.129321	1.444374	-1.608245
21	1	0	-7.154898	1.204670	-1.922899
22	1	0	-5.740408	2.215593	-2.280912
23	1	0	-6.208415	1.878395	-0.601407
24	6	0	-5.744083	-0.891917	-0.689279
25	1	0	-6.770867	-1.187008	-0.946678
26	1	0	-5.772866	-0.543346	0.352196
27	1	0	-5.117730	-1.786315	-0.720046
28	6	0	-1.882770	-3.372001	-0.948810

29	1	0	-2.640680	-3.661480	-1.683992
30	1	0	-2.389245	-3.122495	-0.012461
31	1	0	-1.248319	-4.239800	-0.773183
32	6	0	-0.539491	-2.539351	-2.905950
33	1	0	0.077383	-3.436294	-2.871191
34	1	0	0.055683	-1.739784	-3.358578
35	1	0	-1.387887	-2.737924	-3.568417
36	6	0	1.183369	-3.072411	-0.331420
37	6	0	0.926223	1.806724	-1.683511
38	1	0	0.292303	2.293698	-2.430298
39	1	0	1.777966	1.392930	-2.227445
40	6	0	1.409376	2.862794	-0.673279
41	1	0	1.729650	3.742100	-1.247871
42	8	0	1.024813	-4.111040	-0.948440
43	6	0	2.361462	-2.958876	0.601577
44	6	0	2.696574	-1.816190	1.342093
45	1	0	2.090417	-0.919704	1.285051
46	6	0	3.814667	-1.810775	2.174501
47	1	0	4.058693	-0.915617	2.739910
48	6	0	4.616369	-2.947838	2.281044
49	1	0	5.487373	-2.941758	2.931166
50	6	0	4.293172	-4.093085	1.549204
51	1	0	4.911325	-4.983382	1.627750
52	6	0	3.177458	-4.096735	0.718356
53	1	0	2.913546	-4.977238	0.143303
54	8	0	-0.616233	-2.435644	1.675710
55	6	0	-2.453333	-0.062512	2.904969
56	1	0	-2.761670	-1.103041	2.811326
57	6	0	-0.967340	0.152452	2.715656
58	1	0	-0.439945	-0.556418	3.366254
59	1	0	-0.669029	1.160162	3.008507
60	6	0	2.645599	2.398753	0.148876
61	1	0	2.935276	3.203885	0.828963
62	1	0	2.365670	1.549950	0.788505
63	6	0	0.209451	3.318193	0.191540
64	8	0	-0.427420	2.152671	0.823188
65	6	0	3.823208	1.996723	-0.705688
66	1	0	3.768287	0.994396	-1.126683
67	6	0	4.907516	2.733867	-0.996204
68	6	0	6.006420	2.173160	-1.868065

69	1	0	6.967303	2.162157	-1.334155
70	1	0	6.156978	2.792658	-2.763632
71	1	0	5.786914	1.151917	-2.193479
72	6	0	5.155115	4.142149	-0.510920
73	1	0	6.091158	4.196771	0.062338
74	1	0	4.353371	4.534413	0.119945
75	1	0	5.275720	4.827691	-1.361399
76	6	0	-3.395822	0.852704	3.176997
77	6	0	-3.147216	2.333671	3.331372
78	1	0	-3.822299	2.904455	2.678144
79	1	0	-2.124452	2.624635	3.084013
80	1	0	-3.361112	2.663033	4.358397
81	6	0	-4.836411	0.437935	3.367293
82	1	0	-5.202952	0.722918	4.364065
83	1	0	-4.968017	-0.643031	3.256548
84	1	0	-5.494324	0.939928	2.642728
85	6	0	-0.870909	4.036560	-0.631616
86	1	0	-1.229394	3.436577	-1.470524
87	1	0	-0.478798	4.979346	-1.029083
88	1	0	-1.728554	4.264590	0.008617
89	6	0	0.600393	4.208855	1.376252
90	1	0	1.136825	5.097105	1.023791
91	1	0	1.234260	3.676578	2.088517
92	1	0	-0.298163	4.540533	1.905906

Conformer 2a-12

Center	Atomic	Atomic	Coordinates (Angstroms)		
Number	Number	Type	X	Y	Z
1	6	0	0.173694	-0.933681	-1.129830
2	6	0	0.527529	1.590240	-1.034718
3	6	0	0.815308	1.439385	0.481854
4	6	0	0.642522	0.141339	1.129630
5	6	0	0.275516	-0.943306	0.385389
6	6	0	-0.330748	0.444070	-1.554324
7	8	0	-1.280678	0.613409	-2.298025
8	6	0	1.607366	-1.082019	-1.745710
9	1	0	2.038804	-2.006853	-1.351515
10	1	0	1.473685	-1.233862	-2.823761
11	6	0	1.933271	1.484000	-1.843721
12	6	0	2.568803	0.088512	-1.494562

13	1	0	2.805205	0.104749	-0.424073
14	6	0	3.920416	-0.183480	-2.217935
15	1	0	3.739165	-0.275702	-3.295751
16	1	0	4.575889	0.679543	-2.087439
17	6	0	4.618471	-1.436654	-1.745470
18	1	0	4.243338	-2.368993	-2.167335
19	6	0	5.640496	-1.529892	-0.878346
20	6	0	6.229774	-2.873738	-0.520524
21	1	0	5.727680	-3.693093	-1.042376
22	1	0	6.157704	-3.060273	0.559080
23	1	0	7.298591	-2.913309	-0.769102
24	6	0	6.295115	-0.357604	-0.189659
25	1	0	7.367517	-0.322951	-0.421655
26	1	0	6.219545	-0.464175	0.900165
27	1	0	5.858290	0.604900	-0.460994
28	6	0	1.681492	1.608518	-3.363657
29	1	0	1.034306	0.819465	-3.755957
30	1	0	2.632935	1.549992	-3.898675
31	1	0	1.224218	2.568509	-3.597373
32	6	0	2.890814	2.608927	-1.396195
33	1	0	3.777459	2.614566	-2.035767
34	1	0	3.218093	2.475677	-0.362415
35	1	0	2.416109	3.584424	-1.486252
36	6	0	-0.235282	2.942511	-1.236963
37	6	0	-0.722842	-2.078746	-1.628737
38	1	0	-1.234340	-1.743554	-2.534379
39	1	0	-0.095332	-2.929811	-1.912983
40	6	0	-1.756528	-2.530701	-0.588105
41	1	0	-2.357436	-1.656013	-0.302719
42	8	0	0.200809	3.802051	-1.991324
43	6	0	-1.519036	3.200742	-0.511416
44	6	0	-2.116589	4.457106	-0.707790
45	1	0	-1.623898	5.167146	-1.360772
46	6	0	-3.312876	4.793549	-0.083802
47	1	0	-3.772789	5.764546	-0.234562
48	6	0	-3.943293	3.873781	0.753383
49	6	0	-3.356702	2.608350	0.959145
50	6	0	-2.162914	2.277673	0.337017
51	1	0	-1.746355	1.295575	0.528481
52	8	0	1.131566	2.452498	1.108841

53	6	0	2.247126	-0.438688	2.985895
54	1	0	2.434487	-1.476282	2.708851
55	6	0	0.863020	0.057411	2.628452
56	1	0	0.673507	1.041862	3.057598
57	1	0	0.125523	-0.636960	3.046353
58	6	0	-2.721373	-3.556605	-1.246707
59	1	0	-3.087749	-3.077983	-2.160778
60	1	0	-2.134216	-4.420503	-1.581403
61	6	0	-1.036004	-3.027889	0.691200
62	8	0	0.119414	-2.128154	0.998731
63	6	0	-3.889946	-4.047582	-0.426585
64	1	0	-3.704226	-4.934475	0.178920
65	6	0	-5.128350	-3.527623	-0.384476
66	6	0	-6.202790	-4.153464	0.471851
67	1	0	-5.836362	-5.030191	1.012949
68	1	0	-7.061355	-4.462064	-0.138980
69	1	0	-6.589797	-3.434595	1.205896
70	6	0	-5.569317	-2.306357	-1.153167
71	1	0	-4.788795	-1.891554	-1.793613
72	1	0	-5.890628	-1.515540	-0.463078
73	1	0	-6.438682	-2.538599	-1.781517
74	6	0	3.237934	0.234693	3.591981
75	6	0	3.157896	1.676961	4.030543
76	1	0	4.044127	2.226051	3.688138
77	1	0	3.154174	1.751996	5.126191
78	1	0	2.278822	2.193091	3.643275
79	6	0	4.551557	-0.444588	3.900647
80	1	0	4.558157	-1.490104	3.580040
81	1	0	4.764522	-0.417100	4.977696
82	1	0	5.387330	0.071377	3.409643
83	6	0	-0.411542	-4.416412	0.560954
84	1	0	0.203421	-4.506449	-0.338115
85	1	0	-1.186210	-5.186524	0.529703
86	1	0	0.223135	-4.611273	1.429460
87	6	0	-1.912674	-2.929057	1.941783
88	1	0	-2.791461	-3.569807	1.850478
89	1	0	-2.256137	-1.900388	2.087225
90	1	0	-1.344202	-3.234688	2.824537
91	8	0	-4.061440	1.784647	1.797290
92	1	0	-3.613696	0.933715	1.899140

93	8	0	-5.110867	4.193852	1.364087
94	1	0	-5.388389	3.431969	1.898769

Conformer 2a-13

Center	Atomic	Atomic	Coordinates (Angstroms)		
Number	Number	Type	X	Y	Z
1	6	0	0.588301	-1.183055	-0.867008
2	6	0	1.070781	1.324192	-1.049305
3	6	0	1.134764	1.368041	0.497465
4	6	0	0.793835	0.183595	1.272553
5	6	0	0.475356	-0.989704	0.640061
6	6	0	0.226546	0.153524	-1.526689
7	8	0	-0.605582	0.260765	-2.409567
8	6	0	2.089865	-1.452732	-1.241637
9	1	0	2.417947	-2.334835	-0.681666
10	1	0	2.107268	-1.734496	-2.300837
11	6	0	2.564677	1.051472	-1.628363
12	6	0	3.065711	-0.292795	-0.986561
13	1	0	3.112824	-0.132375	0.095526
14	6	0	4.503143	-0.750033	-1.405117
15	1	0	4.528353	-1.841270	-1.317769
16	1	0	4.665400	-0.544389	-2.468043
17	6	0	5.647159	-0.167898	-0.606464
18	1	0	6.083738	0.750629	-0.994504
19	6	0	6.184412	-0.666683	0.519505
20	6	0	7.340886	0.027443	1.198713
21	1	0	7.643209	0.933814	0.666859
22	1	0	8.212797	-0.636589	1.267215
23	1	0	7.084145	0.305442	2.229471
24	6	0	5.715247	-1.923314	1.211073
25	1	0	4.909588	-2.435083	0.681307
26	1	0	5.356640	-1.694143	2.222995
27	1	0	6.545742	-2.630821	1.332049
28	6	0	2.516603	0.951808	-3.170464
29	1	0	1.874528	0.144334	-3.531455
30	1	0	3.521009	0.772598	-3.562562
31	1	0	2.154630	1.886061	-3.596496
32	6	0	3.512218	2.201479	-1.233585
33	1	0	4.473346	2.062623	-1.734801
34	1	0	3.691818	2.230250	-0.157027
35	1	0	3.110865	3.163861	-1.547492

36	6	0	0.414616	2.660147	-1.538407
37	6	0	-0.295016	-2.337951	-1.386275
38	1	0	-0.788424	-1.995407	-2.297560
39	1	0	0.340453	-3.183052	-1.668691
40	6	0	-1.347087	-2.808463	-0.377158
41	1	0	-2.017463	-1.964579	-0.160284
42	8	0	0.977583	3.374785	-2.356923
43	6	0	-0.919918	3.080686	-1.006230
44	6	0	-1.404143	4.335586	-1.411481
45	1	0	-0.800507	4.930339	-2.086277
46	6	0	-2.627661	4.817464	-0.958305
47	1	0	-2.999286	5.788506	-1.268249
48	6	0	-3.397770	4.051972	-0.082756
49	6	0	-2.926067	2.789253	0.328759
50	6	0	-1.710472	2.309350	-0.131934
51	1	0	-1.391133	1.331591	0.207856
52	8	0	1.438694	2.438034	1.032295
53	6	0	-0.546763	0.905222	3.280745
54	1	0	-0.836805	1.834645	2.790805
55	6	0	0.760666	0.328208	2.784852
56	1	0	0.972338	-0.635429	3.248219
57	1	0	1.572272	1.013403	3.058584
58	6	0	-2.221800	-3.951039	-0.957943
59	1	0	-1.567948	-4.790432	-1.227660
60	1	0	-2.893317	-4.320670	-0.179398
61	6	0	-0.673774	-3.183524	0.959974
62	8	0	0.153409	-2.034950	1.411903
63	6	0	-3.010265	-3.543830	-2.178557
64	1	0	-2.442572	-3.501707	-3.107590
65	6	0	-4.315548	-3.233847	-2.246503
66	6	0	-4.949580	-2.850928	-3.562604
67	1	0	-4.227642	-2.861702	-4.383709
68	1	0	-5.391880	-1.847594	-3.508568
69	1	0	-5.769012	-3.535824	-3.817631
70	6	0	-5.264890	-3.232200	-1.073478
71	1	0	-4.793330	-3.512582	-0.130088
72	1	0	-6.100810	-3.920553	-1.253248
73	1	0	-5.708948	-2.236931	-0.943631
74	6	0	-1.354647	0.434675	4.245266
75	6	0	-1.095148	-0.805912	5.063976

76	1	0	-1.940354	-1.501887	4.990777
77	1	0	-1.000209	-0.547708	6.126891
78	1	0	-0.191044	-1.340604	4.768485
79	6	0	-2.623172	1.170427	4.612870
80	1	0	-2.799276	2.034126	3.965689
81	1	0	-2.587176	1.523016	5.652153
82	1	0	-3.496088	0.508105	4.543586
83	6	0	0.264411	-4.391621	0.895813
84	1	0	1.021149	-4.291937	0.114490
85	1	0	-0.302764	-5.306939	0.708303
86	1	0	0.778712	-4.503238	1.854028
87	6	0	-1.683734	-3.348599	2.098047
88	1	0	-2.304095	-4.234542	1.943569
89	1	0	-2.333889	-2.472048	2.167822
90	1	0	-1.157570	-3.466853	3.048643
91	8	0	-3.754816	2.119208	1.192460
92	1	0	-3.359015	1.278698	1.461731
93	8	0	-4.587378	4.520321	0.368810
94	1	0	-4.963933	3.856394	0.969263

Conformer 3a-1

Center	Atomic	Atomic	Coordinates (Angstroms)		
Number	Number	Type	X	Y	Z
1	6	0	-0.123996	-0.278603	-1.219699
2	6	0	1.399202	1.629399	-0.352399
3	6	0	1.665903	0.811199	0.943101
4	6	0	0.980705	-0.452002	1.062701
5	6	0	0.210705	-0.964503	0.073901
6	6	0	0.075002	1.235097	-1.011799
7	8	0	-0.690199	2.060496	-1.470999
8	6	0	0.990005	-0.655702	-2.267899
9	1	0	0.687404	-0.192902	-3.213499
10	1	0	0.956907	-1.740002	-2.427799
11	6	0	2.521402	1.299401	-1.478599
12	6	0	2.437704	-0.214400	-1.925099
13	6	0	3.220506	-1.256898	-1.053399
14	1	0	3.361105	-0.903698	-0.032399
15	1	0	2.601907	-2.163499	-0.972799
16	6	0	4.543706	-1.654397	-1.660199
17	1	0	4.484607	-1.939797	-2.713299

18	6	0	5.751107	-1.723495	-1.077499
19	6	0	6.960207	-2.171893	-1.865099
20	1	0	7.423909	-3.058892	-1.410199
21	1	0	7.733906	-1.391392	-1.878599
22	1	0	6.706908	-2.416393	-2.901699
23	6	0	6.039806	-1.381494	0.363901
24	1	0	6.432807	-2.258894	0.897101
25	1	0	5.166706	-1.021096	0.912101
26	1	0	6.816805	-0.607693	0.428301
27	6	0	2.267701	2.156900	-2.747299
28	1	0	3.041401	1.921801	-3.487699
29	1	0	2.329999	3.219400	-2.517299
30	1	0	1.295201	1.969399	-3.210999
31	6	0	3.940202	1.633103	-0.973699
32	1	0	4.164102	1.181503	-0.005999
33	1	0	4.059500	2.711703	-0.870699
34	1	0	4.673902	1.266704	-1.700299
35	6	0	1.358100	3.136599	0.066401
36	8	0	2.138098	3.944100	-0.406699
37	6	0	0.348799	3.612797	1.078001
38	6	0	-0.574200	2.791396	1.740801
39	1	0	-0.611498	1.725096	1.550501
40	6	0	-1.465601	3.329595	2.668201
41	1	0	-2.169300	2.676494	3.176901
42	6	0	-1.452503	4.697495	2.944001
43	1	0	-2.149203	5.114794	3.666401
44	6	0	-0.538504	5.526296	2.288801
45	1	0	-0.521105	6.592596	2.498201
46	6	0	0.353797	4.987697	1.367501
47	1	0	1.071896	5.616598	0.853201
48	8	0	2.456602	1.232400	1.784301
49	6	0	1.119206	-1.480502	2.156701
50	1	0	0.494406	-1.234602	3.023201
51	1	0	2.151206	-1.593200	2.501601
52	6	0	0.607508	-2.745502	1.434201
53	1	0	1.448209	-3.304601	1.005101
54	8	0	-0.149493	-2.249603	0.263801
55	6	0	-0.234190	-3.730103	2.255601
56	6	0	0.641410	-4.297202	3.387601
57	1	0	1.518911	-4.825201	2.997501

58	1	0	0.061911	-5.016703	3.979401
59	1	0	0.979309	-3.501602	4.058201
60	6	0	-0.771089	-4.864904	1.370401
61	1	0	0.046512	-5.439103	0.919401
62	1	0	-1.400389	-4.465505	0.573201
63	1	0	-1.369888	-5.559005	1.973201
64	8	0	-1.316892	-2.976105	2.813201
65	1	0	-1.818891	-3.573106	3.390201
66	6	0	-1.506295	-0.629805	-1.825599
67	1	0	-1.518296	-0.180505	-2.824799
68	1	0	-1.536293	-1.715805	-1.976399
69	6	0	-2.796996	-0.156707	-1.103499
70	6	0	-3.088595	-0.887708	0.196701
71	6	0	-3.980996	-0.256409	-2.107699
72	1	0	-3.711396	0.352391	-2.984799
73	1	0	-4.076194	-1.285709	-2.472499
74	6	0	-5.281096	0.248489	-1.540399
75	1	0	-5.194798	1.192889	-1.000199
76	6	0	-6.504295	-0.296813	-1.627999
77	6	0	-7.698296	0.382786	-0.999199
78	1	0	-8.184996	-0.272715	-0.262799
79	1	0	-8.462097	0.622185	-1.752999
80	1	0	-7.419198	1.312086	-0.492999
81	6	0	-3.488293	-2.341008	0.117901
82	1	0	-4.508192	-2.438010	-0.276399
83	1	0	-3.452592	-2.808408	1.104701
84	1	0	-2.830092	-2.912407	-0.546699
85	6	0	-3.046896	-0.241008	1.367201
86	1	0	-2.823197	0.821493	1.421601
87	1	0	-3.237095	-0.749908	2.308201
88	1	0	2.952704	-0.223099	-2.894399
89	6	0	-6.829294	-1.584613	-2.346399
90	1	0	-7.335493	-2.290214	-1.672699
91	1	0	-5.949993	-2.084612	-2.759499
92	1	0	-7.525494	-1.398914	-3.176699
93	1	0	-2.670697	0.904293	-0.870699

Conformer 4a-2

Center	Atomic	Atomic	Coordinates (Angstroms)		
Number	Number	Type	X	Y	Z

1	6	0	-0.382807	-0.069340	-1.550136
2	6	0	1.072225	-1.797771	-0.300526
3	6	0	0.562707	-1.233364	1.054350
4	6	0	-0.289065	-0.067931	0.994388
5	6	0	-0.717163	0.448005	-0.183166
6	6	0	0.982184	-0.770878	-1.427302
7	8	0	1.867798	-0.611803	-2.243377
8	6	0	-1.373517	-1.210936	-1.972415
9	1	0	-1.130227	-1.443655	-3.015255
10	1	0	-2.392201	-0.807134	-1.971521
11	6	0	0.133247	-3.016478	-0.803340
12	6	0	-1.336428	-2.515800	-1.128585
13	6	0	-2.383529	-2.517200	0.027641
14	1	0	-2.279664	-3.463982	0.571536
15	1	0	-2.205395	-1.727019	0.756637
16	6	0	-3.787855	-2.450160	-0.520905
17	1	0	-3.990440	-3.180250	-1.308914
18	6	0	-4.803287	-1.645010	-0.169138
19	6	0	-6.146635	-1.764660	-0.851929
20	1	0	-6.442838	-0.811244	-1.312209
21	1	0	-6.936212	-2.016812	-0.129944
22	1	0	-6.144276	-2.532076	-1.632584
23	6	0	-4.744756	-0.587809	0.906455
24	1	0	-4.941268	0.406814	0.479647
25	1	0	-3.793687	-0.547849	1.440811
26	1	0	-5.535135	-0.760634	1.650633
27	6	0	0.701831	-3.597860	-2.125988
28	1	0	0.084712	-4.452692	-2.426124
29	1	0	1.724474	-3.947996	-1.992490
30	1	0	0.693741	-2.878159	-2.949620
31	6	0	0.107749	-4.159439	0.231054
32	1	0	-0.188110	-3.825380	1.227051
33	1	0	1.095122	-4.614915	0.309882
34	1	0	-0.600207	-4.928827	-0.101204
35	6	0	2.572690	-2.184942	-0.069929
36	8	0	2.960451	-3.329910	-0.216898
37	6	0	3.560849	-1.125512	0.347724
38	6	0	3.249050	0.221699	0.588776
39	1	0	2.236516	0.595889	0.491960
40	6	0	4.239361	1.122284	0.978388

41	1	0	3.971857	2.158343	1.166766
42	6	0	5.558853	0.693938	1.128317
43	1	0	6.329872	1.397628	1.431187
44	6	0	5.883036	-0.643929	0.889413
45	1	0	6.908017	-0.986097	1.004545
46	6	0	4.893309	-1.542913	0.504903
47	1	0	5.128695	-2.584905	0.318774
48	8	0	0.917797	-1.773287	2.099477
49	6	0	-0.851757	0.760113	2.125766
50	1	0	-0.054059	1.127427	2.782450
51	8	0	-1.850718	0.112542	2.909923
52	6	0	-1.356775	-0.561743	4.065402
53	1	0	-0.633474	-1.339003	3.801691
54	1	0	-2.227416	-1.004713	4.555269
55	1	0	-0.880622	0.149407	4.757888
56	6	0	-1.548744	1.910822	1.353241
57	1	0	-2.602527	1.944127	1.639502
58	8	0	-1.512591	1.523134	-0.068716
59	6	0	-0.908363	3.315977	1.451886
60	6	0	-0.771096	3.754833	2.912461
61	1	0	-1.731269	3.692130	3.435901
62	1	0	-0.423660	4.791847	2.947092
63	1	0	-0.040375	3.141753	3.446357
64	6	0	-1.766889	4.332222	0.679689
65	1	0	-2.752937	4.453040	1.142928
66	1	0	-1.916529	4.016007	-0.356692
67	1	0	-1.264545	5.304825	0.680438
68	8	0	0.415921	3.268523	0.915474
69	1	0	0.337241	3.115228	-0.045064
70	6	0	-0.361609	1.044717	-2.626055
71	1	0	-0.067604	0.558440	-3.565268
72	1	0	-1.384169	1.408157	-2.760582
73	6	0	0.597917	2.178907	-2.354910
74	1	0	1.587196	1.868088	-2.023751
75	6	0	0.406564	3.493007	-2.573821
76	6	0	-0.860193	4.102859	-3.123949
77	1	0	-1.694820	3.399729	-3.175206
78	1	0	-0.685635	4.486837	-4.138826
79	1	0	-1.176260	4.962490	-2.518854
80	6	0	1.521561	4.483348	-2.322657

81	1	0	1.756683	5.047053	-3.235887
82	1	0	2.437488	3.990161	-1.983380
83	1	0	1.231034	5.221805	-1.563031
84	1	0	-1.712342	-3.291804	-1.810218

Conformer 4a-4

Center	Atomic	Atomic	Coordinates (Angstroms)		
Number	Number	Type	X	Y	Z
1	6	0	0.245058	-0.135858	1.573998
2	6	0	-0.858133	-1.758406	-0.100358
3	6	0	-0.240811	-0.954972	-1.278265
4	6	0	0.514617	0.227819	-0.935867
5	6	0	0.730717	0.586526	0.353160
6	6	0	-1.012667	-0.920821	1.169604
7	8	0	-1.991524	-0.995259	1.883456
8	6	0	1.270098	-1.250505	1.986611
9	1	0	0.901157	-1.655195	2.935895
10	1	0	2.234578	-0.776618	2.201228
11	6	0	0.126029	-2.962997	0.350889
12	6	0	1.478099	-2.412231	0.974395
13	6	0	2.682554	-2.169569	0.014767
14	1	0	2.744749	-3.025335	-0.668595
15	1	0	2.548472	-1.289575	-0.614171
16	6	0	3.977367	-2.100573	0.788068
17	1	0	4.093463	-2.895357	1.529659
18	6	0	4.992296	-1.229098	0.674582
19	6	0	6.217550	-1.358676	1.550168
20	1	0	6.383646	-0.443287	2.136070
21	1	0	7.123198	-1.505813	0.944722
22	1	0	6.137549	-2.197634	2.248777
23	6	0	5.046104	-0.081997	-0.304721
24	1	0	5.132682	0.876652	0.228125
25	1	0	4.182881	-0.028597	-0.970895
26	1	0	5.944594	-0.161948	-0.933093
27	6	0	-0.555466	-3.801108	1.467197
28	1	0	0.100322	-4.641973	1.721374
29	1	0	-1.508804	-4.204651	1.129258
30	1	0	-0.732829	-3.230532	2.383179
31	6	0	0.412171	-3.912832	-0.829732
32	1	0	0.814964	-3.396404	-1.703010
33	1	0	-0.504112	-4.419727	-1.132984

34	1	0	1.136724	-4.673121	-0.513077
35	6	0	-2.266318	-2.227602	-0.598234
36	8	0	-2.547320	-3.409342	-0.688414
37	6	0	-3.296471	-1.201064	-0.995822
38	6	0	-3.127674	0.188785	-0.904337
39	1	0	-2.209811	0.622691	-0.525388
40	6	0	-4.147062	1.053830	-1.300130
41	1	0	-3.996693	2.127015	-1.221700
42	6	0	-5.350202	0.546410	-1.791722
43	1	0	-6.143020	1.223540	-2.099191
44	6	0	-5.530238	-0.835815	-1.887141
45	1	0	-6.463710	-1.239730	-2.269883
46	6	0	-4.513042	-1.698690	-1.492698
47	1	0	-4.636325	-2.773864	-1.560508
48	8	0	-0.426827	-1.345071	-2.429427
49	6	0	1.213373	1.214736	-1.844370
50	1	0	0.509152	1.689612	-2.538513
51	8	0	2.322274	0.692768	-2.572316
52	6	0	2.002942	0.201011	-3.872338
53	1	0	1.266791	-0.606618	-3.827643
54	1	0	2.940760	-0.162473	-4.299760
55	1	0	1.610362	1.008994	-4.509237
56	6	0	1.785762	2.229527	-0.815563
57	1	0	2.875055	2.239102	-0.900190
58	8	0	1.478426	1.689960	0.511241
59	6	0	1.255219	3.667604	-0.915811
60	6	0	1.695730	4.287596	-2.252182
61	1	0	2.787088	4.341687	-2.330208
62	1	0	1.306062	5.309827	-2.330994
63	1	0	1.315208	3.716267	-3.103552
64	6	0	1.753431	4.519871	0.260722
65	1	0	2.846669	4.595529	0.262875
66	1	0	1.431220	4.092914	1.212712
67	1	0	1.352698	5.538237	0.180114
68	8	0	-0.175253	3.548769	-0.872191
69	1	0	-0.546997	4.444775	-0.891624
70	6	0	-0.029716	0.823415	2.761427
71	1	0	-0.395112	0.215781	3.591883
72	1	0	0.938695	1.240526	3.069630
73	6	0	-0.983685	1.947120	2.440850

74	1	0	-0.645220	2.623497	1.657545
75	6	0	-2.182550	2.197722	2.990062
76	6	0	-2.842234	1.377196	4.068978
77	1	0	-2.247686	0.520082	4.387944
78	1	0	-3.806418	0.990309	3.713599
79	1	0	-3.056010	1.997984	4.950531
80	6	0	-2.996111	3.383122	2.524383
81	1	0	-3.969583	3.058717	2.130435
82	1	0	-2.482206	3.946429	1.738553
83	1	0	-3.210631	4.071204	3.354509
84	1	0	1.817782	-3.254369	1.593389

Conformer 5a-2

Center	Atomic	Atomic	Coordinates (Angstroms)		
Number	Number	Type	X	Y	Z
1	6	0	0.784003	0.452090	-0.314197
2	6	0	-1.348002	-0.199695	0.978803
3	6	0	-1.717008	-1.062792	-0.210297
4	6	0	-0.982109	-1.148998	-1.353397
5	6	0	0.312396	-0.481807	-1.465497
6	8	0	1.045594	-0.680812	-2.429097
7	6	0	-0.408194	0.917698	0.499703
8	8	0	-0.596786	2.063500	0.878203
9	6	0	1.628596	-0.414617	0.689303
10	1	0	2.451293	-0.862522	0.124303
11	1	0	2.089901	0.284180	1.391703
12	6	0	-0.495108	-1.015401	2.087203
13	6	0	0.876788	-1.517511	1.478103
14	1	0	1.488587	-1.689815	2.374503
15	6	0	0.909978	-2.894111	0.743603
16	1	0	0.220273	-3.581206	1.248703
17	1	0	0.562179	-2.820409	-0.285997
18	6	0	2.284574	-3.514021	0.800603
19	1	0	2.658273	-3.671724	1.815503
20	6	0	3.074671	-3.917027	-0.208097
21	6	0	4.403966	-4.577637	0.077903
22	1	0	5.232270	-4.017643	-0.378597
23	1	0	4.441559	-5.588637	-0.352197
24	1	0	4.599666	-4.660338	1.151803
25	6	0	2.743772	-3.796125	-1.676197

26	1	0	3.583575	-3.344631	-2.221297
27	1	0	1.861776	-3.187218	-1.880997
28	1	0	2.586865	-4.790023	-2.119697
29	6	0	-1.303516	-2.189695	2.673803
30	1	0	-2.143214	-1.816989	3.261003
31	1	0	-1.697921	-2.860892	1.908403
32	1	0	-0.654521	-2.776000	3.336103
33	6	0	-0.131401	-0.069204	3.263303
34	1	0	0.414395	-0.647408	4.017503
35	1	0	-1.024398	0.341903	3.731903
36	1	0	0.508905	0.763192	2.959403
37	6	0	-2.674897	0.443315	1.534903
38	6	0	1.628611	1.585583	-0.968097
39	1	0	2.389207	1.066178	-1.555997
40	1	0	0.975414	2.084488	-1.689897
41	6	0	2.294819	2.679079	-0.081897
42	1	0	1.990218	2.541881	0.964103
43	6	0	1.801129	4.123282	-0.483197
44	6	0	2.069036	5.121680	0.659103
45	1	0	1.541734	4.808884	1.568703
46	1	0	1.690444	6.108883	0.374803
47	1	0	3.133037	5.221473	0.901603
48	8	0	0.395329	4.122292	-0.748997
49	1	0	-0.033075	3.606796	-0.041097
50	6	0	2.432733	4.622778	-1.789697
51	1	0	3.502734	4.823470	-1.680897
52	1	0	1.936740	5.552481	-2.086397
53	1	0	2.292928	3.898079	-2.599097
54	6	0	3.848718	2.593767	-0.131697
55	1	0	4.165718	2.553965	-1.178397
56	1	0	4.260525	3.528664	0.273303
57	6	0	4.451310	1.462763	0.656403
58	1	0	4.226410	1.495465	1.725103
59	6	0	5.240003	0.461557	0.233403
60	6	0	5.766395	-0.567346	1.206903
61	1	0	5.408388	-1.573944	0.948203
62	1	0	5.457697	-0.353344	2.235403
63	1	0	6.864495	-0.610454	1.184403
64	6	0	5.677801	0.246054	-1.194997
65	1	0	6.773901	0.273846	-1.271997

66	1	0	5.274807	0.986557	-1.888997
67	1	0	5.367394	-0.746844	-1.549497
68	8	0	-3.011499	0.264717	2.691103
69	6	0	-3.569292	1.259321	0.636003
70	6	0	-4.802389	1.653630	1.184503
71	1	0	-5.028391	1.357632	2.202803
72	6	0	-5.707183	2.405937	0.442403
73	1	0	-6.655181	2.700543	0.884403
74	6	0	-5.395080	2.784034	-0.865797
75	1	0	-6.097776	3.375639	-1.446597
76	6	0	-4.173083	2.403826	-1.422297
77	1	0	-3.915781	2.703424	-2.434197
78	6	0	-3.267389	1.647319	-0.679297
79	1	0	-2.326991	1.376012	-1.141197
80	8	0	-2.858013	-1.736584	-0.023297
81	6	0	-3.506019	-2.548079	-1.087097
82	6	0	-2.603520	-2.698986	-2.288397
83	1	0	-2.962525	-3.367983	-3.066497
84	6	0	-1.445715	-2.041894	-2.411197
85	1	0	-0.810516	-2.148599	-3.284097
86	6	0	-3.798929	-3.899877	-0.427897
87	1	0	-4.407428	-3.760973	0.471603
88	1	0	-2.868733	-4.405784	-0.152697
89	1	0	-4.348934	-4.543673	-1.122897
90	6	0	-4.800914	-1.809370	-1.446297
91	1	0	-5.359518	-2.379766	-2.196997
92	1	0	-4.579907	-0.820272	-1.855897
93	1	0	-5.430813	-1.688565	-0.558797

Conformer 5a -3

Center	Atomic	Atomic	Coordinates (Angstroms)		
Number	Number	Type	X	Y	Z
1	6	0	-1.038307	0.443693	0.018902
2	6	0	1.191998	-0.010986	1.221402
3	6	0	1.870694	0.377021	-0.074998
4	6	0	1.214390	0.763114	-1.203698
5	6	0	-0.241011	0.892100	-1.220898
6	8	0	-0.815916	1.443795	-2.158598
7	6	0	-0.243498	-0.479700	0.922102
8	8	0	-0.722589	-1.425404	1.519002
9	6	0	-1.231819	1.729891	0.917402

10	1	0	-1.743026	2.485386	0.310702
11	1	0	-1.926816	1.441184	1.713102
12	6	0	0.997185	1.265312	2.205102
13	6	0	0.042175	2.348303	1.549402
14	1	0	-0.322631	2.905199	2.423502
15	6	0	0.668964	3.459409	0.649802
16	1	0	1.651362	3.729118	1.055402
17	1	0	0.846267	3.116111	-0.368598
18	6	0	-0.182048	4.705601	0.656502
19	1	0	-0.340452	5.127099	1.652502
20	6	0	-0.738754	5.360295	-0.375298
21	6	0	-1.527366	6.628188	-0.140198
22	1	0	-2.560465	6.523778	-0.500498
23	1	0	-1.091475	7.472392	-0.693398
24	1	0	-1.562369	6.900687	0.919602
25	6	0	-0.634050	4.952496	-1.825198
26	1	0	-1.625950	4.962087	-2.296598
27	1	0	-0.216341	3.955500	-1.973198
28	1	0	-0.019157	5.672502	-2.384798
29	6	0	2.354079	1.887025	2.589202
30	1	0	2.932386	1.188331	3.194302
31	1	0	2.956077	2.161931	1.721002
32	1	0	2.180271	2.793224	3.182802
33	6	0	0.303790	0.813605	3.518802
34	1	0	0.223181	1.681505	4.183402
35	1	0	0.884397	0.047811	4.030802
36	1	0	-0.704507	0.423896	3.356902
37	6	0	2.009709	-1.191978	1.860902
38	6	0	-2.447002	-0.091021	-0.344298
39	1	0	-3.043502	-0.027027	0.571302
40	1	0	-2.883709	0.628775	-1.044998
41	6	0	-2.656988	-1.536823	-0.869698
42	1	0	-2.120381	-2.214418	-0.197398
43	6	0	-2.103585	-1.837518	-2.290698
44	6	0	-2.273171	-3.320519	-2.669398
45	1	0	-1.905464	-3.968616	-1.866398
46	1	0	-1.685568	-3.532214	-3.570798
47	1	0	-3.312668	-3.586829	-2.885498
48	8	0	-0.674687	-1.601904	-2.182398
49	1	0	-0.344790	-1.347101	-3.057998

50	6	0	-2.692593	-0.942923	-3.390298
51	1	0	-3.775392	-1.083034	-3.474298
52	1	0	-2.259691	-1.211019	-4.364198
53	1	0	-2.482404	0.112179	-3.201798
54	6	0	-4.169984	-1.874738	-0.745998
55	1	0	-4.759392	-1.142243	-1.309498
56	1	0	-4.356275	-2.847339	-1.219998
57	6	0	-4.638284	-1.964342	0.684002
58	1	0	-4.023777	-2.613136	1.311102
59	6	0	-5.695489	-1.373952	1.264002
60	6	0	-6.011687	-1.621555	2.720802
61	1	0	-6.014696	-0.681555	3.290902
62	1	0	-5.288280	-2.297948	3.186602
63	1	0	-7.013183	-2.059065	2.840702
64	6	0	-6.661998	-0.447362	0.566602
65	1	0	-7.674994	-0.874471	0.563902
66	1	0	-6.388500	-0.234659	-0.469498
67	1	0	-6.733007	0.511038	1.099602
68	8	0	2.501108	-1.082673	2.970202
69	6	0	2.237121	-2.464076	1.087002
70	6	0	3.143730	-3.380167	1.649902
71	1	0	3.612428	-3.124463	2.593802
72	6	0	3.429742	-4.583864	1.013702
73	1	0	4.134648	-5.278558	1.462802
74	6	0	2.807845	-4.899070	-0.197698
75	1	0	3.025854	-5.840968	-0.694798
76	6	0	1.901936	-4.000679	-0.762298
77	1	0	1.400238	-4.236084	-1.696898
78	6	0	1.617624	-2.790082	-0.129198
79	1	0	0.900118	-2.133689	-0.604098
80	8	0	3.209294	0.340233	0.010402
81	6	0	4.091793	0.499942	-1.171298
82	6	0	3.340587	1.109135	-2.331698
83	1	0	3.946684	1.447241	-3.168698
84	6	0	2.006486	1.205022	-2.347098
85	1	0	1.468582	1.629817	-3.188598
86	6	0	5.222384	1.413153	-0.687698
87	1	0	5.711788	0.980358	0.190602
88	1	0	4.834174	2.401649	-0.424498
89	1	0	5.971183	1.534560	-1.478098

90	6	0	4.615006	-0.900553	-1.514798
91	1	0	5.323606	-0.838646	-2.348798
92	1	0	3.794113	-1.560661	-1.806598
93	1	0	5.126210	-1.340348	-0.651998

Conformer 6a-1

Center Number	Atomic Number	Atomic Type	Coordinates (Angstroms) X	Y	Z
1	6	0	-0.191368	0.273770	-1.136538
2	6	0	-0.701052	-0.314715	0.200285
3	6	0	0.450920	-1.036294	0.907089
4	6	0	1.730923	-0.201898	1.068574
5	6	0	2.099787	0.414713	-0.269911
6	6	0	1.224839	0.607931	-1.290489
7	8	0	3.406649	0.735641	-0.320995
8	6	0	4.010051	1.218355	-1.576227
9	6	0	2.971120	2.037780	-2.353224
10	1	0	3.409149	2.352246	-3.307362
11	1	0	2.754913	2.949991	-1.782897
12	6	0	1.671038	1.258648	-2.576940
13	1	0	0.879718	1.924283	-2.935920
14	1	0	1.794286	0.503792	-3.365512
15	6	0	-1.068953	0.854115	1.186516
16	1	0	-1.555205	0.380145	2.045627
17	1	0	-1.826530	1.477932	0.704755
18	6	0	0.087891	1.749126	1.697559
19	1	0	-0.284068	2.175555	2.639621
20	6	0	1.351367	0.920752	2.166939
21	8	0	0.359527	-2.136793	1.415911
22	6	0	2.907677	-1.149981	1.500391
23	6	0	3.380133	-2.253611	0.584909
24	6	0	2.710695	-2.702062	-0.564502
25	1	0	1.768978	-2.263589	-0.867051
26	8	0	-0.978211	0.477958	-2.057562
27	6	0	-1.887582	-1.301028	-0.037726
28	6	0	-3.356568	-0.858074	0.207350
29	1	0	-3.415083	-0.370771	1.188417
30	6	0	-4.220761	-2.152422	0.318096
31	1	0	-3.779468	-2.766384	1.118434
32	1	0	-4.117644	-2.740310	-0.600308

33	6	0	-5.665930	-1.887552	0.643496
34	1	0	-5.818227	-1.171410	1.453283
35	6	0	-6.764455	-2.422386	0.088707
36	6	0	-8.140817	-2.037918	0.579165
37	1	0	-8.692854	-2.915953	0.944268
38	1	0	-8.744722	-1.608188	-0.232786
39	1	0	-8.095070	-1.305059	1.390916
40	6	0	-6.764814	-3.434637	-1.031533
41	1	0	-7.235851	-4.372813	-0.705195
42	1	0	-5.764553	-3.677808	-1.397703
43	1	0	-7.357777	-3.071870	-1.882777
44	6	0	-3.950725	0.109839	-0.808374
45	6	0	-4.062153	-0.339811	-2.244591
46	1	0	-3.072847	-0.525176	-2.672821
47	1	0	-4.554159	0.424526	-2.854348
48	1	0	-4.649148	-1.263137	-2.329912
49	6	0	-4.427513	1.298365	-0.418014
50	1	0	-4.896469	1.983011	-1.121062
51	1	0	-4.375010	1.629978	0.616639
52	1	0	-1.704134	-2.147028	0.631242
53	1	0	-1.803564	-1.693878	-1.056306
54	6	0	4.592827	-2.871234	0.937138
55	1	0	5.101114	-2.527843	1.831310
56	6	0	5.125354	-3.896980	0.162123
57	1	0	6.065610	-4.359366	0.450160
58	6	0	4.449031	-4.333737	-0.979699
59	1	0	4.859379	-5.138105	-1.584479
60	6	0	3.240338	-3.735237	-1.337275
61	1	0	2.702006	-4.075633	-2.217561
62	8	0	3.481097	-0.989335	2.562345
63	6	0	4.501921	-0.007455	-2.351814
64	1	0	5.011136	0.313152	-3.267671
65	1	0	3.679210	-0.670664	-2.630752
66	1	0	5.205881	-0.583977	-1.744748
67	6	0	0.956412	0.235427	3.503289
68	1	0	0.725674	1.014069	4.239545
69	1	0	1.774273	-0.368640	3.893514
70	1	0	0.075304	-0.405557	3.413062
71	6	0	2.542517	1.852925	2.468274
72	1	0	2.206732	2.662298	3.128452

73	1	0	2.971532	2.303870	1.571508
74	1	0	3.334027	1.302304	2.977437
75	6	0	0.301895	3.000454	0.789651
76	1	0	0.380425	2.726637	-0.261712
77	1	0	1.253753	3.475246	1.055300
78	6	0	5.183050	2.081874	-1.116975
79	1	0	5.738095	2.453168	-1.985090
80	1	0	5.866789	1.499114	-0.491918
81	1	0	4.830982	2.941197	-0.537456
82	6	0	-0.793112	4.017430	0.994229
83	1	0	-0.855907	4.402780	2.014958
84	6	0	-1.672327	4.504634	0.103813
85	6	0	-1.755952	4.102995	-1.348740
86	1	0	-2.778213	3.791500	-1.599223
87	1	0	-1.097227	3.274602	-1.615163
88	1	0	-1.518322	4.956269	-2.000224
89	6	0	-2.680972	5.551123	0.517901
90	1	0	-2.569068	6.466128	-0.081123
91	1	0	-2.580839	5.823586	1.573425
92	1	0	-3.707898	5.196656	0.352094

Conformer 7a-1

Center	Atomic	Atomic	Coordinates (Angstroms)		
Number	Number	Type	X	Y	Z
1	6	0	0.639227	-0.199006	1.156851
2	6	0	0.867709	-0.594754	-0.322558
3	6	0	-0.484414	-0.695800	-1.036460
4	6	0	-1.389546	0.537173	-0.882156
5	6	0	-1.460094	0.939850	0.581825
6	6	0	-0.524581	0.612642	1.512559
7	8	0	-2.565749	1.662990	0.842394
8	6	0	-2.895972	2.050824	2.225318
9	6	0	-1.598110	2.289333	3.010095
10	1	0	-1.852179	2.528574	4.049069
11	1	0	-1.097996	3.171762	2.590859
12	6	0	-0.652834	1.086516	2.939087
13	1	0	0.339361	1.350223	3.318907
14	1	0	-1.004017	0.270287	3.585019
15	6	0	1.577870	0.595461	-1.056416
16	1	0	1.843903	0.244277	-2.061204

17	1	0	2.517607	0.802603	-0.537115
18	6	0	0.770945	1.903015	-1.171870
19	1	0	0.642698	2.307941	-0.161796
20	6	0	-0.670194	1.696716	-1.764208
21	8	0	-0.798685	-1.608452	-1.774286
22	6	0	-2.838358	0.166658	-1.365796
23	6	0	-3.614008	-0.916376	-0.678966
24	6	0	-4.941823	-1.092591	-1.118832
25	1	0	-5.310501	-0.449354	-1.912971
26	8	0	1.435719	-0.552159	2.022422
27	6	0	1.645190	-1.939183	-0.443131
28	6	0	3.187177	-1.931423	-0.645385
29	1	0	3.422639	-1.222024	-1.449035
30	6	0	3.627021	-3.302814	-1.155518
31	6	0	3.537880	-4.474749	-0.206655
32	1	0	2.520772	-4.607485	0.183539
33	1	0	4.195072	-4.332779	0.660290
34	1	0	3.831613	-5.405518	-0.701662
35	6	0	4.069758	-3.459857	-2.407559
36	1	0	4.373626	-4.431547	-2.790316
37	1	0	4.145411	-2.623748	-3.099327
38	6	0	3.994012	-1.499420	0.605758
39	1	0	3.729119	-2.140462	1.451745
40	1	0	3.659163	-0.498794	0.906004
41	6	0	5.480453	-1.484440	0.358556
42	1	0	5.774496	-1.068692	-0.607724
43	6	0	6.469192	-1.894297	1.168323
44	6	0	7.914797	-1.777440	0.744511
45	1	0	8.406716	-2.760806	0.737434
46	1	0	8.486372	-1.151250	1.444917
47	1	0	8.013081	-1.343325	-0.255620
48	6	0	6.268106	-2.482444	2.544325
49	1	0	6.748409	-3.468101	2.621891
50	1	0	5.215808	-2.595597	2.815035
51	1	0	6.740360	-1.848775	3.308707
52	1	0	1.214989	-2.460036	-1.302923
53	1	0	1.405428	-2.540910	0.439669
54	6	0	-3.134094	-1.753875	0.338583
55	1	0	-2.118814	-1.663377	0.701332
56	6	0	-3.947192	-2.733269	0.906387

57	1	0	-3.575144	-3.388312	1.687498
58	6	0	-5.258382	-2.897474	0.465863
59	8	0	-6.052598	-3.850242	1.014960
60	1	0	-6.910607	-3.817284	0.556151
61	6	0	-5.749150	-2.063506	-0.558396
62	8	0	-7.055775	-2.307410	-0.919897
63	1	0	-7.295139	-1.744663	-1.671778
64	8	0	-3.358707	0.768525	-2.291819
65	6	0	-3.754191	0.931628	2.823442
66	1	0	-4.074695	1.208212	3.834241
67	1	0	-3.208204	-0.013188	2.883307
68	1	0	-4.643818	0.766434	2.208802
69	6	0	-0.598088	1.270627	-3.249081
70	1	0	-0.098492	2.053910	-3.827903
71	1	0	-1.598294	1.137047	-3.659596
72	1	0	-0.044336	0.339759	-3.400561
73	6	0	-1.463844	3.017879	-1.662287
74	1	0	-1.073030	3.744779	-2.381387
75	1	0	-1.390078	3.459922	-0.663482
76	1	0	-2.516252	2.860347	-1.895556
77	6	0	1.618207	2.958733	-1.942256
78	1	0	1.008429	3.846421	-2.126032
79	1	0	1.882997	2.557896	-2.929337
80	6	0	-3.705823	3.336004	2.067149
81	1	0	-4.044795	3.689568	3.046712
82	1	0	-4.584802	3.160595	1.438971
83	1	0	-3.102006	4.122734	1.604044
84	6	0	2.891256	3.348736	-1.228740
85	1	0	3.730548	2.665243	-1.359617
86	6	0	3.103798	4.428702	-0.459456
87	6	0	2.071210	5.487981	-0.157987
88	1	0	1.899396	5.561262	0.925102
89	1	0	1.106263	5.303142	-0.635953
90	1	0	2.424285	6.477401	-0.480661
91	6	0	4.450516	4.669085	0.180950
92	1	0	4.878920	5.626657	-0.147689
93	1	0	5.165607	3.876582	-0.060567
94	1	0	4.364709	4.727338	1.275170

Conformer 7a-2

Center	Atomic	Atomic	Coordinates (Angstroms)		

Number	Number	Type	X	Y	Z
1	6	0	-1.073084	0.865130	1.171963
2	6	0	-1.215276	1.030110	-0.358217
3	6	0	-0.418799	0.004944	-1.140602
4	6	0	-0.551522	-1.436858	-0.625919
5	6	0	-0.361254	-1.463663	0.883513
6	6	0	-0.604453	-0.410835	1.710350
7	8	0	0.053180	-2.673022	1.306686
8	6	0	0.406554	-2.884507	2.721006
9	6	0	-0.518068	-2.034121	3.603227
10	1	0	-0.228992	-2.166603	4.652122
11	1	0	-1.540762	-2.419267	3.501579
12	6	0	-0.487663	-0.554656	3.208004
13	1	0	-1.310607	-0.008459	3.679866
14	1	0	0.429491	-0.069629	3.569455
15	6	0	-2.698445	0.629967	-0.690855
16	1	0	-2.863997	0.836164	-1.755635
17	1	0	-3.358684	1.298242	-0.128038
18	6	0	-3.061138	-0.835093	-0.381024
19	1	0	-2.995200	-0.974839	0.705247
20	6	0	-2.071310	-1.875877	-1.025750
21	8	0	0.147309	0.247207	-2.190221
22	6	0	0.563568	-2.328477	-1.282369
23	6	0	2.009904	-1.982863	-1.104069
24	6	0	2.933006	-2.848712	-1.724683
25	1	0	2.547986	-3.696347	-2.284755
26	8	0	-1.405024	1.785295	1.915005
27	6	0	-0.978679	2.491177	-0.804618
28	1	0	-1.583340	3.113934	-0.137960
29	1	0	-1.391304	2.599522	-1.813497
30	6	0	0.476661	3.033210	-0.858402
31	6	0	1.238385	2.939620	0.488238
32	1	0	0.681793	3.455852	1.275927
33	6	0	2.658835	3.433880	0.383737
34	1	0	3.166579	3.155155	-0.541521
35	6	0	3.360934	4.150148	1.276205
36	6	0	4.791052	4.548131	0.994588
37	1	0	4.906240	5.641475	1.005155
38	1	0	5.133102	4.181875	0.021496
39	1	0	5.472569	4.158373	1.764429

40	6	0	2.834021	4.617188	2.611895
41	1	0	3.422374	4.182469	3.432721
42	1	0	1.787238	4.353543	2.779924
43	1	0	2.930560	5.707676	2.709276
44	1	0	1.257174	1.883099	0.796497
45	6	0	0.458123	4.457845	-1.407137
46	6	0	-0.058746	5.560879	-0.513217
47	1	0	0.588071	5.693987	0.363088
48	1	0	-1.065220	5.344585	-0.132954
49	1	0	-0.098688	6.515113	-1.047748
50	6	0	0.879301	4.713961	-2.650503
51	1	0	0.856524	5.718604	-3.067273
52	1	0	1.261863	3.928854	-3.298478
53	1	0	1.020027	2.420466	-1.584529
54	6	0	2.507085	-0.887469	-0.384260
55	1	0	1.835926	-0.197974	0.110218
56	6	0	3.877139	-0.653969	-0.280317
57	1	0	4.258682	0.201091	0.268388
58	6	0	4.780554	-1.514159	-0.900370
59	8	0	6.115502	-1.291334	-0.807622
60	1	0	6.564826	-1.985419	-1.321506
61	6	0	4.291715	-2.618860	-1.626328
62	8	0	5.267287	-3.406188	-2.197515
63	1	0	4.852429	-4.104075	-2.726730
64	8	0	0.269236	-3.319242	-1.932643
65	6	0	1.885602	-2.522651	2.887410
66	1	0	2.204456	-2.723868	3.916339
67	1	0	2.074121	-1.468117	2.670375
68	1	0	2.500350	-3.120611	2.208582
69	6	0	-2.234565	-1.894718	-2.563182
70	1	0	-3.262518	-2.175157	-2.813672
71	1	0	-1.568637	-2.629558	-3.013180
72	1	0	-2.029511	-0.925256	-3.025680
73	6	0	-2.373161	-3.284589	-0.471672
74	1	0	-3.339133	-3.637856	-0.846929
75	1	0	-2.418240	-3.288884	0.622151
76	1	0	-1.613916	-3.997919	-0.791182
77	6	0	-4.552060	-1.071315	-0.752081
78	1	0	-4.776611	-2.142708	-0.669467
79	1	0	-4.721357	-0.801877	-1.798880

80	6	0	0.180107	-4.379076	2.939744
81	1	0	0.455004	-4.655663	3.963218
82	1	0	0.791293	-4.965351	2.246426
83	1	0	-0.870462	-4.641388	2.779831
84	6	0	-5.494742	-0.324411	0.156862
85	1	0	-5.373497	-0.561590	1.216246
86	6	0	-6.448182	0.564770	-0.163480
87	6	0	-6.774471	1.020279	-1.565209
88	1	0	-7.812969	0.768061	-1.821877
89	1	0	-6.127543	0.580802	-2.327755
90	1	0	-6.693743	2.113166	-1.644713
91	6	0	-7.308113	1.191192	0.909471
92	1	0	-7.216021	2.286329	0.900668
93	1	0	-7.038713	0.836676	1.909305
94	1	0	-8.371813	0.968260	0.744211

Conformer 8a-1

Center	Atomic	Atomic	Coordinates (Angstroms)		
Number	Number	Type	X	Y	Z
1	6	0	-0.543055	0.024692	-0.921251
2	6	0	1.729156	-0.783176	-1.765441
3	6	0	2.165829	-0.763656	-0.318008
4	6	0	1.403049	-0.420965	0.756854
5	6	0	0.051660	0.111603	0.519412
6	6	0	2.122910	-0.383110	4.564237
7	1	0	2.248916	-1.454244	4.680640
8	6	0	1.880014	0.127649	3.277344
9	6	0	1.862270	-0.826690	2.138368
10	6	0	2.197979	0.472258	5.657805
11	1	0	2.377138	0.069100	6.650686
12	6	0	2.045230	1.850826	5.477514
13	1	0	2.106738	2.520315	6.331543
14	6	0	1.812995	2.367818	4.201558
15	1	0	1.696355	3.438392	4.058917
16	6	0	1.723892	1.510682	3.106657
17	1	0	1.531295	1.918682	2.122181
18	8	0	2.231953	-1.990710	2.303755
19	8	0	-0.629611	0.573698	1.428108
20	6	0	0.218307	-1.028901	-1.735443
21	8	0	-0.309691	-1.940917	-2.336665

22	6	0	-0.309799	1.369891	-1.681841
23	1	0	-0.803391	2.174378	-1.135252
24	1	0	-0.851308	1.285373	-2.630960
25	6	0	1.983854	0.567509	-2.583231
26	6	0	1.156828	1.768581	-1.987258
27	6	0	1.863600	2.572586	-0.849777
28	1	0	2.788491	2.986100	-1.260336
29	1	0	2.171149	1.909753	-0.036964
30	6	0	1.022304	3.683569	-0.270084
31	1	0	0.286120	3.369882	0.470544
32	6	0	1.078402	4.995102	-0.554676
33	6	0	2.020144	5.622095	-1.554027
34	1	0	2.645271	6.386152	-1.071471
35	1	0	1.456080	6.138535	-2.343001
36	1	0	2.684416	4.901870	-2.037924
37	6	0	0.162533	5.975819	0.138794
38	1	0	0.738984	6.734213	0.687250
39	1	0	-0.507111	5.479437	0.847919
40	1	0	-0.455126	6.522210	-0.587652
41	6	0	3.489399	0.893498	-2.636441
42	1	0	3.938353	0.992122	-1.644891
43	1	0	3.649207	1.834913	-3.175017
44	1	0	4.039880	0.118716	-3.180431
45	6	0	1.503017	0.347699	-4.039540
46	1	0	0.434379	0.125495	-4.105111
47	1	0	2.035017	-0.473692	-4.528543
48	1	0	1.694084	1.253767	-4.625221
49	6	0	-2.040816	-0.387689	-0.855632
50	1	0	-2.320189	-0.741477	-1.852335
51	1	0	-2.087471	-1.274111	-0.214311
52	6	0	-3.089681	0.621732	-0.332474
53	6	0	-4.373116	-0.064515	0.115996
54	1	0	-5.058400	0.592453	0.654881
55	6	0	-4.764021	-1.322747	-0.115826
56	1	0	-4.127619	-2.007131	-0.674466
57	6	0	-6.091776	-1.903989	0.357009
58	6	0	-5.978277	-3.410730	0.616213
59	1	0	-5.697847	-3.936208	-0.303742
60	1	0	-6.939489	-3.802422	0.963426
61	1	0	-5.225490	-3.616186	1.380393

62	6	0	-7.207986	-1.600105	-0.655349
63	1	0	-6.956911	-2.031191	-1.630170
64	1	0	-7.329708	-0.519560	-0.776170
65	1	0	-8.156782	-2.028985	-0.315928
66	6	0	-3.503651	1.727024	-1.313251
67	6	0	-3.535200	3.005338	-0.918697
68	1	0	-3.217298	3.305306	0.077211
69	1	0	-3.883267	3.798950	-1.575870
70	6	0	-3.967942	1.317308	-2.690649
71	1	0	-3.153675	0.888428	-3.288976
72	1	0	-4.370227	2.172356	-3.242287
73	1	0	-4.748576	0.549003	-2.629931
74	6	0	2.542248	-2.006669	-2.265401
75	1	0	2.901814	-1.889969	-3.288057
76	1	0	1.890676	-2.882028	-2.233770
77	6	0	3.709810	-2.165726	-1.271246
78	8	0	3.408669	-1.231615	-0.182622
79	6	0	3.829816	-3.564331	-0.606759
80	6	0	4.062575	-4.635895	-1.676025
81	1	0	4.977904	-4.437096	-2.245310
82	1	0	3.220484	-4.693322	-2.371807
83	1	0	4.161717	-5.611752	-1.191351
84	6	0	4.976920	-3.569209	0.417365
85	1	0	5.942571	-3.357622	-0.057677
86	1	0	5.034112	-4.554836	0.889871
87	1	0	4.806421	-2.822382	1.198277
88	8	0	2.593755	-3.875215	0.023529
89	1	0	2.496173	-3.307726	0.817154
90	1	0	4.672371	-1.851539	-1.686027
91	1	0	1.078754	2.495183	-2.807837
92	1	0	-2.671568	1.111259	0.551223
93	8	0	-6.558042	-1.215284	1.540313
94	8	0	-5.671707	-1.500449	2.653067
95	1	0	-4.922547	-0.906008	2.450031

Conformer 8a-2

Center	Atomic	Atomic	Coordinates (Angstroms)		
Number	Number	Type	X	Y	Z
1	6	0	-0.332221	0.324901	-1.098634
2	6	0	2.145968	0.284515	-1.709902
3	6	0	2.367594	-0.365849	-0.363345

4	6	0	1.412520	-0.685502	0.553683
5	6	0	0.027391	-0.253660	0.305460
6	6	0	1.579631	-2.467061	3.990230
7	1	0	1.901981	-3.433268	3.617002
8	6	0	1.417941	-1.414167	3.073095
9	6	0	1.750419	-1.671675	1.647592
10	6	0	1.330122	-2.265792	5.343204
11	1	0	1.449267	-3.086663	6.044966
12	6	0	0.929188	-1.005742	5.799091
13	1	0	0.737049	-0.846895	6.857029
14	6	0	0.775057	0.048214	4.896497
15	1	0	0.466175	1.028238	5.248906
16	6	0	1.011765	-0.155389	3.538469
17	1	0	0.879048	0.664216	2.843190
18	8	0	2.321451	-2.715747	1.327176
19	8	0	-0.853358	-0.381935	1.148698
20	6	0	0.723529	-0.101582	-2.124980
21	8	0	0.472412	-0.644851	-3.180141
22	6	0	-0.266607	1.886486	-1.075530
23	1	0	-0.978378	2.258878	-0.337360
24	1	0	-0.646962	2.225368	-2.045746
25	6	0	2.239661	1.881153	-1.730022
26	6	0	1.120671	2.535612	-0.835855
27	6	0	1.493294	2.736659	0.667886
28	1	0	2.365575	3.394285	0.709353
29	1	0	1.812011	1.793711	1.119502
30	6	0	0.378471	3.316018	1.504629
31	1	0	-0.375595	2.600053	1.833149
32	6	0	0.213774	4.592703	1.888472
33	6	0	1.140911	5.728166	1.528495
34	1	0	1.535113	6.209694	2.434169
35	1	0	0.598213	6.507736	0.976187
36	1	0	1.991111	5.417566	0.916457
37	6	0	-0.962957	4.995822	2.745423
38	1	0	-0.627515	5.441584	3.692547
39	1	0	-1.608525	4.144006	2.980753
40	1	0	-1.574758	5.758771	2.244052
41	6	0	3.644663	2.349035	-1.303082
42	1	0	3.933265	1.981529	-0.315041
43	1	0	3.681995	3.444443	-1.280835

44	1	0	4.403286	2.019931	-2.021071
45	6	0	2.012249	2.360697	-3.186085
46	1	0	1.022591	2.099174	-3.570360
47	1	0	2.748632	1.939696	-3.876989
48	1	0	2.111239	3.451077	-3.229213
49	6	0	-1.721809	-0.211125	-1.543359
50	1	0	-1.779948	-0.105660	-2.630542
51	1	0	-1.720853	-1.290509	-1.359341
52	6	0	-3.000829	0.377375	-0.877738
53	6	0	-4.119202	-0.642081	-0.986657
54	1	0	-4.484707	-0.870103	-1.989992
55	6	0	-4.667546	-1.278976	0.053057
56	1	0	-4.298391	-1.078941	1.059176
57	6	0	-5.798714	-2.292933	-0.063306
58	6	0	-6.636608	-2.341850	1.219195
59	1	0	-6.014921	-2.642404	2.070171
60	1	0	-7.448987	-3.066428	1.105846
61	1	0	-7.073885	-1.363598	1.431528
62	6	0	-5.254543	-3.683841	-0.423783
63	1	0	-4.553709	-4.021929	0.346350
64	1	0	-4.724304	-3.650487	-1.380068
65	1	0	-6.074193	-4.406597	-0.496167
66	6	0	-3.494357	1.705486	-1.461179
67	6	0	-3.860029	2.705793	-0.650690
68	1	0	-3.776211	2.625630	0.430708
69	1	0	-4.261264	3.639675	-1.037608
70	6	0	-3.629932	1.831153	-2.961599
71	1	0	-2.655036	1.809160	-3.465236
72	1	0	-4.123334	2.768887	-3.234370
73	1	0	-4.214899	1.006706	-3.389391
74	6	0	3.245767	-0.439646	-2.530684
75	1	0	3.705142	0.200375	-3.284277
76	1	0	2.785195	-1.287929	-3.040398
77	6	0	4.279973	-0.929625	-1.498166
78	8	0	3.648517	-0.702263	-0.195022
79	6	0	4.601690	-2.447687	-1.553656
80	6	0	5.192436	-2.810063	-2.919412
81	1	0	6.117493	-2.255012	-3.113674
82	1	0	4.483208	-2.605762	-3.726856
83	1	0	5.420652	-3.879899	-2.939237

84	6	0	5.577628	-2.826001	-0.427427
85	1	0	6.531395	-2.293347	-0.524424
86	1	0	5.777610	-3.901244	-0.469382
87	1	0	5.151049	-2.592499	0.552343
88	8	0	3.385822	-3.174502	-1.429028
89	1	0	3.063352	-3.090216	-0.507024
90	1	0	5.200699	-0.338373	-1.499289
91	1	0	1.015031	3.561012	-1.216344
92	1	0	-2.794801	0.524539	0.184161
93	8	0	-6.632169	-1.982262	-1.205763
94	8	0	-7.306257	-0.715589	-0.986406
95	1	0	-6.569312	-0.088236	-1.129729

Conformer 9a-1

Center	Atomic	Atomic	Coordinates (Angstroms)		
Number	Number	Type	X	Y	Z
1	6	0	1.671495	-0.618002	-0.835096
2	6	0	-0.745906	-0.991397	-1.589396
3	6	0	-1.102103	0.227204	-0.743596
4	6	0	-0.267602	0.857702	0.119904
5	6	0	1.105197	0.311699	0.280004
6	6	0	-2.140796	3.785806	1.811204
7	1	0	-1.442995	4.562204	1.515704
8	6	0	-1.838499	2.456405	1.473904
9	6	0	-0.554099	2.197403	0.749604
10	6	0	-3.306196	4.090008	2.507704
11	1	0	-3.534293	5.122409	2.757904
12	6	0	-4.180598	3.067410	2.888704
13	1	0	-5.089097	3.303912	3.436404
14	6	0	-3.883900	1.741309	2.568104
15	1	0	-4.556002	0.943111	2.871204
16	6	0	-2.720701	1.438007	1.861004
17	1	0	-2.495503	0.405607	1.617304
18	8	0	0.289502	3.077501	0.650904
19	8	0	1.789697	0.591498	1.250204
20	6	0	0.703695	-0.705800	-2.018896
21	8	0	1.032195	-0.601101	-3.182896
22	6	0	1.724192	-2.066502	-0.271696
23	1	0	2.433892	-2.087803	0.559904
24	1	0	2.146091	-2.713003	-1.048396

25	6	0	-0.776209	-2.429297	-0.873296
26	6	0	0.359191	-2.620999	0.206904
27	6	0	0.020492	-2.209298	1.678404
28	1	0	-0.637206	-1.339297	1.715504
29	1	0	0.955792	-1.879600	2.150604
30	6	0	-0.539311	-3.348397	2.498704
31	1	0	-0.066612	-4.313598	2.301404
32	6	0	-1.482510	-3.320895	3.452904
33	6	0	-2.224308	-2.084794	3.898204
34	1	0	-2.049208	-1.896194	4.966504
35	1	0	-3.309708	-2.217492	3.783104
36	1	0	-1.930406	-1.182495	3.357704
37	6	0	-1.870513	-4.581095	4.190604
38	1	0	-1.693113	-4.478695	5.270504
39	1	0	-1.309515	-5.452096	3.837304
40	1	0	-2.942313	-4.795492	4.072304
41	6	0	-2.159709	-2.700894	-0.255996
42	1	0	-2.447608	-1.941093	0.476304
43	1	0	-2.154611	-3.664594	0.262404
44	1	0	-2.942509	-2.742892	-1.018596
45	6	0	-0.525411	-3.490297	-1.976796
46	1	0	0.449389	-3.369899	-2.459596
47	1	0	-1.284811	-3.458096	-2.762796
48	1	0	-0.559813	-4.491797	-1.534396
49	6	0	3.039096	-0.077705	-1.342296
50	1	0	3.257895	-0.588605	-2.284096
51	1	0	2.872498	0.970996	-1.611196
52	6	0	4.255596	-0.144407	-0.393896
53	6	0	5.300298	0.960191	-0.771396
54	1	0	5.704498	0.726890	-1.764196
55	1	0	6.145398	0.873889	-0.075496
56	6	0	4.971193	-1.493908	-0.332296
57	6	0	4.751101	2.369592	-0.772896
58	1	0	4.545402	2.793792	-1.756096
59	6	0	5.189892	-2.273209	-1.397996
60	1	0	4.830992	-2.021208	-2.392396
61	1	0	5.752290	-3.200210	-1.315196
62	6	0	5.507092	-1.871810	1.029204
63	1	0	6.160294	-1.084511	1.429504
64	1	0	6.080191	-2.804011	1.000604

65	1	0	4.691392	-1.990808	1.755904
66	6	0	-1.762506	-0.874695	-2.759696
67	1	0	-2.454107	-1.716493	-2.770096
68	1	0	-1.231106	-0.856396	-3.713396
69	6	0	-2.516903	0.438607	-2.514796
70	1	0	-2.040501	1.264006	-3.057296
71	6	0	-4.027403	0.528710	-2.795896
72	6	0	-4.861605	-0.469989	-1.980596
73	1	0	-4.649507	-1.510889	-2.247796
74	1	0	-5.921804	-0.279786	-2.171996
75	1	0	-4.679004	-0.347089	-0.907996
76	6	0	-4.277903	0.370710	-4.297396
77	1	0	-5.335103	0.559312	-4.507596
78	1	0	-4.029505	-0.638090	-4.642996
79	1	0	-3.686402	1.095609	-4.866096
80	8	0	-4.441400	1.863011	-2.477096
81	1	0	-4.194900	2.024710	-1.550896
82	8	0	-2.303202	0.730906	-1.093296
83	1	0	0.501689	-3.708699	0.252604
84	1	0	3.903996	0.095694	0.611904
85	6	0	4.443103	3.122193	0.295104
86	6	0	3.806405	4.480994	0.132704
87	1	0	4.374007	5.255993	0.667004
88	1	0	2.790905	4.478696	0.551804
89	1	0	3.734706	4.773694	-0.919996
90	6	0	4.642002	2.680392	1.724004
91	1	0	5.112903	3.476691	2.316204
92	1	0	5.263100	1.784191	1.809004
93	1	0	3.673501	2.459294	2.191604

Conformer 9a-3

Center	Atomic	Atomic	Coordinates (Angstroms)		
Number	Number	Type	X	Y	Z
1	6	0	1.593002	0.970102	-0.269106
2	6	0	-0.051095	0.176795	1.506394
3	6	0	-0.784392	-0.540607	0.380994
4	6	0	-0.552693	-0.379206	-0.945306
5	6	0	0.555604	0.526698	-1.345106
6	6	0	-3.125885	-2.517416	-2.874906
7	1	0	-2.400383	-3.091713	-3.441406
8	6	0	-2.655289	-1.491114	-2.039706

9	6	0	-1.175790	-1.243009	-2.012706
10	6	0	-4.488484	-2.783021	-2.970406
11	1	0	-4.840181	-3.584823	-3.613806
12	6	0	-5.403887	-2.016025	-2.243106
13	1	0	-6.468586	-2.219729	-2.320806
14	6	0	-4.947891	-0.985023	-1.419606
15	1	0	-5.655893	-0.378926	-0.860806
16	6	0	-3.581092	-0.727618	-1.315506
17	1	0	-3.235695	0.076383	-0.675306
18	8	0	-0.458688	-1.749306	-2.863206
19	8	0	0.673902	0.943098	-2.485206
20	6	0	1.419605	0.238801	1.052194
21	8	0	2.331006	-0.172696	1.742594
22	6	0	1.254596	2.454500	0.094394
23	1	0	1.404694	3.057101	-0.808306
24	1	0	2.005495	2.785703	0.821694
25	6	0	-0.522401	1.686894	1.814694
26	6	0	-0.163304	2.702495	0.659694
27	6	0	-1.232805	2.932291	-0.457706
28	1	0	-1.780102	2.017089	-0.688906
29	1	0	-0.693306	3.181793	-1.381706
30	6	0	-2.180710	4.067987	-0.144606
31	1	0	-1.722013	4.898089	0.397994
32	6	0	-3.469910	4.210183	-0.488706
33	6	0	-4.267606	3.200480	-1.276206
34	1	0	-4.628208	3.644878	-2.214306
35	1	0	-5.163405	2.889076	-0.719906
36	1	0	-3.699903	2.305382	-1.538506
37	6	0	-4.236115	5.456480	-0.111706
38	1	0	-4.612417	5.975378	-1.004506
39	1	0	-3.619017	6.161782	0.454094
40	1	0	-5.117614	5.210976	0.497694
41	6	0	-2.029401	1.734888	2.126394
42	1	0	-2.640599	1.330186	1.314894
43	1	0	-2.344005	2.771487	2.281994
44	1	0	-2.273599	1.178887	3.036094
45	6	0	0.240298	2.157696	3.080194
46	1	0	1.324598	2.174401	2.935494
47	1	0	0.038300	1.520896	3.945994
48	1	0	-0.081006	3.171695	3.342394

49	6	0	3.034502	0.934507	-0.836706
50	1	0	2.995000	1.435507	-1.810306
51	1	0	3.653600	1.558009	-0.181806
52	6	0	3.751907	-0.433490	-0.971006
53	6	0	5.185207	-0.224785	-1.549706
54	1	0	5.105105	0.237715	-2.542806
55	1	0	5.625510	-1.215483	-1.709106
56	6	0	2.978611	-1.463593	-1.781006
57	6	0	6.089803	0.626018	-0.693406
58	1	0	5.990099	1.703218	-0.833806
59	6	0	2.424015	-2.517395	-1.166806
60	1	0	2.524716	-2.669195	-0.094006
61	1	0	1.851418	-3.259897	-1.715806
62	6	0	2.880610	-1.286094	-3.278006
63	1	0	2.521207	-0.286195	-3.543506
64	1	0	2.185613	-2.009196	-3.710506
65	1	0	3.861911	-1.419190	-3.753506
66	6	0	-0.278791	-0.782705	2.706894
67	1	0	-0.876493	-0.309808	3.485394
68	1	0	0.681410	-1.069402	3.140794
69	6	0	-1.020787	-1.990908	2.121094
70	1	0	-0.312784	-2.774406	1.825494
71	6	0	-2.141484	-2.675212	2.924494
72	6	0	-3.290688	-1.727317	3.298394
73	1	0	-2.979991	-0.950816	4.005594
74	1	0	-4.088186	-2.312320	3.766094
75	1	0	-3.698990	-1.238218	2.407994
76	6	0	-1.546382	-3.344710	4.165194
77	1	0	-2.324680	-3.925413	4.669594
78	1	0	-1.152185	-2.605609	4.870294
79	1	0	-0.741279	-4.031607	3.884494
80	8	0	-2.651180	-3.744114	2.118394
81	1	0	-2.935882	-3.354015	1.274794
82	8	0	-1.602589	-1.496710	0.868794
83	1	0	-0.090308	3.671795	1.170294
84	1	0	3.872409	-0.830290	0.040294
85	6	0	6.989205	0.212322	0.213694
86	6	0	7.821801	1.205525	0.989294
87	1	0	8.895402	1.043629	0.815894
88	1	0	7.663602	1.096524	2.071694

89	1	0	7.586697	2.238824	0.714494
90	6	0	7.262710	-1.233077	0.551694
91	1	0	8.323611	-1.475573	0.396894
92	1	0	6.666513	-1.934179	-0.036706
93	1	0	7.052611	-1.426578	1.612794

Conformer 10a-1

Center	Atomic	Atomic	Coordinates (Angstroms)		
Number	Number	Type	X	Y	Z
1	6	0	-0.205313	0.944125	-1.016485
2	6	0	2.202701	-0.016640	-0.931155
3	6	0	1.857587	-0.563526	0.473219
4	6	0	0.489376	-0.462368	0.960801
5	6	0	-0.477692	0.226532	0.289501
6	6	0	-0.242039	-3.326054	1.266333
7	1	0	-0.125824	-2.887343	0.280930
8	6	0	-0.094436	-2.524920	2.409519
9	6	0	0.215495	-1.070646	2.318673
10	6	0	-0.542207	-4.682207	1.393265
11	1	0	-0.656906	-5.296072	0.505424
12	6	0	-0.693789	-5.247980	2.661090
13	1	0	-0.925564	-6.304338	2.758943
14	6	0	-0.547763	-4.456077	3.805896
15	1	0	-0.665408	-4.897629	4.790701
16	6	0	-0.251951	-3.102892	3.680990
17	1	0	-0.136135	-2.472777	4.556173
18	8	0	0.266343	-0.366114	3.321387
19	6	0	0.888774	0.125915	-1.720340
20	8	0	0.727446	-0.340884	-2.832311
21	6	0	0.387872	2.380390	-0.795772
22	1	0	-0.315488	2.955145	-0.184967
23	1	0	0.381662	2.845092	-1.787693
24	6	0	2.824452	1.462861	-0.821417
25	6	0	1.804012	2.489529	-0.189029
26	1	0	2.182561	3.462214	-0.531277
27	6	0	1.775408	2.633804	1.364760
28	1	0	1.286806	1.791186	1.852954
29	1	0	2.809215	2.642971	1.727219
30	6	0	1.137876	3.940958	1.767705
31	1	0	1.584079	4.816405	1.290270

32	8	0	2.718451	-1.120564	1.160224
33	6	0	0.128035	4.167539	2.624173
34	6	0	-0.342802	5.577561	2.897354
35	1	0	-1.409032	5.689094	2.659668
36	1	0	0.215205	6.317291	2.316538
37	1	0	-0.237564	5.828593	3.961236
38	6	0	-0.614657	3.104321	3.395328
39	1	0	-0.525334	3.288060	4.474565
40	1	0	-0.266138	2.089834	3.198853
41	1	0	-1.688292	3.147773	3.168339
42	6	0	3.155508	1.957855	-2.251826
43	1	0	3.884671	1.304932	-2.736806
44	1	0	3.596219	2.958273	-2.197285
45	1	0	2.278152	2.018553	-2.902240
46	6	0	4.142632	1.478389	-0.022671
47	1	0	4.932533	0.962169	-0.568330
48	1	0	4.051114	1.004670	0.955729
49	1	0	4.460226	2.516743	0.124757
50	6	0	3.074452	-1.067895	-1.700688
51	1	0	2.510199	-2.009580	-1.633034
52	1	0	3.038587	-0.801202	-2.756198
53	6	0	4.490791	-1.349941	-1.270630
54	1	0	4.622782	-1.529830	-0.208238
55	6	0	5.565883	-1.483263	-2.066958
56	6	0	6.907285	-1.858210	-1.480435
57	1	0	7.661982	-1.090830	-1.699077
58	1	0	7.283335	-2.792448	-1.918734
59	1	0	6.857124	-1.988030	-0.395801
60	6	0	5.578269	-1.299550	-3.565429
61	1	0	5.860608	-2.235972	-4.064644
62	1	0	6.332810	-0.557305	-3.856509
63	1	0	4.618188	-0.982229	-3.976245
64	8	0	-1.687514	0.302237	0.849813
65	6	0	-2.959727	0.347912	0.063603
66	6	0	-2.624473	0.122640	-1.429381
67	1	0	-2.266279	-0.913260	-1.519807
68	6	0	-1.489763	1.060783	-1.865006
69	1	0	-1.846282	2.095055	-1.834571
70	1	0	-1.226528	0.847321	-2.903447
71	6	0	-3.818924	0.296049	-2.403519

72	1	0	-4.225057	1.307019	-2.305184
73	1	0	-3.383006	0.252194	-3.412525
74	6	0	-4.903410	-0.746219	-2.324801
75	1	0	-4.539845	-1.774757	-2.302991
76	6	0	-6.234777	-0.567091	-2.335588
77	6	0	-7.170718	-1.752648	-2.311319
78	1	0	-7.818528	-1.759859	-3.197807
79	1	0	-7.839333	-1.708347	-1.441586
80	1	0	-6.629028	-2.701844	-2.277781
81	6	0	-6.928942	0.772208	-2.384072
82	1	0	-7.540606	0.853764	-3.292023
83	1	0	-6.243024	1.621038	-2.369327
84	1	0	-7.619217	0.878405	-1.537531
85	6	0	-3.597603	1.700973	0.378944
86	1	0	-3.649534	1.833949	1.462701
87	1	0	-4.616356	1.742490	-0.014223
88	1	0	-3.031093	2.535548	-0.040529
89	6	0	-3.770411	-0.791927	0.681372
90	1	0	-4.769470	-0.829453	0.243749
91	1	0	-3.862499	-0.638553	1.760221
92	1	0	-3.278568	-1.753250	0.508510

Conformer 10a-3

Center	Atomic	Atomic	Coordinates (Angstroms)		
Number	Number	Type	X	Y	Z
1	6	0	0.611580	-0.920146	-0.189229
2	6	0	-1.982920	-0.962094	-0.346483
3	6	0	-1.966297	0.523187	0.086198
4	6	0	-0.689122	1.211965	0.203859
5	6	0	0.508354	0.568633	0.083235
6	6	0	-0.564808	3.377508	-1.831087
7	1	0	-0.564012	2.335059	-2.131552
8	6	0	-0.649801	3.711994	-0.470540
9	6	0	-0.738716	2.673207	0.593321
10	6	0	-0.487143	4.380506	-2.797218
11	1	0	-0.424087	4.115391	-3.847942
12	6	0	-0.492189	5.722997	-2.412115
13	1	0	-0.431012	6.503124	-3.165001
14	6	0	-0.576924	6.064828	-1.057528
15	1	0	-0.580815	7.108827	-0.759646
16	6	0	-0.656678	5.065962	-0.093134

17	1	0	-0.723765	5.310705	0.961397
18	8	0	-0.864598	2.979286	1.774607
19	6	0	-0.635600	-1.275142	-1.020551
20	8	0	-0.549552	-1.799400	-2.114978
21	6	0	0.515308	-1.775212	1.122238
22	1	0	1.319674	-1.470389	1.798688
23	1	0	0.750311	-2.798558	0.810893
24	6	0	-2.078369	-1.925301	0.940465
25	6	0	-0.825285	-1.770457	1.889829
26	1	0	-0.823293	-2.709345	2.460148
27	6	0	-0.881651	-0.671673	2.995687
28	1	0	-0.785174	0.335278	2.590858
29	1	0	-1.868931	-0.717902	3.468976
30	6	0	0.149209	-0.930849	4.066883
31	1	0	0.138606	-1.951821	4.455445
32	8	0	-3.022123	1.130745	0.281137
33	6	0	1.039328	-0.085051	4.611712
34	6	0	1.967086	-0.550686	5.710179
35	1	0	3.017821	-0.409111	5.424456
36	1	0	1.818897	-1.606535	5.953461
37	1	0	1.816649	0.035110	6.626821
38	6	0	1.201280	1.367085	4.235179
39	1	0	2.239337	1.573822	3.942683
40	1	0	0.998453	2.008164	5.103824
41	1	0	0.547245	1.687971	3.423495
42	6	0	-2.111188	-3.393537	0.446578
43	1	0	-2.980065	-3.577019	-0.189478
44	1	0	-2.187705	-4.064762	1.307774
45	1	0	-1.218852	-3.679101	-0.117610
46	6	0	-3.373713	-1.689456	1.741429
47	1	0	-4.245312	-2.012800	1.172294
48	1	0	-3.521579	-0.642254	2.008783
49	1	0	-3.337970	-2.279783	2.664054
50	6	0	-3.088259	-1.173551	-1.437772
51	1	0	-2.885562	-0.406881	-2.200070
52	1	0	-2.871116	-2.118514	-1.933876
53	6	0	-4.547970	-1.085144	-1.075050
54	1	0	-4.825299	-0.211545	-0.493502
55	6	0	-5.530087	-1.916880	-1.464338
56	6	0	-6.964479	-1.633538	-1.082133

57	1	0	-7.390927	-2.465076	-0.505134
58	1	0	-7.596613	-1.522066	-1.973394
59	1	0	-7.055404	-0.721902	-0.485102
60	6	0	-5.348522	-3.165052	-2.293864
61	1	0	-5.896718	-3.079789	-3.241532
62	1	0	-5.767394	-4.036993	-1.774897
63	1	0	-4.306700	-3.384552	-2.533545
64	8	0	1.607537	1.307310	0.267235
65	6	0	2.941478	0.952452	-0.298437
66	6	0	2.691081	-0.051309	-1.444358
67	1	0	2.036216	0.486456	-2.145399
68	6	0	1.923634	-1.273577	-0.932453
69	1	0	2.569570	-1.860882	-0.275025
70	1	0	1.666449	-1.916753	-1.777308
71	6	0	3.932142	-0.463304	-2.289460
72	1	0	3.534265	-0.925968	-3.197475
73	1	0	4.441499	0.449028	-2.622870
74	6	0	4.945424	-1.379152	-1.647823
75	1	0	5.606575	-0.911428	-0.920191
76	6	0	5.152938	-2.684024	-1.895092
77	6	0	6.249596	-3.436594	-1.178860
78	1	0	6.971181	-3.853170	-1.893907
79	1	0	5.841240	-4.289613	-0.621346
80	1	0	6.793922	-2.798662	-0.477302
81	6	0	4.364710	-3.520260	-2.873049
82	1	0	5.033464	-3.974074	-3.615668
83	1	0	3.596983	-2.959452	-3.408446
84	1	0	3.870986	-4.351926	-2.354212
85	6	0	3.781520	0.458872	0.881623
86	1	0	3.706551	1.182126	1.698135
87	1	0	4.832829	0.379568	0.599423
88	1	0	3.452626	-0.512721	1.255073
89	6	0	3.467394	2.296863	-0.803712
90	1	0	4.495703	2.196826	-1.159798
91	1	0	3.458997	3.030100	0.007075
92	1	0	2.848113	2.675825	-1.621302

Conformer 11a-1

Center	Atomic	Atomic	Coordinates (Angstroms)		
Number	Number	Type	X	Y	Z

1	6	0	-0.410412	1.861701	-1.199204
2	6	0	-0.799320	3.327899	-0.813904
3	6	0	-0.278010	1.605602	-2.783304
4	6	0	0.974190	1.535009	-0.639704
5	6	0	-1.515306	0.917895	-0.687204
6	6	0	-1.192097	-0.528904	-0.241204
7	6	0	0.311404	-0.694095	0.096496
8	6	0	1.376899	0.055512	-0.725204
9	6	0	0.055299	0.086904	-3.001604
10	8	0	-2.662709	1.324588	-0.677804
11	6	0	1.312902	-0.358889	-2.233104
12	8	0	0.656808	-1.401292	1.025496
13	8	0	1.711885	2.397514	-0.206704
14	6	0	2.782300	-0.146180	-0.117804
15	6	0	-1.119595	-0.914603	-2.854704
16	6	0	-1.558613	2.006594	-3.545904
17	6	0	-1.461891	-1.524205	-1.464004
18	6	0	-2.866687	-2.231514	-1.560004
19	8	0	-3.343485	-2.679916	-0.268504
20	8	0	-2.227182	-3.172610	0.525996
21	6	0	-2.067687	-2.224209	1.597996
22	6	0	-2.024096	-0.791008	1.052996
23	6	0	-2.745180	-3.484313	-2.447804
24	6	0	-4.037693	-1.350321	-2.021604
25	6	0	-3.155986	-2.427115	2.695196
26	6	0	-3.247477	-3.916516	3.067296
27	6	0	-2.831691	-1.592013	3.939896
28	8	0	-4.433889	-1.961223	2.246196
29	6	0	0.882185	2.447309	-3.373004
30	1	0	0.320299	0.029706	-4.065804
31	1	0	1.423308	-1.446788	-2.306804
32	1	0	2.204099	0.060417	-2.705304
33	6	0	3.410309	-1.549276	-0.299904
34	1	0	2.597613	-2.285181	-0.335904
35	6	0	4.232911	-1.943771	0.972196
36	1	0	4.628017	-2.959369	0.820396
37	1	0	3.523211	-2.011075	1.801996
38	6	0	5.374205	-1.019164	1.295996
39	1	0	6.132405	-0.947060	0.515896
40	6	0	4.243510	-1.750771	-1.565704

41	6	0	4.238518	-3.162271	-2.107504
42	1	0	4.543123	-3.886669	-1.340604
43	1	0	4.911419	-3.274067	-2.963204
44	1	0	3.229120	-3.458977	-2.426604
45	1	0	2.709199	0.072320	0.950296
46	1	0	3.434296	0.629424	-0.524304
47	1	0	-0.884890	-1.757702	-3.512904
48	1	0	-2.010198	-0.450608	-3.280104
49	1	0	-1.641519	3.091994	-3.597504
50	1	0	-2.474710	1.629989	-3.087504
51	1	0	-1.501410	1.616995	-4.570004
52	1	0	-0.766587	-2.349501	-1.286204
53	1	0	-1.097086	-2.514603	2.005796
54	1	0	-1.613300	-0.148406	1.838696
55	1	0	-3.042798	-0.448015	0.880196
56	1	0	-3.705777	-4.008419	-2.454104
57	1	0	-1.988976	-4.167008	-2.046804
58	1	0	-2.485681	-3.240311	-3.482404
59	1	0	-4.070898	-0.403921	-1.479004
60	1	0	-4.972589	-1.891826	-1.842304
61	1	0	-3.984094	-1.146320	-3.094704
62	1	0	-3.508274	-4.520717	2.194096
63	1	0	-4.020276	-4.050520	3.830596
64	1	0	-2.296275	-4.291710	3.464396
65	1	0	-3.589490	-1.779418	4.707296
66	1	0	-2.847297	-0.521913	3.714196
67	1	0	-1.850990	-1.855807	4.350996
68	1	0	-4.578587	-2.340924	1.363196
69	1	0	0.687278	3.510808	-3.236904
70	1	0	0.952586	2.243209	-4.448104
71	1	0	1.856086	2.225815	-2.929704
72	6	0	-1.757127	4.468193	3.229196
73	1	0	-2.011029	4.775792	4.239996
74	6	0	-1.120720	3.245297	3.010296
75	1	0	-0.868616	2.602398	3.848896
76	6	0	-0.801018	2.846299	1.713896
77	1	0	-0.284012	1.902602	1.578796
78	6	0	-1.106022	3.665897	0.617696
79	6	0	-1.735130	4.899293	0.852796
80	1	0	-1.961334	5.531292	0.001096

81	6	0	-2.063032	5.295091	2.145696
82	1	0	-2.557538	6.248488	2.309896
83	8	0	-0.871126	4.196498	-1.664004
84	6	0	4.974204	-0.797867	-2.157604
85	1	0	5.578505	-1.017063	-3.034604
86	1	0	5.012698	0.225734	-1.797704
87	6	0	5.567501	-0.289163	2.406796
88	6	0	6.792796	0.583644	2.548396
89	1	0	7.389098	0.291848	3.424896
90	1	0	6.513290	1.635742	2.700696
91	1	0	7.438296	0.528748	1.666096
92	6	0	4.642201	-0.254369	3.599296
93	1	0	5.167403	-0.586966	4.505796
94	1	0	3.754805	-0.879674	3.479496
95	1	0	4.306395	0.773129	3.796996

Conformer 11b-1					
Center	Atomic	Atomic	Coordinates (Angstroms)		
Number	Number	Type	X	Y	Z
1	6	0	0.215099	0.385795	-1.922395
2	6	0	0.379101	1.769895	-2.639395
3	6	0	0.262297	-0.879805	-2.916895
4	6	0	1.369698	0.179594	-0.941095
5	6	0	-1.159701	0.357297	-1.233195
6	6	0	-1.396302	-0.478203	0.044705
7	6	0	-0.074603	-0.842604	0.770405
8	6	0	1.235497	-1.038806	-0.029295
9	6	0	0.033195	-2.183705	-2.071695
10	8	0	-2.071800	0.982398	-1.743395
11	6	0	1.042195	-2.316006	-0.915495
12	8	0	-0.076203	-1.027204	1.972005
13	8	0	2.322300	0.935692	-0.912995
14	6	0	2.413797	-1.167408	0.965605
15	6	0	-1.424405	-2.507103	-1.649195
16	6	0	-0.781503	-0.767203	-4.047295
17	6	0	-2.031104	-1.888302	-0.354895
18	6	0	-3.598805	-2.015399	-0.405995
19	8	0	-4.228404	-1.352199	0.718405
20	8	0	-3.423704	-1.546900	1.917105
21	6	0	-2.875902	-0.254600	2.240505

22	6	0	-2.258201	0.406099	1.001705
23	6	0	-3.985707	-3.503999	-0.324795
24	6	0	-4.307004	-1.337899	-1.588295
25	6	0	-3.942101	0.644301	2.937905
26	6	0	-4.615102	-0.137698	4.078205
27	6	0	-3.300599	1.925300	3.484405
28	8	0	-4.933500	1.083202	2.001705
29	6	0	1.656197	-0.982407	-3.587195
30	1	0	0.281094	-2.990305	-2.774695
31	1	0	0.752394	-3.146706	-0.259295
32	1	0	2.023795	-2.575607	-1.314395
33	6	0	3.847696	-1.381210	0.415705
34	1	0	3.995397	-0.715710	-0.439895
35	6	0	4.878497	-0.934211	1.502505
36	1	0	5.876997	-1.211413	1.134205
37	1	0	4.717896	-1.507811	2.422005
38	6	0	4.849499	0.546789	1.771905
39	1	0	4.937500	1.163689	0.876305
40	6	0	4.182694	-2.797910	-0.040795
41	6	0	3.929493	-3.944610	0.911105
42	1	0	4.423593	-3.782311	1.877405
43	1	0	4.299791	-4.888310	0.499305
44	1	0	2.860893	-4.070408	1.126705
45	1	0	2.162295	-1.962107	1.674505
46	1	0	2.429798	-0.246908	1.556805
47	1	0	-1.464107	-3.594202	-1.521295
48	1	0	-2.071605	-2.291702	-2.501095
49	1	0	-0.476702	-0.008804	-4.768195
50	1	0	-1.778702	-0.501002	-3.691895
51	1	0	-0.849204	-1.730103	-4.568995
52	1	0	-1.749805	-2.534502	0.480105
53	1	0	-2.104203	-0.517702	2.967705
54	1	0	-1.621100	1.227098	1.347805
55	1	0	-3.053801	0.862900	0.415705
56	1	0	-5.076107	-3.586097	-0.282095
57	1	0	-3.574607	-3.959500	0.582005
58	1	0	-3.635807	-4.071499	-1.192495
59	1	0	-3.996902	-0.298599	-1.704295
60	1	0	-5.387204	-1.366497	-1.409595
61	1	0	-4.120204	-1.872899	-2.523695

62	1	0	-5.106503	-1.036597	3.696005
63	1	0	-5.367501	0.497703	4.555605
64	1	0	-3.887402	-0.448299	4.837905
65	1	0	-4.059198	2.513601	4.010505
66	1	0	-2.904498	2.545500	2.675305
67	1	0	-2.491999	1.696299	4.187505
68	1	0	-5.221901	0.298503	1.505505
69	1	0	1.854998	-0.096807	-4.189895
70	1	0	1.666696	-1.859207	-4.245695
71	1	0	2.477197	-1.098008	-2.874595
72	6	0	-0.135894	5.537096	-0.610495
73	1	0	-0.266693	6.498796	-0.121595
74	6	0	-0.043496	4.372096	0.152805
75	1	0	-0.093596	4.423596	1.236805
76	6	0	0.117803	3.137795	-0.472995
77	1	0	0.208701	2.253695	0.147905
78	6	0	0.195902	3.047895	-1.869895
79	6	0	0.111104	4.228795	-2.625495
80	1	0	0.178004	4.153995	-3.705195
81	6	0	-0.057494	5.461696	-2.002995
82	1	0	-0.127993	6.364696	-2.603095
83	8	0	0.646701	1.822295	-3.826395
84	6	0	4.757594	-3.010811	-1.232095
85	1	0	5.056493	-4.005312	-1.555795
86	1	0	4.962095	-2.195811	-1.922995
87	6	0	4.736100	1.185289	2.947805
88	6	0	4.752002	2.695289	3.006105
89	1	0	5.589202	3.059488	3.618805
90	1	0	3.835702	3.082790	3.473705
91	1	0	4.840203	3.141589	2.010905
92	6	0	4.601599	0.513489	4.293205
93	1	0	4.558697	-0.576511	4.233605
94	1	0	3.693999	0.857790	4.808005
95	1	0	5.445999	0.779588	4.944505

Conformer 11b-3

Center	Atomic	Atomic	Coordinates (Angstroms)		
Number	Number	Type	X	Y	Z
1	6	0	-0.283299	0.991098	1.324500
2	6	0	-0.509597	2.533198	1.487500
3	6	0	-0.575801	0.137498	2.658200

4	6	0	-1.238400	0.440399	0.266600
5	6	0	1.194500	0.750196	0.965100
6	6	0	1.622698	-0.460405	0.104500
7	6	0	0.441697	-1.068403	-0.694600
8	6	0	-0.995202	-1.015001	-0.128000
9	6	0	-0.250003	-1.371102	2.366300
10	8	0	2.027101	1.517895	1.411600
11	6	0	-1.030104	-1.912501	1.153500
12	8	0	0.647397	-1.629403	-1.753300
13	8	0	-2.125199	1.121201	-0.214800
14	6	0	-1.990703	-1.495599	-1.206300
15	6	0	1.247096	-1.782404	2.358900
16	6	0	0.238500	0.651597	3.864200
17	6	0	2.101897	-1.642406	1.063600
18	6	0	3.628096	-1.729508	1.441400
19	8	0	4.480097	-1.462209	0.301600
20	8	0	3.899396	-2.057508	-0.894500
21	6	0	3.485698	-0.954908	-1.723300
22	6	0	2.683899	0.070994	-0.911800
23	6	0	3.948194	-3.158308	1.918400
24	6	0	4.146398	-0.695109	2.451600
25	6	0	4.704699	-0.322710	-2.461200
26	6	0	5.540797	-1.429311	-3.125500
27	6	0	4.239500	0.689591	-3.514900
28	8	0	5.523500	0.422089	-1.551700
29	6	0	-2.074401	0.240201	3.040300
30	1	0	-0.656404	-1.895201	3.241600
31	1	0	-0.663605	-2.910801	0.883100
32	1	0	-2.079504	-2.047099	1.420000
33	6	0	-3.494403	-1.555097	-0.798800
34	1	0	-3.656802	-0.915697	0.074500
35	6	0	-4.356702	-0.929196	-1.938300
36	1	0	-4.196203	-1.504696	-2.861700
37	1	0	-3.952901	0.069504	-2.125800
38	6	0	-5.830802	-0.866594	-1.639600
39	1	0	-6.369904	-1.808493	-1.746500
40	6	0	-3.974205	-2.954796	-0.426400
41	6	0	-3.873107	-4.050196	-1.463900
42	1	0	-4.453307	-3.803896	-2.362800
43	1	0	-4.251809	-4.998996	-1.072200

44	1	0	-2.840907	-4.214298	-1.797900
45	1	0	-1.643905	-2.471200	-1.555400
46	1	0	-1.881102	-0.827000	-2.065900
47	1	0	1.268395	-2.842904	2.632100
48	1	0	1.737097	-1.259805	3.181800
49	1	0	-0.155298	1.608798	4.205800
50	1	0	1.298800	0.791896	3.645200
51	1	0	0.151499	-0.069103	4.687000
52	1	0	1.935495	-2.539505	0.462200
53	1	0	2.848397	-1.456707	-2.455000
54	1	0	2.157300	0.716794	-1.622700
55	1	0	3.380100	0.712592	-0.374000
56	1	0	5.023394	-3.237510	2.106200
57	1	0	3.682993	-3.890208	1.148700
58	1	0	3.422794	-3.415108	2.843400
59	1	0	3.857400	0.320192	2.176700
60	1	0	5.239598	-0.755110	2.480500
61	1	0	3.785198	-0.910108	3.461300
62	1	0	5.904596	-2.142511	-2.380900
63	1	0	6.401298	-0.977312	-3.628700
64	1	0	4.955696	-1.986110	-3.867700
65	1	0	5.111701	1.083990	-4.046100
66	1	0	3.724401	1.536392	-3.052400
67	1	0	3.571799	0.222692	-4.247700
68	1	0	5.677399	-0.146511	-0.778400
69	1	0	-2.338599	1.271201	3.273200
70	1	0	-2.255301	-0.374399	3.930300
71	1	0	-2.750201	-0.109598	2.255400
72	6	0	0.636307	5.395197	-1.523000
73	1	0	0.924208	6.135496	-2.264500
74	6	0	0.597205	4.042497	-1.864900
75	1	0	0.845705	3.726696	-2.874100
76	6	0	0.235504	3.091397	-0.912900
77	1	0	0.192802	2.050897	-1.214500
78	6	0	-0.099696	3.477198	0.392700
79	6	0	-0.067494	4.842598	0.720300
80	1	0	-0.334493	5.135698	1.729700
81	6	0	0.301808	5.792997	-0.226400
82	1	0	0.329410	6.844397	0.045900
83	8	0	-1.007996	2.979499	2.505300

84	6	0	-4.524806	-3.197696	0.769500
85	1	0	-4.898407	-4.182695	1.040200
86	1	0	-4.639405	-2.416095	1.517200
87	6	0	-6.538701	0.204208	-1.246200
88	6	0	-8.025001	0.097210	-0.997800
89	1	0	-8.584100	0.792111	-1.640600
90	1	0	-8.274300	0.366710	0.038300
91	1	0	-8.399802	-0.914090	-1.185300
92	6	0	-5.963698	1.582007	-1.017900
93	1	0	-6.390697	2.305307	-1.727600
94	1	0	-4.875698	1.622305	-1.103000
95	1	0	-6.229398	1.943607	-0.014900

Conformer 11b-11

Center	Atomic	Atomic	Coordinates (Angstroms)		
Number	Number	Type	X	Y	Z
1	6	0	0.242100	0.385699	-1.874096
2	6	0	0.455701	1.768798	-2.579096
3	6	0	0.280699	-0.876501	-2.874096
4	6	0	1.371900	0.142498	-0.872996
5	6	0	-1.149300	0.389800	-1.218996
6	6	0	-1.440101	-0.446800	0.046304
7	6	0	-0.147401	-0.840501	0.806904
8	6	0	1.179699	-1.064802	0.045404
9	6	0	0.016798	-2.178401	-2.035496
10	8	0	-2.030499	1.044401	-1.746496
11	6	0	1.000798	-2.330102	-0.860296
12	8	0	-0.187201	-1.017701	2.009004
13	8	0	2.362000	0.848597	-0.849196
14	6	0	2.329799	-1.205403	1.070804
15	6	0	-1.453103	-2.482800	-1.643496
16	6	0	-0.744701	-0.740601	-4.018696
17	6	0	-2.089602	-1.843899	-0.373196
18	6	0	-3.657102	-1.937598	-0.476796
19	8	0	-4.308601	-1.261897	0.627104
20	8	0	-3.549602	-1.479198	1.851404
21	6	0	-2.982100	-0.201399	2.198004
22	6	0	-2.311300	0.450901	0.982504
23	6	0	-4.078003	-3.417698	-0.411196
24	6	0	-4.311101	-1.243997	-1.680796
25	6	0	-4.048800	0.718502	2.866604

26	6	0	-4.775900	-0.053197	3.980604
27	6	0	-3.395399	1.981302	3.440604
28	8	0	-4.999399	1.184703	1.901804
29	6	0	1.680599	-1.000303	-3.528296
30	1	0	0.265597	-2.988201	-2.734696
31	1	0	0.710397	-3.174802	-0.224896
32	1	0	1.988897	-2.582203	-1.248596
33	6	0	3.728098	-1.597805	0.542904
34	1	0	3.895299	-1.087205	-0.413396
35	6	0	4.828199	-1.069506	1.518304
36	1	0	5.797598	-1.458006	1.171004
37	1	0	4.660598	-1.518505	2.503304
38	6	0	4.899600	0.432694	1.590704
39	1	0	4.922501	0.927794	0.619204
40	6	0	3.965797	-3.092805	0.320904
41	6	0	4.875097	-3.429506	-0.838296
42	1	0	4.431097	-3.118005	-1.794696
43	1	0	5.086896	-4.501706	-0.894896
44	1	0	5.832897	-2.897806	-0.758796
45	1	0	2.004698	-1.910303	1.839104
46	1	0	2.422299	-0.239803	1.577404
47	1	0	-1.505603	-3.568000	-1.504496
48	1	0	-2.075702	-2.271899	-2.514696
49	1	0	-0.422100	0.023899	-4.725396
50	1	0	-1.745001	-0.469900	-3.675596
51	1	0	-0.812802	-1.696600	-4.552896
52	1	0	-1.851203	-2.492600	0.473004
53	1	0	-2.239601	-0.485399	2.947304
54	1	0	-1.666199	1.254800	1.352904
55	1	0	-3.077399	0.929502	0.375104
56	1	0	-5.170803	-3.476497	-0.405496
57	1	0	-3.707704	-3.882998	0.508004
58	1	0	-3.711304	-3.991598	-1.267696
59	1	0	-3.978300	-0.210298	-1.783296
60	1	0	-5.397101	-1.252796	-1.539496
61	1	0	-4.102402	-1.780498	-2.610796
62	1	0	-5.277901	-0.936896	3.577604
63	1	0	-5.526500	0.598104	4.439004
64	1	0	-4.080201	-0.387098	4.760104
65	1	0	-4.156998	2.585803	3.943404

66	1	0	-2.956698	2.594701	2.648404
67	1	0	-2.617399	1.729401	4.169804
68	1	0	-5.288400	0.409604	1.391204
69	1	0	1.896400	-0.121303	-4.134696
70	1	0	1.688198	-1.881803	-4.180596
71	1	0	2.492899	-1.116003	-2.805896
72	6	0	0.035605	5.528699	-0.515496
73	1	0	-0.070894	6.488799	-0.017796
74	6	0	0.077104	4.352999	0.235804
75	1	0	0.011404	4.394199	1.319304
76	6	0	0.207502	3.120999	-0.401696
77	1	0	0.258202	2.227799	0.211004
78	6	0	0.304702	3.043999	-1.798196
79	6	0	0.270803	4.235199	-2.541496
80	1	0	0.351503	4.169798	-3.620896
81	6	0	0.133505	5.466099	-1.907496
82	1	0	0.102205	6.377399	-2.498196
83	8	0	0.734701	1.823398	-3.763296
84	6	0	3.482096	-4.052504	1.117704
85	1	0	3.726295	-5.099005	0.952204
86	1	0	2.836896	-3.843104	1.966204
87	6	0	4.938101	1.216194	2.680504
88	6	0	5.028302	2.718394	2.544304
89	1	0	5.942002	3.105293	3.017904
90	1	0	4.186003	3.212595	3.049104
91	1	0	5.027502	3.034494	1.496704
92	6	0	4.916000	0.721494	4.106804
93	1	0	5.834901	1.017594	4.632404
94	1	0	4.822199	-0.363706	4.190104
95	1	0	4.083501	1.175695	4.661904

Conformer 12a-2

Center	Atomic	Atomic	Coordinates (Angstroms)		
Number	Number	Type	X	Y	Z
1	6	0	-3.962364	0.876399	0.159108
2	6	0	-2.953802	0.632818	-2.137248
3	1	0	-2.392522	-1.139610	0.810292
4	6	0	4.415062	-1.545004	0.465042
5	6	0	5.236073	-2.641916	0.718832
6	6	0	6.279087	-2.956550	-0.157003

7	6	0	6.512216	-2.155629	-1.277320
8	6	0	5.708918	-1.041418	-1.517096
9	8	0	4.556966	1.534169	-1.216905
10	6	0	3.869116	0.517711	-0.904331
11	6	0	2.435178	0.622284	-0.738331
12	6	0	4.641087	-0.738756	-0.659804
13	6	0	1.847249	1.917055	-0.789023
14	6	0	0.438385	2.172472	-0.647638
15	6	0	-0.383900	1.111709	-0.389227
16	6	0	0.118942	-0.286188	-0.134123
17	6	0	1.531230	-0.526198	-0.699934
18	8	0	2.599304	2.974572	-1.021005
19	6	0	-0.080890	3.592284	-0.794483
20	6	0	0.072501	4.403379	0.473691
21	6	0	-0.887304	4.995932	1.201257
22	6	0	-0.534188	5.783202	2.441708
23	6	0	-2.362442	4.960729	0.885094
24	8	0	-1.691289	1.349990	-0.207094
25	6	0	-2.752175	0.394340	-0.637644
26	6	0	-2.328683	-1.051876	-0.281935
27	6	0	-0.873008	-1.316596	-0.699477
28	6	0	-3.226712	-2.165340	-0.884369
29	6	0	-4.630937	-2.251144	-0.346509
30	6	0	-5.778737	-2.411136	-1.026241
31	6	0	-7.095046	-2.535064	-0.295298
32	6	0	-5.891460	-2.500281	-2.528694
33	6	0	0.249881	-0.406021	1.448502
34	6	0	0.543977	-1.798576	1.933014
35	6	0	-0.056524	-2.461956	2.936164
36	6	0	0.394495	-3.853762	3.310672
37	6	0	-1.180195	-1.923408	3.786595
38	8	0	1.848275	-1.660652	-1.058489
39	1	0	-3.787761	0.760544	1.232629
40	1	0	-4.151179	1.933136	-0.050375
41	1	0	-4.848073	0.300431	-0.112978
42	1	0	-3.834534	0.094050	-2.495079
43	1	0	-3.111518	1.698763	-2.320515
44	1	0	-2.090274	0.307692	-2.724187
45	1	0	3.612601	-1.302170	1.152743
46	1	0	5.062786	-3.251546	1.600448

47	1	0	6.910723	-3.818304	0.037081
48	1	0	7.324252	-2.392614	-1.958067
49	1	0	5.897382	-0.401515	-2.372799
50	1	0	3.550658	2.599923	-1.169823
51	1	0	0.486296	4.080409	-1.595816
52	1	0	-1.122346	3.549530	-1.113051
53	1	0	1.101988	4.508023	0.815547
54	1	0	-1.034720	5.368702	3.326826
55	1	0	0.542924	5.789236	2.630210
56	1	0	-0.871913	6.824617	2.356802
57	1	0	-2.607225	4.329351	0.029887
58	1	0	-2.929650	4.591050	1.749001
59	1	0	-2.738196	5.971979	0.680836
60	1	0	-0.787920	-1.327207	-1.792185
61	1	0	-0.574657	-2.310523	-0.361406
62	1	0	-2.717508	-3.111290	-0.646878
63	1	0	-3.217186	-2.095453	-1.975759
64	1	0	-4.698468	-2.219646	0.742181
65	1	0	-6.969327	-2.472218	0.789098
66	1	0	-7.793463	-1.746858	-0.605839
67	1	0	-7.585111	-3.489833	-0.527458
68	1	0	-4.940537	-2.364004	-3.046738
69	1	0	-6.295767	-3.477130	-2.824790
70	1	0	-6.595717	-1.748546	-2.907217
71	1	0	1.050612	0.280690	1.751906
72	1	0	-0.669785	-0.014889	1.887796
73	1	0	1.349720	-2.310089	1.409148
74	1	0	1.210153	-4.206095	2.673693
75	1	0	0.734418	-3.888721	4.353954
76	1	0	-0.435638	-4.567783	3.232533
77	1	0	-1.519555	-0.930627	3.486688
78	1	0	-2.042176	-2.601771	3.758574
79	1	0	-0.868022	-1.865284	4.837327

Conformer 12a-3

Center	Atomic	Atomic	Coordinates (Angstroms)		
Number	Number	Type	X	Y	Z
1	6	0	-4.194682	0.787000	0.539415
2	6	0	-3.430285	0.089485	-1.757975
3	1	0	-2.664450	-1.160844	1.399715
4	6	0	4.229631	-1.706411	0.458606

5	6	0	5.122984	-2.656016	0.950161
6	6	0	6.016901	-3.295280	0.085862
7	6	0	6.029628	-2.965333	-1.271320
8	6	0	5.157240	-1.993390	-1.759635
9	8	0	3.994158	0.534103	-2.237679
10	6	0	3.401533	-0.259637	-1.448586
11	6	0	2.019878	-0.024416	-1.074076
12	6	0	4.237664	-1.370876	-0.903062
13	6	0	1.431938	1.213525	-1.453612
14	6	0	0.081795	1.610465	-1.133995
15	6	0	-0.692500	0.735055	-0.431362
16	6	0	-0.192545	-0.569559	0.124005
17	6	0	1.165448	-1.025826	-0.444716
18	8	0	2.114889	2.075333	-2.177859
19	6	0	-0.424770	2.961289	-1.608716
20	6	0	0.082894	4.103702	-0.754962
21	6	0	-0.627998	4.925403	0.033675
22	6	0	0.055912	6.029477	0.805837
23	6	0	-2.122826	4.859996	0.230614
24	8	0	-1.955469	1.083185	-0.118924
25	6	0	-3.084641	0.117574	-0.266231
26	6	0	-2.672502	-1.264168	0.307746
27	6	0	-1.257735	-1.656218	-0.150858
28	6	0	-3.639321	-2.431179	-0.029975
29	6	0	-5.007217	-2.359810	0.596011
30	6	0	-6.201261	-2.592149	0.025306
31	6	0	-7.470343	-2.527070	0.842212
32	6	0	-6.413724	-2.945562	-1.426750
33	6	0	0.009584	-0.411113	1.685018
34	6	0	1.082237	0.552950	2.116130
35	6	0	2.004288	0.375150	3.077877
36	6	0	2.976127	1.479383	3.421238
37	6	0	2.160699	-0.873065	3.912027
38	8	0	1.468797	-2.213902	-0.323679
39	1	0	-3.913123	0.865149	1.593375
40	1	0	-4.382622	1.793235	0.154020
41	1	0	-5.115529	0.206283	0.467374
42	1	0	-2.636188	-0.367131	-2.355785
43	1	0	-3.585355	1.109866	-2.117684
44	1	0	-4.352288	-0.472255	-1.925274

45	1	0	3.536392	-1.211782	1.129696
46	1	0	5.121376	-2.899695	2.008329
47	1	0	6.703422	-4.043745	0.470406
48	1	0	6.724188	-3.456458	-1.946116
49	1	0	5.177380	-1.713508	-2.807678
50	1	0	3.016055	1.606096	-2.375423
51	1	0	-0.082483	3.114026	-2.638958
52	1	0	-1.513756	2.935939	-1.626934
53	1	0	1.160315	4.257073	-0.804469
54	1	0	-0.111658	5.917371	1.885146
55	1	0	1.134628	6.046534	0.627594
56	1	0	-0.351345	7.011686	0.531610
57	1	0	-2.586954	4.002955	-0.259216
58	1	0	-2.363610	4.804840	1.299983
59	1	0	-2.603292	5.772853	-0.145518
60	1	0	-1.263552	-1.876790	-1.225189
61	1	0	-0.949000	-2.578047	0.346984
62	1	0	-3.143732	-3.339007	0.345206
63	1	0	-3.698850	-2.558741	-1.114497
64	1	0	-5.002462	-2.131075	1.662986
65	1	0	-7.272603	-2.269183	1.886233
66	1	0	-8.165357	-1.783369	0.430794
67	1	0	-8.000297	-3.488475	0.822057
68	1	0	-7.116781	-2.244832	-1.894800
69	1	0	-5.494254	-2.945645	-2.014756
70	1	0	-6.867019	-3.941459	-1.515910
71	1	0	-0.950235	-0.083110	2.100517
72	1	0	0.199613	-1.416818	2.067983
73	1	0	1.062928	1.521305	1.617101
74	1	0	2.824197	2.366487	2.800507
75	1	0	2.877155	1.776651	4.473467
76	1	0	4.012997	1.143119	3.291313
77	1	0	1.489714	-1.682274	3.619458
78	1	0	3.190416	-1.248168	3.855857
79	1	0	1.972840	-0.649063	4.970178

Conformer 12a-6

Center	Atomic	Atomic	Coordinates (Angstroms)		
Number	Number	Type	X	Y	Z
1	6	0	-4.194691	0.787015	0.539325
2	6	0	-3.430164	0.089582	-1.758037

3	1	0	-2.664437	-1.160882	1.399620
4	6	0	5.157360	-1.993356	-1.759555
5	6	0	6.029723	-2.965254	-1.271110
6	6	0	6.016838	-3.295169	0.086077
7	6	0	5.122794	-2.655918	0.950257
8	6	0	4.229475	-1.706351	0.458574
9	8	0	3.994115	0.534033	-2.237887
10	6	0	3.401551	-0.259644	-1.448719
11	6	0	2.019887	-0.024402	-1.074151
12	6	0	4.237664	-1.370848	-0.903113
13	6	0	1.431946	1.213517	-1.453667
14	6	0	0.081797	1.610461	-1.134002
15	6	0	-0.692471	0.735043	-0.431360
16	6	0	-0.192522	-0.569612	0.123918
17	6	0	1.165442	-1.025879	-0.444920
18	8	0	2.114842	2.075313	-2.177998
19	6	0	-0.424773	2.961302	-1.608665
20	6	0	0.082978	4.103665	-0.754880
21	6	0	-0.627817	4.925424	0.033782
22	6	0	0.056209	6.029418	0.805949
23	6	0	-2.122637	4.860107	0.230788
24	8	0	-1.955419	1.083185	-0.118807
25	6	0	-3.084613	0.117598	-0.266268
26	6	0	-2.672501	-1.264165	0.307645
27	6	0	-1.257752	-1.656229	-0.150997
28	6	0	-3.639380	-2.431117	-0.030104
29	6	0	-5.007241	-2.359659	0.595957
30	6	0	-6.201326	-2.591969	0.025329
31	6	0	-7.470364	-2.526777	0.842296
32	6	0	-6.413864	-2.945510	-1.426683
33	6	0	0.009685	-0.411323	1.684904
34	6	0	1.082327	0.552729	2.116095
35	6	0	2.004183	0.374960	3.078033
36	6	0	2.976004	1.479177	3.421505
37	6	0	2.160413	-0.873234	3.912245
38	8	0	1.468727	-2.213990	-0.324120
39	1	0	-3.913233	0.865089	1.593318
40	1	0	-4.382537	1.793280	0.153965
41	1	0	-5.115560	0.206343	0.467179
42	1	0	-2.635994	-0.366938	-2.355822

43	1	0	-3.585292	1.109974	-2.117686
44	1	0	-4.352116	-0.472216	-1.925434
45	1	0	5.177599	-1.713504	-2.807605
46	1	0	6.724389	-3.456374	-1.945801
47	1	0	6.703339	-4.043604	0.470718
48	1	0	5.121071	-2.899585	2.008427
49	1	0	3.536158	-1.211705	1.129569
50	1	0	3.015954	1.606037	-2.375648
51	1	0	-0.082547	3.114077	-2.638920
52	1	0	-1.513762	2.935965	-1.626800
53	1	0	1.160415	4.256923	-0.804395
54	1	0	-0.111513	5.917429	1.885247
55	1	0	1.134950	6.046252	0.627836
56	1	0	-0.350826	7.011685	0.531596
57	1	0	-2.586933	4.003507	-0.259650
58	1	0	-2.363352	4.804193	1.300138
59	1	0	-2.602975	5.773320	-0.144628
60	1	0	-1.263568	-1.876742	-1.225338
61	1	0	-0.949054	-2.578097	0.346798
62	1	0	-3.143829	-3.338995	0.345004
63	1	0	-3.698981	-2.558625	-1.114629
64	1	0	-5.002416	-2.130843	1.662914
65	1	0	-8.165336	-1.783024	0.430903
66	1	0	-8.000403	-3.488139	0.822176
67	1	0	-7.272553	-2.268899	1.886305
68	1	0	-7.117279	-2.245091	-1.894652
69	1	0	-5.494480	-2.945238	-2.014827
70	1	0	-6.866753	-3.941603	-1.515743
71	1	0	-0.950123	-0.083412	2.100512
72	1	0	0.199782	-1.417059	2.067762
73	1	0	1.063165	1.521044	1.616987
74	1	0	2.824296	2.366211	2.800621
75	1	0	2.876771	1.776586	4.473671
76	1	0	4.012892	1.142837	3.291904
77	1	0	1.489191	-1.682299	3.619824
78	1	0	3.190037	-1.248582	3.855954
79	1	0	1.972767	-0.649114	4.970408

Conformer 13a-1

Center	Atomic	Atomic	Coordinates (Angstroms)		

Number	Number	Type	X	Y	Z
1	6	0	4.419589	-1.216650	-0.483701
2	6	0	3.197475	-2.337735	1.402099
3	6	0	-4.879765	-5.582737	-0.152501
4	6	0	-4.271357	-4.933444	2.223099
5	6	0	-3.937355	-4.827148	0.754799
6	6	0	-2.904347	-4.138261	0.246399
7	6	0	-3.576707	-0.881952	-0.778601
8	6	0	-3.514005	-0.669453	1.693099
9	6	0	-1.854337	-3.341573	0.970499
10	6	0	-2.937010	-1.120960	0.371199
11	6	0	-1.632220	-1.911176	0.382399
12	6	0	-0.469312	-1.252590	1.163299
13	6	0	-3.670647	4.008649	-0.702601
14	6	0	-4.094634	5.119554	0.023099
15	6	0	-3.186225	5.820443	0.818899
16	6	0	-1.856230	5.399627	0.891399
17	6	0	-1.439244	4.269521	0.192099
18	8	0	-2.453267	2.427834	-2.645201
19	6	0	-1.930767	2.432427	-1.497901
20	6	0	-0.959679	1.404016	-1.135201
21	6	0	-2.344553	3.560433	-0.609101
22	6	0	-0.457789	0.574709	-2.170101
23	6	0	0.602899	-0.395403	-1.997801
24	6	0	1.046296	-0.635809	-0.735401
25	6	0	0.376102	-0.096401	0.497999
26	6	0	-0.583683	1.092911	0.237999
27	8	0	-0.897988	0.692615	-3.407301
28	6	0	1.210891	-1.051711	-3.225601
29	6	0	2.194402	-0.153623	-3.943901
30	6	0	3.408998	-0.468238	-4.420301
31	6	0	4.049781	-1.832146	-4.329801
32	6	0	4.241110	0.565052	-5.144401
33	8	0	2.065385	-1.505621	-0.555601
34	6	0	3.162789	-1.186235	0.392499
35	6	0	2.893906	0.223869	0.974699
36	6	0	1.469208	0.335186	1.532899
37	6	0	3.949513	0.763156	1.986899
38	6	0	3.919106	0.209256	3.390299
39	6	0	3.498714	0.819761	4.510899

40	6	0	2.927531	2.215168	4.582699
41	6	0	3.577805	0.114160	5.844799
42	8	0	-1.016876	1.675816	1.231899
43	1	0	4.513877	-2.194951	-0.965101
44	1	0	5.321191	-1.043561	0.112799
45	1	0	4.361598	-0.455149	-1.267501
46	1	0	2.379276	-2.290325	2.124799
47	1	0	3.122663	-3.285934	0.860199
48	1	0	4.141175	-2.340147	1.954999
49	1	0	-5.910960	-5.218324	-0.041801
50	1	0	-4.598563	-5.485640	-1.205801
51	1	0	-4.900678	-6.653236	0.097499
52	1	0	-4.239970	-5.981644	2.552599
53	1	0	-5.294352	-4.580331	2.413699
54	1	0	-3.595350	-4.364252	2.865499
55	1	0	-2.784047	-4.151462	-0.838501
56	1	0	-4.531101	-0.361541	-0.804401
57	1	0	-3.169611	-1.188157	-1.739301
58	1	0	-4.525500	-0.273941	1.559799
59	1	0	-3.569315	-1.493253	2.416699
60	1	0	-2.904495	0.123739	2.141999
61	1	0	-0.891344	-3.870785	0.896899
62	1	0	-2.074836	-3.266171	2.041099
63	1	0	-1.321121	-2.048780	-0.661201
64	1	0	-0.838907	-0.837786	2.106799
65	1	0	0.247979	-2.038899	1.420599
66	1	0	-4.359154	3.475257	-1.349601
67	1	0	-5.129530	5.444967	-0.041101
68	1	0	-3.511715	6.694347	1.377399
69	1	0	-1.143224	5.949618	1.499699
70	1	0	-0.407948	3.944209	0.259799
71	1	0	-1.650679	1.393224	-3.363401
72	1	0	1.679079	-1.989317	-2.923201
73	1	0	0.392788	-1.302201	-3.913301
74	1	0	1.836514	0.862581	-4.108601
75	1	0	4.194776	-2.256047	-5.333601
76	1	0	5.048382	-1.765358	-3.876201
77	1	0	3.464172	-2.548838	-3.749401
78	1	0	4.460307	0.246649	-6.173601
79	1	0	3.737122	1.535458	-5.191601

80	1	0	5.213612	0.709240	-4.652101
81	1	0	2.960815	0.897168	0.106299
82	1	0	1.382700	-0.268913	2.440899
83	1	0	1.269720	1.363488	1.845499
84	1	0	4.951711	0.606543	1.565799
85	1	0	3.812826	1.849057	2.016999
86	1	0	4.310794	-0.800649	3.504799
87	1	0	2.903537	2.728468	3.618999
88	1	0	1.900130	2.190081	4.970399
89	1	0	3.507938	2.833761	5.280999
90	1	0	2.585404	0.035772	6.310199
91	1	0	4.206912	0.674552	6.550599
92	1	0	3.989893	-0.895645	5.751599

Conformer 13a-2

Center	Atomic	Atomic	Coordinates (Angstroms)		
Number	Number	Type	X	Y	Z
1	6	0	3.432694	-1.025515	-0.276595
2	6	0	1.958684	-2.960907	0.366005
3	6	0	4.264022	4.475981	-1.718595
4	6	0	5.243127	5.473376	0.399005
5	6	0	4.050524	4.825882	-0.265595
6	6	0	2.917123	4.608988	0.421905
7	6	0	0.458415	3.113100	2.802405
8	6	0	2.656209	1.970789	2.633805
9	6	0	1.633520	3.994294	-0.067895
10	6	0	1.406912	2.567495	2.032805
11	6	0	1.331812	2.570296	0.501205
12	6	0	-0.024290	2.087803	-0.073895
13	6	0	-5.890692	1.662632	-1.008595
14	6	0	-6.707391	1.826937	-2.124595
15	6	0	-6.534796	1.005536	-3.240895
16	6	0	-5.538401	0.027331	-3.236695
17	6	0	-4.697501	-0.114874	-2.134595
18	8	0	-4.792298	0.582527	1.314705
19	6	0	-4.099998	0.518323	0.261505
20	6	0	-2.662999	0.277916	0.353905
21	6	0	-4.862797	0.706527	-1.011095
22	6	0	-2.137801	-0.106187	1.618505
23	6	0	-0.766803	-0.521794	1.835305
24	6	0	0.122598	-0.286898	0.835905

25	6	0	-0.209598	0.545404	-0.371495
26	6	0	-1.719198	0.462511	-0.749795
27	8	0	-2.916702	-0.201683	2.675805
28	6	0	-0.402107	-1.208295	3.139005
29	6	0	-0.924714	-2.625293	3.232005
30	6	0	-0.273220	-3.722796	3.646705
31	6	0	-0.976326	-5.059792	3.695905
32	6	0	1.159580	-3.756403	4.120505
33	8	0	1.404496	-0.706805	0.946105
34	6	0	1.992792	-1.522108	-0.154995
35	6	0	1.149293	-1.332403	-1.446495
36	6	0	0.681000	0.126299	-1.558695
37	6	0	1.853290	-1.758407	-2.760895
38	6	0	2.144783	-3.229008	-2.908595
39	6	0	3.274180	-3.825714	-3.323895
40	6	0	3.346572	-5.329214	-3.456695
41	6	0	4.543784	-3.106420	-3.711695
42	8	0	-2.032997	0.679013	-1.916295
43	1	0	4.013091	-1.694018	-0.918895
44	1	0	3.485599	-0.012615	-0.687295
45	1	0	3.899094	-1.015517	0.713305
46	1	0	2.393681	-3.643810	-0.368995
47	1	0	2.530284	-3.035610	1.296605
48	1	0	0.930183	-3.274002	0.572905
49	1	0	5.116219	3.790677	-1.829295
50	1	0	3.395320	4.005785	-2.185195
51	1	0	4.513727	5.373580	-2.301695
52	1	0	5.048128	5.706977	1.450405
53	1	0	5.521232	6.406775	-0.110795
54	1	0	6.126724	4.820972	0.352705
55	1	0	2.902924	4.910488	1.469405
56	1	0	-0.445783	3.559105	2.395505
57	1	0	0.551915	3.134500	3.885105
58	1	0	2.672710	2.078589	3.723205
59	1	0	3.553212	2.455585	2.226305
60	1	0	2.729204	0.905089	2.387205
61	1	0	0.797023	4.650799	0.213405
62	1	0	1.621319	3.930094	-1.161595
63	1	0	2.130109	1.915592	0.133205
64	1	0	-0.831088	2.414607	0.587805

65	1	0	-0.204188	2.572104	-1.041695
66	1	0	-6.038289	2.271833	-0.122795
67	1	0	-7.484788	2.586141	-2.119095
68	1	0	-7.177995	1.123639	-4.109095
69	1	0	-5.410104	-0.623370	-4.097395
70	1	0	-3.920105	-0.868378	-2.144295
71	1	0	-3.842100	0.128922	2.363305
72	1	0	-0.832004	-0.615793	3.958105
73	1	0	0.681893	-1.176501	3.253105
74	1	0	-1.970615	-2.738987	2.948705
75	1	0	-2.012826	-4.989487	3.351405
76	1	0	-0.985228	-5.465492	4.717605
77	1	0	-0.459430	-5.803395	3.072605
78	1	0	1.669385	-2.794006	4.033805
79	1	0	1.739477	-4.499306	3.555405
80	1	0	1.208879	-4.064204	5.174605
81	1	0	0.251589	-1.960699	-1.338195
82	1	0	0.099101	0.271002	-2.473095
83	1	0	1.555103	0.783795	-1.630695
84	1	0	2.753794	-1.153311	-2.906795
85	1	0	1.167692	-1.466703	-3.571195
86	1	0	1.295580	-3.877404	-2.683495
87	1	0	2.406670	-5.811910	-3.170995
88	1	0	4.147970	-5.744418	-2.829495
89	1	0	3.578671	-5.623616	-4.489895
90	1	0	4.490589	-2.023420	-3.577695
91	1	0	5.396082	-3.479325	-3.126995
92	1	0	4.786683	-3.298122	-4.766195

Conformer 13b-1					
Center	Atomic	Atomic	Coordinates (Angstroms)		
Number	Number	Type	X	Y	Z
1	6	0	3.166514	2.397780	1.286700
2	6	0	4.656003	0.850569	-0.010800
3	6	0	-5.537077	3.734042	0.118600
4	6	0	-3.947385	2.570731	-1.486100
5	6	0	-4.176981	3.110732	-0.094200
6	6	0	-3.281182	3.073326	0.905400
7	6	0	-3.135699	0.580825	3.639000

8	6	0	-1.512986	2.424813	3.986200
9	6	0	-1.882286	2.514416	0.864500
10	6	0	-2.176694	1.365218	3.135600
11	6	0	-1.718195	1.206215	1.686200
12	6	0	-0.285899	0.602805	1.693600
13	6	0	-3.587527	-3.247772	-2.238100
14	6	0	-4.841724	-2.929863	-2.755600
15	6	0	-5.620017	-1.944457	-2.145200
16	6	0	-5.134613	-1.274261	-1.019800
17	6	0	-3.866515	-1.568170	-0.523700
18	8	0	-1.650034	-4.264886	-0.457100
19	6	0	-1.777825	-3.014785	-0.554300
20	6	0	-0.717119	-2.126292	-0.091700
21	6	0	-3.079522	-2.553476	-1.131200
22	6	0	0.414577	-2.714000	0.537400
23	6	0	1.589082	-1.983509	0.947400
24	6	0	1.610092	-0.634009	0.756900
25	6	0	0.395898	0.164300	0.327700
26	6	0	-0.645109	-0.707293	-0.432100
27	8	0	0.476268	-4.014601	0.735400
28	6	0	2.787777	-2.751717	1.481400
29	6	0	3.673273	-3.283924	0.376800
30	6	0	3.966064	-4.558926	0.079900
31	6	0	4.891062	-4.884632	-1.069800
32	6	0	3.439855	-5.764822	0.818200
33	8	0	2.727797	0.033883	1.105700
34	6	0	3.204905	1.203680	0.329300
35	6	0	2.317506	1.312186	-0.930700
36	6	0	0.836406	1.381697	-0.535400
37	6	0	2.719214	2.406883	-1.956700
38	6	0	2.499224	3.845185	-1.562900
39	6	0	3.356031	4.875679	-1.658100
40	6	0	4.778730	4.782869	-2.155000
41	6	0	2.925941	6.271082	-1.268900
42	8	0	-1.386005	-0.159688	-1.245800
43	1	0	2.147315	2.661287	1.580000
44	1	0	3.725312	2.147876	2.194500
45	1	0	3.620820	3.276677	0.823200
46	1	0	4.702596	-0.002031	-0.696500
47	1	0	5.199301	0.586966	0.901800

48	1	0	5.165009	1.700766	-0.475800
49	1	0	-5.706971	4.560743	-0.586000
50	1	0	-5.655874	4.124643	1.134400
51	1	0	-6.340182	3.005548	-0.062400
52	1	0	-3.940479	3.387231	-2.222900
53	1	0	-3.021289	2.002324	-1.590800
54	1	0	-4.769190	1.901937	-1.775400
55	1	0	-3.589679	3.487328	1.866000
56	1	0	-3.628905	-0.180872	3.039900
57	1	0	-3.466799	0.671627	4.671000
58	1	0	-0.428087	2.274706	4.059000
59	1	0	-1.917586	2.421816	5.002800
60	1	0	-1.662379	3.429414	3.569600
61	1	0	-1.180880	3.273111	1.245700
62	1	0	-1.591887	2.307614	-0.165100
63	1	0	-2.384400	0.472620	1.223200
64	1	0	-0.327106	-0.285195	2.333400
65	1	0	0.410606	1.297500	2.176600
66	1	0	-2.989832	-4.035676	-2.685100
67	1	0	-5.217128	-3.460760	-3.626300
68	1	0	-6.604216	-1.704850	-2.539400
69	1	0	-5.742407	-0.519957	-0.527500
70	1	0	-3.497111	-1.048873	0.353200
71	1	0	-0.366235	-4.402495	0.290200
72	1	0	3.372682	-2.070621	2.108900
73	1	0	2.433171	-3.559715	2.124100
74	1	0	4.118279	-2.506827	-0.246600
75	1	0	5.763158	-5.459138	-0.726500
76	1	0	5.256568	-3.982635	-1.571200
77	1	0	4.384657	-5.509229	-1.819400
78	1	0	2.718557	-5.513817	1.597100
79	1	0	4.264351	-6.328528	1.277400
80	1	0	2.944251	-6.452818	0.119700
81	1	0	2.466499	0.359885	-1.463300
82	1	0	0.210606	1.407601	-1.431600
83	1	0	0.646813	2.310298	0.005200
84	1	0	3.755212	2.239776	-2.266200
85	1	0	2.109112	2.204688	-2.850700
86	1	0	1.495025	4.075092	-1.205700
87	1	0	4.911535	5.394068	-3.058500

88	1	0	5.477233	5.183364	-1.407300
89	1	0	5.092523	3.764666	-2.396500
90	1	0	1.889541	6.297789	-0.918100
91	1	0	3.014546	6.962881	-2.118300
92	1	0	3.566344	6.675777	-0.472500

Conformer 14a-3

Center	Atomic	Atomic	Coordinates (Angstroms)		
Number	Number	Type	X	Y	Z
1	8	0	2.909503	0.041773	0.978301
2	6	0	-0.358823	5.960114	-1.595099
3	6	0	2.052980	6.273184	-0.868699
4	6	0	0.740172	5.561500	-0.640099
5	6	0	0.612760	4.658302	0.345101
6	6	0	-0.611249	3.858917	0.719201
7	6	0	-2.128880	1.386636	2.031301
8	6	0	-3.069871	2.156948	-0.175999
9	6	0	-5.529923	-2.084822	-0.819699
10	6	0	-5.787820	-1.805419	0.524301
11	6	0	-4.729419	-1.725532	1.433301
12	6	0	-3.417121	-1.896548	0.995101
13	6	0	-3.152324	-2.173151	-0.352399
14	6	0	-4.220426	-2.289338	-1.251199
15	6	0	-1.772529	-2.513569	-0.828499
16	8	0	-1.683941	-3.519670	-1.566599
17	6	0	-0.568620	-1.794984	-0.398799
18	6	0	-0.545403	-0.435384	0.038701
19	6	0	0.625905	0.173802	0.479401
20	6	0	1.805395	-0.596713	0.507301
21	6	0	1.865879	-1.921314	0.051901
22	6	0	0.683372	-2.487499	-0.451099
23	8	0	0.762656	-3.734300	-0.946999
24	8	0	-1.705994	0.258631	-0.078599
25	6	0	-1.843378	1.580132	0.534801
26	6	0	-0.552468	2.384416	0.245301
27	6	0	0.663323	1.643401	0.820701
28	6	0	3.148369	-2.737130	0.102601
29	6	0	4.346177	-2.046045	-0.512299
30	6	0	5.587479	-1.907360	-0.006999

31	6	0	6.668787	-1.214373	-0.803399
32	6	0	6.039672	-2.435266	1.333801
33	1	0	3.703997	-0.473137	0.730701
34	1	0	-0.058226	5.759710	-2.632799
35	1	0	-1.301430	5.437226	-1.416799
36	1	0	-0.554510	7.039816	-1.533699
37	1	0	1.934294	7.361985	-0.774699
38	1	0	2.431778	6.089579	-1.883999
39	1	0	2.820777	5.953374	-0.157199
40	1	0	1.494858	4.465791	0.956301
41	1	0	-1.511244	4.332428	0.316101
42	1	0	-0.714849	3.877918	1.813401
43	1	0	-3.051387	0.813347	2.159301
44	1	0	-2.254468	2.352137	2.532101
45	1	0	-1.319087	0.846126	2.530201
46	1	0	-3.392059	3.093352	0.288801
47	1	0	-3.896380	1.442358	-0.117199
48	1	0	-2.853768	2.345045	-1.232999
49	1	0	-6.350424	-2.157212	-1.528599
50	1	0	-6.809918	-1.661306	0.864801
51	1	0	-4.927616	-1.533129	2.484601
52	1	0	-2.594320	-1.835558	1.700801
53	1	0	-4.011029	-2.541141	-2.285999
54	1	0	-0.123646	-3.923589	-1.366099
55	1	0	-0.449468	2.397015	-0.849099
56	1	0	0.714825	1.793101	1.910101
57	1	0	1.584828	2.080090	0.423601
58	1	0	2.961957	-3.669027	-0.441699
59	1	0	3.359765	-3.029932	1.139301
60	1	0	4.161082	-1.635842	-1.506499
61	1	0	7.085698	-0.362379	-0.249099
62	1	0	6.298392	-0.848269	-1.765699
63	1	0	7.506879	-1.897684	-0.998699
64	1	0	5.230367	-2.865656	1.927701
65	1	0	6.512682	-1.640672	1.926301
66	1	0	6.801763	-3.214775	1.196801

Conformer 14a-4

Center	Atomic	Atomic	Coordinates (Angstroms)		
Number	Number	Type	X	Y	Z
1	8	0	-2.938161	0.321529	0.778471

2	6	0	0.602877	6.166269	-0.517004
3	6	0	-1.850305	6.020967	-1.155982
4	6	0	-0.567982	5.278219	-0.863294
5	6	0	-0.520876	3.937456	-0.920713
6	6	0	0.668321	3.043794	-0.667123
7	6	0	2.032699	1.038458	2.447340
8	6	0	3.268107	2.078017	0.534250
9	6	0	4.432051	-2.175480	1.569429
10	6	0	5.576423	-2.211323	0.768285
11	6	0	5.450364	-2.241512	-0.622484
12	6	0	4.185882	-2.241584	-1.208795
13	6	0	3.035778	-2.168131	-0.412330
14	6	0	3.168175	-2.142591	0.982011
15	6	0	1.695974	-2.285301	-1.073505
16	8	0	1.611054	-3.132516	-1.990018
17	6	0	0.511601	-1.542039	-0.631758
18	6	0	0.545654	-0.282395	0.040023
19	6	0	-0.613857	0.339970	0.496162
20	6	0	-1.843499	-0.316248	0.285632
21	6	0	-1.958708	-1.516251	-0.430765
22	6	0	-0.777355	-2.090118	-0.927353
23	8	0	-0.901446	-3.197855	-1.677305
24	8	0	1.761953	0.316137	0.139481
25	6	0	1.925229	1.496846	0.983112
26	6	0	0.723130	2.452995	0.769736
27	6	0	-0.570010	1.704982	1.144642
28	6	0	-3.309920	-2.129693	-0.765465
29	6	0	-4.219640	-2.306371	0.430687
30	6	0	-5.515498	-1.960318	0.559685
31	6	0	-6.343574	-1.291352	-0.511711
32	6	0	-6.269192	-2.265866	1.833228
33	1	0	-3.703590	-0.286447	0.738979
34	1	0	0.382403	6.764832	0.378032
35	1	0	0.800012	6.883499	-1.326096
36	1	0	1.525866	5.612833	-0.327243
37	1	0	-2.159317	6.633689	-0.297185
38	1	0	-1.723116	6.713454	-2.000310
39	1	0	-2.671075	5.338590	-1.398218
40	1	0	-1.444700	3.421755	-1.183198
41	1	0	1.593501	3.595209	-0.854837

42	1	0	0.651854	2.218636	-1.390391
43	1	0	2.914873	0.404744	2.574638
44	1	0	1.155955	0.466569	2.762067
45	1	0	2.131211	1.905687	3.110369
46	1	0	3.482534	3.005395	1.077339
47	1	0	4.068743	1.361320	0.741101
48	1	0	3.277127	2.288092	-0.537499
49	1	0	4.524810	-2.176589	2.652471
50	1	0	6.561770	-2.226453	1.226804
51	1	0	6.337205	-2.278453	-1.249520
52	1	0	4.075195	-2.298359	-2.287037
53	1	0	2.278630	-2.115944	1.603793
54	1	0	0.010848	-3.397555	-2.032096
55	1	0	0.848318	3.293905	1.464483
56	1	0	-0.651664	1.615904	2.236316
57	1	0	-1.443105	2.288392	0.841297
58	1	0	-3.121262	-3.111275	-1.213005
59	1	0	-3.804863	-1.535400	-1.544577
60	1	0	-3.747427	-2.803335	1.279739
61	1	0	-6.830689	-0.388270	-0.120144
62	1	0	-5.767079	-1.009908	-1.395617
63	1	0	-7.150685	-1.960503	-0.840430
64	1	0	-6.688745	-1.351940	2.275636
65	1	0	-5.631974	-2.746963	2.581442
66	1	0	-7.119873	-2.931874	1.632228

3. Experimental and Computed NMR Chemical Shifts

3.1 ^{13}C- and ^{1}H-NMR chemical shifts

The TMS-corrected computed ^{13}C- and ^{1}H-NMR chemical shifts of compound **1** was fitted to the experimental values by Ordinary Least Squares (OLS) Linear Regression method in order to remove systematic error that results from the conformational search and random error from experimental conditions (**Table S4**).

Table S4 Experimental and computed chemical shifts of **1**, **1a** and **1b**.

Position	Exp.**1**	**1a**	Residue	**1b**	Residue
1	79.3	78.6312	0.668799	79.10126	0.198736
2	193	190.1998	2.800182	189.548	3.45197

3	124.7	123.6892	1.010848	121.1516	3.548442
4	167.3	169.8642	-2.56423	169.4701	-2.17013
5	51.2	55.87211	-4.67211	52.50057	-1.30057
6	40.7	42.59626	-1.89626	43.82515	-3.12515
7	42.8	42.00988	0.79012	42.55668	0.243315
8	48	52.66118	-4.66118	53.01461	-5.01461
9	208.4	207.6405	0.759548	208.7908	-0.39082
10	193.9	193.2381	0.661926	193.2734	0.626643
11	137	133.043	3.957025	133.2221	3.777878
12	128.4	127.999	0.401046	128.4998	-0.09979
13	128	125.3296	2.670407	125.7245	2.275495
14	132.1	131.0094	1.090616	131.3153	0.784662
15	128	125.3296	2.670407	125.7245	2.275495
16	128.4	127.999	0.401046	128.4998	-0.09979
17	22	23.58446	-1.58446	23.52147	-1.52147
18	121.4	121.1743	0.225651	121.1428	0.257246
19	132	135.3881	-3.38811	136.0553	-4.05526
20	18.1	16.9918	1.108196	17.46392	0.636077
21	26	25.65243	0.347572	26.11216	-0.11216
22	29.1	26.3814	2.718596	24.15882	4.941183
23	40	43.25089	-3.25089	43.24097	-3.24097
24	84.2	85.21627	-1.01627	82.58739	1.61261
25	28.4	27.45977	0.940232	26.61992	1.780079
26	21.4	19.80179	1.598213	26.24971	-4.84971
27	30.2	30.63679	-0.43679	26.5906	3.609404
28	121.5	122.6686	-1.16861	123.0176	-1.51757
29	134.4	136.9878	-2.58782	136.7623	-2.36228
30	18.2	16.96342	1.236581	16.75979	1.440209
31	26.1	25.53438	0.565619	25.73611	0.363888
32	26.9	27.76519	-0.86519	28.03312	-1.13312
33	122.8	124.1521	-1.35214	124.0533	-1.25326
34	133.4	135.8991	-2.49908	136.68	-3.27996
35	18.1	16.9189	1.181101	17.30072	0.799285
36	26	25.5929	0.407104	25.95824	0.041763
37	23.2	21.07653	2.123474	21.71536	1.48464
38	16.3	14.69117	1.608826	14.9224	1.377598

Table S5 Statistics of Ordinary Least Squares Linear Regression (OLS-LR) of experimental and computed ^{13}C-NMR chemical shifts.

Compound	CMAD[a]	CLAD[b]	R^2	RMSD	*F*	*p* value
**1a**	1.68	4.67	0.9988	2.1181	29345.94	< 0.01
**1b**	1.87	5.01	0.9984	2.4532	21867.46	< 0.01

[a]CMAD = corrected mean absolute deviation, computed as $(1/n)\sum_{i}^{n}|\delta_{\mathrm{calc}}-\delta_{\mathrm{exp}}|$, where δ_{calc} and δ_{exp} refer to the calculated and experimental chemical shifts.

[b]CLAD = corrected largest absolute deviation, computed as $\max(|\delta_{\mathrm{calc}}-\delta_{\mathrm{exp}}|)$.

3.2 DP4+ analysis

3.2.1 DP4+ analysis of compound **1** with two possible structures of **1a** and **1b**.

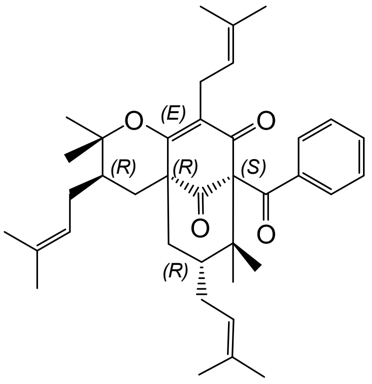

1a

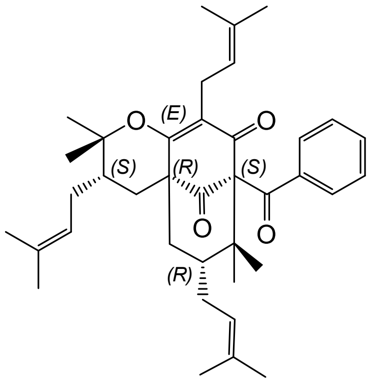

1b

(**DP4**+: 100.00%)-**1a** (**DP4**+: 0.00%)-**1b**

Figure S154 DP4+ probability for compounds **1a** and **1b** based on its ^{1}H and ^{13}C-NMR data

Functional	Solvent?	Basis Set	Type of Data
mPW1PW91	PCM	6-311+G(d,p)	Unscaled Shifts

	Isomer 1	Isomer 2	Isomer 3	Isomer 4	Isomer 5	Isomer 6	I
sDP4+ (H data)	99.98%	0.02%	-	-	-	-	
sDP4+ (C data)	99.91%	0.09%	-	-	-	-	
sDP4+ (all data)	100.00%	0.00%	-	-	-	-	
uDP4+ (H data)	96.73%	3.27%	-	-	-	-	
uDP4+ (C data)	99.82%	0.18%	-	-	-	-	
uDP4+ (all data)	99.99%	0.01%	-	-	-	-	
DP4+ (H data)	100.00%	0.00%	-	-	-	-	
DP4+ (C data)	100.00%	0.00%	-	-	-	-	
DP4+ (all data)	100.00%	0.00%	-	-	-	-	

Functional	Solvent?	Basis Set	Type of Data
mPW1PW91	PCM	6-311+G(d,p)	Unscaled Shifts

Nuclei	sp2?	Experimental	Isomer 1	Isomer 2	Isomer 3	Isomer 4	Isomer 5
		DP4+	100.00%	0.00%	–	–	–
C		79.3	83.4	83.5			
C	x	193	200.4	199.4			
C	x	124.7	130.6	127.6			
C	x	167.3	179.0	178.3			
C		51.2	59.5	55.6			
C		40.7	45.6	46.5			
C		42.8	45.0	45.1			
C		48	56.1	56.1			
C	x	208.4	218.7	219.6			
C	x	193.9	203.6	203.3			
C	x	137	140.4	140.3			
C	x	128.4	135.14	135.33			
C	x	128	132.34	132.42			
C	x	132.1	138.29	138.29			
C	x	128	132.34	132.42			
C	x	128.4	135.14	135.33			
C		22	25.63	25.16			
C	x	121.4	127.98	127.61			
C	x	132	142.89	143.26			
C		18.1	18.71	18.80			
C		26	27.80	27.88			
C		29.1	28.56	25.83			
C		40	46.25	45.85			
C		84.2	90.27	87.15			
C		28.4	29.69	28.41			
C		21.4	21.66153	28.02237			
C		30.2	33.02498	28.38013			
C	x	121.5	129.5453	129.5802			
C	x	134.4	144.5629	144.0053			
C		18.2	18.68473	18.0627			
C		26.1	27.67371	27.48335			
C		26.9	30.01332	29.89407			
C	x	122.8	131.1012	130.6672			
C	x	133.4	143.4211	143.9189			
C		18.1	18.63804	18.6304			
C		26	27.73508	27.71647			
C		23.2	22.99844	23.26357			
C		16.3	16.30166	16.13436			
H		1.28	1.425237	1.512908			
H		2.27	2.056587	2.168448			
H		1.62	1.797224	1.830541			
H	x	7.52	8.132675	8.144635			
H	x	7.24	7.652489	7.584945			
H	x	7.39	7.828015	7.781073			
H	x	7.24	7.652489	7.584945			
H	x	7.52	8.132675	8.144635			
H		3.08	2.844069	2.819454			
H		3.13	3.569851	3.67463			
H	x	5.09	5.665017	5.720924			
H		1.67	1.78784	1.709828			
H		1.64	1.658201	1.541112			
H		1.68	0.77818	1.411078			
H		1.99	3.215701	3.363966			
H		1.85	1.587381	1.683796			
H		1.55	1.522744	1.559166			
H		1.2	1.339778	1.474254			
H		1.8	2.035188	1.462161			
H		2.17	2.086592	2.570202			
H	x	5.03	5.300046	5.693153			
H		1.63	1.641085	1.613762			
H		1.74	1.698995	1.610756			
H		2.21	2.117685	2.212147			
H		1.7	1.924701	1.924481			
H	x	5	5.296184	5.357876			
H		1.58	1.611179	1.618588			
H		1.72	1.724018	1.719915			
H		1.35	1.315343	1.396779			
H		1.15	1.155048	1.199381			

4. Experimental and calculated ECD spectra

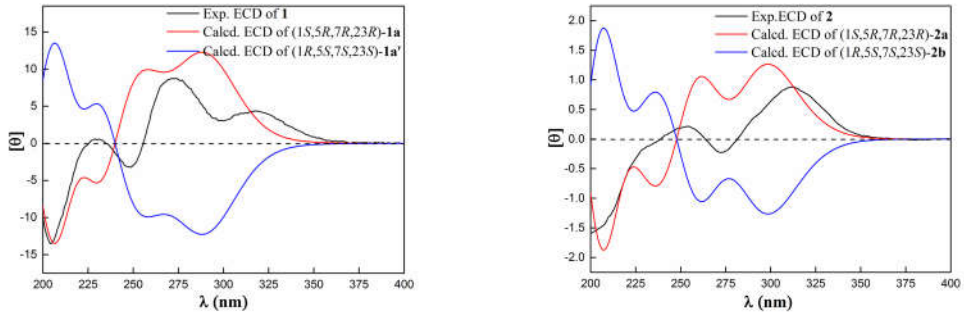

Figure S155 Calculated ECD spectra of compounds **1** and **2** was compared with the experimental.

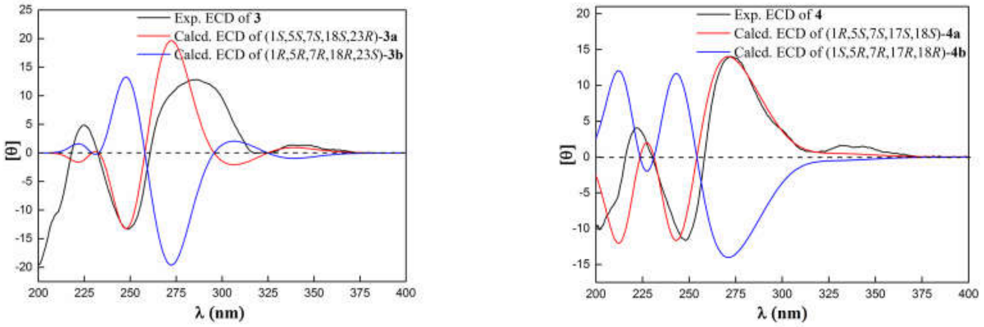

Figure S156 Calculated ECD spectra of compounds **3** and **4** was compared with the experimental.

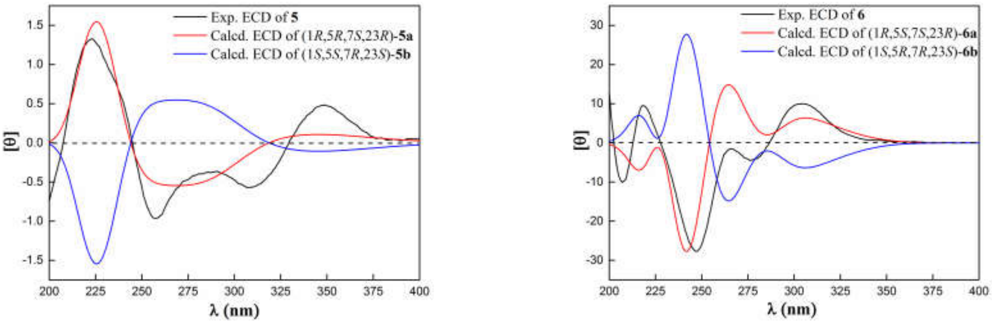

Figure S157 Calculated ECD spectra of compounds **5** and **6** was compared with the experimental.

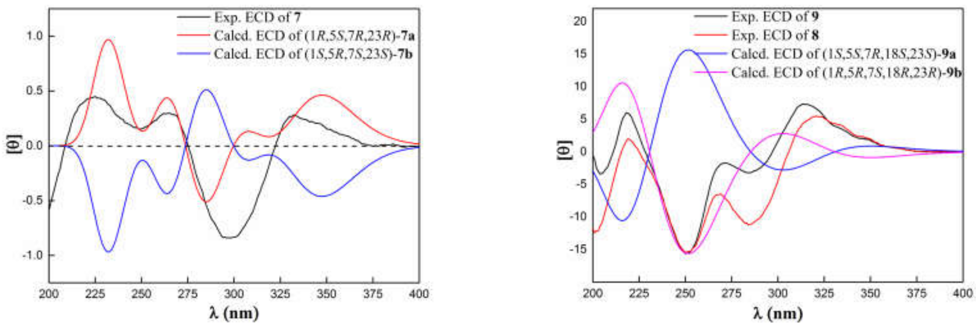

Figure S158 Calculated ECD spectra of compounds **7**, **8** and **9** was compared with the experimental.

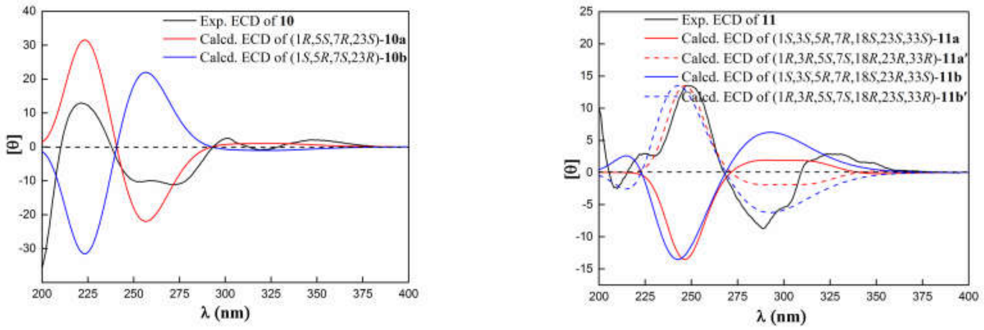

Figure S159 Calculated ECD spectra of compounds **10** and **11** was compared with the experimental.

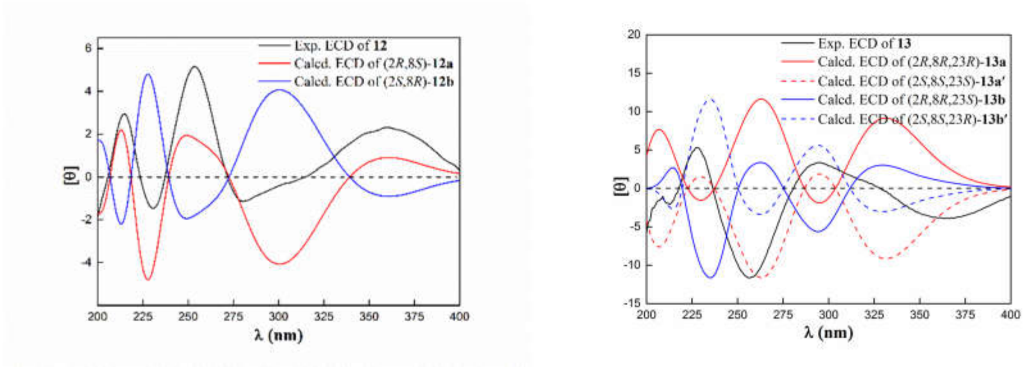

Figure S160 Calculated ECD spectra of compounds **12** and **13** was compared with the experimental.

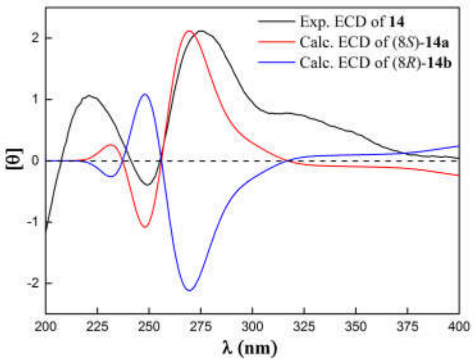

Figure S161 Calculated ECD spectra of compound **14** was compared with the experimental.
